# Six pairs of enantiomeric prenylated flavonoids with cytotoxic activities from *Epimedium sagittatum* Maxim

**DOI:** 10.1007/s13659-025-00510-1

**Published:** 2025-05-13

**Authors:** Shuang-Shuang Xie, Xiang Yu, Qi-Mei Tie, Jing-Ke Zhang, Bei-Bei Zhang, Meng-Nan Zeng, Xiao-Ke Zheng, Wei-Sheng Feng

**Affiliations:** 1 School of Pharmacy, Henan University of Chinese Medicine, Zhengzhou, 450046 People’s Republic of China; 2 The Engineering and Technology Center for Chinese Medicine Development of Henan Province, Zhengzhou, 450046 People’s Republic of China

**Keywords:** *Epimedium sagittatum* Maxim, Enantiomeric prenylated flavonoids, ECD, Cytotoxicity, Sphk1

## Abstract

In this work, six pairs of undescribed enantiomeric prenylated flavonoids, ( ±)-epimesatines J–O (**1a/1b–6a/6b**), were isolated from the aerial parts of *Epimedium sagittatum* Maxim. Their structures and absolute configurations were determined based on spectroscopic data, quantum chemical calculations of electronic circular dichroism (ECD) and ^13^C NMR, as well as ECD experiments induced by Mo_2_(OAc)_4_ and Rh_2_(OCOCF_3_)_4_. The cytotoxicity assay revealed that compounds** 1a/1b, 2a/2b**, and** 4a/4b–6a/6b** demonstrated significant inhibitory effects on the viability of human breast cancer cells MCF-7 while exhibiting no obvious toxicity towards human breast epithelial cells MCF-10A. Additionally, these compounds were found to decrease the expression of sphingosine kinase 1 (Sphk1) in MCF-7 cells. Notably, compounds** 4a** and** 5b** exhibited IC_50_ values of 7.45 and 8.97 μM, respectively, in MCF-7 cells.

**Graphical Abstract:**

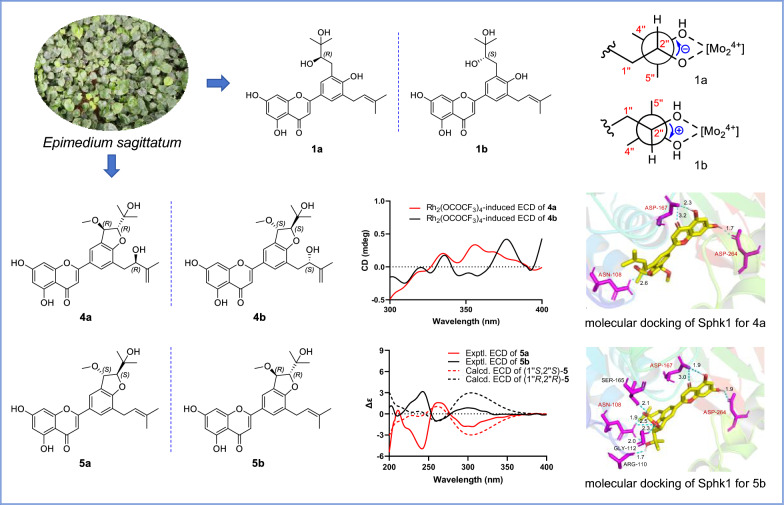

**Supplementary Information:**

The online version contains supplementary material available at 10.1007/s13659-025-00510-1.

## Introduction

Flavonoids represent a significant class of natural specialised metabolites widely distributed in plants, showcasing diverse biological properties such as cytotoxicity, anti-inflammatory, antiviral, antimicrobial, and antioxidant activities [1–5]. Prenylated flavonoids are characterized by the prenyl side chains in the flavonoid skeleton, resulting in enhanced lipophilicity and structural diversity compared to nonprenylated forms [6, 7]. Notably, prenylated flavonoids have demonstrated promising pharmacological properties, particularly in terms of their potential cytotoxic effects against various types of cancer cells [8, 9].

*Epimedium sagittatum* Maxim. (Berberidaceae) is a traditional Chinese medicine with a long history of use in treating various conditions such as sexual dysfunction, osteoporosis, cardiovascular diseases, asthma, and chronic nephritis [10, 11]. Flavonoids have been identified as the primary bioactive constituents of this plant, with several isolated from *E. sagittatum* demonstrating efficacy against various types of cancer cells [11–14]. Notably, icaritin, a prenylated flavonoid derived from *E. sagittatum*, has been commercialized as an anti-hepatocellular carcinoma drug [15, 16].

**Fig. 1:**
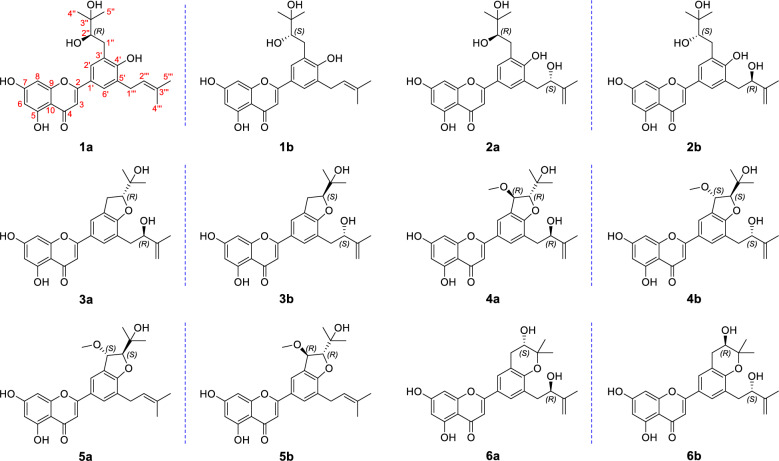
Chemical structures of compounds **1a**/**1b**–**6a/6b**

In our ongoing dedication to the exploration of structurally fascinating prenylated flavonoids with anticancer properties derived from *E. sagittatum* [17, 18], six pairs of enantiomers, designated as ( ±)-epimesatines J–O, were isolated and characterized (Fig. 1). The elucidation of their structures with absolute configurations, which was a challenging task, was successfully accomplished by means of spectroscopic techniques (HRESIMS and NMR), quantum chemical calculations (ECD and ^13^C NMR), and chemical methods (ECD experiments induced by Mo_2_(OAc)_4_ and Rh_2_(OCOCF_3_)_4_ transition metal complexes) [19–21]. The evaluation of cell viability of the isolated compounds on human breast cancer cells MCF-7 was conducted by MTT method, and their effects on human breast epithelial cells MCF-10A were also evaluated. Furthermore, the impact of these compounds on the expression levels of sphingosine kinase 1 (Sphk1), an enzyme implicated in cancer development [22], was investigated in MCF-7 cells. Herein, the isolation, structural elucidation, and biological activity evaluation of these metabolites are described.

## Results and discussion

**Fig. 2:**
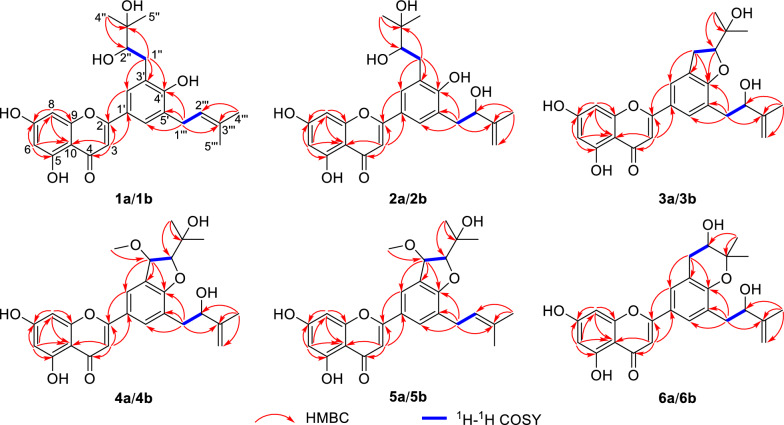
^1^H–^1^H COSY and Key HMBC correlations of compounds **1a**/**1b**–**6a**/**6b**

Compound **1a** was obtained as a yellow amorphous powder. The molecular formula of** 1a** was established as C_25_H_28_O_7_ based on the high–resolution electrospray ionization mass spectrometric (HRESIMS) (*m/z* 463.1725 [M + Na]^+^, calcd. for 463.1727) and ^13^C NMR data, indicating twelve degrees of unsaturation. The ^1^H NMR data of 1a (Table 1) revealed the presence of six olefinic/aromatic protons (*δ*_H_ 7.71, 7.69, 6.60, 6.50, 6.24, 5.38; 1H each), one oxygenated methine (*δ*_H_ 3.71, 1H), two methylene groups (*δ*_H_ 3.40, 2H, d, *J* = 7.3 Hz; 3.02, 1H, d, *J* = 14.5 Hz and 2.87, 1H, dd, *J* = 14.5, 9.9 Hz), and four methyl singlets (*δ*_H_ 1.75, 1.74, 1.30, and 1.28). The ^13^C NMR and DEPT-135 spectra of **1a** indicated 25 carbon signals, including four methyls (*δ*_C_ 25.9, 25.5, 25.2, and 17.9), two methylenes (*δ*_C_ 35.2 and 29.4), seven methines (including one oxygenated, *δ*_C_ 81.7), and twelve nonprotonated carbons (including one ketone carbonyl, *δ*_C_ 182.9). Based on the above data, compound **1a** was identified as a prenylated flavone derivative [23–25]. The ^1^H − ^1^H COSY interaction (Fig. 2) between H_2_-1″ (*δ*_H_ 3.02 and 2.87) and H-2″ (*δ*_H_ 3.71), along with HMBC correlations (Fig. 2) from Me-4″/Me-5″ to C-2″ (*δ*_C_ 81.7) and C-3″ (*δ*_C_ 72.7), confirmed the presence of a 2,3-dihydroxy-3-methylbutyl unit.

**Table 1:** ^1^H NMR spectroscopic data for compounds **1a/1b**–**6a/6b** (*δ* in ppm, *J* in Hz)

No	1a/1b^*a*^	2a/2b^*a*^	3a/3b^*a*^	4a/4b^*a*^	5a/5b^*a*^	6a/6b^*a*^
3	6.60 s	6.62 s	6.59 s	6.67 s	6.65 s	6.58 s
6	6.24 s	6.25 s	6.24 d (2.1)	6.26 d (2.1)	6.26 d (2.0)	6.24 s
8	6.50 s	6.52 s	6.52 d (2.1)	6.56 d (2.1)	6.54 d (2.0)	6.53 s
2′	7.71 d (2.3)	7.77 d (2.4)	7.73^*b*^	7.99 d (2.0)	7.99 d (1.9)	7.62 d (2.5)
6′	7.69 d (2.3)	7.75 d (2.4)	7.74^*b*^	7.92 d (2.0)	7.83 d (1.9)	7.69 d (2.5)
1″	3.02 d (14.5)	3.07 dd (14.2, 1.9)	3.37 dd (15.9, 7.7)	5.14 d (2.8)	5.14 d (2.7)	3.11 dd (16.6, 5.1)
	2.87 dd (14.5, 9.9)	2.75 dd (14.2, 10.0)	3.29 dd (15.9, 9.6)			2.83 dd (16.6, 7.4)
2″	3.71 d (9.9)	3.69 dd (10.0, 1.9)	4.78 dd (9.6, 7.7)	4.49 d (2.8)	4.49 d (2.7)	3.87 dd (7.4, 5.1)
4″	1.28 s	1.28 s	1.26 s	1.33 s	1.33 s	1.42 s
5″	1.30 s	1.26 s	1.29 s	1.22 s	1.15 s	1.34 s
1$1^{\prime\prime\prime}$	3.40 d (7.3)	3.00 dd (14.1, 3.7)	2.97 dd (13.8, 4.8)	2.97 dd (13.7, 4.9)	3.41 d (7.5)	3.03 dd (13.3, 4.6)
		2.93 dd (14.1, 8.2)	2.77 dd (13.8, 8.2)	2.86 dd (13.7, 8.5)		2.67 dd (13.3, 8.2)
$2^{\prime\prime\prime}$	5.38 m	4.44 dd (8.2, 3.7)	4.46 dd (8.2, 4.8)	4.42 dd (8.5, 4.9)	5.38 m	4.37 dd (8.2, 4.6)
$4^{\prime\prime\prime}$	1.74 s	4.97 s	4.91 s	4.92 s	1.74 s	4.90 s
		4.78 s	4.75 s	4.76 s		4.73 s
$5^{\prime\prime\prime}$	1.75 s	1.83 s	1.83 s	1.84 s	1.77 s	1.83 s
OC*H*_*3*_				3.48 s	3.48 s	

^*a*^Recorded in acetone-*d*_6_ at 500 MHz

^*b*^Signals partially overlapped

In addition, the spin coupling system of H_2_-$1^{\prime\prime\prime}$/H-$2^{\prime\prime\prime}$ according to the ^1^H − ^1^H COSY spectrum, together with the HMBC correlations from Me-$4^{\prime\prime\prime}$/Me-$5^{\prime\prime\prime}$ to C-$2^{\prime\prime\prime}$ and C-$3^{\prime\prime\prime}$, indicated the existence of a prenyl group. Then, the key HMBC correlations from H_2_-1″ to C-2′, C-3′, and C-4′, and from H_2_-$1^{\prime\prime\prime}$ to C-4′, C-5′, and C-6′ verified the location of these two substituents at C-3′ and C-5′, respectively. Consequently, the planar structure of **1a** was established as 5,7,4′-trihydroxy-3′-(2,3-dihydroxy-3-methylbutyl)-5′-prenylflavone.

**Fig. 3:**
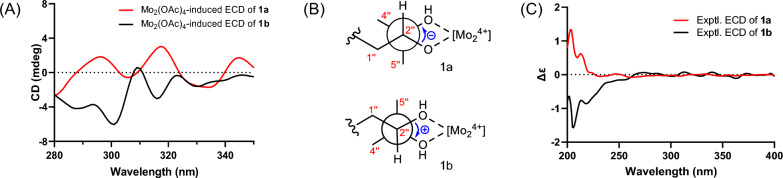
**A** ICD spectrum of the Mo_2_^4+^ complex of** 1a** and** 1b** in DMSO. **B** Conformation of the Mo_2_^4+^ complex of** 1a** and** 1b**. **C** Experimental ECD spectra of compounds **1a** and **1b**

The Mo_2_(OAc)_4_-induced circular dichroism (ICD) experiment represents a highly valuable method for determining the absolute configuration of carbon atoms in the vicinal diol subunit, based on the helicity rule proposed by Snatzke [26–28]. According to the empirical rule, the observed ICD curve at approximately 310 nm corresponds to the same torsion angle of the (HO)-C–C-(OH) moiety. Specifically, a positive CD band corresponds to a positive torsional angle, while a negative CD band corresponds to a negative torsional angle. Consequently, the ICD curve of **1a** exhibits a negative Cotton effect at 306 nm (Fig. 3A), suggesting a negative torsional angle (Fig. 3B), and thus deducing an *R* configuration of C-2″. Therefore, the structure and absolute configuration of 1a was conclusively defined and assigned the trivial name (*R*)-epimesatine J.

A comparison of the ^1^H NMR data of** 1b ** with those of** 1a** indicated that they shared the same planar structure. Given the presence of a chiral center in compound** 1a**, a comparative analysis of optical rotation and ECD spectra between compounds** 1a** and** 1b** was conducted. Their mirror-image ECD spectra (Fig. 3C) and opposite-sign optical rotation values ([*α*]_D_^20^: − 4 for **1a **and + 6 for **1b**) demonstrated that compound** 1b** is the enantiomer of compound** 1a**. Moreover, further support for this conclusion came from the Mo_2_(OAc)_4_-induced ECD spectrum of** 1b**, which demonstrated a positive Cotton effect at 309 nm (Fig. 3A), indicating a positive torsional angle (Fig. 3B), and leading to the deduction of an *S* configuration of C-2″. Thus, we determined that** 1b** is indeed an enantiomer of 1a and named it (*S*)-epimesatine J.

Compound** 2a** was isolated as a yellow amorphous powder, and the molecular formula of** 2a** was identified as C_25_H_28_O_8_ based on its HRESIMS and NMR data, indicating twelve degrees of unsaturation. The ^1^H and ^13^C NMR data (Tables 1 and 2) of 2a were similar to those of 1a/1b, suggesting that compound 2a was also a flavone derivative. The primary difference between** 2** and** 1a/1b** was the absence of a methyl signal and the presence of the signals of a methylene group [*δ*_H_ 4.97, 4.78 (each 1H, s, H_2_-$4^{\prime\prime\prime}$)] in** 2a**, indicating the replacement of the prenyl group in 1a/1b with a 2-hydroxy-3-methylbut-3-enyl moiety. The ^1^H − ^1^H COSY correlation of H_2_-$1^{\prime\prime\prime}$ (*δ*_H_ 3.00, 1H, dd, *J* = 14.1, 3.7 Hz and 2.93, 1H, dd, *J* = 14.1, 8.2 Hz)/H-$2^{\prime\prime\prime}$ (*δ*_H_ 4.44, 1H, dd, *J* = 8.2, 3.7 Hz), along with the HMBC correlations (Fig. 2) from Me-$5^{\prime\prime\prime}$ to C-$2^{\prime\prime\prime}$, C-$3^{\prime\prime\prime}$, and C-$4^{\prime\prime\prime}$ support above speculation. Additionally, the key HMBC correlations from H_2_-$1^{\prime\prime\prime}$ to C-4′, C-5′, and C-6′ confirmed the positioning of the substituent at C-5′. Similarly to 1b, 2b was identified as an enantiomer of 2a.

**Table 2:** ^13^C NMR spectroscopic data for compounds** 1a/1b–6a/6b **(*δ* in ppm)

No	1a/1b^*a*^	2a/2b^*a*^	3a/3b^*a*^	4a/4b^*a*^	5a/5b^*a*^	6a/6b^*a*^
2	165.4	165.6	165.5	165.2	165.3	165.3
3	104.1	104.0	104.1	104.5	104.5	104.2
4	182.9	182.9	182.9	183.0	183.0	182.9
5	163.1	163.1	163.0	163.1	163.0	163.0
6	99.6	99.5	99.7	99.7	99.8	99.6
7	164.8	164.8	165.1	164.9	165.1	164.9
8	94.7	94.7	94.8	94.9	94.9	94.8
9	158.8	158.8	158.8	158.9	158.9	158.8
10	105.3	105.3	105.1	105.3	105.1	105.2
1′	123.0	122.5	123.9	124.2	124.5	122.9
2′	128.4	129.0	122.0	123.5	123.3	127.4
3′	128.7	129.3	129.4	128.7	128.9	121.5
4′	158.9	159.7	162.9	163.7	163.2	155.6
5′	130.8	128.2	122.3	123.6	125.5	129.0
6′	127.2	129.1	129.6	131.3	129.7	128.4
1″	35.2	34.7	30.8	81.9	81.8	32.2
2″	81.7	80.3	91.0	95.9	96.1	69.3
3″	72.7	72.8	71.5	71.1	71.0	79.2
4″	25.5	25.6	26.2	26.4	26.9	26.1
5″	25.2	25.4	25.3	25.1	24.6	21.4
$1^{\prime\prime\prime}$	29.4	38.8	37.2	36.7	29.0	37.9
$2^{\prime\prime\prime}$	123.4	76.4	74.9	75.4	122.3	75.1
$3^{\prime\prime\prime}$	132.9	148.6	149.0	149.0	133.6	149.3
$4^{\prime\prime\prime}$	25.9	110.7	110.5	110.7	25.9	110.2
$5^{\prime\prime\prime}$	17.9	18.3	18.2	18.1	18.0	18.3
O*C*H_3_				55.9	56.0	

^*a*^Recorded in acetone-*d*_6_ at 125 MHz

**Fig. 4:**
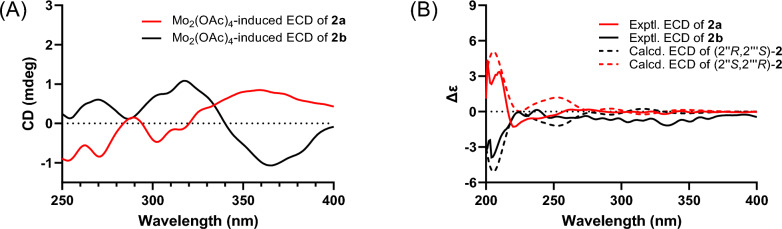
**A** Mo_2_(OAc)_4_-induced ECD spectra of compounds** 2a** and** 2b**. **B** Experimental and calculated ECD spectra of compounds **2a** and **2b**

Due to the existence of a vicinal diol moiety in their side chains, the Mo_2_(OAc)_4_-induced ECD experiments were conducted to determine the absolute configuration of C-2″ in both compounds 2a and 2b, following the same approach as that for** 1a/1b**. Based on these experiments (Fig. 4A), the configurations of C-2″ in** 2a** and** 2b** were assigned as *R* and *S*, respectively. Additionally, the absolute configurations of C-$2^{\prime\prime\prime}$ in** 2a ** and ** 2b** were elucidated by performing ECD calculations at the B3LYP/6-31G(d) level in MeOH. As shown in Fig. 4B, the experimental ECD spectra of** 2a** and** 2b** show reasonable alignment with the calculated conformations of (2″*R*,2${\prime\prime\prime}$*S*)-**2** and (2″*S*,** 2**${\prime\prime\prime}$*R*)-2, respectively. These computational correlations support the assignment of** 2a** and** 2b** as (2″*R*,2${\prime\prime\prime}$*S*)-epimesatine K and (2″*S*,2${\prime\prime\prime}$*R*)-epimesatine K, respectively.

**Fig. 5:**
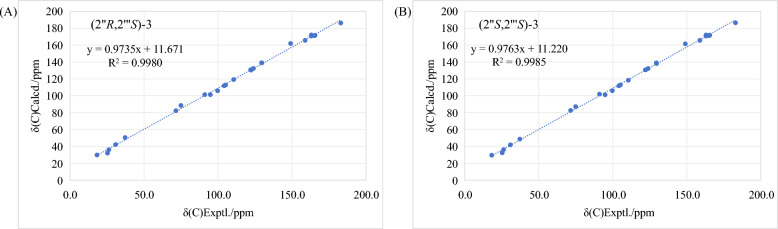
Regression analyses of experimental vs calculated ^13^C NMR chemical shifts of (2″*R*,2${\prime\prime\prime}$*S*)**-3** and (2″*S*,2${\prime\prime\prime}$*S*)**-3** at the B3LYP/6-311G(d,p) level; linear fitting was shown as a line

The molecular formula of **3a** was established as C_25_H_26_O_7_ based on its HRESIMS (*m/z* 461.1568 [M + Na]^+^, calcd. for 461.1571) and ^13^C NMR data, requiring thirteen degrees of unsaturation. Upon detailed analysis, it was found that the NMR data (Tables 1 and 2) of** 3a** resembled those of** 2a/2b**, with the presence of 2-hydroxy-3-methylbut-3-enyl and 2,3-dihydroxy-3-methylbutyl units still evident in** 3a**. This conclusion could be deduced by the ^1^H − ^1^H COSY spin coupling system of H_2_-1″/H-2″ and H_2_-1${\prime\prime\prime}$/H-2${\prime\prime\prime}$, along with the HMBC correlations from Me-4″ and Me-5″ to C-2″ and C-3″ and from Me-5${\prime\prime\prime}$ to C-2${\prime\prime\prime}$, C-3${\prime\prime\prime}$, and C-4${\prime\prime\prime}$. Subsequently, 3a exhibited one additional degree of unsaturation compared to 2a/2b, which means** 3a** formed an extra ring compared to** 2a/2b**. In addition, the molecular mass of 3a was lower by 18 amu than that of 2a/2b, indicating a loss of H_2_O, suggesting that 3a is a dehydration product of 2a/2b. By combining the diagnostic HMBC correlation from H-2″ to C-4′ and the chemical shifts of C-2″ (*δ*_C_ 91.0) and C-3″ (*δ*_C_ 71.5), a furan ring was constructed. The stereochemical assignment of** 3a** was investigated through comparative ^13^C NMR computational analysis employing DP4 + probability assessment at the B3LYP/6-311G(d,p) level of theory with acetone-*d*_6_ solvent modeling. Statistical analysis suggests the (2″*S*,2${\prime\prime\prime}$*S*)-3 configuration demonstrates an apparent high probability of 99.87% (Figures S1 and S2) and a relatively higher correlation coefficient value (*R*^2^) of 0.9985 (Fig. 5). Therefore, the (2″*S*,2${\prime\prime\prime}$*S*)-configuration currently represents the most computationally supported stereochemical interpretation. Subsequently, the ECD spectra of the (2″*R*,2${\prime\prime\prime}$*R*) and (2′′*S*,2′′′*S*) isomers of** 3** were calculated, and the absolute configuration of** 3a **was assigned as 2″*R*,2${\prime\prime\prime}$*R* based on the comparison between its experimental and calculated ECD spectra (Figure S3). Compound** 3b** was determined to be the enantiomer of 3a based on their identical ^1^H NMR spectra, mirror-image ECD curves (Fig. S3), and opposite-sign optical rotation values ([α]^20^_D_: − 12 for **3a **and + 11 for 3**b)**. Therefore, ** 3a**and** 3b** were named as (2″*R*,2${\prime\prime\prime}$*R*)-epimesatine L and (2″*S*,2${\prime\prime\prime}$*S*)-epimesatine L, respectively.

**Fig. 6:**
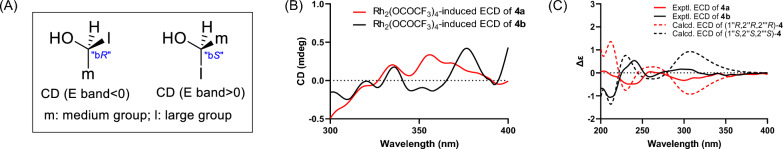
**A** Bulkiness rule for correlation of the alcohol geometry with the sign of the CD band E (around 350 nm) according to Gerards and Snatzke. **B** Rh_2_(OCOCF_3_)_4_-induced ECD spectra of compounds** 4a** and** 4b**. **C** Experimental and calculated ECD spectra of compounds** 4a** and** 4b**

Compounds **4a** and **4b** were identified as enantiomers similar to **1a**/**1b**–**3a**/**3b**. Their molecular formulas were determined as C_26_H_28_O_8_ through positive ion HRESIMS data analysis, indicating a mass 30 Da higher than** 3a**/**3b**, suggesting the existence of an additional methoxy group in** 4a** and** 4b**. Detailed NMR spectral analysis further confirmed that** 4a**/**4b** shared a similar planar structure with** 3a**/**3b**, except for the methoxy group at C-1″ in** 4a**/**4b**. The relative configuration of the two oxygenated methine protons on the furan ring of** 4a**/**4b **was determined to be *trans* by evaluating the coupling constant between H-1″ and H-2″ (*J* = 2.8 Hz) [29–31]. The configurations of C-2${\prime\prime\prime}$ in** 4a**/**4b** were determined through analysis of the ECD spectra of the Rh_2_(OCOCF_3_)_4_-complex, with the inherent contribution subtracted. According to the bulkiness rule [32], the positive and negative Cotton effects of the complex at around 350 nm correspond to the “b*S*” and “b*R*” configurations (Fig. 6A), respectively. Therefore, based on their respective positive and negative Cotton effects (Fig. 6B), the configurations of C-2${\prime\prime\prime}$ in** 4a** and** 4b** were assigned as *R* and *S*, respectively. Subsequently, the absolute configurations of** 4a** and** 4b** were determined through ECD calculations (Fig. 6C) and designated as (1″*R*,2″*R*,2${\prime\prime\prime}$*R*)-epimesatine M and (1″*S*,2″*S*,2${\prime\prime\prime}$*S*)-epimesatine M, respectively.

Compounds** 5a** and** 5b** were separated using chiral HPLC and were also confirmed to be enantiomers. Preliminary analysis of the 2D NMR data indicated that** 5a**/**5b** shared similar planar structures with** 4a**/**4b**, except for the different substituent at C-5′, which was identified as a prenyl group instead of a 2-hydroxy-3-methylbut-3-enyl group. Similarly, the relative configurations of H-1″/H-2″ in 5a/5b were determined to be *trans* based on their coupling constant (*J* = 2.7 Hz). Then, their absolute configurations were ascertained through ECD calculations, of which the experimental and calculated ECD curves displayed good agreement (Figure S4). Therefore, the structures of** 5a**/**5b** were verified and named as (1″*S*,2″*S*)-epimesatine N and (1″*R*,2″*R*)-epimesatine N, respectively.

Compounds** 6a** and** 6b** were also recognized as enantiomers and shared the same molecular formula as** 3a**/**3b **based on their HRESIMS data. Detailed analysis of the 1D and 2D NMR spectroscopic data revealed that** 6a** and** 6b** are structural analogs of** 3a**/**3b**, with the main difference being the presence of a pyran ring fused between C-3′ and C-4′ positions instead of a furan ring. This deduction was supported by the HMBC correlations from H_2_-1″ to C-2′ (*δ*_C_ 127.4), C-3′ (*δ*_C_ 121.5), and C-4′ (*δ*_C_ 155.6) and from Me-4″/Me-5″ to C-2″ (*δ*_C_ 69.3) and C-3″ (*δ*_C_ 79.2), along with the ^1^H − ^1^H COSY correlation of H_2_-1″ (*δ*_H_ 3.11, 1H, dd, *J* = 16.6, 5.1 Hz and 2.83, 1H, dd, *J* = 16.6, 7.4 Hz)/H-2″ (*δ*_H_ 3.87, 1H, dd, *J* = 7.4, 5.1 Hz). Consequently, the structures of **6a**/** 6b** were established based on the aforementioned evidence. Subsequently, the relative configurations of 6a/6b were evaluated through ^13^C NMR analysis using methodology consistent with that employed for** 3a**/**3b**. DP4 + probability evaluation suggested a strong preference for the (2″*S*,2${\prime\prime\prime}$*R*)-configuration with a statistical probability of 99.79% (Figures S5 and S6) and a higher *R*^2^ value (Figure S7). Moreover, ECD calculations (Figure S8) were utilized to determine the absolute configurations of** 6a**/** 6b**, leading to their designation as (2″*S*,2${\prime\prime\prime}$*R*)-epimesatine O and (2″*R*,2${\prime\prime\prime}$*S*)-epimesatine O, respectively.

Chiral compounds occurring in nature are typically found in an optically pure state, while, sometimes there are instances present in enantiomeric form within the same or different genera [33–35]. The chiral separation is usually conducted on mixtures with null ECD or optical rotation values, where these mixtures often exhibit similar ratios. However, in cases where enantiomers display varying ratios or are not separated as enantiomeric mixtures, there exists a potential risk of overlooking enantiomers of natural products. It has been verified that there are notable differences or even contradictory effects in the biological activity of two enantiomeric forms of certain compounds [35, 36]. This underscores the importance of meticulous analysis during the separation and identification of chiral compounds to ensure accurate determination of their structures.

**Fig. 7:**
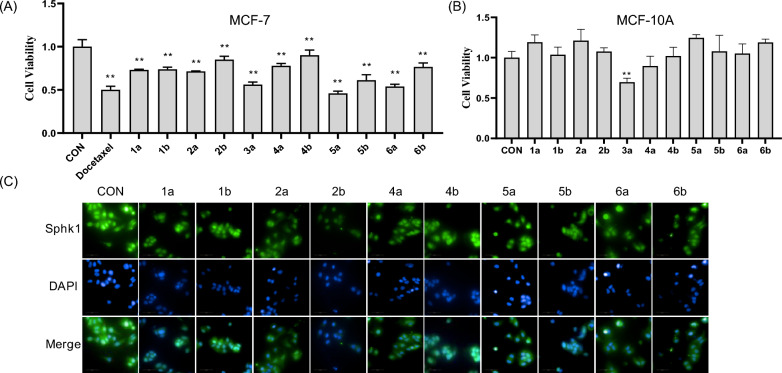
**A** Viability of MCF-7 cells after treated by isolated compounds at the concentration of 10 μM. **B** Viability of MCF-10A cells after treated by isolated compounds at the concentration of 10 μM. **C** Effects of** 1a/1b, 2a/2b**, and** 4a/4b–6a/6b** on the expression of Sphk1 in MCF-7 cells at the concentration of 10 μM. Docetaxel was used as positive control. ^**^P < 0.01 compared to CON group. Each bar and vertical line represents the mean ± SD of the values from three independent experiments

All compounds, with the exception of** 3b** due to its limited availability, were evaluated for their cytotoxicity. The results indicated that all tested compounds significantly inhibited the viability of MCF-7 cells (Fig. 7A) and did not exhibit cytotoxic effects on human breast epithelial cells MCF-10A, with the exception of** 3a** (Fig. 7B).

**Fig. 8:**
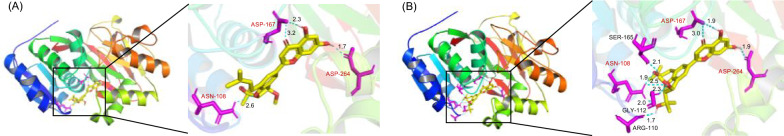
The molecular docking of Sphk1 for** 4a** (**A**) and** 5b** (**B**). Low-energy binding conformations of compounds** 4a** and bound to Sphk1 generated by virtual ligand docking. Compounds were exhibited as the ball-and-stick model showing carbon (yellow), hydrogen (grey) and oxygen (red). The key hydrogen bonding interactions with the enzymes were shown with the cyan line

Subsequently, the expression levels of Sphk1 in MCF-7 cells treated with these compounds were assessed through immunofluorescence. The results (Fig. 7C) indicated that all tested compounds effectively inhibited Sphk1 expression in MCF-7 cells, suggesting that Sphk1 may serve as a target for breast cancer treatment. Notably, compounds** 4a **and** 5b** demonstrated IC_50_ values of 7.45 and 8.97 μM, respectively, in MCF-7 cells (Table S1), highlighting their potential as therapeutic agents for breast cancer. Then, Sphk1 (PDBID 4v24) was subjected to molecular docking analyses with compounds** 4a** and** 5b**. As shown in Fig. 8, both** 4a** and** 5b** demonstrated hydrophilic interactions with the amino acid residues ASP-167, ASP-264, and ASN-108 of Sphk1. Especially, the hydrogen bond distances for both 4a and 5b were found to be less than 3.2 Å, indicating robust interactions between the compounds and Sphk1. These three amino acid residues are likely to serve as significant active sites for the treatment of breast cancer.

## Conclusions

In conclusion, six pairs of undescribed enantiomeric prenylated flavonoids, ( ±)-epimesatines J–O, were isolated and identified from the aerial parts of *Epimedium sagittatum* Maxim. Compounds** 1a/1b, 2a/2b**, and** 4a/4b–6a/6b** not only exhibited significant inhibitory effects on the viability of human breast cancer cells MCF-7 but also showed no cytotoxicity towards human breast epithelial cells MCF-10A. Therefore, these compounds show considerable promise as therapeutic agents against breast cancer. Furthermore, their pronounced ability to significantly reduce Sphk1 expression in MCF-7 cells highlights the potential of Sphk1 as a therapeutic target for breast cancer. Consequently, *E. sagittatum* is proposed as a valuable source for the development of breast cancer treatments.

## Experimental section

### General experimental procedures

Column chromatography (CC) was performed with silica gel (100–200 and 200–300 mesh, Marine Chemical Industry, Qingdao, China) and ODS (50 μm, YMC Group, Kyoto, Japan). Semipreparative high performance liquid chromatography (HPLC) separations were carried out on a Shimadzu LC-40 HPLC system, equipped with a DAD detector, using a reversed-phase (RP) C_18_ ODS column (10ID × 250 mm and 4.6ID × 250 mm, Cosmosil 5C_18_-MS-II Packed column, Nacalai Tesque, Japan). HRESIMS data were collected in the positive-ion mode on a Bruker Maxis HD mass spectrometer (Bruker, Germany). UV and IR spectra were obtained on an Evolution 300 instrument (Thermo Scientific, MA, USA) and a Nicolet IS 10 spectrophotometer (Thermo Scientific, MA, USA), respectively. Optical rotations and CD spectra were acquired by a Rudolph AP-IV polarimeter (Rudolph, Hackettstown, NJ, USA) and a Chirascan qCD spectrometer (Applied Photophysics Ltd, Surrey, U.K), respectively. The NMR spectra were measured on a Bruker Advance III 500 spectrometer (Bruker, Germany), and chemical shifts were referenced to the residual acetone-*d*_6_ (*δ*_H_ 2.05/*δ*_C_ 29.84) signals. Incubator and microplate reader used in activity experiments were carbon dioxide incubator 3111 and Multiskan MK3 microplate reader (Thermo Scientific, USA).

### Plant material

The aerial parts of *Epimedium sagittatum* Maxim. (Berberidaceae) were collected from Fenghui Epimedium herb GAP Base, Zhumadian, Henan Province, People’s Republic of China (GPS data: 114.496128, 33.018587), in September 2020. A voucher specimen (no. 20200960) was deposited in the Department of Pharmacy, Henan University of Chinese Medicine.

### Extraction and isolation

The air-dried aerial parts of *E. sagittatum* (80 kg) were extracted with 70% EtOH in three cycles, resulting in the production of an extract weighing 6.5 kg. Then, the extract was suspended with distilled water and successively partitioned into CH_2_Cl_2_ and EtOAc sequentially to obtain CH_2_Cl_2_, EtOAc, and aqueous fractions. Subsequently, the CH_2_Cl_2_ fraction (2.1 kg) was subjected to silica gel column chromatography (CC, 100–200 mesh) and eluted with a petroleum ether/ethyl acetate mixture (v:v, 50:1–0:1) to obtain eight fractions (Fr. A–H).

Fraction F (13.7 g) was subjected to silica gel CC (200–300 mesh) using a petroleum ether/EtOAc elution gradient (v:v, 35:1–0:1) to afford fifteen subfractions, Fr. F1–F15. Then, Fr. F15 (1.8 g) was further separated by ODS CC (MeOH:H_2_O = 40:60–100:0) and semipreparative HPLC to obtained a mixture of **5a** and **5b**. This mixture was then separated using a Chiralpak IC column (5 μm, 10 × 250 mm, Daicel Chiral Technologies Co., Ltd., Japan) with elution using MeOH:H_2_O (v:v, 80:20) to yield enantiomers of **5a** (1.3 mg, *t*_R_ = 39.3 min) and **5b** (1.2 mg, *t*_R_ = 33.3 min).

Fraction G (100.0 g) was separated through silica gel CC (100–200 mesh) eluting with petroleum ether/EtOAc (v:v, 50:1–0:1) to yield eighteen subfractions, Fr. G1–G18. Then, Fr. G17 (65.0 g) was separated by silica gel CC (200–300 mesh) to obtain nineteen fractions, Fr. G17.1–G17.19. Subsequently, Fr. G17.15 (38.9 g) was separated by ODS CC (MeOH:H_2_O = 40:60–100:0) to obtain twelve fractions, Fr. G17.15.1–G17.15.12. Further purification of Fr. G17.15.9 by semipreparative HPLC yielded six subfractions, Fr. G17.15.9.1–G17.15.9.6. Subsequent purification of subfraction Fr. G17.15.9.5 through HPLC (MeCN-H_2_O, 60:40, 1 mL/min) afforded compound 1b (9.9 mg, *t*_R_ 13.7 min), while compound** 1a** (5.6 mg, *t*_R_ 14.0 min) was isolated from subfraction Fr. G17.15.9.6 under identical chromatographic conditions. Fr. G17.15.10 was isolated by ODS CC (MeOH:H_2_O = 50:50–100:0) to obtain Fr. G17.15.10.1–G17.15.10.13, and then Fr. G17.15.10.6 was purified by HPLC to yield Fr. G17.15.10.6.1–G17.15.10.6.7. Compounds** 2a **(2.2 mg, *t*_R_ 42.2 min) and** 2b** (1.9 mg, *t*_R_ 48.9 min) were isolated from Fr. G17.15.10.6.1 via HPLC (MeCN–H_2_O, 40:60, 2 mL/min). Fr. G17.15.10.6.2 was subjected to HPLC separation to obtain Fr. G17.15.10.6.2.1–G17.15.10.6.2.6. Compounds** 4a** (3.6 mg, *t*_R_ 63.1 min) and** 4b** (2.1 mg, *t*_R_ 59.1 min) were purified from Fr. G17.15.10.6.2.2 and Fr. G17.15.10.6.2.3, respectively. Fr. G17.15.10.6.2.4 was further separated by HPLC (MeCN–H_2_O, 60:40, 2 mL/min) into five subfractions, Fr. G17.15.10.6.2.4.1–G17.15.10.6.2.4.5, with the third and fifth fractions identified as** 3a** (1.0 mg, *t*_R_ 58.8 min) and** 6b** (11.2 mg, *t*_R_ 63.6 min), respectively. Compounds** 3b** (1.4 mg, *t*_R_ 20.8 min) and** 6a** (9.0 mg, *t*_R_ 19.3 min) were purified from Fr. G17.15.10.6.2.4.4 (MeOH–H_2_O, 80:20, 2 mL/min).

**1a **and** 1b**: UV (MeOH) *λ*_max_ (log *ε*): 207 (4.24), 242 (3.89), 268 (3.82), 342 (3.96) nm; IR (*ν*_max_): 3400, 2976, 1653, 1616, 1477, 1439, 1359, 1166, 1031, 842 cm^–1^; ^1^H and ^13^C NMR data, see Tables 1 and 2.

*(R)-epimesatine J (****1a****)*: Yellow amorphous powder; [*α*]_D_^20^ − 4 (*c* 0.4, MeOH); HRESIMS *m/z* 463.1725 [M + Na]^+^ (calcd. for C_25_H_28_O_7_Na, 463.1727).

*(S)-epimesatine J (***1b**): Yellow amorphous powder; [*α*]_D_^20^ + 6 (*c* 0.4, MeOH); HRESIMS *m/z* 463.1713 [M + Na]^+^ (calcd. for C_25_H_28_O_7_Na, 463.1727).

**2a** and** 2b**: UV (MeOH) *λ*_max_ (log *ε*): 208 (4.32), 240 (3.98), 268 (3.93), 341 (4.01) nm; IR (*ν*_max_): 3444, 2975, 1699, 1653, 1436, 1363, 1260, 1170, 1052 cm^–1^; ^1^H and ^13^C NMR data, see Tables 1 and 2.

*(2″R,2*$\prime\prime\prime$*S)-epimesatine K* (**2a**): Yellow amorphous powder; [*α*]_D_^20^ − 3 (*c* 0.2, MeOH); HRESIMS *m/z* 479.1665 [M + Na]^+^ (calcd. for C_25_H_28_O_8_Na, 479.1676).

*(2″S,2*$\prime\prime\prime$*R)-epimesatine K* (**2b**): Yellow amorphous powder; [*α*]_D_^20^ + 3 (*c* 0.2, MeOH); HRESIMS *m/z* 479.1669 [M + Na]^+^ (calcd. for C_25_H_28_O_8_Na, 479.1676).

**3a** and** 3b**: UV (MeOH) *λ*_max_ (log *ε*): 204 (4.43), 238 (4.11), 269 (4.04), 344 (4.14) nm; IR (*ν*_max_): 3408, 2933, 1653, 1606, 1367, 1166, 1035, 843 cm^–1^; ^1^H and ^13^C NMR data, see Tables 1 and 2.

*(2″R,2*$\prime\prime\prime$*R)-epimesatine L* (**3a**): Yellow amorphous powder; [*α*]_D_^20^ − 12 (*c* 0.1, MeOH); HRESIMS *m/z* 461.1568 [M + Na]^+^ (calcd. for C_25_H_26_O_7_Na, 461.1571).

*(2″S,2*$\prime\prime\prime$S)-epimesatine L (**3b**): Yellow amorphous powder; [*α*]_D_^20^ + 11 (*c* 0.1, MeOH); HRESIMS *m/z* 461.1567 [M + Na]^+^ (calcd. for C_25_H_26_O_7_Na, 461.1571).

4a and 4b: UV (MeOH) *λ*_max_ (log *ε*): 202 (4.34), 269 (3.96), 336 (4.05) nm; IR (*ν*_max_): 3388, 2932, 2860, 1654, 1608, 1365, 1167, 1048, 843 cm^–1^; ^1^H and ^13^C NMR data, see Tables 1 and 2.

*(1″R,2″R,2*$\prime\prime\prime$*R)-epimesatine M *(**4a**): Yellow amorphous powder; [*α*]_D_^20^ − 3 (*c* 0.3, MeOH); HRESIMS *m/z* 491.1672 [M + Na]^+^ (calcd. for C_26_H_28_O_8_Na, 491.1676).

*(1″S,2″S,2*$\prime\prime\prime$*S)-epimesatine M* (**4b**): Yellow amorphous powder; [*α*]_D_^20^ + 4 (*c* 0.2, MeOH); HRESIMS *m/z* 491.1675 [M + Na]^+^ (calcd. for C_26_H_28_O_8_Na, 491.1676).

5a and 5b: UV (MeOH) *λ*_max_ (log *ε*): 203 (4.45), 268 (4.19), 335 (4.18) nm; IR (*ν*_max_): 3415, 1653, 1608, 1475, 1437, 1364, 1168, 1033, 981 cm^–1^; ^1^H and ^13^C NMR data, see Tables 1 and 2.

*(1″S,2″S)-epimesatine N *(**5a**): Yellow amorphous powder; [*α*]_D_^20^ − 7 (*c* 0.1, MeOH); HRESIMS *m/z* 475.1728 [M + Na]^+^ (calcd. for C_26_H_28_O_7_Na, 475.1727).

*(1″R,2″R)-epimesatine N *(**5b**): Yellow amorphous powder; [*α*]_D_^20^ + 10 (*c* 0.1, MeOH); HRESIMS *m/z* 475.1730 [M + Na]^+^ (calcd. for C_26_H_28_O_7_Na, 475.1727).

6a and 6b: UV (MeOH) *λ*_max_ (log *ε*): 202 (4.50), 244 (4.15), 268 (4.08), 341 (4.26) nm; IR (*ν*_max_): 3368, 2975, 1655, 1614, 1475, 1439, 1362, 1166, 1044, 841 cm^–1^; ^1^H and ^13^C NMR data, see Tables 1 and 2.

*(2″S,2*$\prime\prime\prime$*R)-epimesatine O* (**6a**): Yellow amorphous powder; [*α*]_D_^20^ − 11 (*c* 0.4, MeOH); HRESIMS *m/z* 461.1570 [M + Na]^+^ (calcd. for C_25_H_26_O_7_Na, 461.1571).

*(2″R,2*$\prime\prime\prime$*S)-epimesatine O* (**6b**): Yellow amorphous powder; [*α*]_D_^20^ + 16 (*c* 0.5, MeOH); HRESIMS *m/z* 461.1568 [M + Na]^+^ (calcd. for C_25_H_26_O_7_Na, 461.1571).

### Preparation of the Mo_2_(OAc)_4_ complex of compounds 1a, 1b, 2a, and 2b

Firstly, Mo_2_(OAc)_4_ (1.65 mg) was dissolved in DMSO (1 mL) at room temperature. Then, this solution was added to compound** 1a** (0.50 mg) and thoroughly mixed. The initial ECD spectrum was recorded immediately to establish a background absorption, followed by the recording of the complex-induced ECD spectra at ten minute intervals. The preparation of the Mo_2_(OAc)_4_-complex and ECD spectra of** 1b**,** 2a**, and** 2b** followed a similar procedure as that of** 1a**. The absolute configurations of these compounds were determined based on the Cotton effect observed in the complex-induced ECD spectra, in accordance with the helicity rule.

### ECD calculations

The conformations of** 2a/2b–6a/6b** were analyzed by GMMX software using the MMFF94 force field. The geometry optimizations and predictions of the ECD spectra of the conformers were conducted through density functional theory (DFT) at the B3LYP/6-31G(d) level using the Gaussian 16W software [37]. The ECD curves were simulated by SpecDis software (version 1.71) based on the Boltzmann distribution theory [38].

### Preparation of the Rh_2_(OCOCF_3_)_4_ complex of compounds 4a and 4b

Firstly, Rh_2_(OCOCF_3_)_4_ (1.20 mg) was dissolved in anhydrous CH_2_Cl_2_ (800 μL) at room temperature. Then, this solution was combined with compound **4a** (0.63 mg) and thoroughly mixed. The first ECD spectrum was immediately recorded as a baseline, after which complex-induced ECD spectra were recorded at ten minute intervals until reaching a stable state. The preparation of the Rh_2_(OCOCF_3_)_4_-complex and ECD spectra of** 4b** (0.55 mg) followed a similar procedure as that of** 4a**. The absolute configurations of** 4a** and** 4b** were determined by the Cotton effect observed in the E band around 350 nm in the complex-induced ECD spectra.

### Cytotoxicity assay

#### Cell culture

Take frozen MCF-7 and MCF-10A cells (Shanghai Cell Bank, China) and melt them in a 37 °C water bath until the state of ice and water coexisted. Then, the mixture was centrifuged immediately at 1000 rpm for 5 min. Subsequently, discard the supernatant and transfer the cells to a DMEM medium containing 10% FBS (100 kU/L for both penicillin and streptomycin). Cultivate in a 37 °C constant temperature incubator containing 5% CO_2_ until the cells grow to 80% ~ 90% of the dish for passage. Replace fresh culture medium every 24 h.

#### Cell viability of isolated compounds on MCF-7 and MCF-10A cells using MTT method

The MCF-7 and MCF-10A cells were cultured until the logarithmic growth phase in an incubator containing 5% CO_2_ at 37 °C. Then, the cells were inoculated into a 96-well plate (E190236X, PerkinElmer, United States) at a density of 2 × 10^4^ cells/mL and 200 μL per well. After 24 h of incubation, these wells were divided into normal control (CON) group and sample (10 μM) groups to culture for another 24 h. Subsequently, 20 μL of MTT (Solarbio life sciences, Beijing, China) solution (5 mg/mL) were added to each well and continue to culture for 4 h. Then, the culture medium was aspirated carefully and 150 μL of DMSO were added to each well to dissolve the blue-violet crystals. The OD values of each well were measured by a microplate reader at 490 nm, and the cell viability was calculated.

#### Cellular immunofluorescence

The assay for cellular immunofluorescence was performed in 96-well plates, with MCF-7 cells seeded at a density of 2 × 10^4^ cells/mL. After 24 h, the cells were divided into control (CON) and various sample groups (10 μM) and cultured for an additional 24 h. The cells were then fixed with 4% paraformaldehyde for 15 min and permeabilized with 0.25% Triton X-100 for 10 min. Subsequently, 1% BSA was added for blocking for 30 min, after which the primary antibody Sphk1 (ab262697, Abcam) was introduced and incubated overnight at 4 °C. The cells were subsequently washed three times with PBST, counterstained with DAPI for 4 min, washed once with PBS, and then imaged using the OperettaCLS high-content imaging analysis system (Opera Phenix, PerkinElmer, United States).

#### Real time cellular analysis (RTCA)

Baseline measurements were conducted using culture medium. MCF-7 cells in logarithmic growth phase were seeded into E-plate plates at a density of 2 × 10^4^ cells/mL per well. After 24 h of incubation, these wells were divided into control (CON) group and sample (1 μM, 5 μM, 10 μM, 20 μM, 50 μM, and 100 μM) groups, and their growth curves were continuously measured. After completion, the Sigmoidal dose–response (Vanable slope) algorithm was employed to determine the target time for calculating the IC_50_ values of the analyzed compounds. Docetaxel (Shanghai yuanye Bio-Technology Co., Ltd, Shanghai, China) was used as positive control.

#### Statistical analysis

The experimental data were expressed as mean ± standard deviation (SD) and analyzed by SPSS 26.0 software. One-way analysis of variance was used for comparison between groups. ^*^P < 0.05 indicates a significant difference, while ^**^P < 0.01 indicates an extremely significant difference.

### Molecular docking

Molecular docking simulations were performed using the software AutoDock Tools (1.5.7). The crystal structures of Sphk1 was obtained from the RCSB Protein Data Bank (http://www.pdb.org/) and embellished through PyMOL (3.0.3) and the AutoDock Tools softwares.

## Supplementary Information

Supplementary material 1.

## Data Availability

The data that support the findings of this study are openly available in the Science Data Bank at.
