# Supplementary material for: Six pairs of enantiomeric prenylated flavonoids with cytotoxic activities from Epimedium sagittatum Maxim
Source: Nat Prod Bioprospect. 2025 May 13;15(1):31. doi: 10.1007/s13659-025-00510-1 (PMC12075077; doi:10.1007/s13659-025-00510-1)
Supplement: Supplementary file 1 — Supplementary material 1. [file 13659_2025_510_MOESM1_ESM.pdf]

Supplementary Information

Six pairs of enantiomeric flavonoids with cytotoxic activities from *Epimedium sagittatum* Maxim

Shuang-Shuang Xie[1,2], Xiang Yu[1,2], Qi-Mei Tie[1,2], Jing-Ke Zhang[1,2], Bei-Bei Zhang[1,2], Meng-Nan Zeng[1,2], Xiao-Ke Zheng[1,2], Wei-Sheng Feng[1,2]*

[1] *School of Pharmacy, Henan University of Chinese Medicine, Zhengzhou, 450046, PR China*

[2] *The Engineering and Technology Center for Chinese Medicine Development of Henan Province, Zhengzhou, 450046, PR China*

* Corresponding author.

E-*mail address:* fwsh@hactcm.edu.cn (Weisheng Feng)

List of Supplementary Information

Figure S1. Chemical structures of the calculated configurations of compound **3**............................6

Figure S2. The data of DP4+ method of compound **3**...6

Figure S3. Experimental and calculated ECD spectra of compounds **3a** and **3b**.............................7

Figure S4. Experimental and calculated ECD spectra of compounds **5a** and **5b**.............................7

Figure S5. Chemical structures of the calculated configurations of compound **6**............................7

Figure S6. The data of DP4+ method of compound **6**...8

Figure S7. Regression analyses of experimental vs calculated ^{13}C NMR chemical shifts of (2''*R*,2'''*R*)-**6** and (2''*S*,2'''*R*)-**6** at the B3LYP/6-311G(d,p) level; linear fitting was shown as a line...8

Figure S8. Experimental and calculated ECD spectra of compounds **6a** and **6b**.............................9

Table S1. The inhibitory effects of isolated compounds on MCF-7 cells..9

1D and 2D-NMR, IR, UV, HRESIMS spectra for compounds 1−6

Figure S9. ^{1}H NMR (500 MHz) spectrum of **1a/1b** in Acetone-d_6..10

Figure S10. DEPT and ^{13}C NMR (125 MHz) spectra of **1a** in Acetone-d_6....................................10

Figure S11. HSQC NMR spectrum of **1a** in Acetone-d_6...11

Figure S12. HMBC NMR spectrum of **1a** in Acetone-d_6...11

Figure S13. ^{1}H-^{1}H COSY NMR spectrum of **1a** in Acetone-d_6..12

Figure S14. NOESY NMR spectrum of **1a** in Acetone-d_6...12

Figure S15. IR spectrum of **1a**...13

Figure S16. UV spectrum of **1a**..13

Figure S17. HRESIMS spectrum of **1a**...14

Figure S18. HRESIMS spectrum of **1b**...14

Figure S19. ^{1}H NMR (500 MHz) spectrum of **2a/2b** in Acetone-d_6..15

Figure S20. DEPT and ^{13}C NMR (125 MHz) spectra of **2a** in Acetone-d_6..................................15

Figure S21. HSQC NMR spectrum of **2a** in Acetone-d_6...16

Figure S22. HMBC NMR spectrum of **2a** in Acetone-d_6...16

Figure S23. ^{1}H-^{1}H COSY NMR spectrum of **2a** in Acetone-d_6..17

Figure S24. NOESY NMR spectrum of **2a** in Acetone-d_6...17

Figure S25. IR spectrum of **2a**...18

Figure S26. UV spectrum of **2a**..18

Figure S27. HRESIMS spectrum of **2a**..19

Figure S28. HRESIMS spectrum of **2b**..19

Figure S29. ^{1}H NMR (500 MHz) spectrum of **3a/3b** in Acetone-d_6...20

Figure S30. DEPT and ^{13}C NMR (125 MHz) spectra of **3a** in Acetone-d_6...................................20

Figure S31. HSQC NMR spectrum of **3a** in Acetone-d_6...;..................21

Figure S32. HMBC NMR spectrum of **3a** in Acetone-d_6...21

Figure S33. ^{1}H-^{1}H COSY NMR spectrum of **3a** in Acetone-d_6..22

Figure S34. NOESY NMR spectrum of **3a** in Acetone-d_6...22

Figure S35. IR spectrum of **3a**...23

Figure S36. UV spectrum of **3a**..23

Figure S37. HRESIMS spectrum of **3a**..24

Figure S38. HRESIMS spectrum of **3b**..24

Figure S39. ^{1}H NMR (500 MHz) spectrum of **4a/4b** in Acetone-d_6...25

Figure S40. DEPT and ^{13}C NMR (125 MHz) spectra of **4a** in Acetone-d_6...................................25

Figure S41. HSQC NMR spectrum of **4a** in Acetone-d_6...;..................26

Figure S42. HMBC NMR spectrum of **4a** in Acetone-d_6..26

Figure S43. ^{1}H-^{1}H COSY NMR spectrum of **4a** in Acetone-d_6..27

Figure S44. NOESY NMR spectrum of **4a** in Acetone-d_6...27

Figure S45. IR spectrum of **4a**...28

Figure S46. UV spectrum of **4a**..28

Figure S47. HRESIMS spectrum of **4a**..29

Figure S48. HRESIMS spectrum of **4b**..29

Figure S49. ^{1}H NMR (500 MHz) spectrum of **5a/5b** in Acetone-d_6...30

Figure S50. DEPT and ^{13}C NMR (125 MHz) spectra of **5a** in Acetone-d_6...................................30

Figure S51. HSQC NMR spectrum of **5a** in Acetone-d_6..31

Figure S52. HMBC NMR spectrum of **5a** in Acetone-d_6..31

Figure S53. ^{1}H-^{1}H COSY NMR spectrum of **5a** in Acetone-d_6..32

Figure S54. NOESY NMR spectrum of **5a** in Acetone-d_6...32

Figure S55. IR spectrum of **5a**...33

Figure S56. UV spectrum of **5a**......33

Figure S57. HRESIMS spectrum of **5a**......35

Figure S58. HRESIMS spectrum of **5b**......35

Figure S59. ^{1}H NMR (500 MHz) spectrum of **6a/6b** in Acetone-d_6......36

Figure S60. DEPT and ^{13}C NMR (125 MHz) spectra of **6a** in Acetone-d_6......36

Figure S61. HSQC NMR spectrum of **6a** in Acetone-d_6......;......37

Figure S62. HMBC NMR spectrum of **6a** in Acetone-d_6......37

Figure S63. ^{1}H-^{1}H COSY NMR spectrum of **6a** in Acetone-d_6......38

Figure S64. NOESY NMR spectrum of **6a** in Acetone-d_6......38

Figure S65. IR spectrum of **6a**......39

Figure S66. UV spectrum of **6a**......39

Figure S67. HRESIMS spectrum of **6a**......40

Figure S68. HRESIMS spectrum of **6b**......40

Figure S69. HPLC separation of **1a**......41

Figure S70. HPLC separation of **1b**......41

Figure S71. HPLC separation of **2a** and **2b**......41

Figure S72. HPLC separation of **3a** and **6b**......42

Figure S73. HPLC separation of **3b** and **6a**......42

Figure S74. HPLC separation of **4a** and **4b**......42

Figure S75. HPLC separation of **5a** and **5b**......43

ECD calculation parts

Table S2. Conformational analysis of (2″*S*,2‴*S*)-**2**......43

Table S3. Coordinates of (2″*S*,2‴*S*)-**2**......43

Table S4. Conformational analysis of (2″*S*,2‴*R*)-**2**......52

Table S5 Coordinates of (2″*S*,2‴*R*)-**2**......52

Table S6. Conformational analysis of (2″*S*,2‴*S*)-**3**......65

Table S7. Coordinates of (2″*S*,2‴*S*)-**3**......65

Table S8. Conformational analysis of (1''*S*,2''*S*,2'''*S*)-**4**......77

Table S9. Coordinates of (1''*S*,2''*S*,2'''*S*)-**4**......78

Table S10. Conformational analysis of (1''*S*,2''*S*,2'''*R*)-**4**......87

Table S11. Coordinates of (1"*S*,2"*S*,2'''*R*)-**4**......88

Table S12. Conformational analysis of (1"*S*,2"*S*)-**5**......98

Table S13. Coordinates of (1"*S*,2"*S*)-**5**......99

Table S14. Conformational analysis of (2"*S*,2'''*R*)-**6**......116

Table S15. Coordinates of (2"*S*,2'''*R*)-**6**......117

^{13}C NMR calculation parts

Table S16. Energies and populations of conformers of the calculated configuration of **3** at B3LYP/6-311G(d,p) theory level......130

Table S17. Energies and populations of conformers of the calculated configuration of **6** at B3LYP/6-311G(d,p) theory level......131

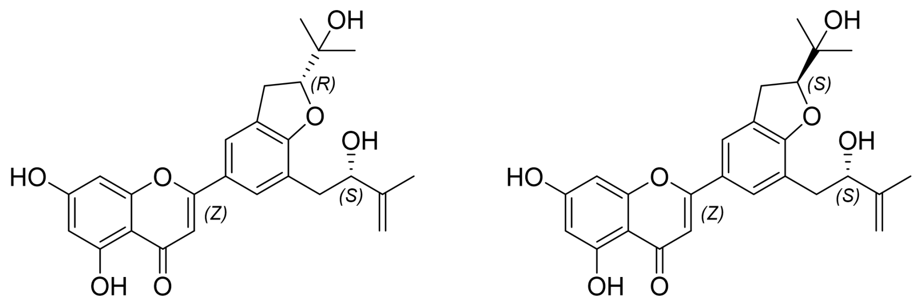

(2"*R*,2'''*S*)-**3** (2"*S*,2'''*S*)-**3**

Figure S1. Chemical structures of the calculated configurations of compound **3**

Functional	Solvent?	Basis Set	Type of Data
B3LYP	PCM	6-311G(d,p)	Unscaled Shifts

Nuclei	sp2?	xperimenta	Isomer 1	Isomer 2	Isomer 3	Isomer 4	Isomer 5
		DP4+	0.13%	99.87%	-	-	-
C	x	165.5	171.552173	171.82898			
C	x	104.1	111.88367	111.765028			
C	x	182.9	186.187536	186.457271			
C	x	163	172.06831	172.085147			
C	x	99.7	105.903983	106.001097			
C	x	165.1	171.204533	171.222905			
C	x	94.8	101.297877	101.176706			
C	x	158.8	165.648602	165.598024			
C	x	105.1	112.764629	112.797596			
C	x	123.9	132.344326	132.298691			
C	x	122	130.880601	130.746178			
C	x	129.4	139.078281	139.01064			
C	x	162.9	170.744948	170.749458			
C	x	122.3	130.379689	130.842109			
C	x	129.6	138.93685	138.176176			
C		30.8	42.2069461	41.8588999			
C		91	101.36024	101.953811			
C		71.5	82.2686934	82.617476			
C		26.2	36.1614948	36.2538931			
C		25.3	32.0814571	32.3915288			
C		37.2	50.4846186	48.6639083			
C		74.9	88.5496435	87.1419191			
C	x	149	161.966169	161.531094			
C	x	110.5	119.163752	118.384818			
C		18.2	29.8513387	29.6022152			

Figure S2. The data of DP4+ method of compound **3**

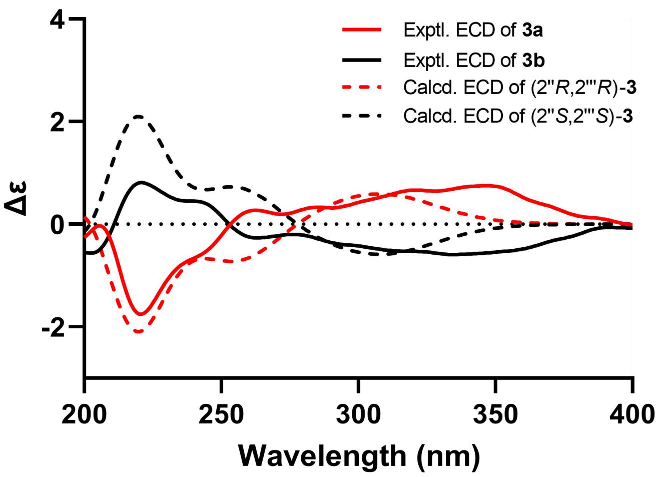

Figure S3. Experimental and calculated ECD spectra of compounds **3a** and **3b**

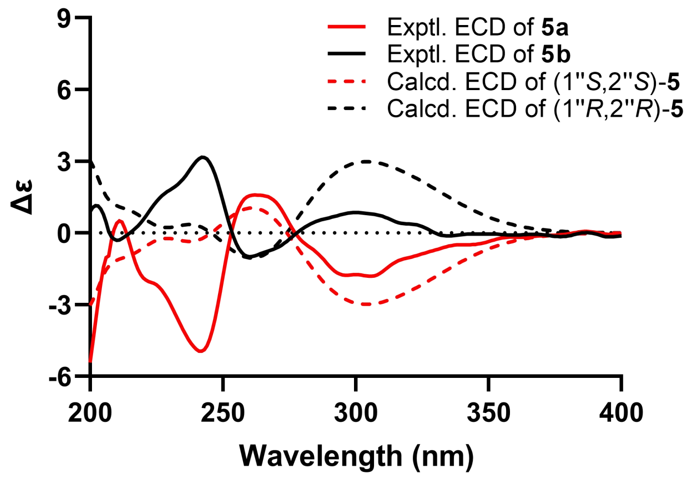

Figure S4. Experimental and calculated ECD spectra of compounds **5a** and **5b**

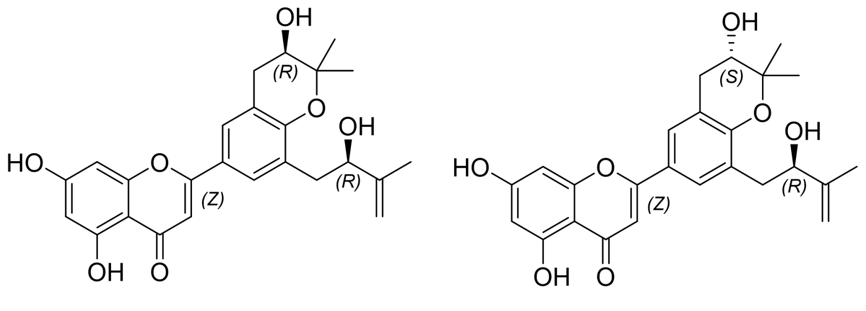

(2''*R*,2'''*R*)-**6** (2''*S*,2'''*R*)-**6**

Figure S5. Chemical structures of the calculated configurations of compound **6**

Functional		Solvent?		Basis Set		Type of Data	
B3LYP		PCM		6-311G(d,p)		Unscaled Shifts	
		DP4+	0.21%	99.79%	-	-	-
Nuclei	sp2?	xperimenta	Isomer 1	Isomer 2	Isomer 3	Isomer 4	Isomer 5
C	x	165.3	170.8083625	170.9197843			
C	x	104.2	108.2823695	108.3931804			
C	x	182.9	186.5937735	186.6190188			
C	x	163	171.4782683	171.4699937			
C	x	99.6	102.072658	102.0231864			
C	x	164.9	170.6268556	170.6310538			
C	x	94.8	97.01780091	97.11317047			
C	x	158.8	164.6434074	164.6871866			
C	x	105.2	109.2193429	109.2129567			
C	x	122.9	129.017686	129.0384437			
C	x	127.4	134.3544992	134.435958			
C	x	121.5	126.7174829	126.4906031			
C	x	155.6	163.4163259	163.3695456			
C	x	129	135.9606617	135.9031414			
C	x	128.4	134.4928339	134.4143577			
C		32.2	36.00297861	35.98162005			
C		69.3	73.90579752	73.90676544			
C		79.2	86.9282097	86.95529476			
C		26.1	25.11857926	26.32211952			
C		21.4	26.18255344	25.32955746			
C		37.9	42.77229927	41.6002136			
C		75.1	82.87689088	82.27072543			
C	x	149.3	162.6958078	160.1806576			
C	x	110.2	114.0960631	113.5848008			
C		18.3	21.35244189	22.60104198			

Figure S6. The data of DP4+ method of compound **6**

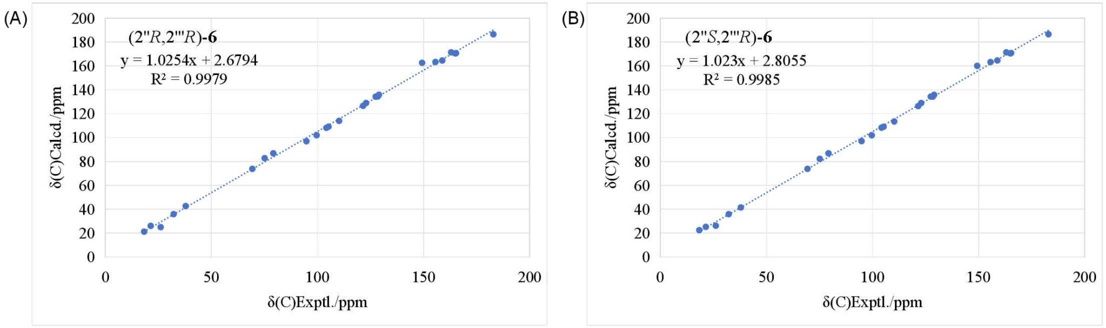

Figure S7. Regression analyses of experimental vs calculated ^{13}C NMR chemical shifts of (2''*R*,2'''*R*)-**6** and (2''*S*,2'''*R*)-**6** at the B3LYP/6-311G(d,p) level; linear fitting was shown as a line

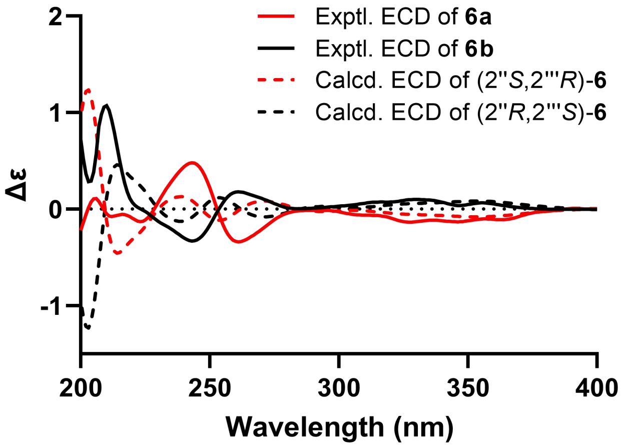

Figure S8. Experimental and calculated ECD spectra of compounds **6a** and **6b**

Table S1 The inhibitory effects of isolated compounds on MCF-7 cells

Compounds	IC_{50} (μM)[a]	Compounds	IC_{50} (μM)[a]
**1a**	54.9	**5a**	86.3
**1b**	51.5	**5b**	8.97
**2a**	>100	**6a**	63.8
**2b**	68.6	**6b**	>100
**4a**	7.45	Docetaxel[b]	2.13
**4b**	95.7		

[a] Data represent the means from triplicate experiments

[b] Docetaxel was used as a positive control

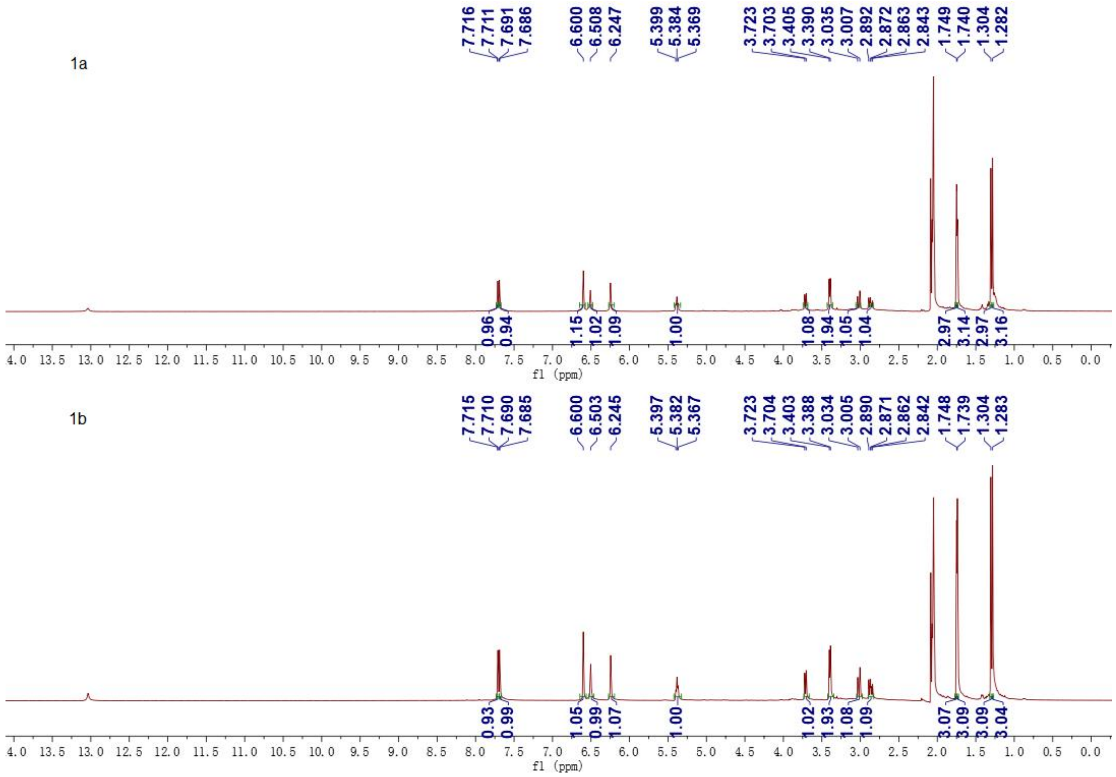

Figure S9. ^{1}H NMR (500 MHz) spectrum of **1a/1b** in Acetone-d_6.

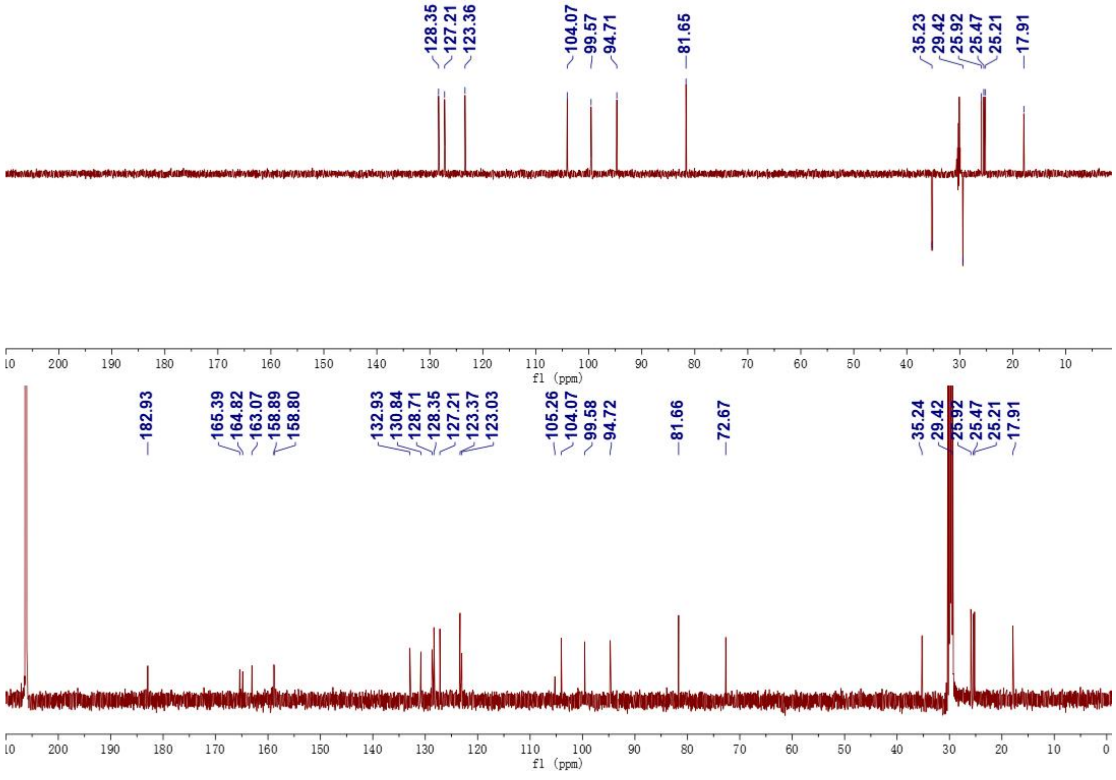

Figure S10. DEPT and ^{13}C NMR (125 MHz) spectra of **1a** in Acetone-d_6.

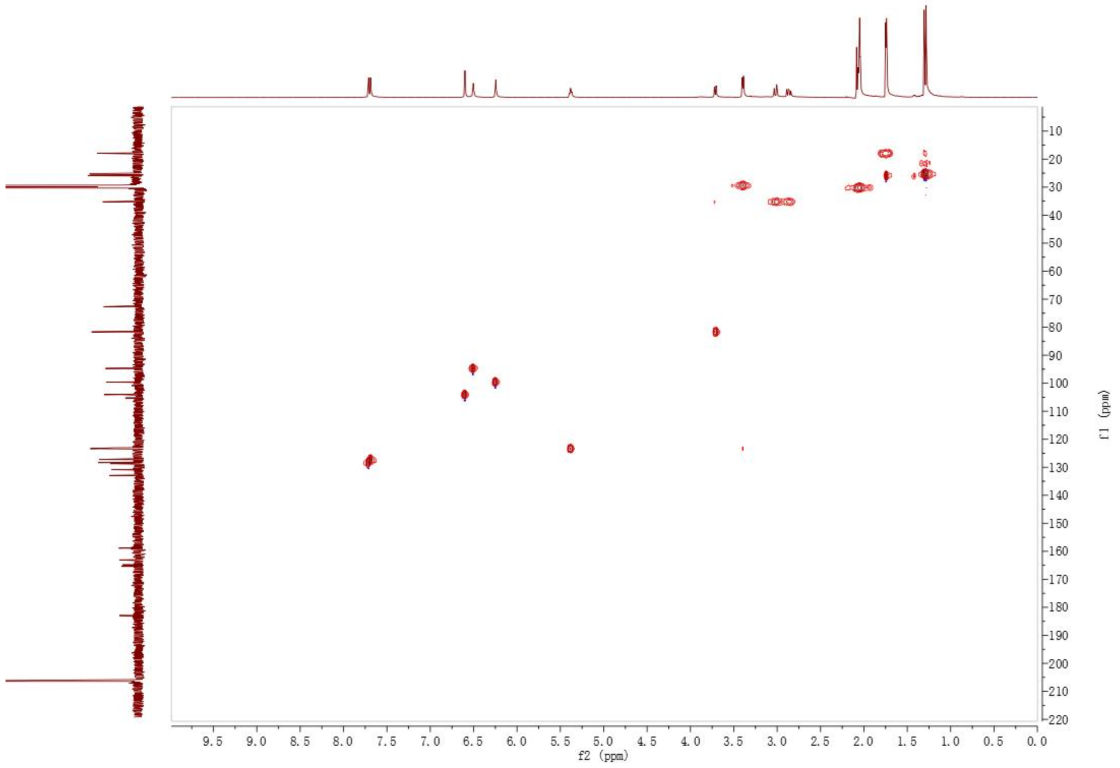

Figure S11. HSQC NMR spectrum of **1a** in Acetone-*d*6.

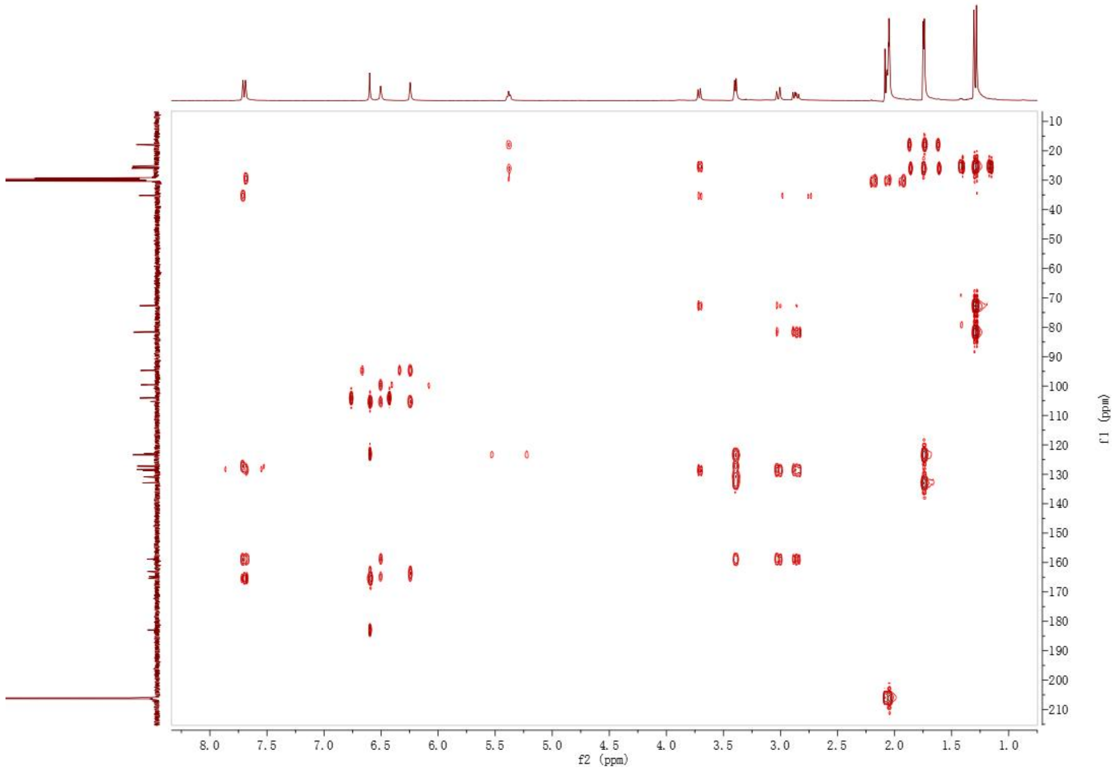

Figure S12. HMBC NMR spectrum of **1a** in Acetone-*d*6.

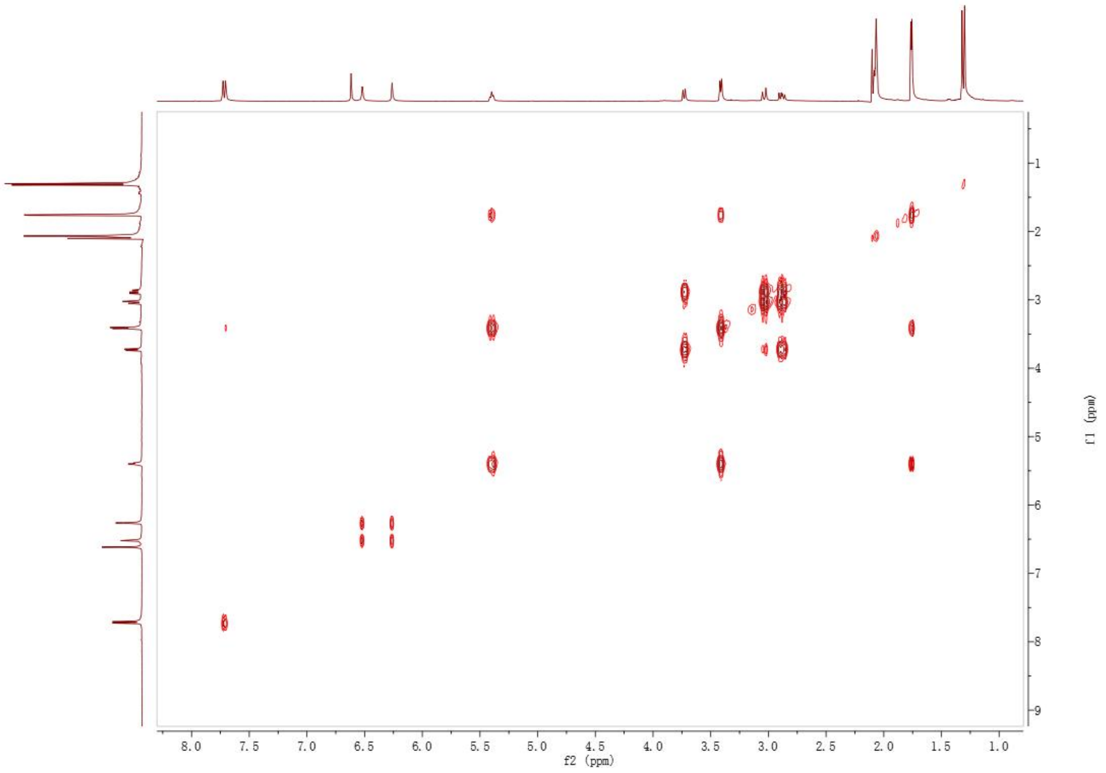

Figure S13. ^{1}H-^{1}H COSY NMR spectrum of **1a** in Acetone-d_6.

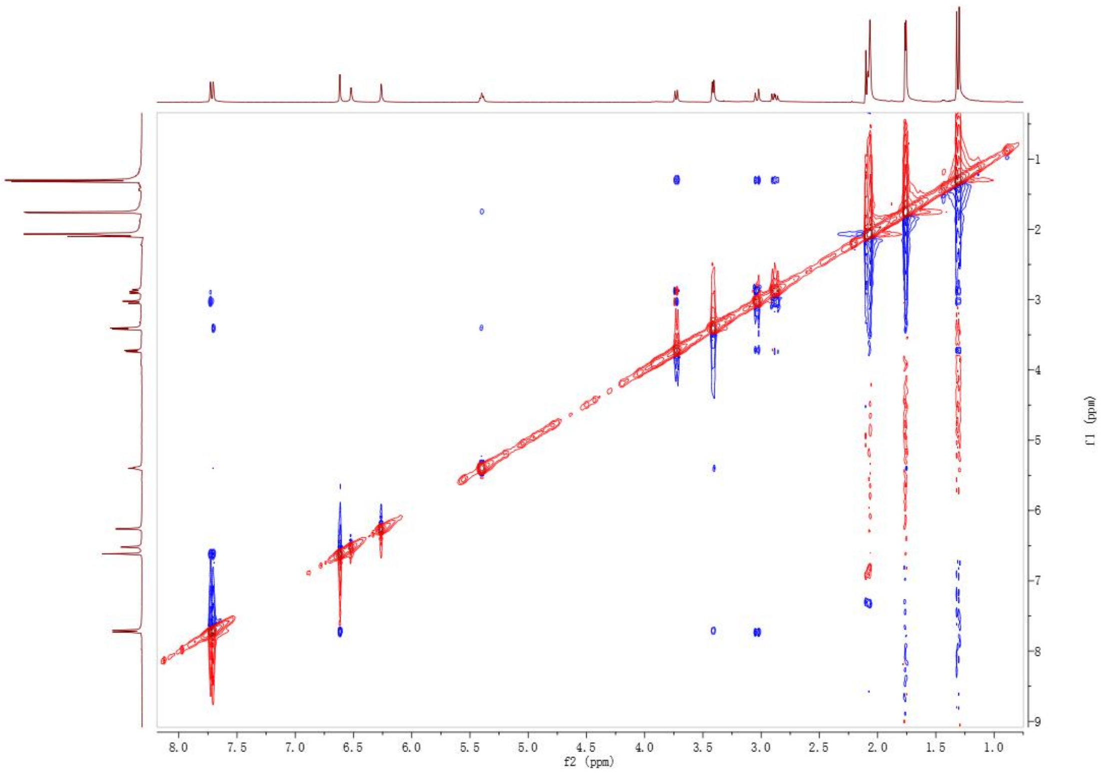

Figure S14. NOESY NMR spectrum of **1a** in Acetone-d_6.

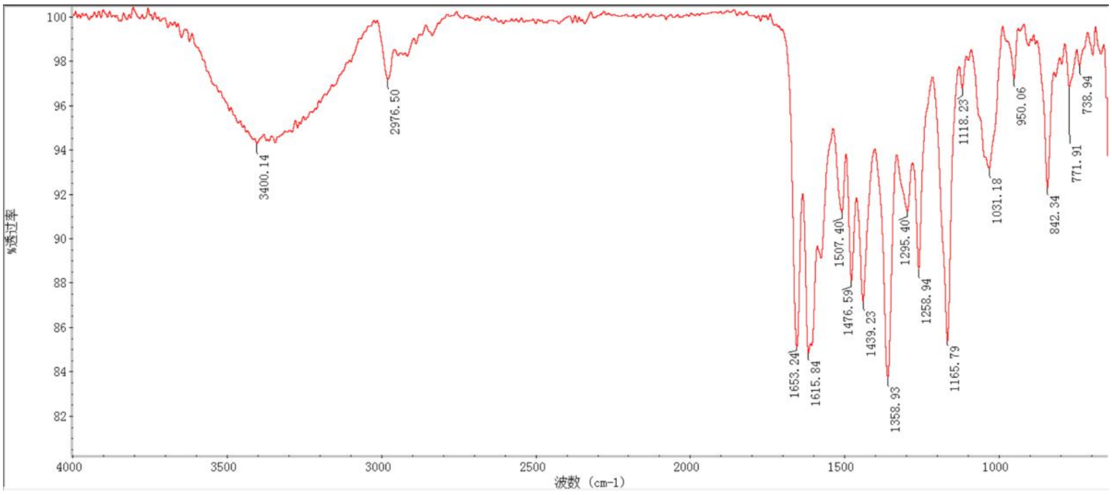

Figure S15. IR spectrum of **1a**.

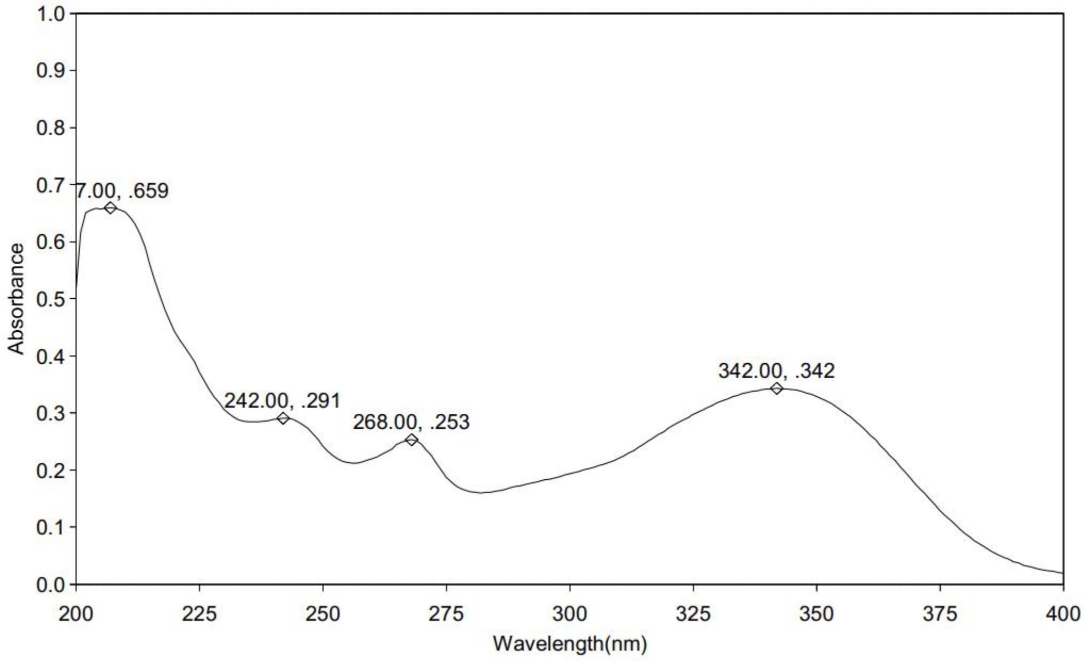

Figure S16. UV spectrum of **1a**.

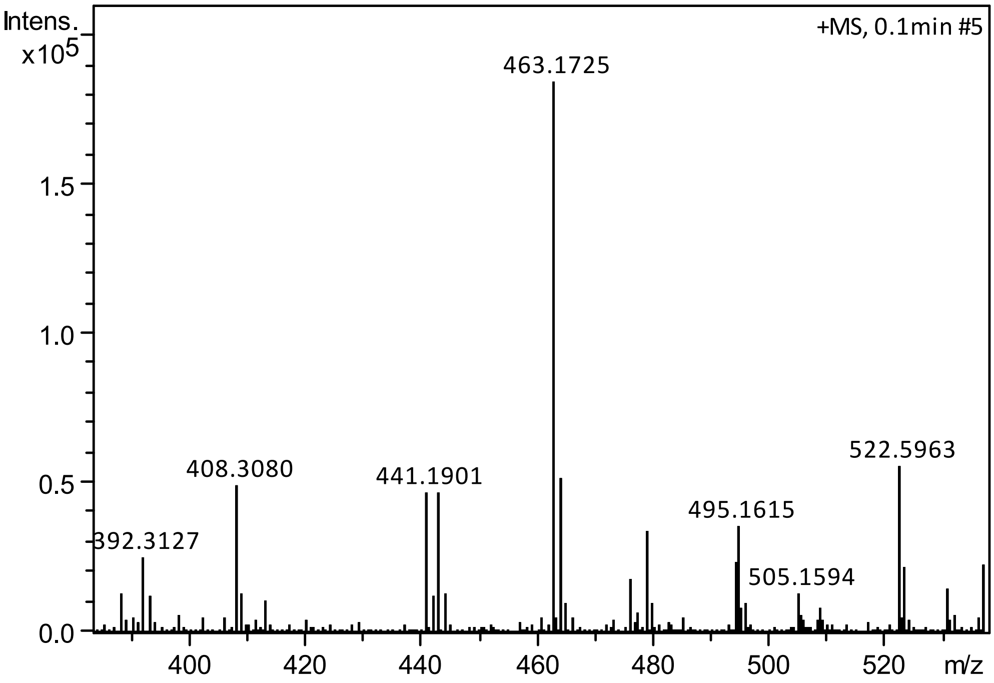

Figure S17. HRESIMS spectrum of **1a**.

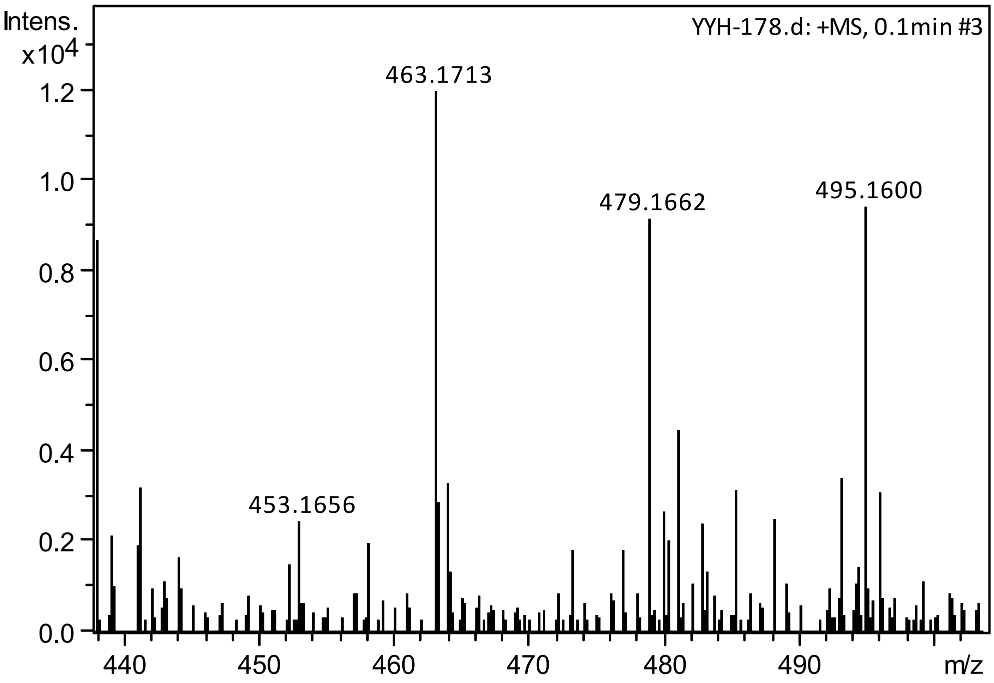

Figure S18. HRESIMS spectrum of **1b**.

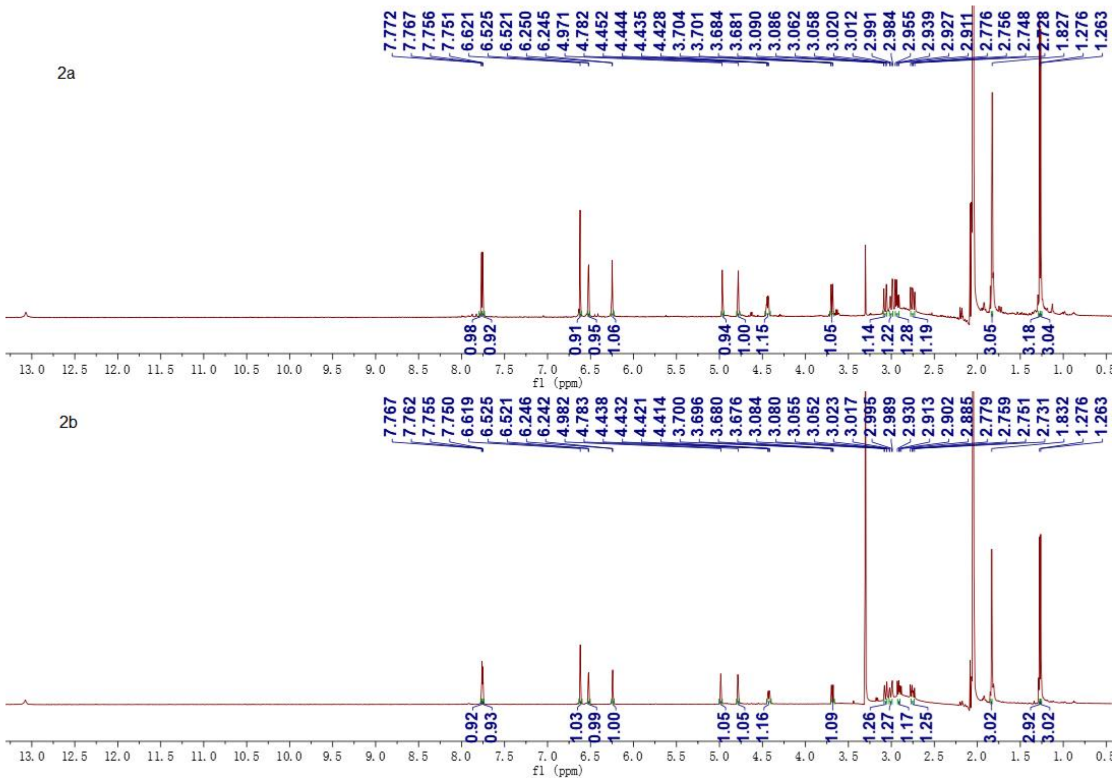

Figure S19. ^{1}H NMR (500 MHz) spectrum of **2a/2b** in Acetone-d_6.

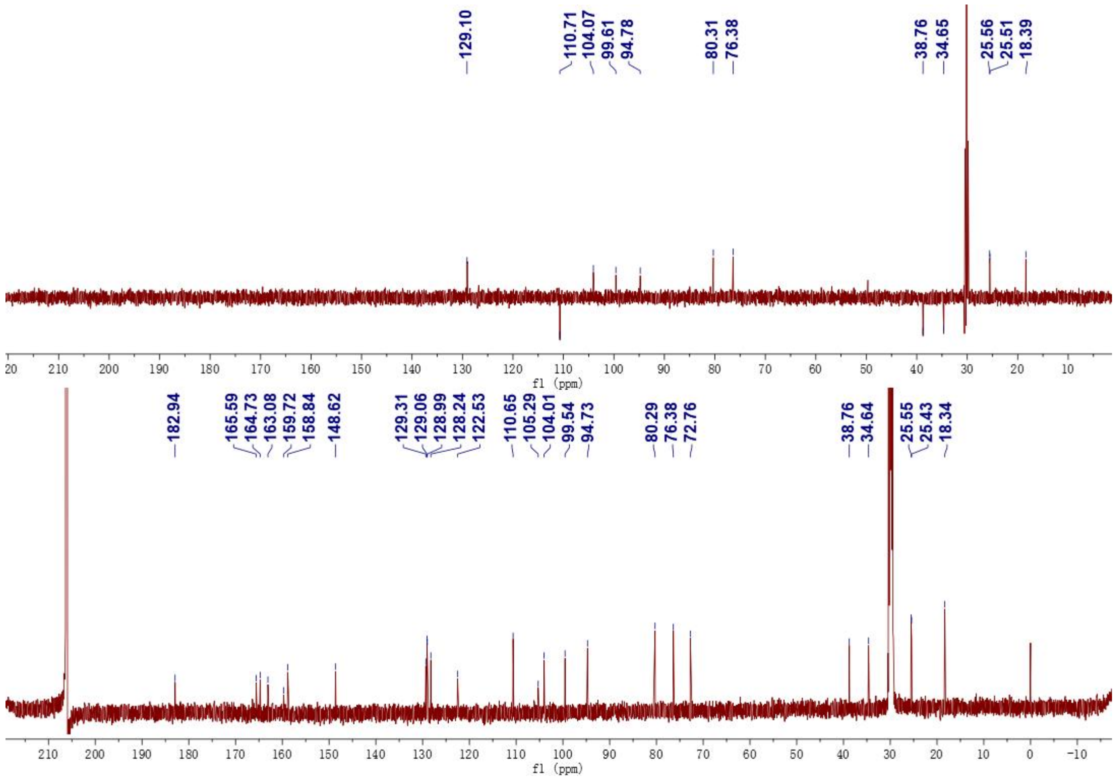

Figure S20. DEPT and ^{13}C NMR (125 MHz) spectra of **2a** in Acetone-d_6.

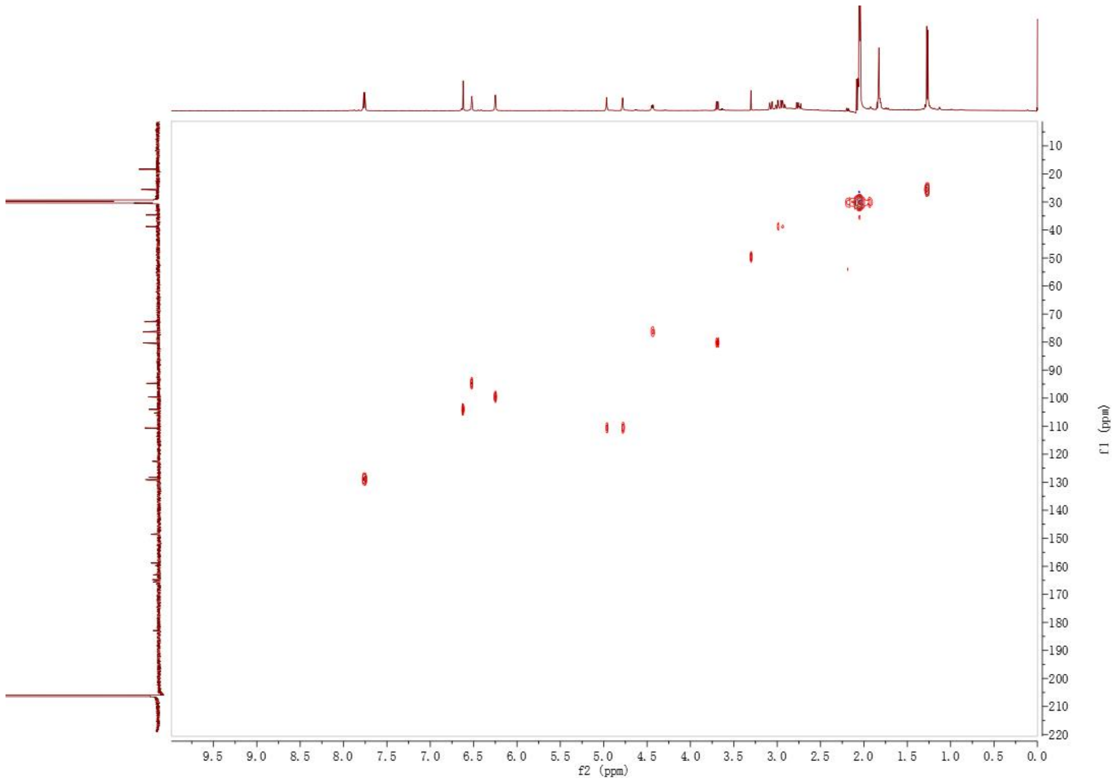

Figure S21. HSQC NMR spectrum of **2a** in Acetone-d_6.

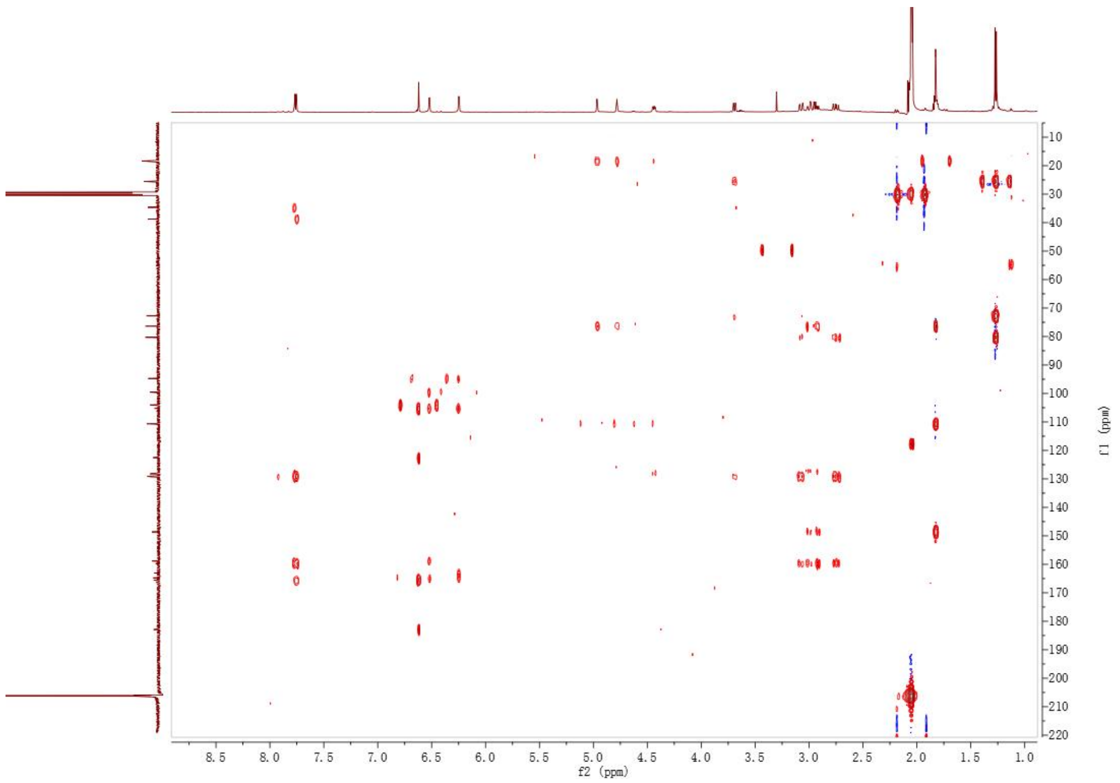

Figure S22. HMBC NMR spectrum of **2a** in Acetone-d_6.

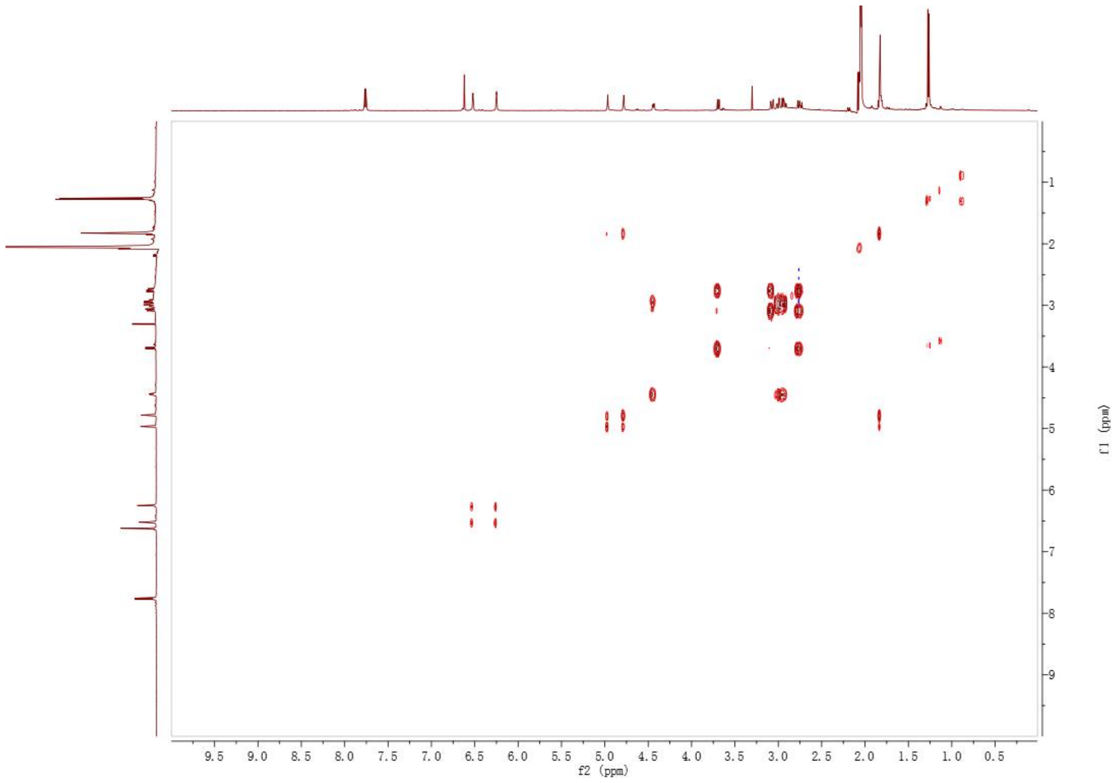

Figure S23. ^{1}H-^{1}H COSY NMR spectrum of **2a** in Acetone-d_6.

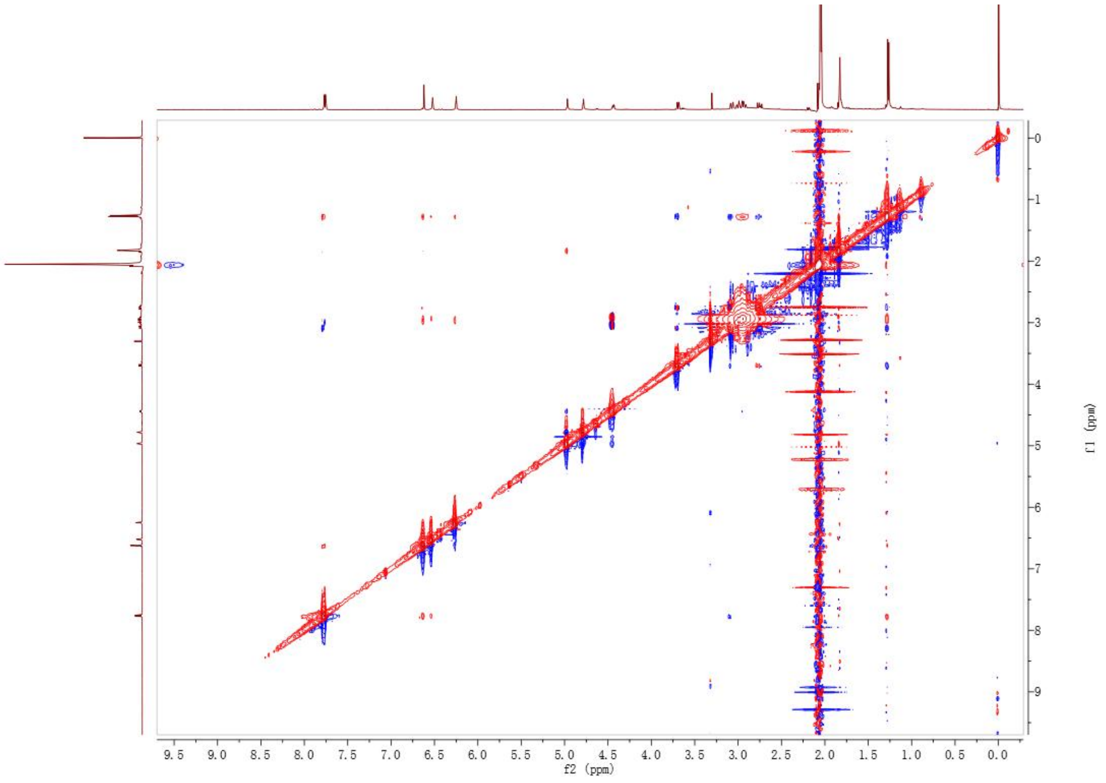

Figure S24. NOESY NMR spectrum of **2a** in Acetone-d_6.

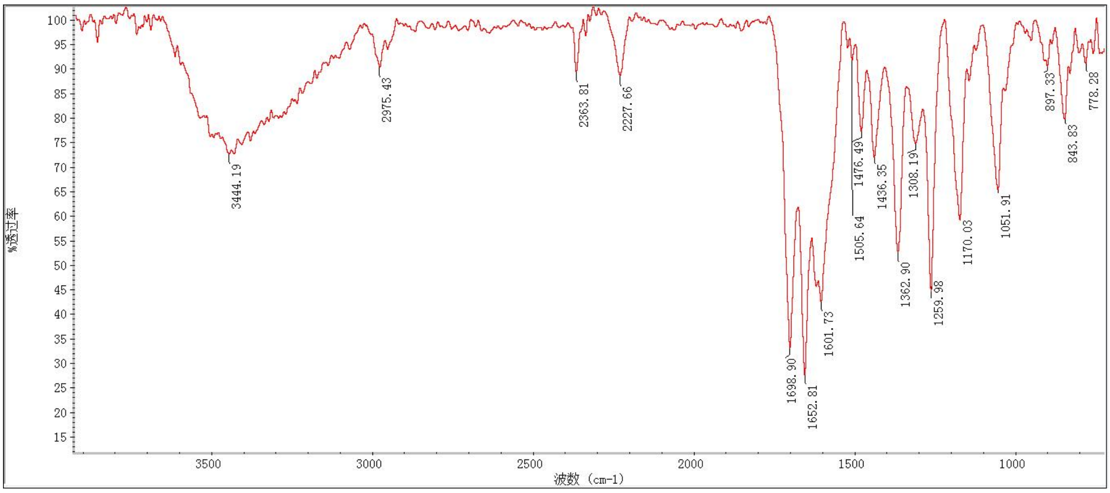

Figure S25. IR spectrum of **2a**.

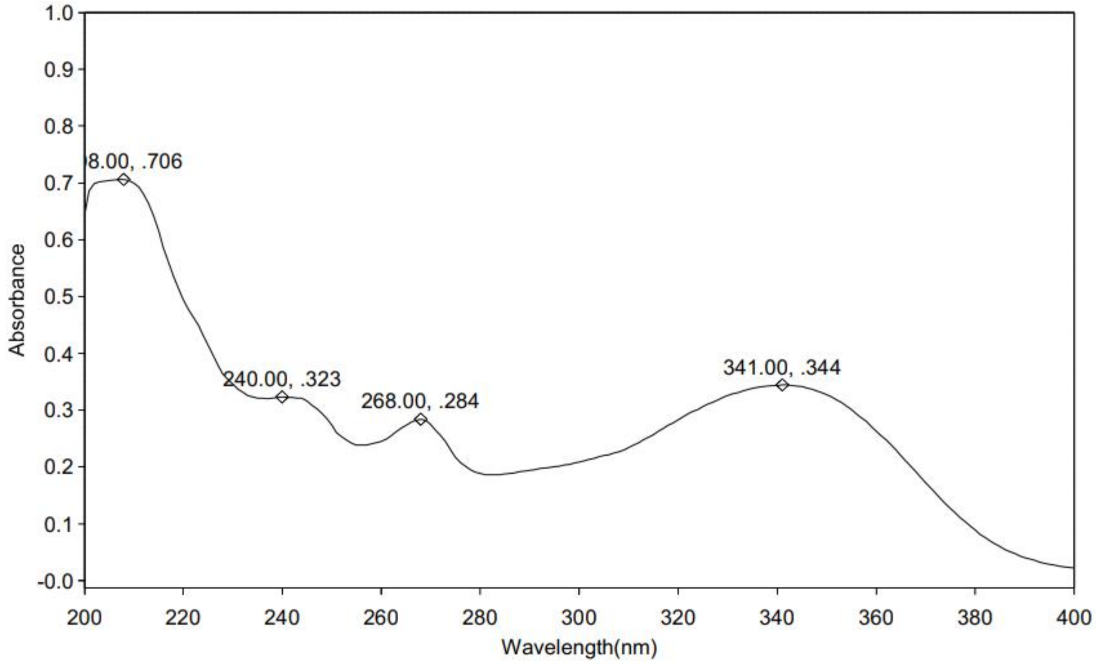

Figure S26. UV spectrum of **2a**.

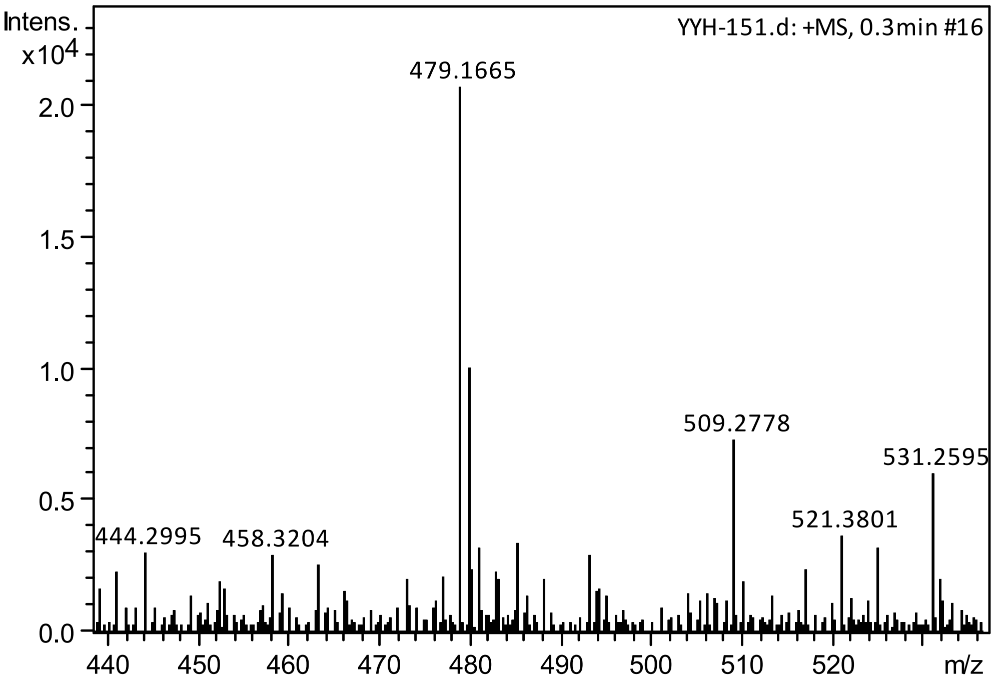

Figure S27. HRESIMS spectrum of **2a**.

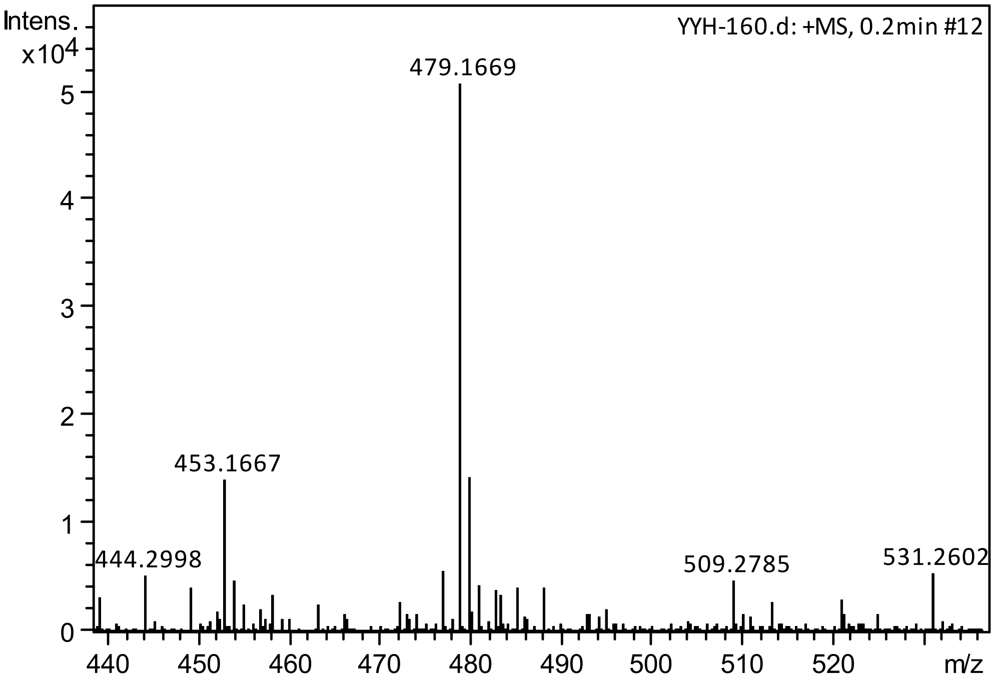

Figure S28. HRESIMS spectrum of **2b**.

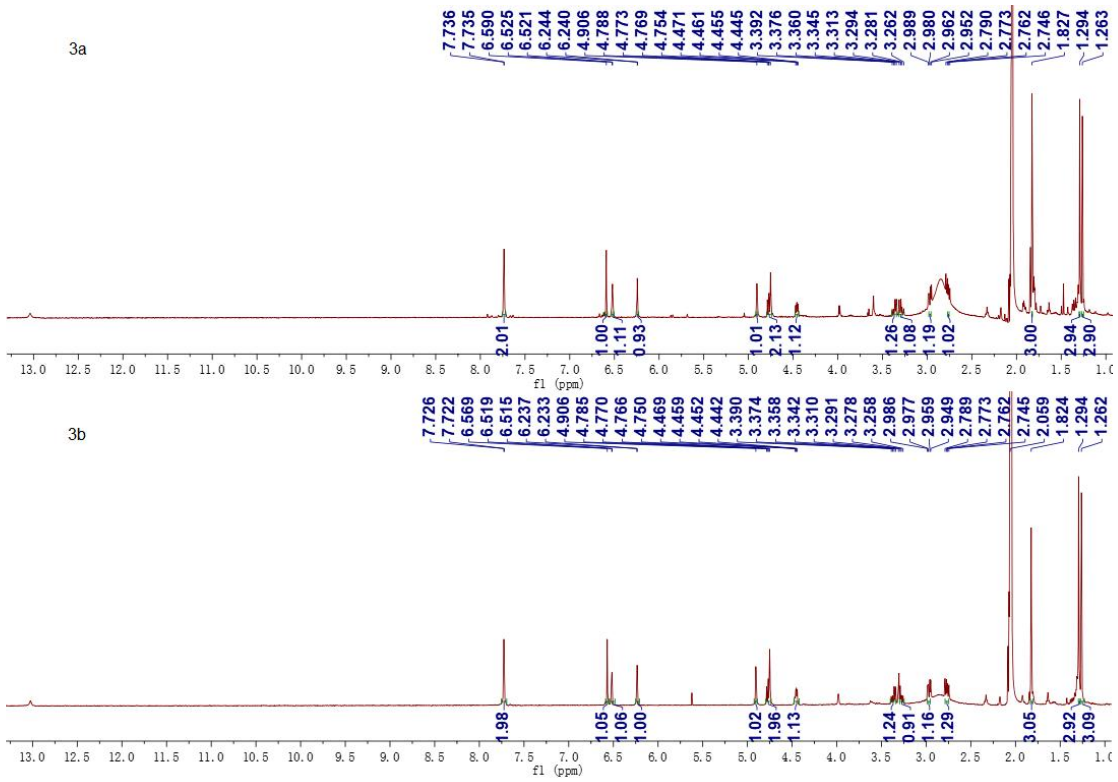

Figure S29. ^{1}H NMR (500 MHz) spectrum of **3a/3b** in Acetone-d_6.

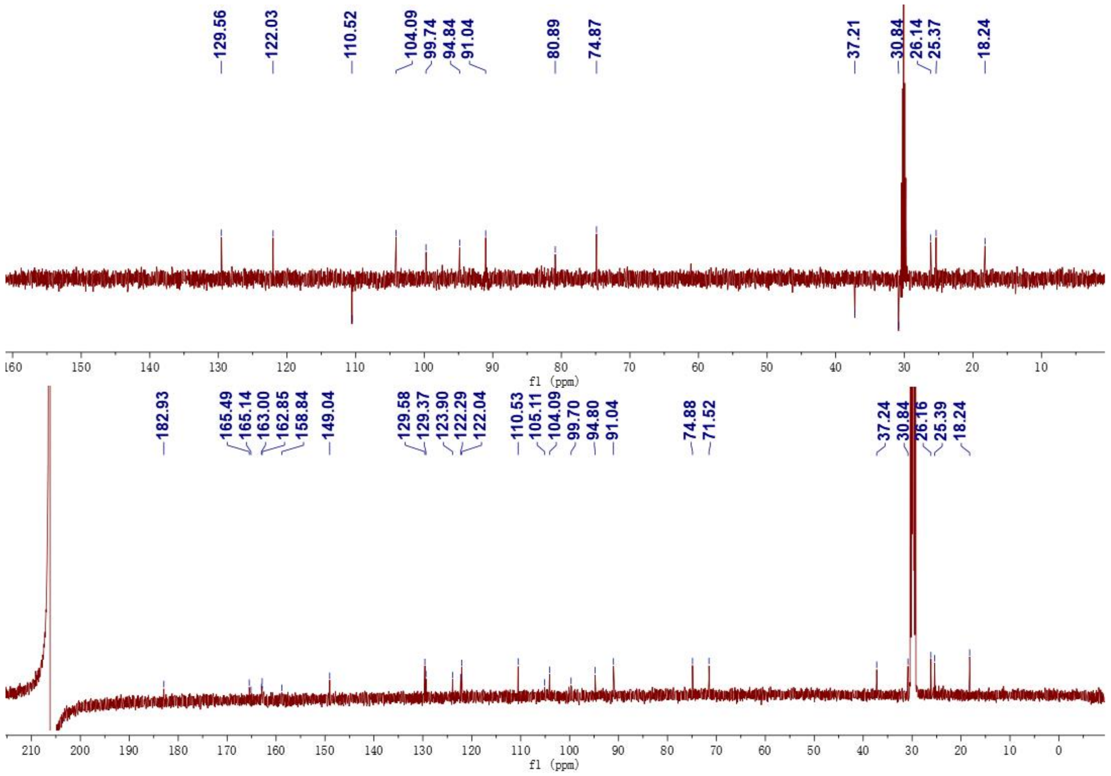

Figure S30. DEPT and ^{13}C NMR (125 MHz) spectra of **3a** in Acetone-d_6.

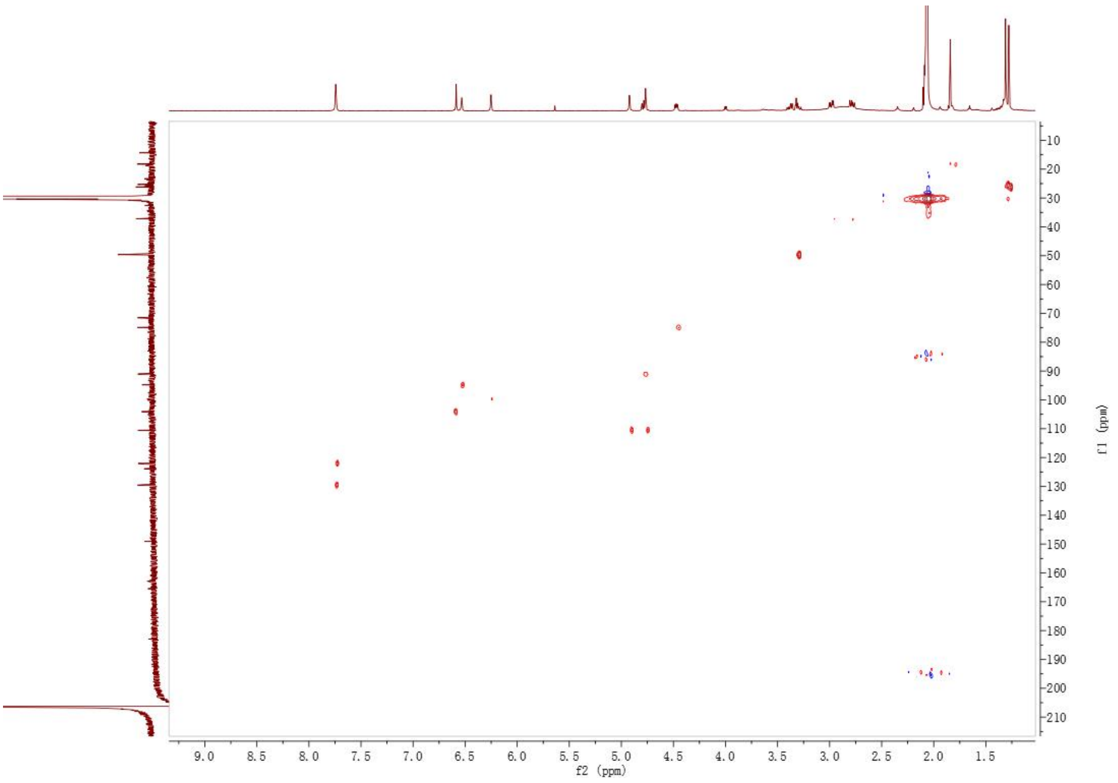

Figure S31. HSQC NMR spectrum of **3a** in Acetone-d_6.

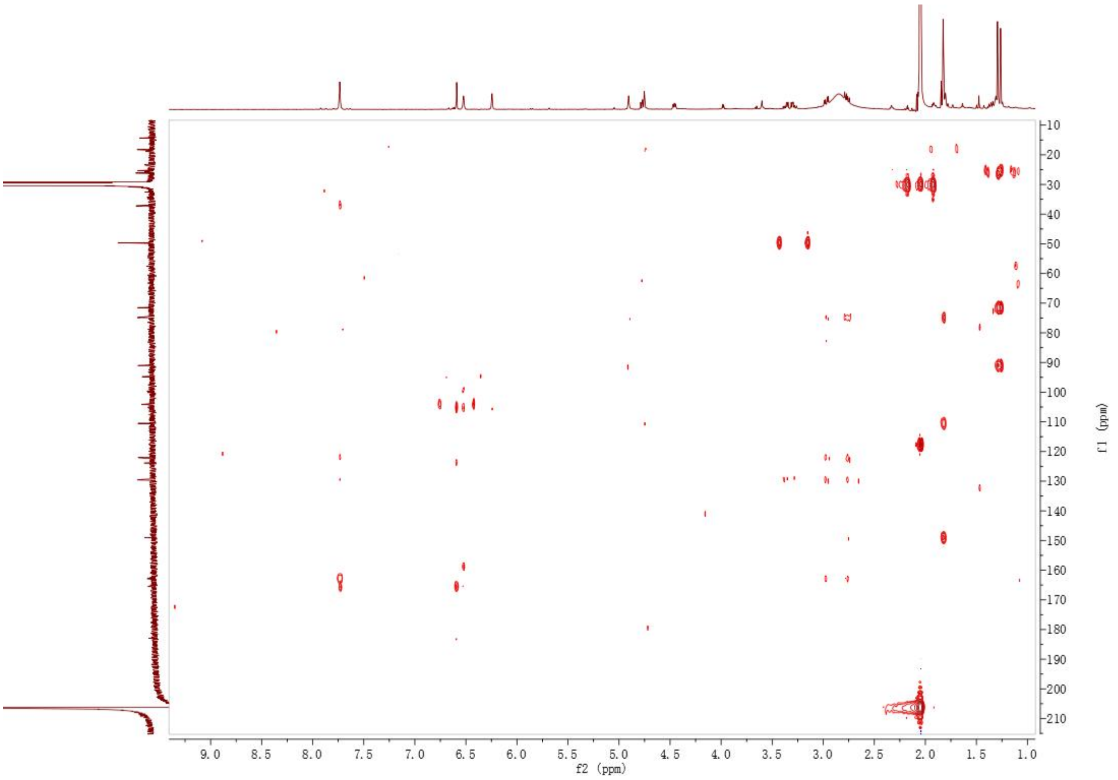

Figure S32. HMBC NMR spectrum of **3a** in Acetone-d_6.

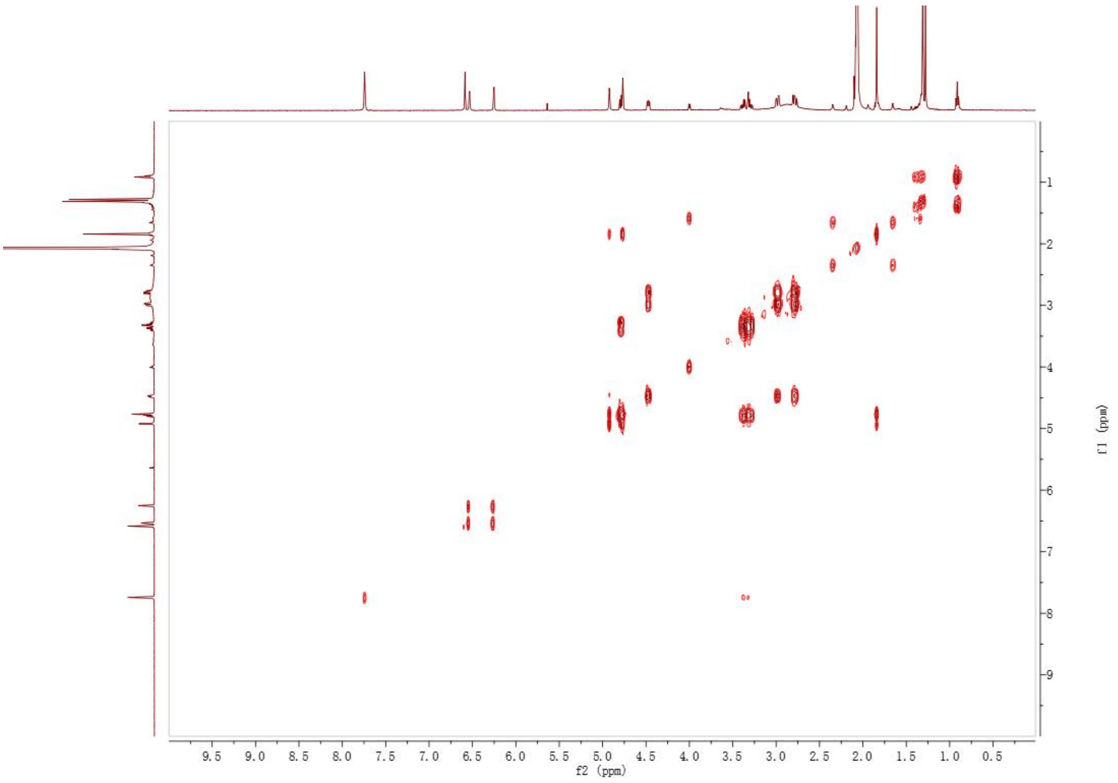

Figure S33. ^{1}H-^{1}H COSY NMR spectrum of **3a** in Acetone-d_6.

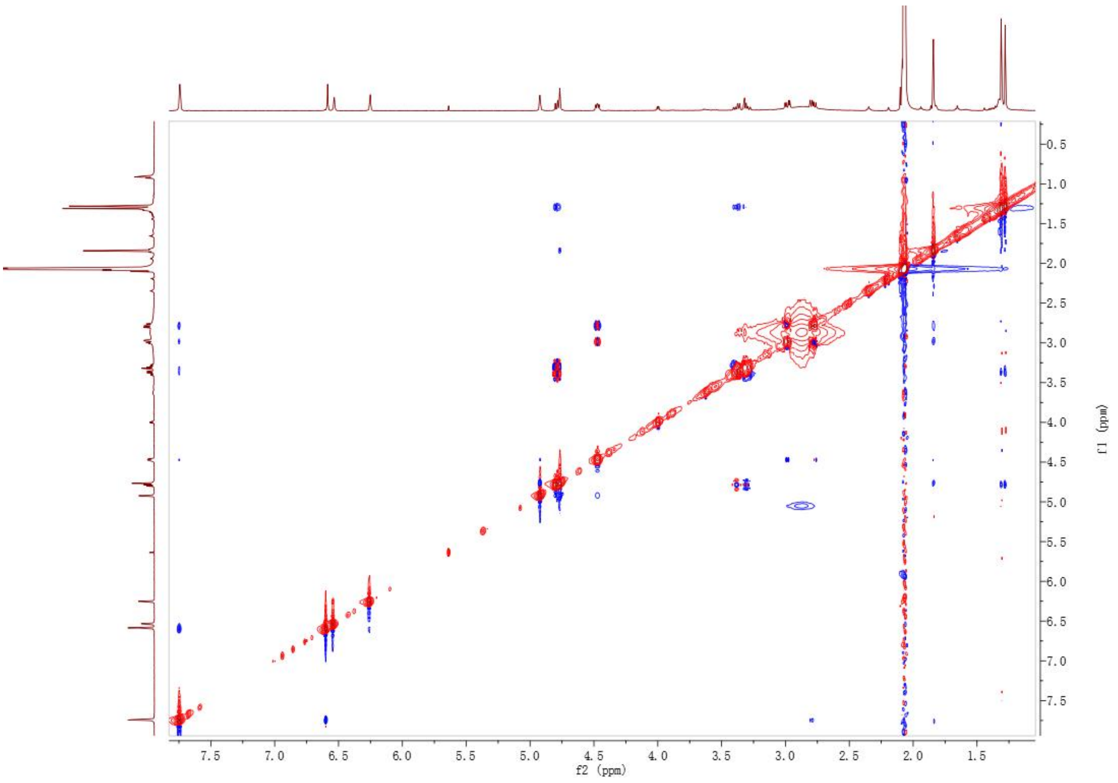

Figure S34. NOESY NMR spectrum of **3a** in Acetone-d_6.

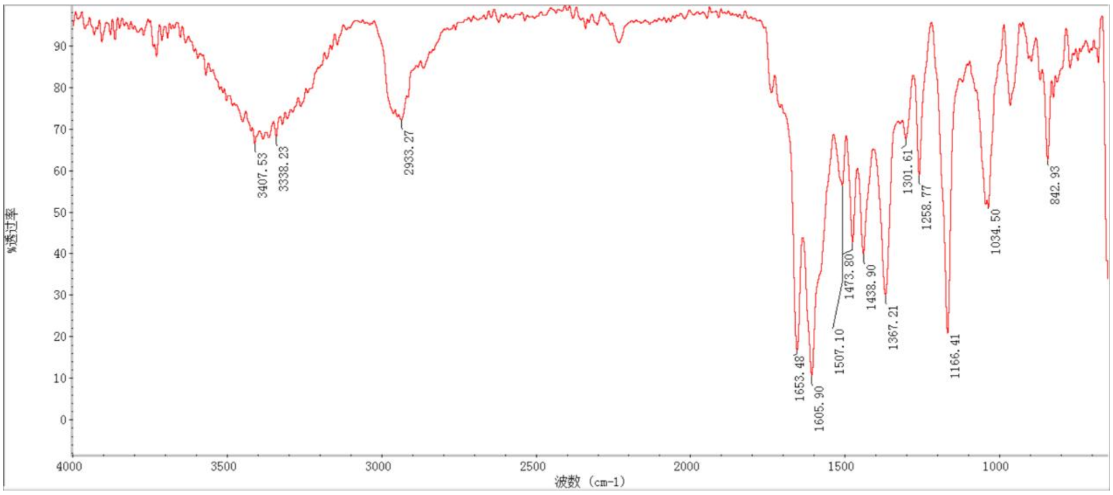

Figure S35. IR spectrum of **3a**.

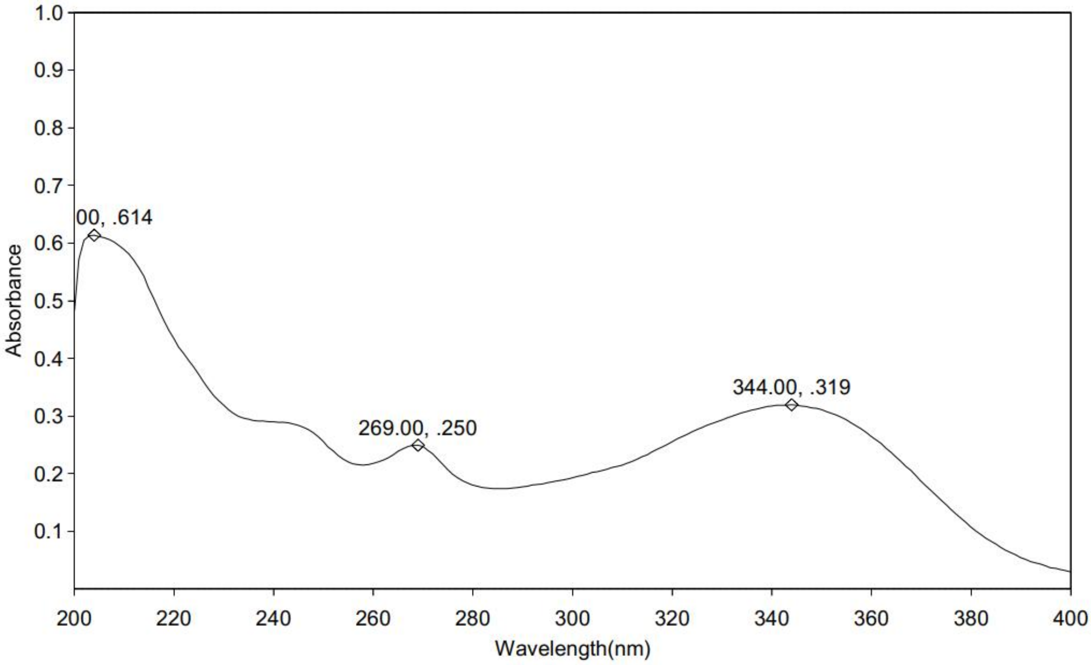

Figure S36. UV spectrum of **3a**.

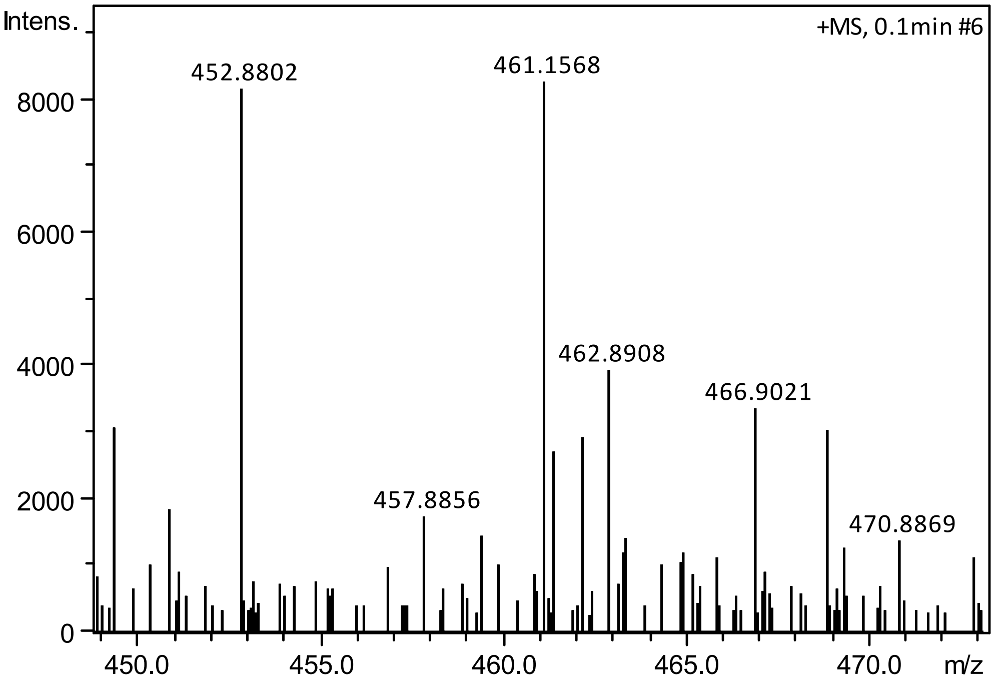

Figure S37. HRESIMS spectrum of **3a**.

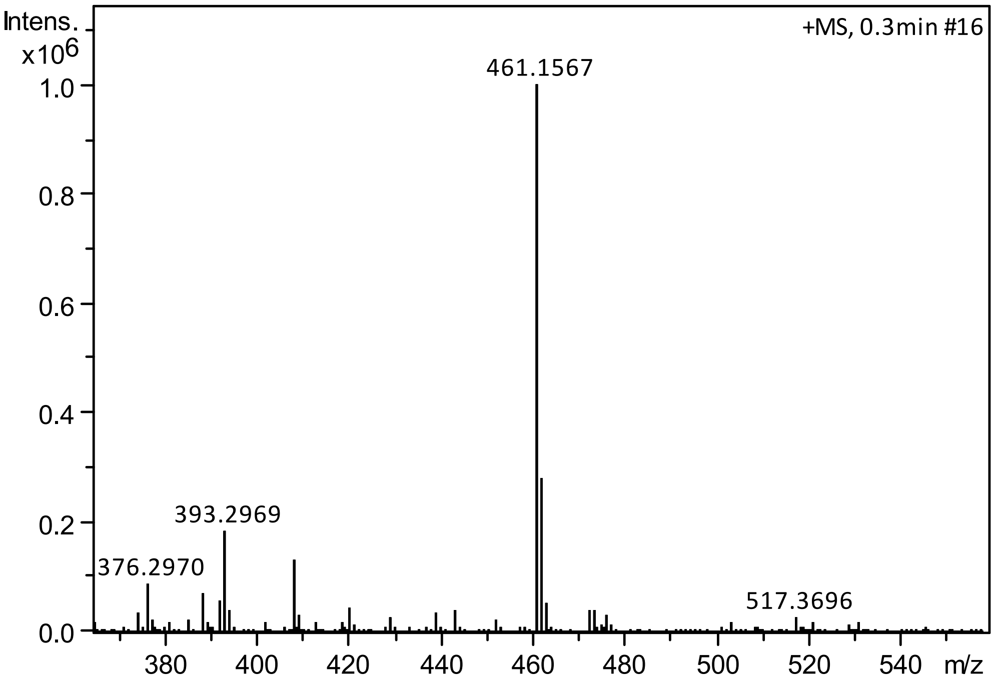

Figure S38. HRESIMS spectrum of **3b**.

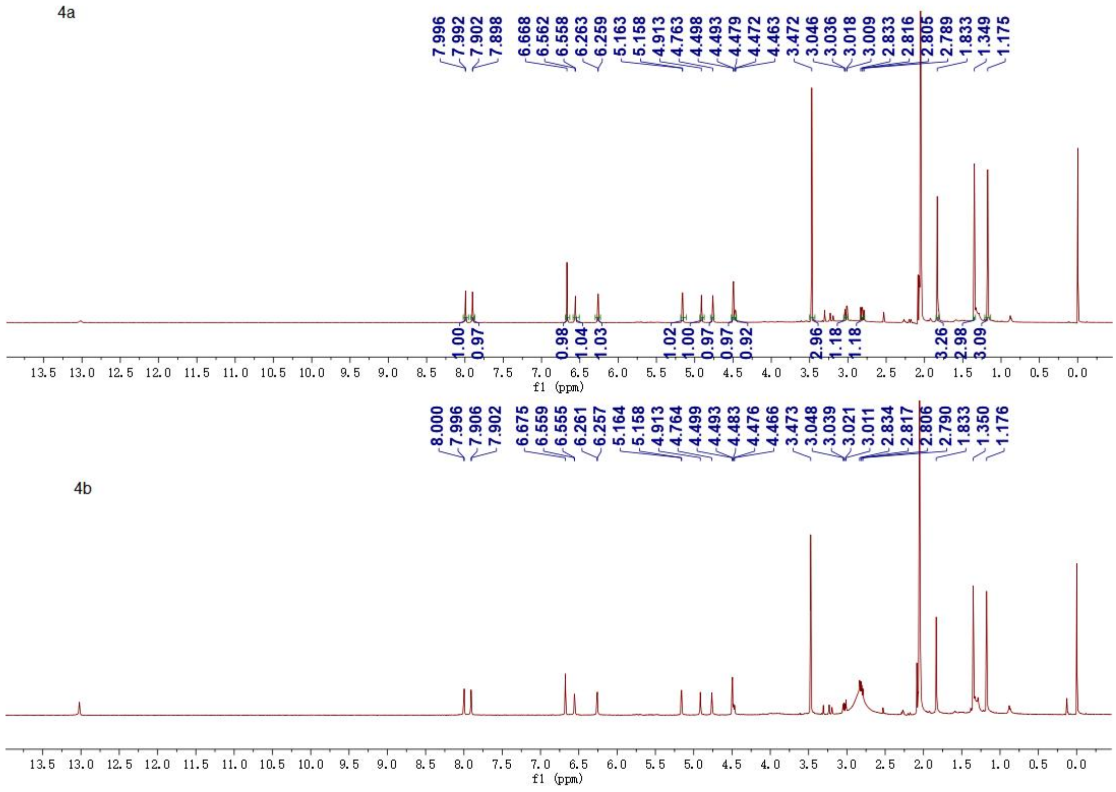

Figure S39. ^{1}H NMR (500 MHz) spectrum of **4a/4b** in Acetone-d_6.

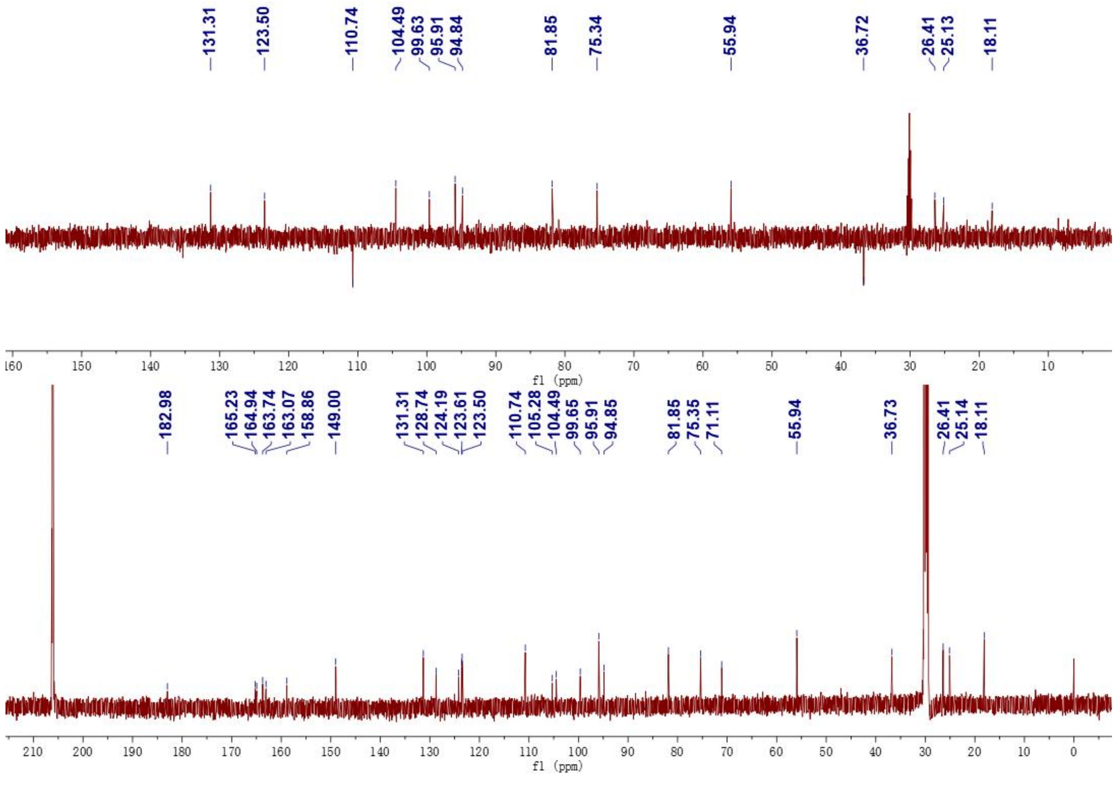

Figure S40. DEPT and ^{13}C NMR (125 MHz) spectra of **4a** in Acetone-d_6.

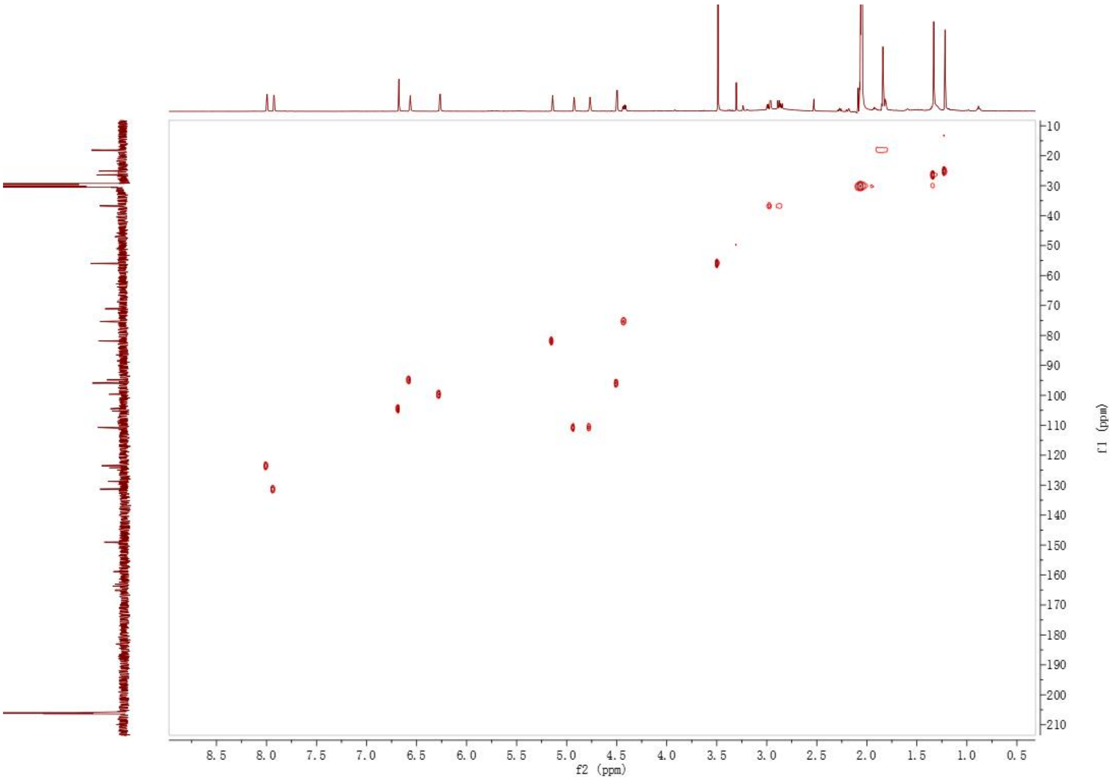

Figure S41. HSQC NMR spectrum of **4a** in Acetone-d_6.

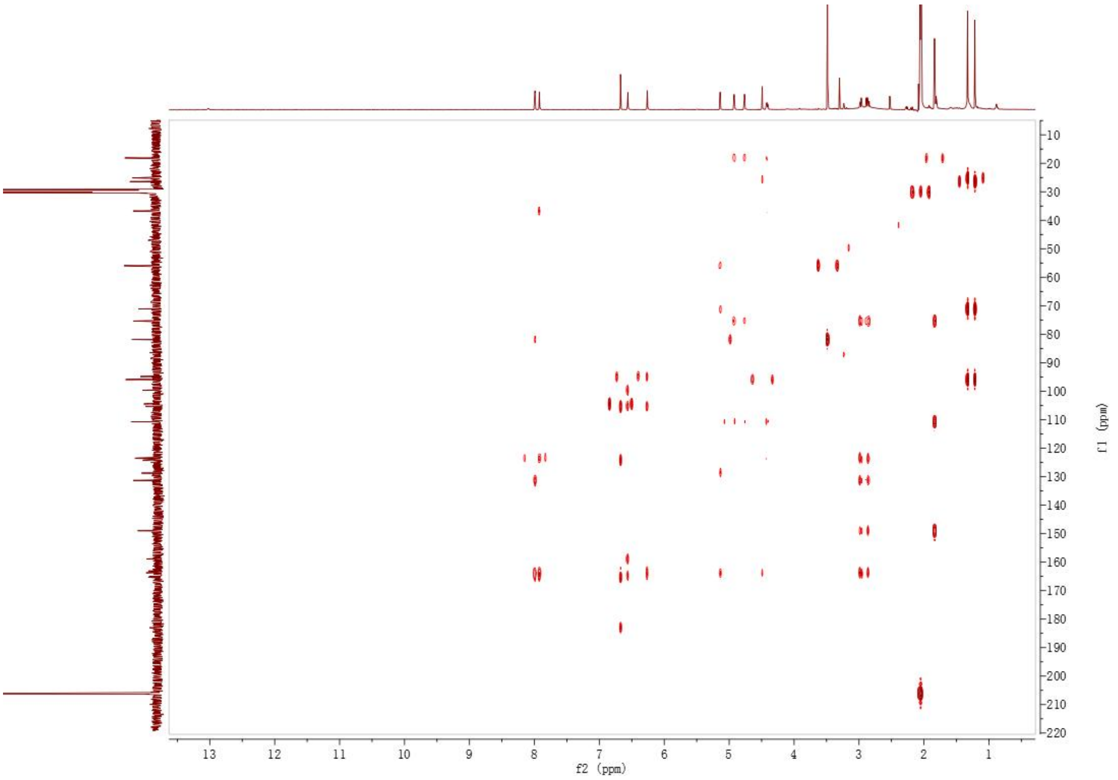

Figure S42. HMBC NMR spectrum of **4a** in Acetone-d_6.

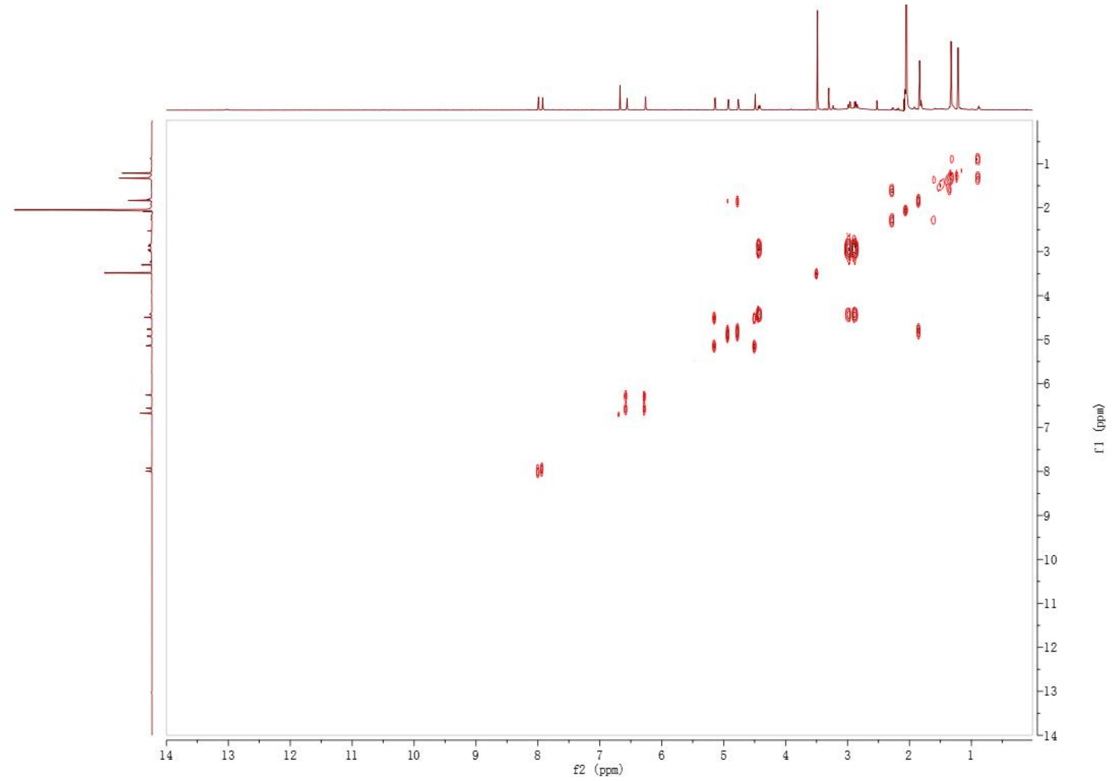

Figure S43. ^{1}H-^{1}H COSY NMR spectrum of **4a** in Acetone-d_6.

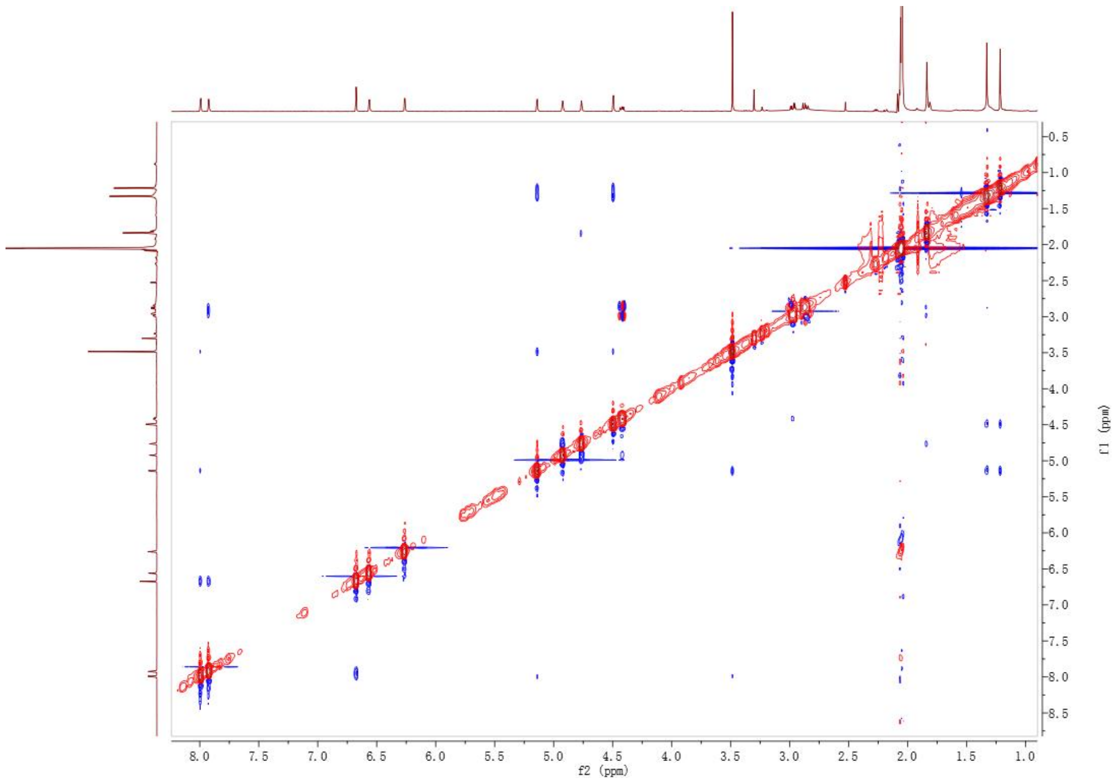

Figure S44. NOESY NMR spectrum of **4a** in Acetone-d_6.

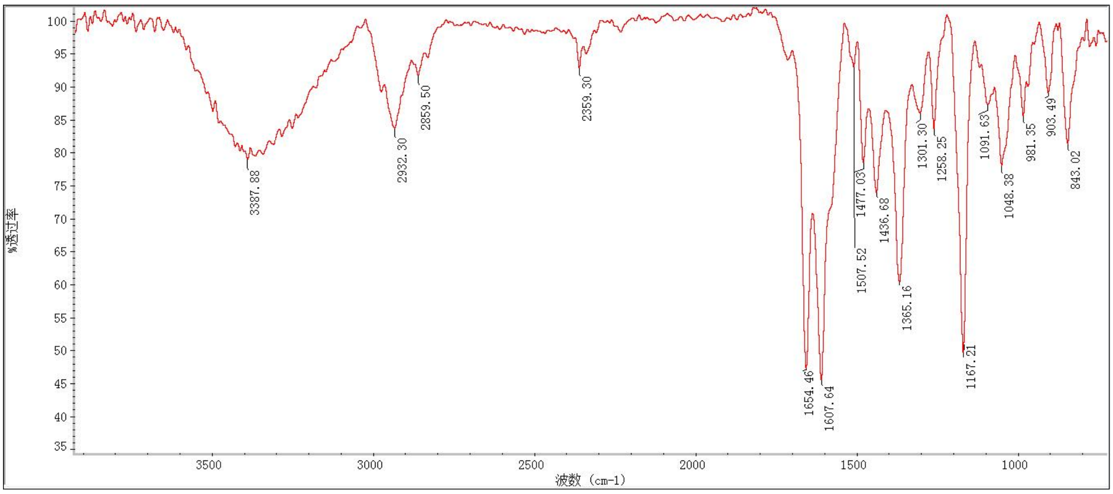

Figure S45. IR spectrum of **4a**.

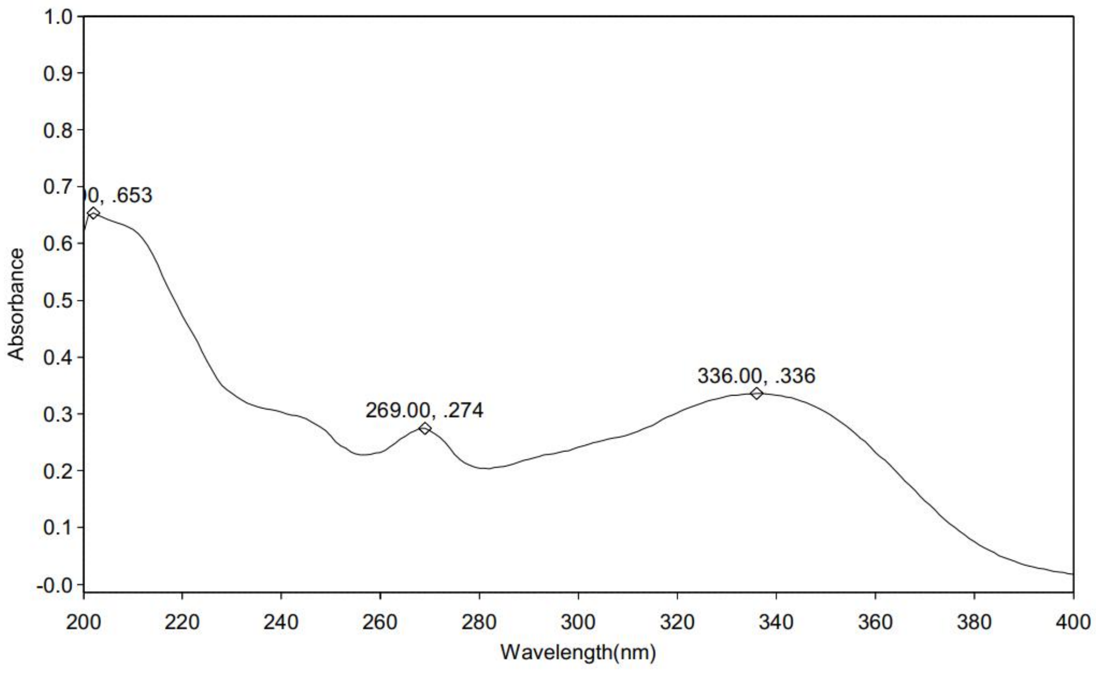

Figure S46. UV spectrum of **4a**.

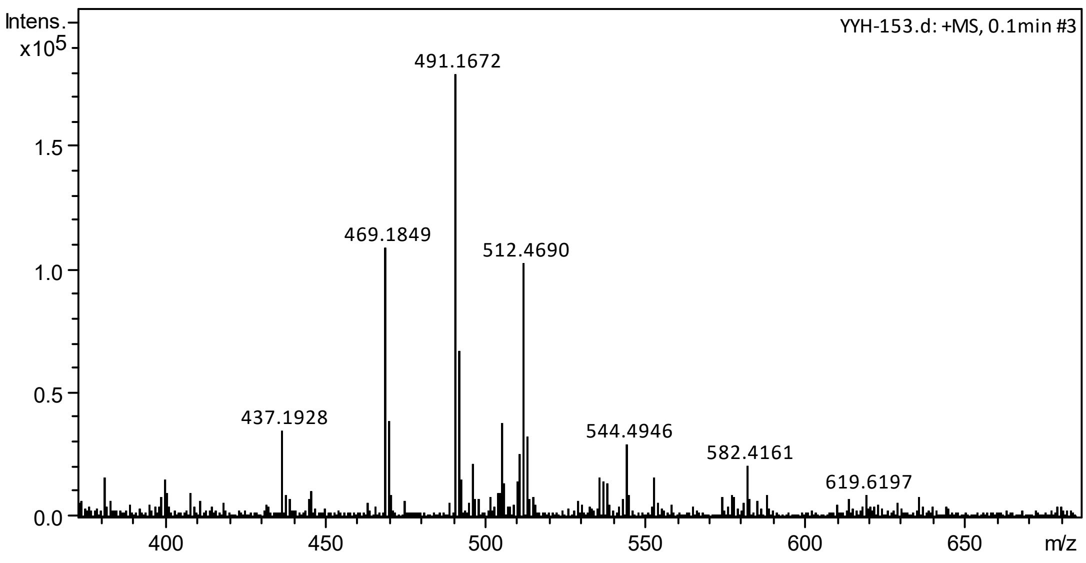

Figure S47. HRESIMS spectrum of **4a**.

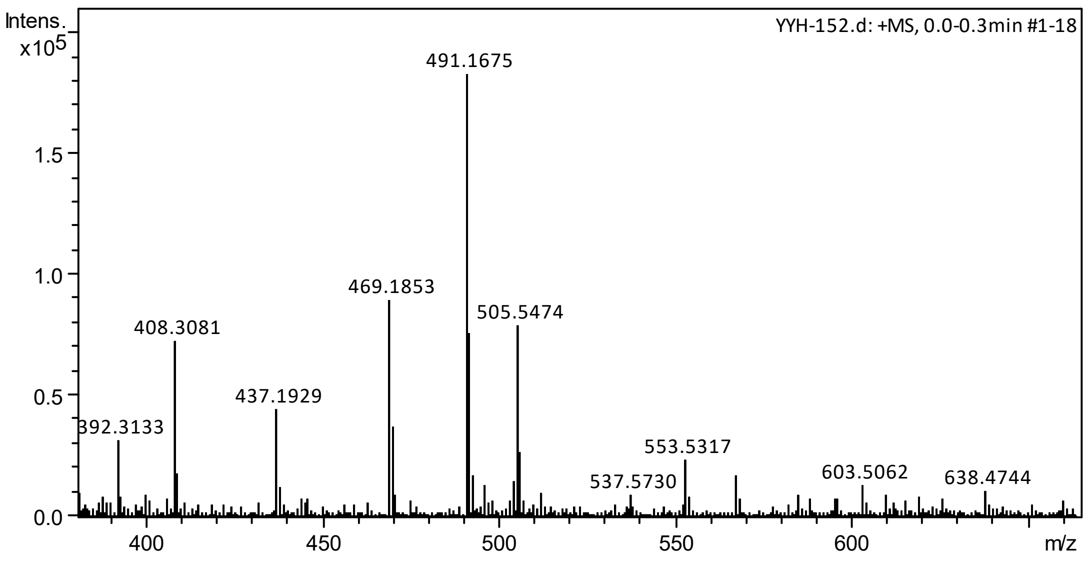

Figure S48. HRESIMS spectrum of **4b**.

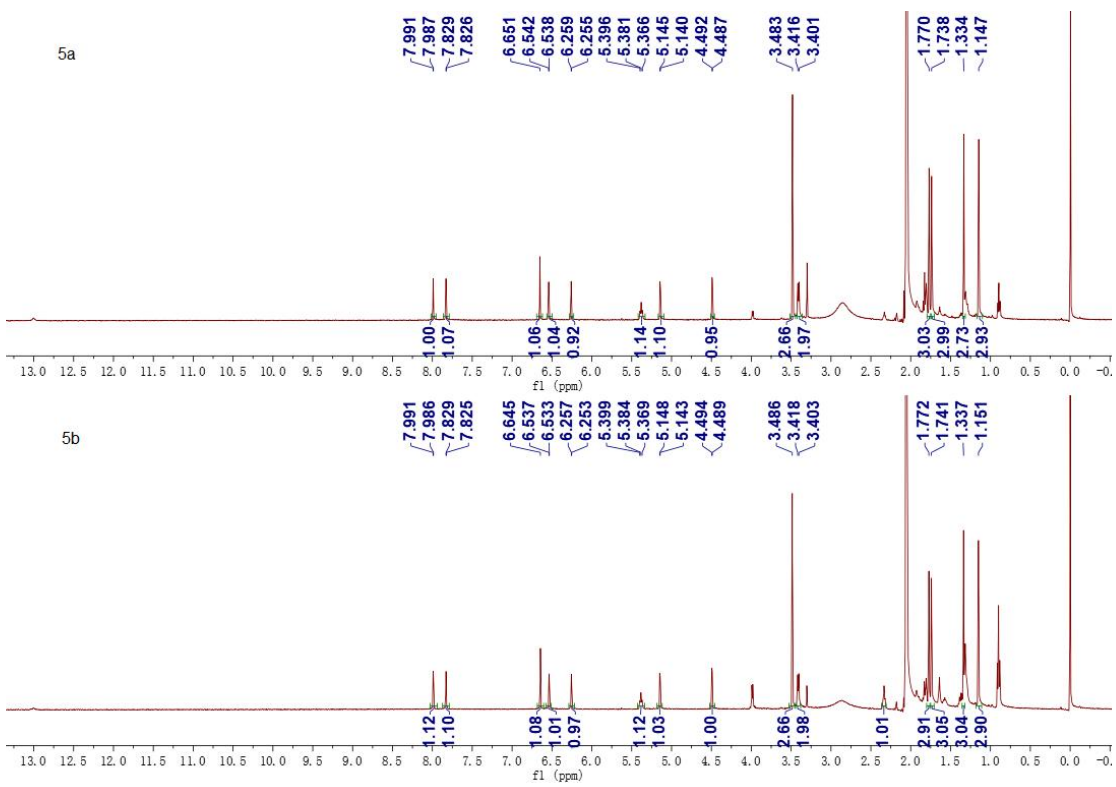

Figure S49. ^{1}H NMR (500 MHz) spectrum of **5a/5b** in Acetone-d_6.

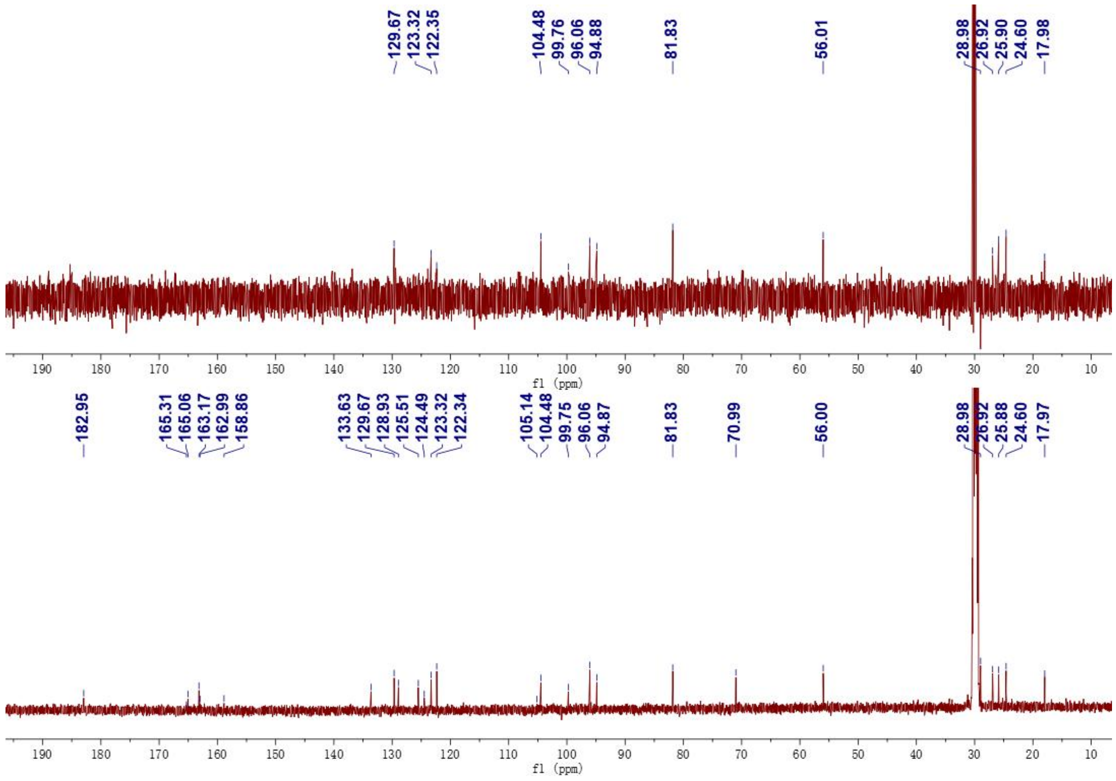

Figure S50. DEPT and ^{13}C NMR (125 MHz) spectra of **5a** in Acetone-d_6.

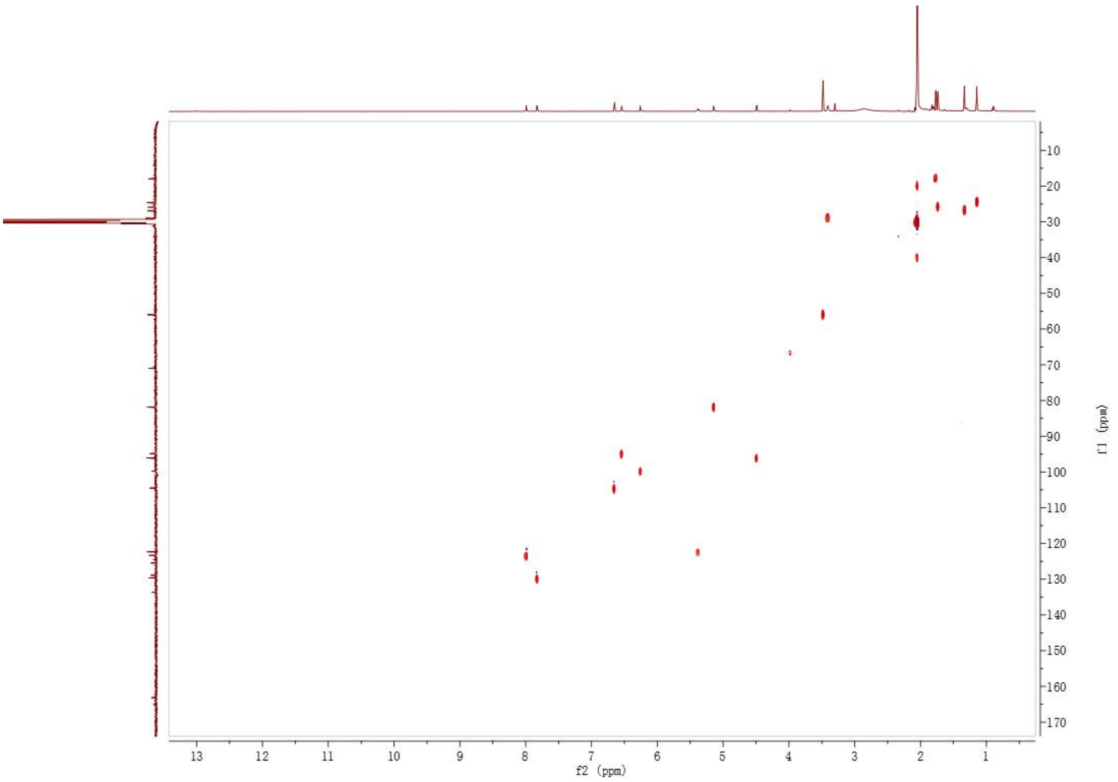

Figure S51. HSQC NMR spectrum of **5a** in Acetone-d_6.

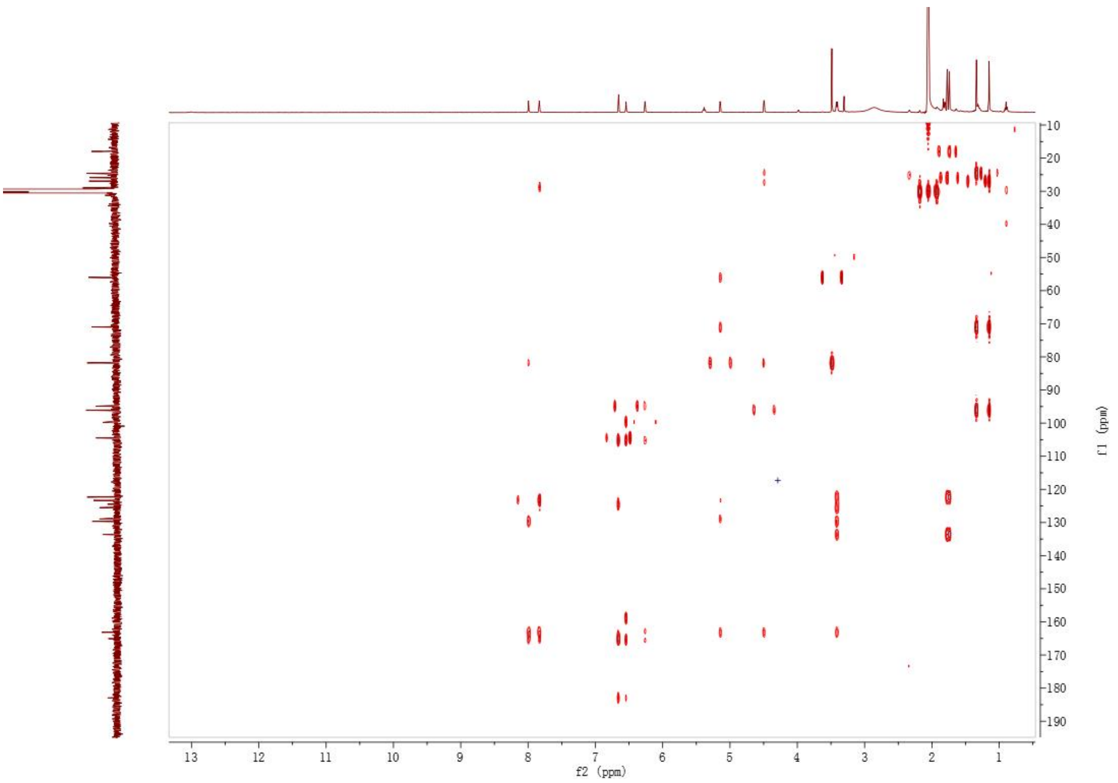

Figure S52. HMBC NMR spectrum of **5a** in Acetone-d_6.

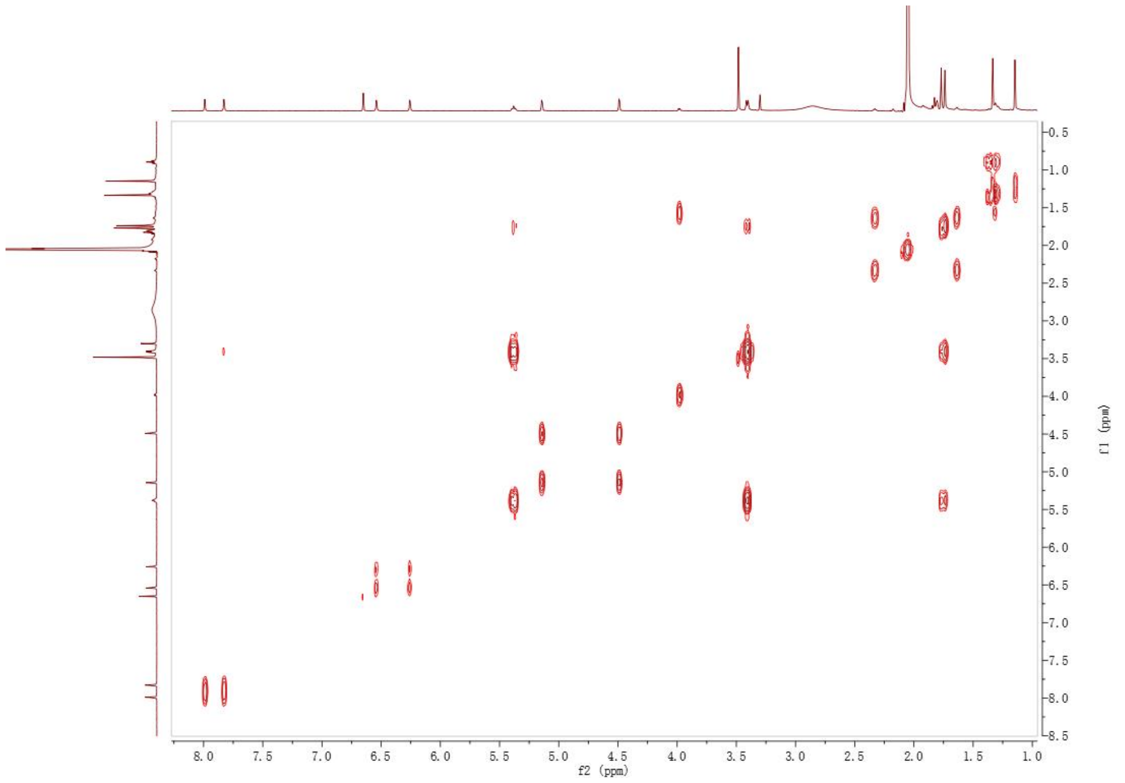

Figure S53. ^{1}H-^{1}H COSY NMR spectrum of **5a** in Acetone-d_6.

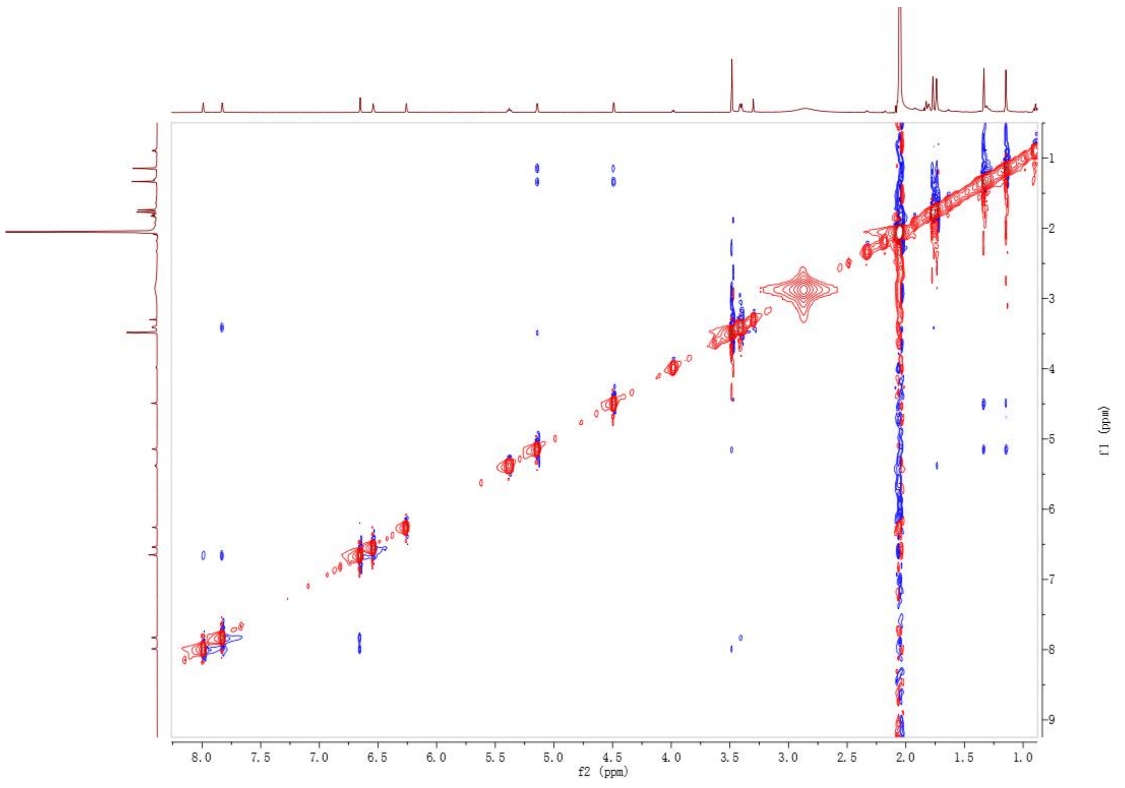

Figure S54. NOESY NMR spectrum of **5a** in Acetone-d_6.

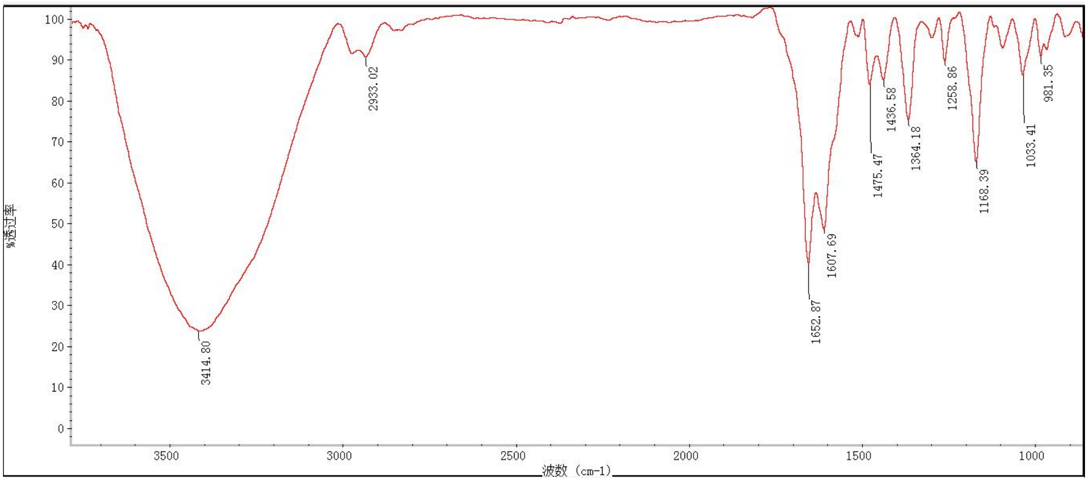

Figure S55. IR spectrum of **5a**.

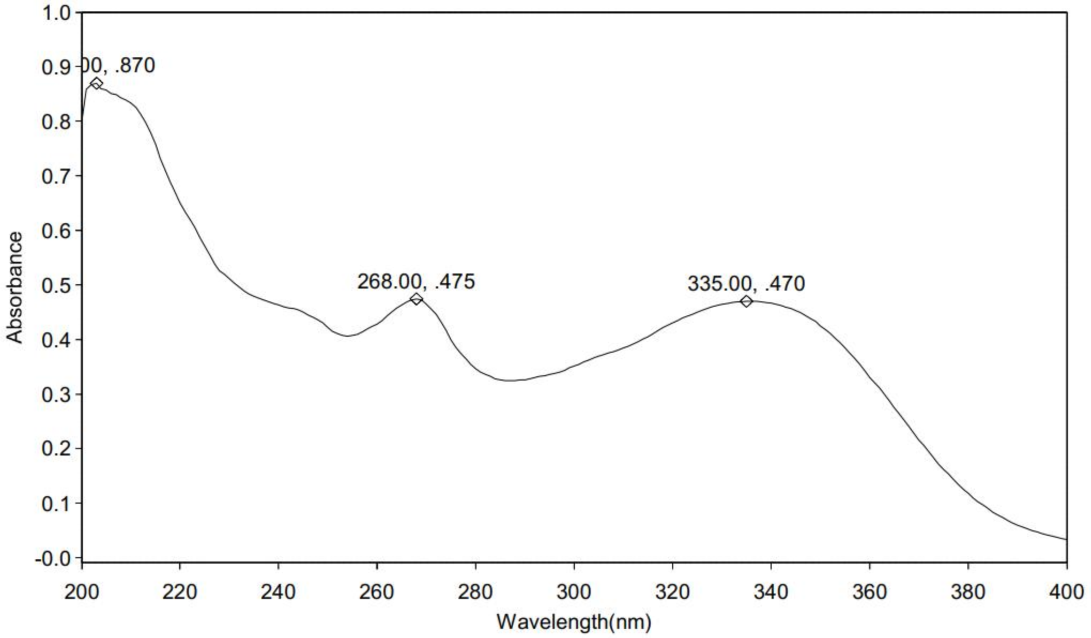

Figure S56. UV spectrum of **5a**.

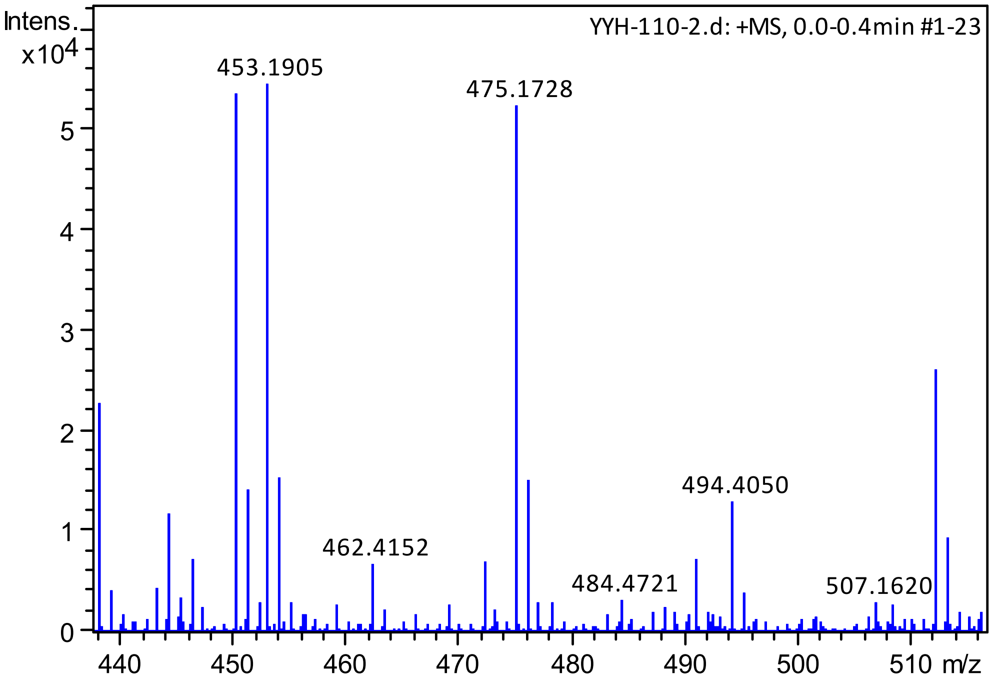

Figure S57. HRESIMS spectrum of **5a**.

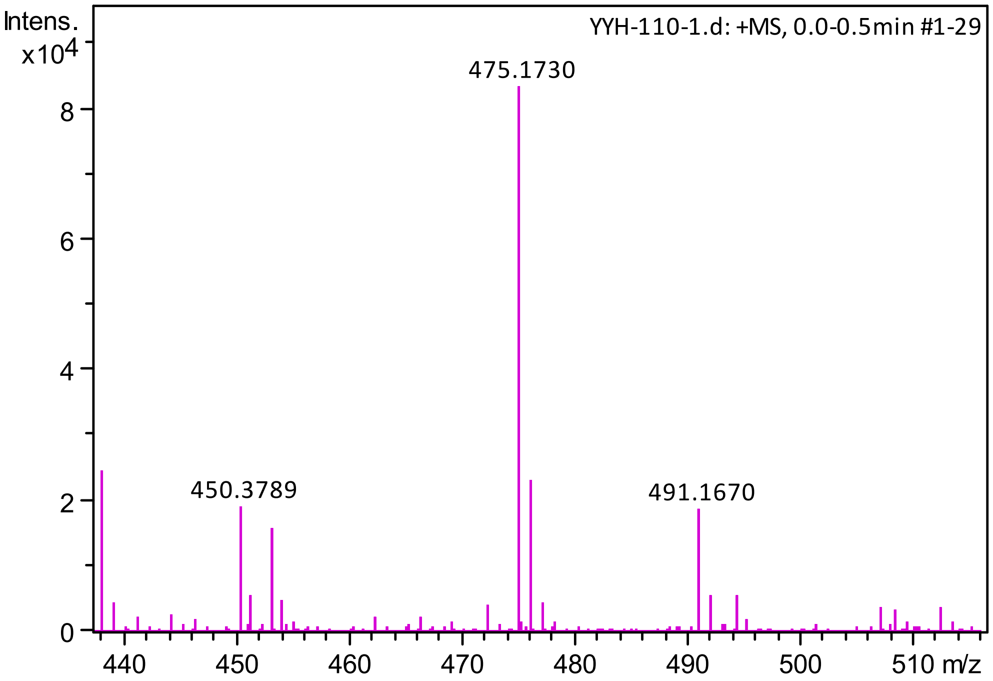

Figure S58. HRESIMS spectrum of **5b**.

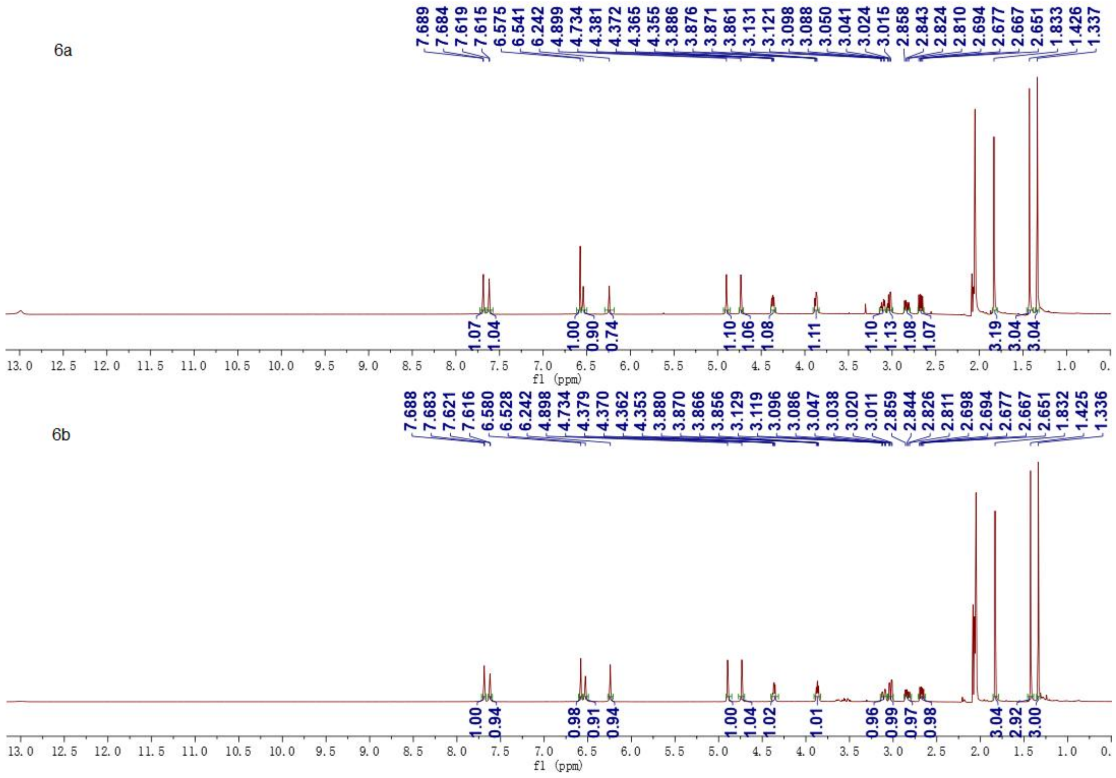

Figure S59. ^{1}H NMR (500 MHz) spectrum of **6a/6b** in Acetone-d_6.

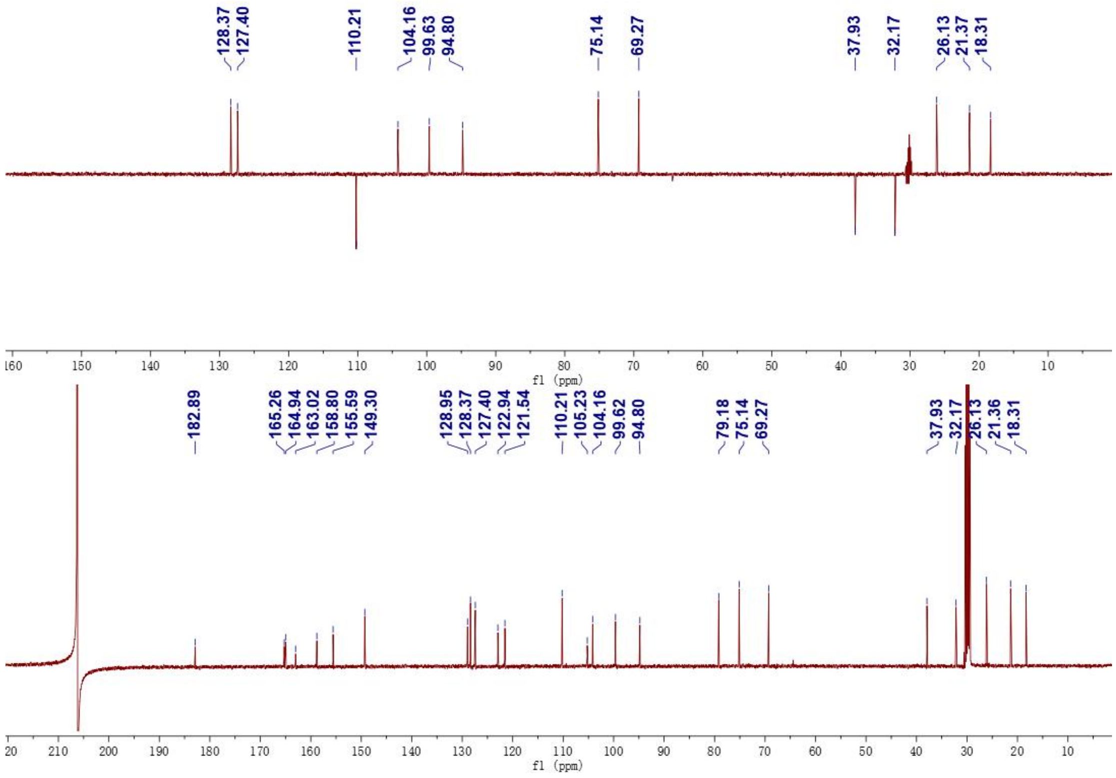

Figure S60. DEPT and ^{13}C NMR (125 MHz) spectra of **6a** in Acetone-d_6.

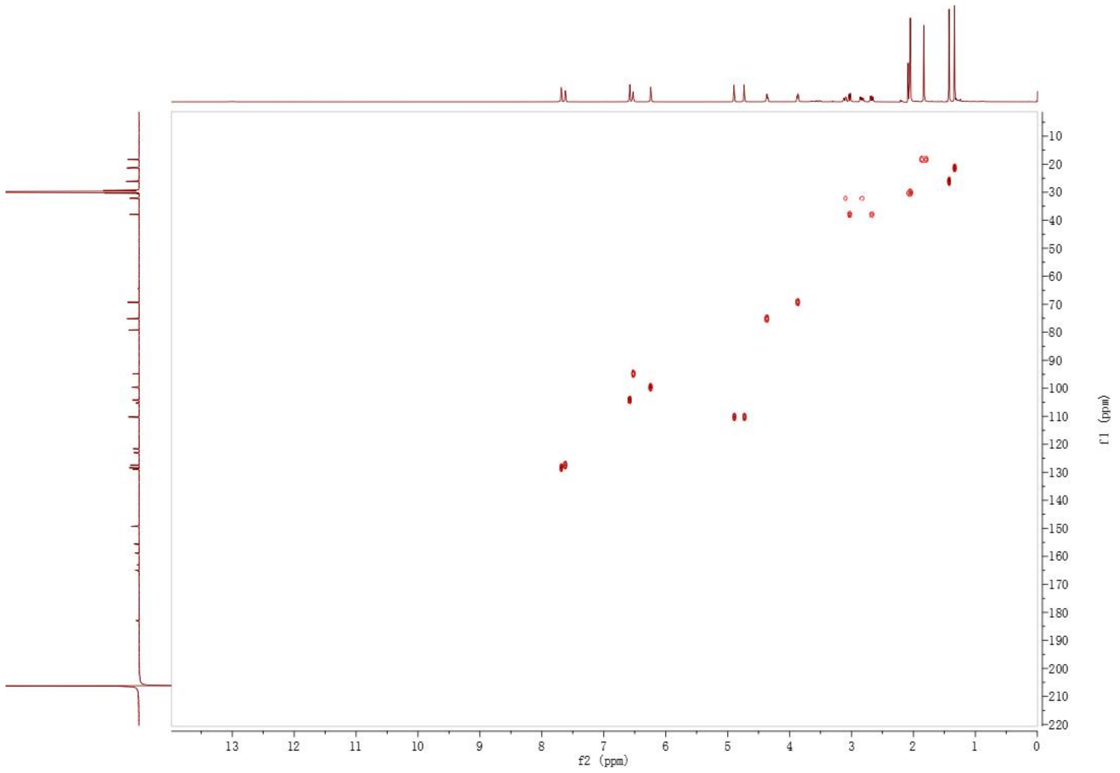

Figure S61. HSQC NMR spectrum of **6a** in Acetone-d_6.

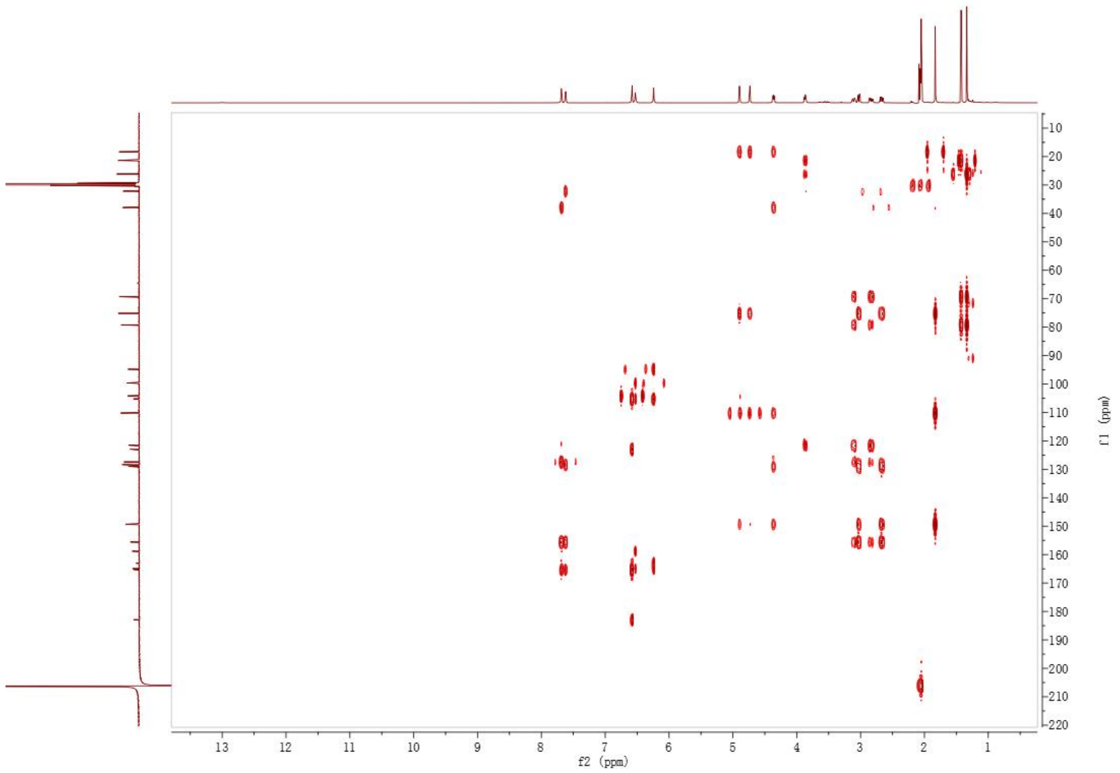

Figure S62. HMBC NMR spectrum of **6a** in Acetone-d_6.

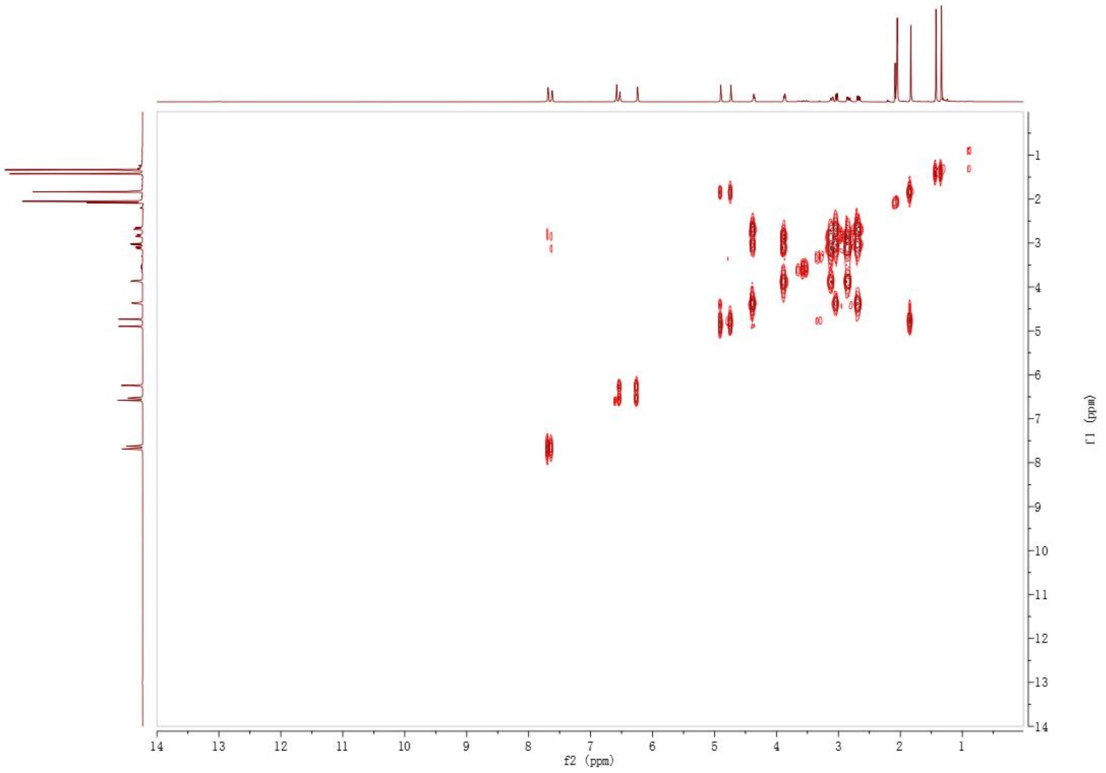

Figure S63. ^{1}H-^{1}H COSY NMR spectrum of **6a** in Acetone-d_6.

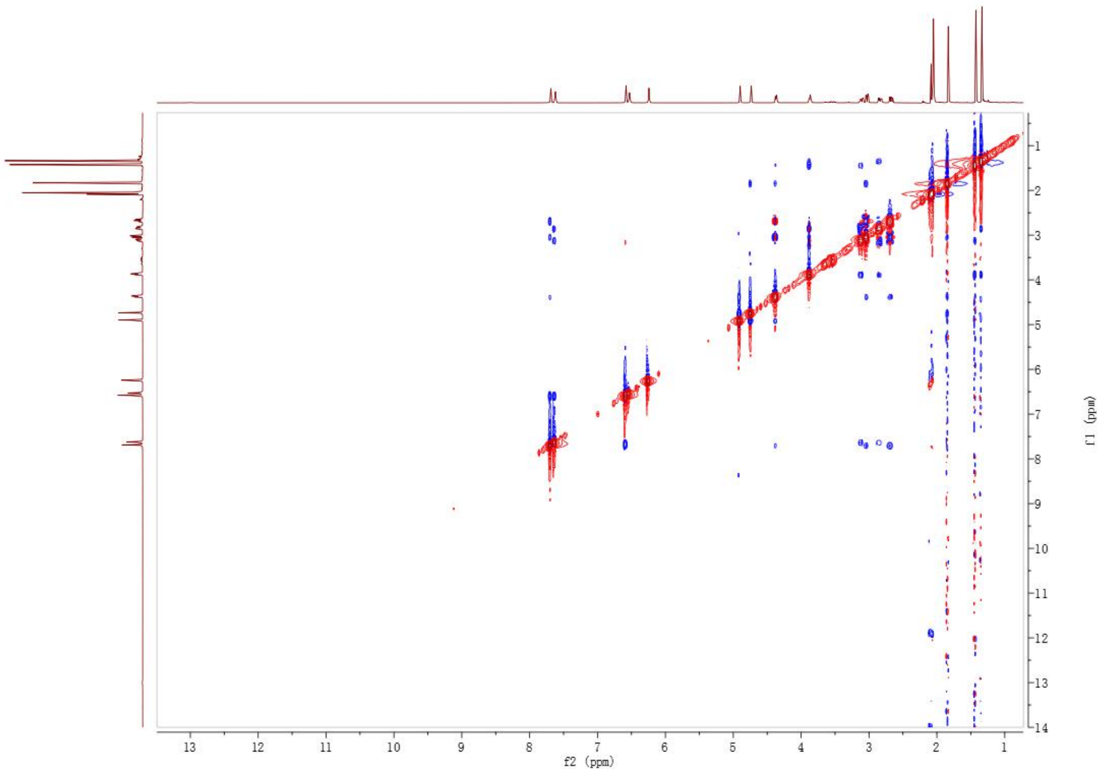

Figure S64. NOESY NMR spectrum of **6a** in Acetone-d_6.

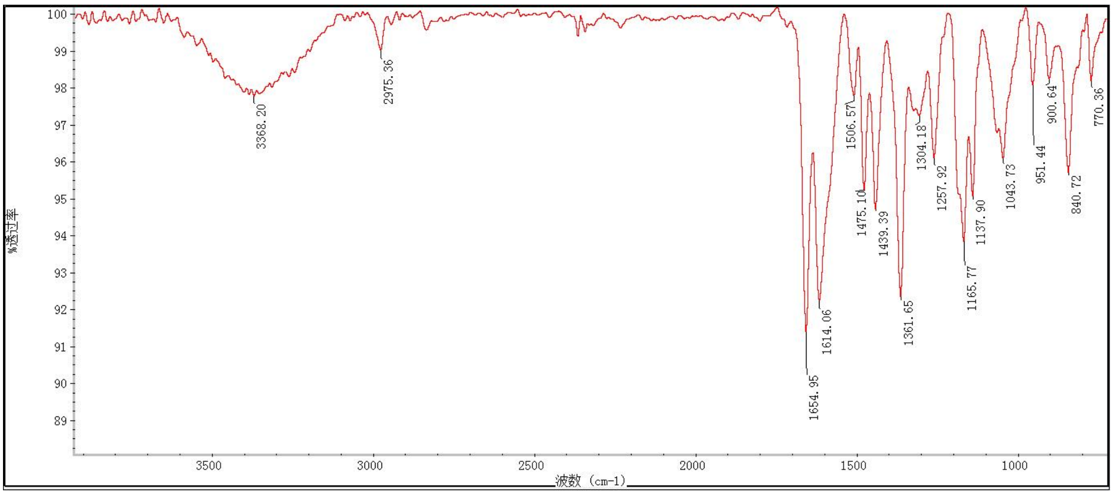

Figure S65. IR spectrum of **6a**.

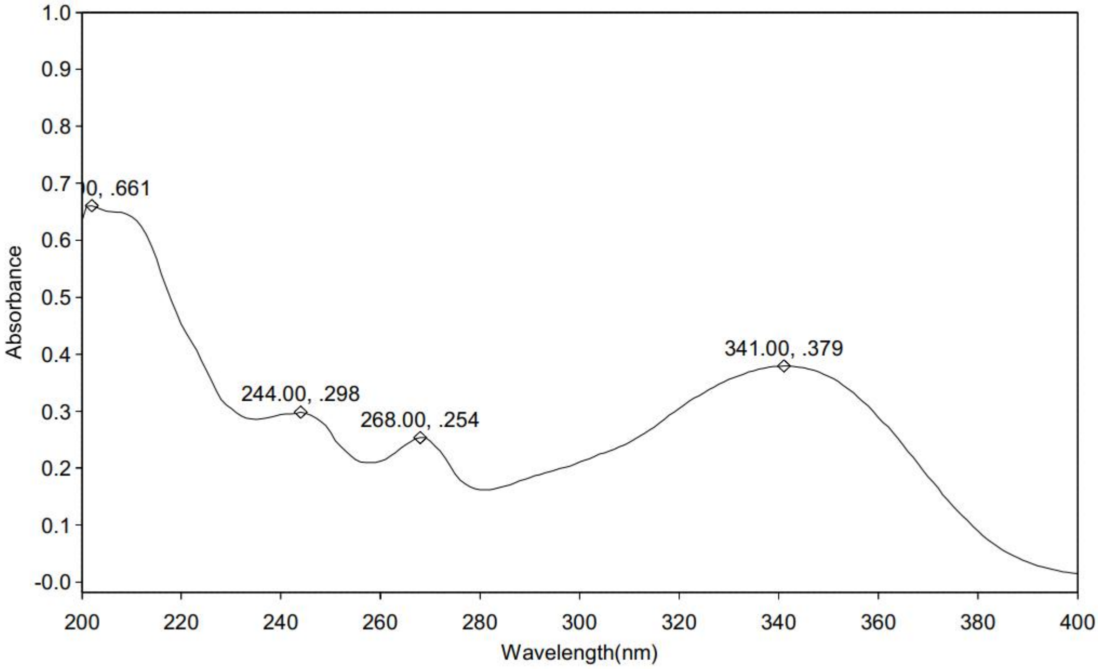

Figure S66. UV spectrum of **6a**.

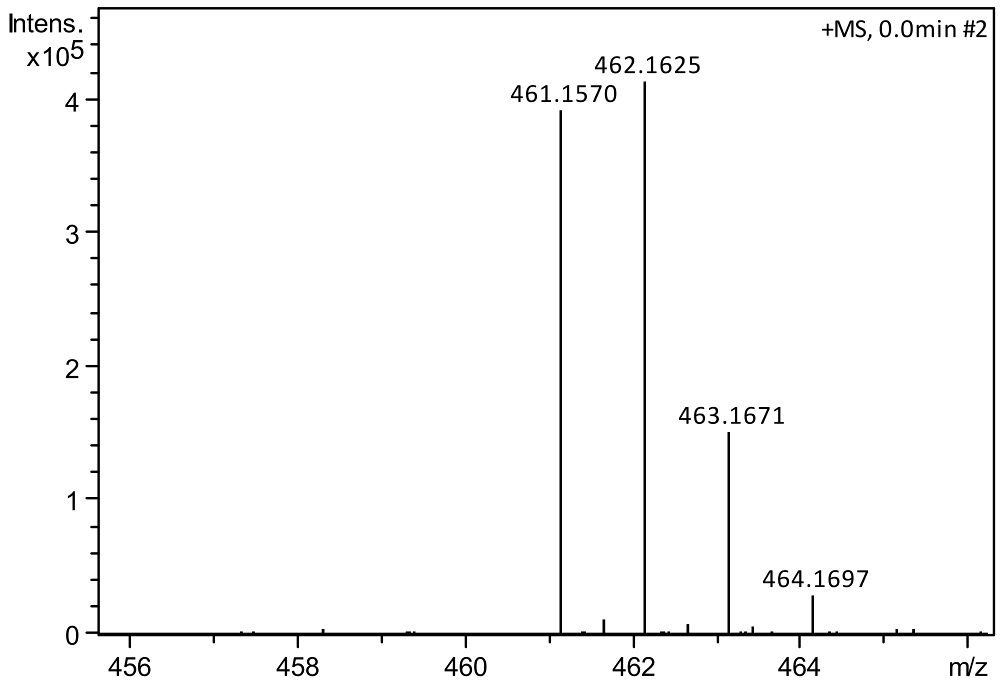

Figure S67. HRESIMS spectrum of **6a**.

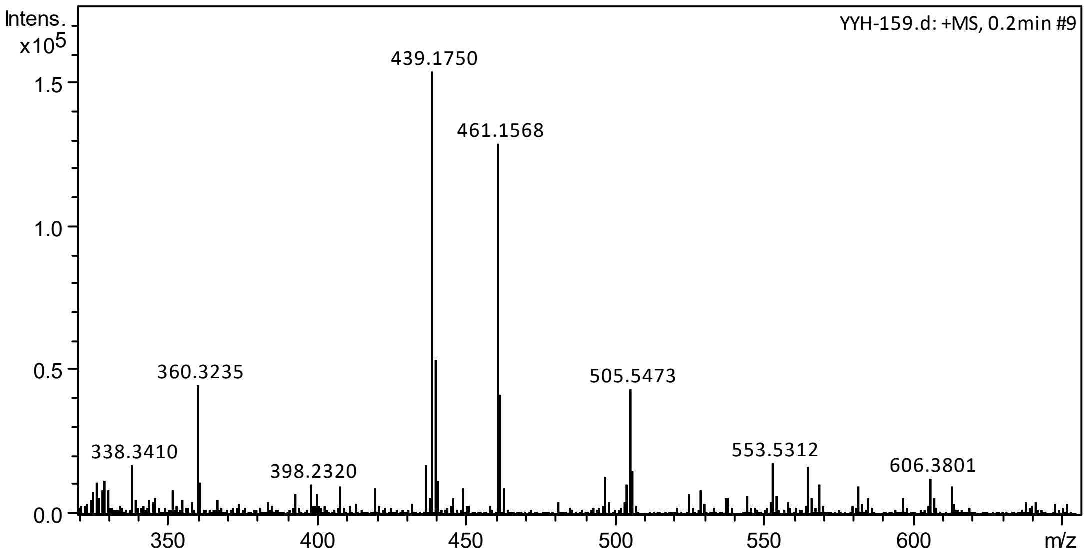

Figure S68. HRESIMS spectrum of **6b**.

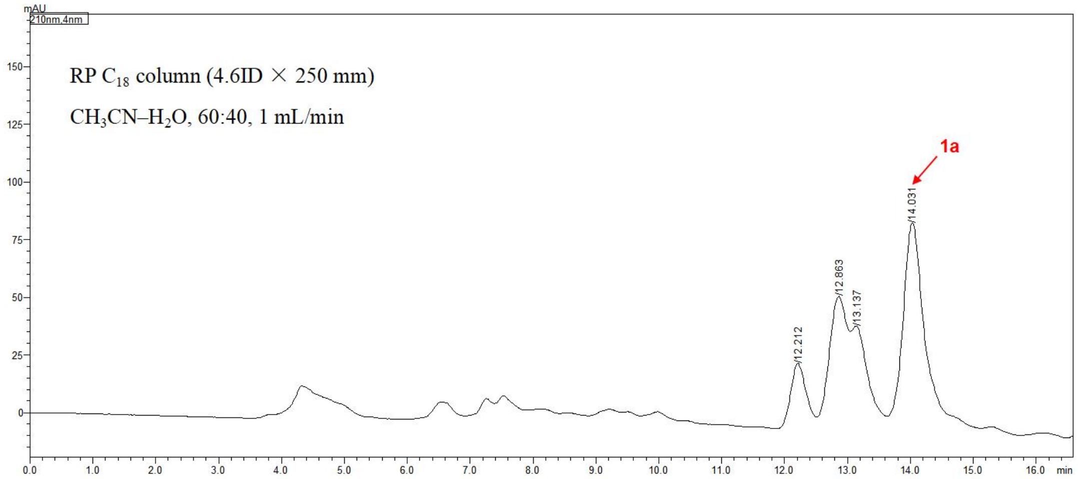

Figure S69. HPLC separation of **1a**.

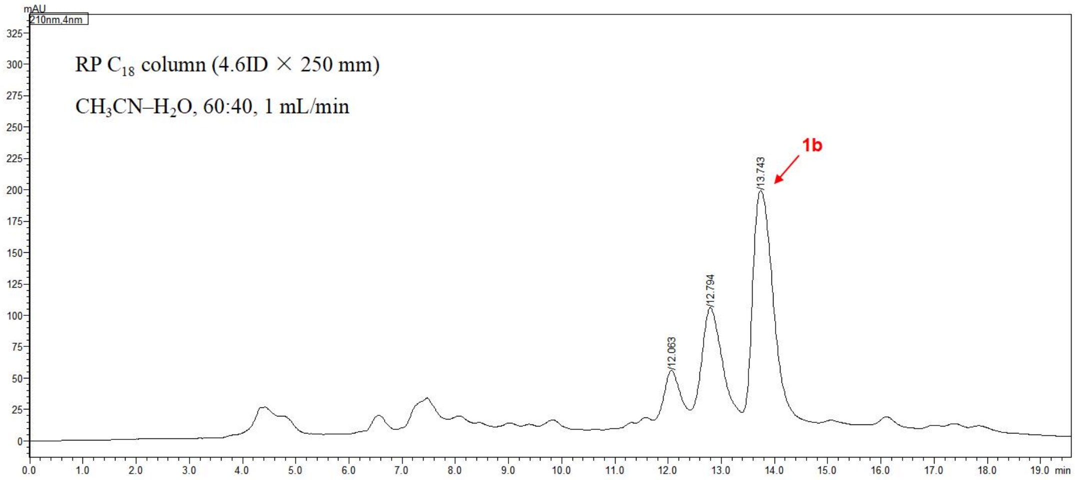

Figure S70. HPLC separation of **1b**.

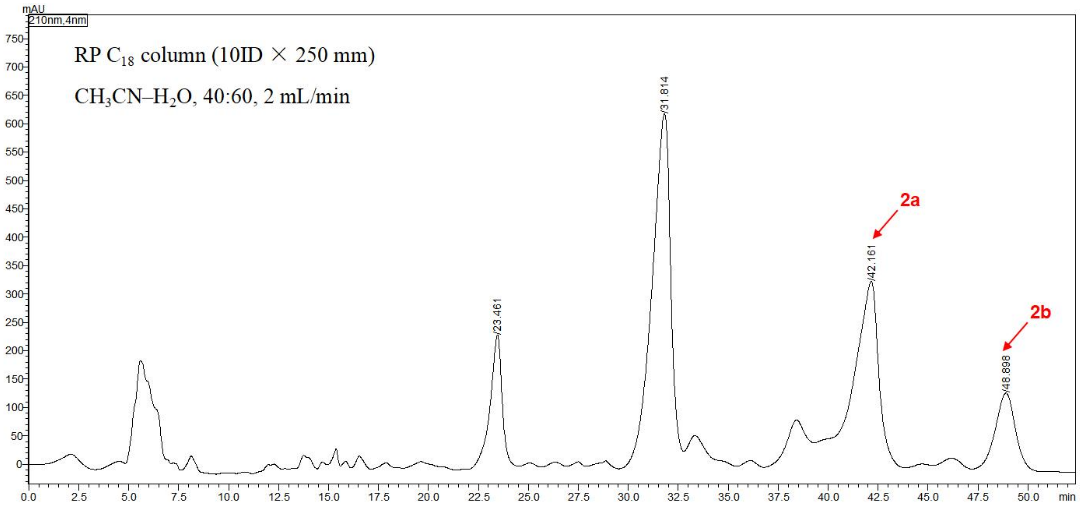

Figure S71. HPLC separation of **2a** and **2b**.

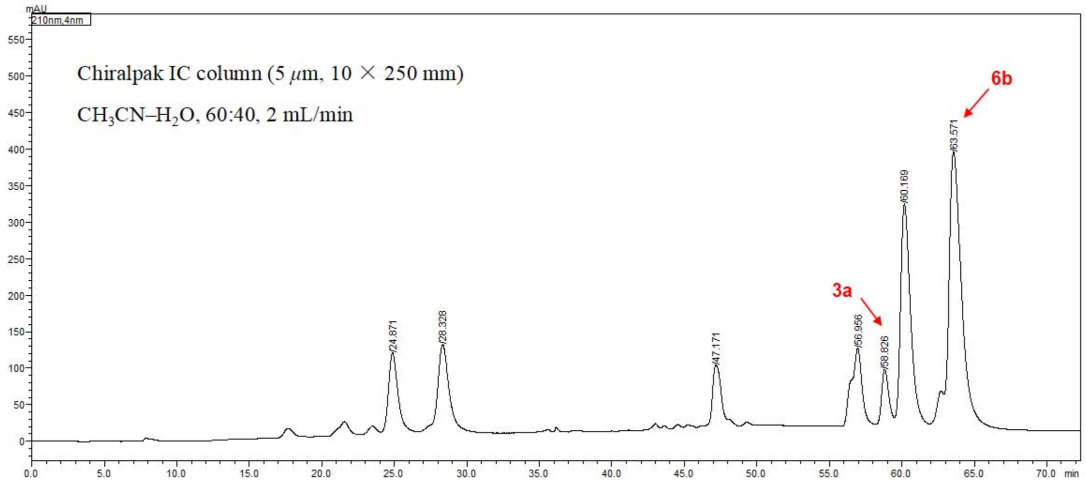

Figure S72. HPLC separation of **3a** and **6b**.

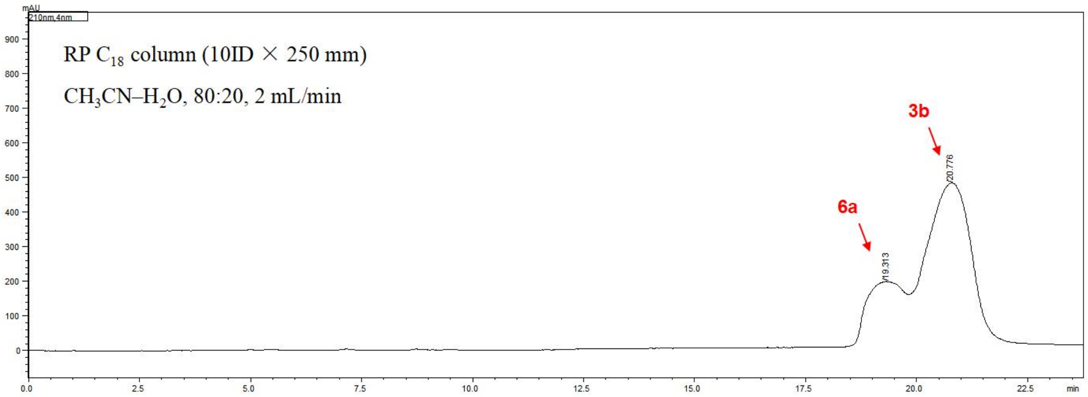

Figure S73. HPLC separation of **3b** and **6a**.

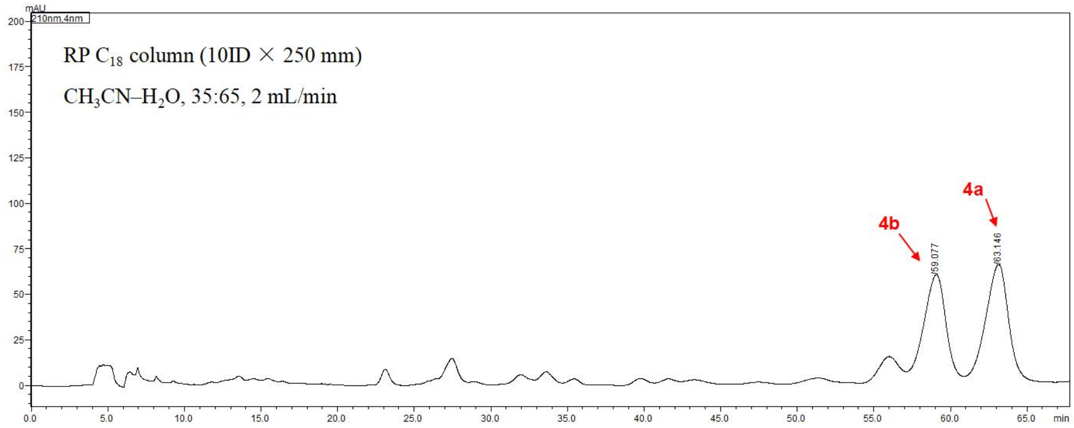

Figure S74. HPLC separation of **4a** and **4b**.

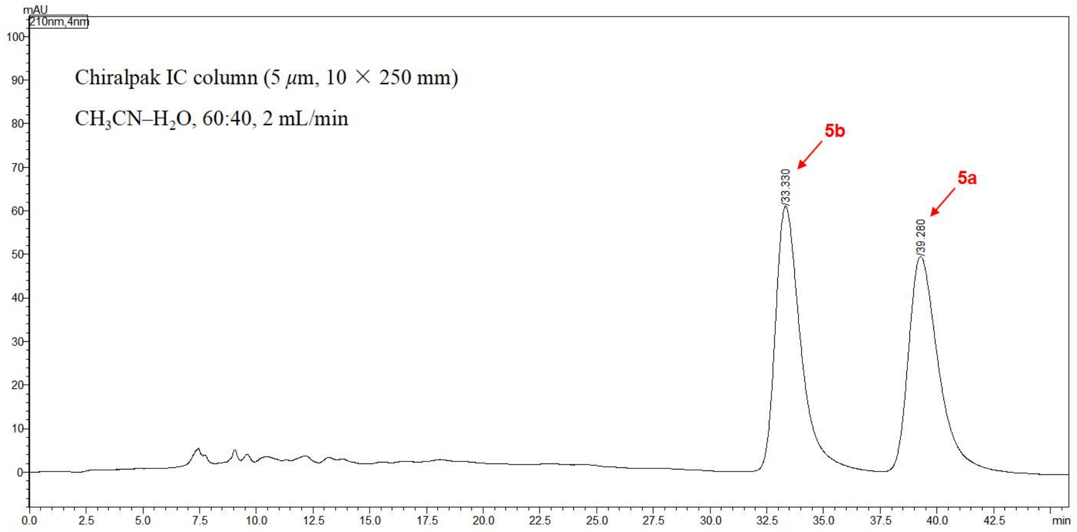

Figure S75. HPLC separation of **5a** and **5b**.

ECD calculation parts

Table S2. Conformational analysis of (2″*S*,2‴*S*)-**2**

Conformers	Gibbs Free Energy (Hartree)	Relative Gibbs Free Energy (kcal/mol)	Population (%)
1	-1571.023229	1.55559729	2.29
2	-1571.022991	1.70494467	1.78
3	-1571.022994	1.70306214	1.79
4	-1571.022983	1.70996475	1.76
5	-1571.022813	1.81664145	1.47
6	-1571.025708	0	31.63
7	-1571.023123	1.62211335	2.05
8	-1571.02285	1.79342358	1.53
9	-1571.025394	0.19703814	22.68
10	-1571.022857	1.78903101	1.54
11	-1571.022624	1.93524084	1.21
12	-1571.022666	1.90888542	1.26
13	-1571.022648	1.9201806	1.24
14	-1571.023007	1.69490451	1.81
15	-1571.022874	1.77836334	1.57
16	-1571.02507	0.40035138	16.09
17	-1571.024286	0.89231922	7.02
18	-1571.022673	1.90449285	1.27

Table S3. Coordinates of (2″*S*,2‴*S*)-**2**

	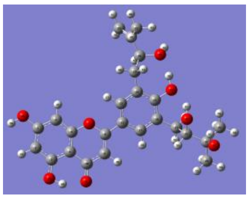 Conformer 1			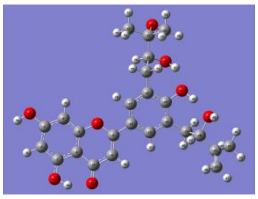 Conformer 2			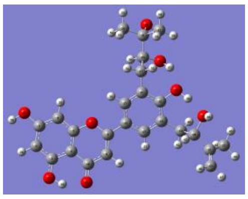 Conformer 3		
1	-6.232294	0.910646	0.190422	5.966949	1.568943	0.008131	5.966905	1.568925	0.00728
2	-6.782484	-0.377853	0.127593	6.709611	0.379596	0.037408	6.709563	0.379581	0.036989
3	-5.939336	-1.480363	0.021313	6.046485	-0.844243	0.048304	6.046424	-0.844244	0.048476
4	-4.530008	-1.301226	-0.025213	4.625773	-0.888448	0.031292	4.625709	-0.888461	0.031624
5	-4.029315	0.00979	0.040673	3.928585	0.331078	0.000548	3.928536	0.331069	0.000398
6	-4.849467	1.123093	0.147294	4.567376	1.561987	-0.011002	4.567333	1.561968	-0.011746
7	-2.681222	0.240023	-0.005937	2.5601	0.347232	-0.011331	2.560036	0.347253	-0.011248
8	-1.795215	-0.790433	-0.095808	1.845695	-0.812054	-0.014627	1.845609	-0.812042	-0.014314
9	-2.218166	-2.085956	-0.148264	2.463439	-2.027648	-0.003087	2.463358	-2.027642	-0.002427
10	-3.617905	-2.425104	-0.127471	3.898743	-2.144339	0.034092	3.898648	-2.144333	0.034738
11	-4.030769	-3.614101	-0.184341	4.490946	-3.256129	0.055376	4.49085	-3.256128	0.056602
12	-0.406684	-0.327708	-0.116866	0.402134	-0.573358	-0.044704	0.40207	-0.573332	-0.044473
13	-0.093882	1.002957	0.199213	-0.504586	-1.594834	0.276698	-0.504689	-1.594834	0.276837
14	1.218361	1.473699	0.199659	-1.8827	-1.396245	0.23931	-1.882781	-1.396232	0.239323
15	2.245834	0.576655	-0.153124	-2.361295	-0.128708	-0.152908	-2.361354	-0.128697	-0.152962
16	1.963639	-0.769485	-0.463954	-1.475238	0.923731	-0.454257	-1.4753	0.923758	-0.454167
17	0.644589	-1.200881	-0.443143	-0.107274	0.688119	-0.393333	-0.107325	0.688148	-0.393117
18	3.555517	0.947101	-0.189023	-3.691754	0.153017	-0.225505	-3.691799	0.152939	-0.225664
19	1.496374	2.899428	0.631368	-2.809254	-2.511243	0.679803	-2.809476	-2.511165	0.679684
20	2.002475	3.835092	-0.479407	-3.657038	-3.138354	-0.439641	-3.656703	-3.138599	-0.440035
21	1.826719	5.301041	-0.151723	-4.207791	-4.500202	-0.079652	-4.207656	-4.500318	-0.079814
22	2.511004	5.810376	1.089445	-5.097526	-4.578401	1.133	-5.098404	-4.577939	1.132134
23	1.092368	6.07716	-0.954016	-3.898853	-5.561102	-0.83089	-3.898015	-5.561585	-0.830247
24	3.413983	3.533462	-0.641844	-4.750332	-2.212583	-0.677615	-4.749772	-2.212882	-0.678981
25	-6.461853	-2.717163	-0.036797	6.75405	-1.98652	0.074267	6.75404	-1.986524	0.074893
26	-7.013444	2.015356	0.295973	6.568269	2.785419	-0.002872	6.568218	2.78541	-0.004261
27	3.091874	-1.708771	-0.816211	-2.022453	2.277977	-0.836121	-2.022405	2.278051	-0.836001
28	4.544863	-4.527512	-0.531786	-2.001375	5.451795	-0.593032	-2.001122	5.451745	-0.592734
29	3.794921	-2.290186	0.42078	-2.403857	3.135523	0.381079	-2.403973	3.135483	0.381278
30	4.233699	-1.270827	1.329201	-3.289305	2.449795	1.277044	-3.289688	2.449723	1.276954
31	4.998354	-3.217502	0.106585	-3.028257	4.512559	0.033462	-3.028102	4.512617	0.033776
32	6.062938	-2.519219	-0.749442	-4.274702	4.379107	-0.851076	-4.274684	4.379543	-0.850618
33	5.574557	-3.562808	1.377331	-3.407676	5.103844	1.287382	-3.407297	5.103932	1.28779
34	-7.856803	-0.529342	0.161403	7.794836	0.398097	0.051101	7.79479	0.39806	0.050562
35	-4.435853	2.122533	0.195658	4.004129	2.486415	-0.032485	4.004095	2.486396	-0.033572
36	-1.501958	-2.895332	-0.19377	1.879703	-2.937806	-0.039449	1.879582	-2.937787	-0.038588
37	-0.891094	1.683945	0.473802	-0.135807	-2.562161	0.601643	-0.135931	-2.562162	0.601808

38	0.436986	-2.231005	-0.713231	0.576926	1.491967	-0.640795	0.576893	1.491998	-0.640528
39	3.63508	1.931255	-0.321478	-4.215398	-0.682525	-0.365502	-4.21524	-0.682617	-0.366266
40	0.571465	3.32234	1.03314	-2.20465	-3.304116	1.128709	-3.493151	-2.152104	1.459655
41	2.232126	2.91445	1.445854	-3.49254	-2.152366	1.460204	-2.205048	-3.303904	1.129072
42	1.476192	3.60351	-1.412185	-3.049584	-3.217898	-1.348093	-3.048681	-3.218515	-1.348078
43	3.58035	5.572799	1.065308	-5.513028	-5.582358	1.254805	-4.549061	-4.323535	2.047539
44	2.39443	6.892853	1.1897	-4.547324	-4.324811	2.048114	-5.513711	-5.581934	1.254291
45	2.101445	5.339096	1.99199	-5.923819	-3.863482	1.051459	-5.924833	-3.863312	1.049393
46	0.614	5.682913	-1.847342	-3.258004	-5.474023	-1.704987	-3.256508	-5.474893	-1.703903
47	0.93846	7.132251	-0.742536	-4.270614	-6.554919	-0.594774	-4.269849	-6.555316	-0.593884
48	3.715464	3.892965	-1.492097	-5.151696	-2.41431	-1.53859	-5.150632	-2.41478	-1.540152
49	-5.677637	-3.338703	-0.106458	6.075036	-2.724791	0.07467	6.075105	-2.724873	0.075733
50	-7.94846	1.752438	0.319547	7.533096	2.672568	0.014609	7.533045	2.672567	0.013265
51	2.701514	-2.538779	-1.412489	-2.899917	2.144039	-1.476373	-1.272811	2.821665	-1.418561
52	3.822447	-1.17785	-1.434315	-1.272991	2.82156	-1.41888	-2.899786	2.144249	-1.476387
53	4.157375	-4.367345	-1.542301	-2.427148	6.456772	-0.681462	-2.426635	6.456872	-0.680665
54	5.392495	-5.217461	-0.599259	-1.711009	5.117478	-1.593483	-1.102602	5.513651	0.030775
55	3.764251	-5.002161	0.072574	-1.103117	5.514184	0.0308	-1.711227	5.117659	-1.593402
56	3.0704	-2.890027	0.986661	-1.497631	3.335993	0.967172	-1.497813	3.335715	0.967553
57	4.380152	-0.447355	0.827109	-3.793352	1.785897	0.770349	-3.793702	1.785954	0.770067
58	6.349364	-1.561117	-0.301621	-4.03009	4.039814	-1.862936	-4.030301	4.040161	-1.862503
59	5.715901	-2.332821	-1.771063	-4.768245	5.352823	-0.926739	-4.984951	3.670705	-0.410537
60	6.954801	-3.151232	-0.801326	-4.984817	3.670055	-0.411094	-4.767957	5.353399	-0.926248
61	5.586013	-2.717589	1.864695	-3.822851	4.367895	1.775265	-3.822775	4.368113	1.775579
	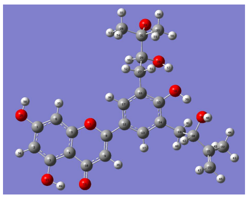 Conformer 4			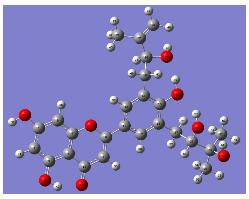 Conformer 5			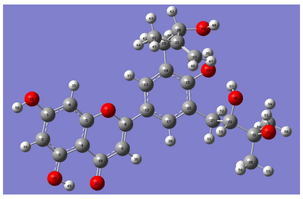 Conformer 6		
1	5.96991	1.566277	0.003725	-6.219972	0.810341	0.179636	-6.122497	0.955457	0.449167
2	6.712787	0.377153	0.036525	-6.74964	-0.486527	0.112326	-6.658537	-0.340544	0.456466
3	6.051151	-0.845138	0.050299	-5.888786	-1.575423	0.007213	-5.824615	-1.430272	0.222503
4	4.62851	-0.891035	0.033007	-4.48224	-1.374095	-0.033282	-4.438371	-1.231048	-0.020974
5	3.930886	0.326095	-0.000889	-4.002535	-0.055518	0.037429	-3.951447	0.086874	-0.018571
6	4.570285	1.559118	-0.015605	-4.840496	1.044537	0.14271	-4.762686	1.187738	0.212183
7	2.563203	0.344082	-0.013101	-2.658081	0.196042	-0.003093	-2.624725	0.335889	-0.242862
8	1.847664	-0.814926	-0.014152	-1.755624	-0.820178	-0.091489	-1.752419	-0.679828	-0.491816
9	2.465657	-2.030191	0.000231	-2.157747	-2.122066	-0.148591	-2.165507	-1.978802	-0.522274
10	3.901085	-2.146976	0.038367	-3.552129	-2.483266	-0.13439	-3.538418	-2.340247	-0.27687
11	4.492654	-3.258782	0.062632	-3.945971	-3.678432	-0.19559	-3.940694	-3.533918	-0.291838
12	0.404332	-0.575371	-0.0448	-0.374626	-0.335203	-0.105739	-0.38919	-0.195935	-0.715393
13	-0.502959	-1.596434	0.276309	-0.084158	0.998016	0.220241	-0.146656	1.165763	-0.947706
14	-1.880976	-1.397202	0.238679	1.220176	1.490153	0.226752	1.136859	1.659411	-1.171488

15	-2.358902	-0.129324	-0.153191	2.262584	0.612742	-0.130926	2.212302	0.751151	-1.145134
16	-1.472277	0.922691	-0.454314	2.003357	-0.735789	-0.45128	2.000207	-0.623424	-0.92062
17	-0.104446	0.686359	-0.393461	0.691494	-1.188837	-0.435695	0.704867	-1.075992	-0.706507
18	-3.689177	0.153255	-0.225651	3.566073	1.005245	-0.16379	3.504856	1.146274	-1.340423
19	-2.808118	-2.511939	0.678566	1.472375	2.918071	0.665908	1.328932	3.140429	-1.419732
20	-3.656398	-3.137825	-0.441218	1.969867	3.876423	-0.433792	2.087139	3.901009	-0.307406
21	-4.207589	-4.499746	-0.082221	1.71264	5.328511	-0.080367	1.625362	3.517796	1.082382
22	-5.097138	-4.578613	1.130523	0.299153	5.802102	-0.298225	0.315479	4.127693	1.505391
23	-3.899152	-5.560142	-0.834376	2.682964	6.116483	0.389036	2.308191	2.664662	1.851412
24	-4.749364	-2.211395	-0.678233	3.375388	3.599596	-0.586844	3.487816	3.648585	-0.531529
25	6.75773	-1.9887	0.079521	-6.391557	-2.820156	-0.055472	-6.334393	-2.67372	0.226599
26	6.680632	2.722364	-0.008121	-7.018621	1.902573	0.283795	-6.895473	2.048509	0.671371
27	-2.018815	2.277366	-0.835603	3.147494	-1.653663	-0.808596	3.174589	-1.572397	-0.858384
28	-1.998283	5.450835	-0.591763	4.654457	-4.445313	-0.546061	4.587361	-4.01319	0.639163
29	-2.399966	3.134328	0.382127	3.859135	-2.231732	0.425138	3.866434	-1.57614	0.513873
30	-3.284962	2.448178	1.278196	4.275973	-1.211939	1.343336	4.329804	-0.270285	0.889623
31	-3.024578	4.51148	0.035479	5.08096	-3.132908	0.10579	5.058412	-2.561531	0.635322
32	-4.271885	4.378304	-0.847848	6.134206	-2.406421	-0.740785	6.121607	-2.321812	-0.443915
33	-3.402634	5.102472	1.289964	5.660219	-3.47899	1.374892	5.641175	-2.322694	1.927302
34	7.79572	0.41396	0.050511	-7.8216	-0.655006	0.142026	-7.715156	-0.507636	0.640698
35	3.990484	2.475073	-0.040083	-4.442843	2.050262	0.19457	-4.359256	2.192493	0.209731
36	1.882122	-2.940568	-0.034175	-1.428502	-2.919835	-0.192586	-1.464648	-2.77115	-0.748741
37	-0.134745	-2.564048	0.601022	-0.893038	1.664286	0.497166	-0.981795	1.856276	-0.966877
38	0.580052	1.489947	-0.640957	0.501104	-2.220289	-0.713355	0.556277	-2.131576	-0.503735
39	-4.213377	-0.68197	-0.365772	3.628815	1.991041	-0.282652	3.601838	2.115532	-1.132466
40	-2.203928	-3.305444	1.126904	0.542021	3.313584	1.080044	1.872281	3.317468	-2.356498
41	-3.491127	-2.153172	1.459259	2.214576	2.953999	1.474335	0.345835	3.603463	-1.537635
42	-3.049186	-3.216872	-1.34987	1.450371	3.638985	-1.372458	1.911592	4.974564	-0.459861
43	-5.512749	-5.582594	1.251732	0.182598	6.848569	-0.00391	0.344368	5.222525	1.437856
44	-5.923351	-3.863533	1.049578	0.013521	5.705914	-1.354091	-0.502072	3.791991	0.853707
45	-4.546771	-4.325686	2.045722	-0.422501	5.206434	0.275398	0.06195	3.848103	2.531308
46	-4.271169	-6.554028	-0.598966	3.694702	5.748658	0.524162	1.948475	2.398101	2.841182
47	-3.258468	-5.472569	-1.708545	2.490632	7.154754	0.64491	3.220686	2.175419	1.525557
48	-5.151149	-2.412428	-1.539175	3.690703	3.999176	-1.413915	3.977094	3.869483	0.279013
49	6.079101	-2.726483	0.0815	-5.597476	-3.42919	-0.123388	-5.56129	-3.283502	0.035989
50	6.073407	3.48046	-0.02871	-7.949545	1.625207	0.301943	-7.815026	1.772852	0.821069
51	-2.896216	2.144206	-1.47609	3.869546	-1.106357	-1.422398	2.82787	-2.587558	-1.072677
52	-1.268972	2.82095	-1.417882	2.771622	-2.486013	-1.410892	3.902544	-1.310822	-1.632747
53	-1.099281	5.512813	0.03106	5.515287	-5.118375	-0.616916	5.428232	-4.671854	0.880091
54	-1.709167	5.116928	-1.592717	4.267337	-4.283177	-1.556392	3.806897	-4.164421	1.392701
55	-2.423989	6.455921	-0.679221	3.88106	-4.940264	0.051241	4.193994	-4.309825	-0.337648
56	-1.493503	3.334599	0.967949	3.145251	-2.850118	0.984449	3.132412	-1.866354	1.277112
57	-3.789504	1.784728	0.771371	4.412075	-0.382756	0.847904	4.382933	0.274967	0.081474
58	-4.98166	3.669327	-0.407224	5.786344	-2.217482	-1.761653	7.006318	-2.925392	-0.219151

59	-4.028269	4.039012	-1.859949	7.038191	-3.020666	-0.795906	6.421072	-1.268069	-0.459892
60	-4.765357	5.352093	-0.923018	6.401064	-1.447069	-0.2836	5.765336	-2.595015	-1.442602
61	-3.81833	4.366727	1.777692	5.653842	-2.638431	1.870345	5.679899	-1.350109	1.990672
	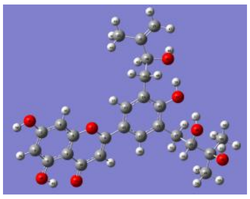 Conformer 7			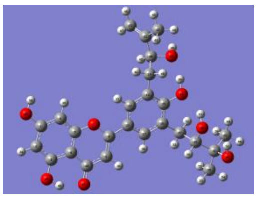 Conformer 8			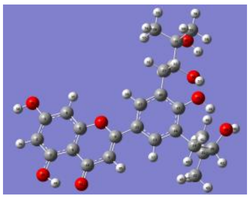 Conformer 9		
1	-6.15838	0.74695	0.818009	-6.23802	0.903947	0.154471	5.425603	2.29257	0.475457
2	-6.732524	-0.453257	0.374222	-6.7863	-0.386181	0.113871	6.374314	1.260552	0.432882
3	-5.914635	-1.460619	-0.129877	-5.942565	-1.487708	0.030578	5.961969	-0.041126	0.162607
4	-4.506856	-1.274985	-0.192761	-4.53115	-1.308409	-0.014613	4.587793	-0.322776	-0.067115
5	-3.981404	-0.055098	0.26501	-4.031923	0.00224	0.029428	3.677349	0.745911	-0.012855
6	-4.776122	0.962876	0.770431	-4.855354	1.117721	0.112862	4.064797	2.050556	0.254049
7	-2.63288	0.174861	0.231352	-2.685064	0.235628	-0.016622	2.342285	0.530116	-0.221607
8	-1.772428	-0.760182	-0.258711	-1.796505	-0.794603	-0.086299	1.866963	-0.714975	-0.501219
9	-2.222258	-1.958268	-0.730054	-2.217251	-2.091298	-0.117516	2.70181	-1.790223	-0.581455
10	-3.622005	-2.297638	-0.71852	-3.616687	-2.432542	-0.095	4.119429	-1.664457	-0.359389
11	-4.057888	-3.401348	-1.141899	-4.026965	-3.622941	-0.132079	4.906945	-2.646007	-0.420646
12	-0.379918	-0.310055	-0.21617	-0.40889	-0.329827	-0.110491	0.415684	-0.721939	-0.695449
13	-0.07074	1.025633	0.081295	-0.097025	1.000682	0.206835	-0.298418	-1.927079	-0.774866
14	1.24323	1.490496	0.116245	1.214826	1.472586	0.205636	-1.676983	-1.957794	-0.965209
15	2.2775	0.580111	-0.176441	2.242361	0.577078	-0.150932	-2.362324	-0.730052	-1.064865
16	2.000007	-0.774741	-0.451514	1.96088	-0.768689	-0.46393	-1.67405	0.495169	-0.996563
17	0.678761	-1.199433	-0.466527	0.642263	-1.201486	-0.44081	-0.296755	0.482414	-0.809891
18	3.589774	0.943953	-0.177803	3.551628	0.948908	-0.18893	-3.714904	-0.664165	-1.240742
19	1.511675	2.927494	0.51433	1.492502	2.897537	0.640064	-2.386898	-3.292926	-1.039439
20	2.065011	3.837521	-0.599494	1.995621	3.836377	-0.46938	-3.340502	-3.600874	0.138031
21	1.831063	5.305927	-0.301612	1.818997	5.301317	-0.137554	-2.740797	-3.251408	1.48327
22	0.434912	5.802483	-0.573604	2.505836	5.808325	1.103163	-1.736051	-4.243625	2.005398
23	2.806007	6.088352	0.167518	1.081755	6.078632	-0.936037	-3.048117	-2.123342	2.130172
24	3.467772	3.524547	-0.698777	3.407028	3.53647	-0.635187	-4.564845	-2.894431	-0.14065
25	-6.460414	-2.61147	-0.558598	-6.461698	-2.727401	-0.006967	6.867781	-1.032941	0.118754
26	-6.913265	1.758915	1.315811	-7.118728	1.933344	0.235763	5.780089	3.576795	0.733704
27	3.136385	-1.734147	-0.711874	3.089202	-1.705661	-0.821954	-2.434295	1.798262	-1.080998
28	4.555515	-4.545032	-0.246274	4.548905	-4.522043	-0.550879	-2.868581	4.745416	0.066907
29	3.779278	-2.266291	0.578873	3.79682	-2.289475	0.411346	-3.05368	2.215148	0.262271
30	4.183579	-1.212057	1.463167	4.236042	-1.272154	1.321713	-3.939903	1.212819	0.78326
31	4.988825	-3.212528	0.358517	5.00129	-3.213294	0.090846	-3.820155	3.563449	0.23036
32	6.097009	-2.555965	-0.474788	6.062269	-2.509827	-0.76543	-4.920345	3.579605	-0.838327
33	5.503735	-3.508899	1.66735	5.581345	-3.561789	1.358965	-4.424787	3.707868	1.5264
34	-7.805686	-0.610315	0.416751	-7.861022	-0.520645	0.148021	7.426599	1.461533	0.607328
35	-4.343854	1.893125	1.116962	-4.427186	2.113652	0.14231	3.340911	2.854895	0.290452

36	-1.52978	-2.6826	-1.137205	-1.499168	-2.899896	-0.145404	2.31411	-2.769788	-0.826649
37	-0.873902	1.719659	0.30031	-0.894408	1.680346	0.484451	0.22253	-2.873453	-0.677447
38	0.478686	-2.245498	-0.673726	0.434296	-2.230936	-0.713387	0.233332	1.426097	-0.75139
39	3.677414	1.923592	-0.327325	3.629954	1.933371	-0.319493	-4.143653	-1.505156	-0.923521
40	2.22807	2.975699	1.345137	2.229821	2.911222	1.453156	-1.634921	-4.08511	-1.08221
41	0.577248	3.355742	0.884364	0.568025	3.318722	1.044655	-2.978308	-3.373811	-1.960177
42	1.569409	3.580693	-1.545966	1.467768	3.606663	-1.40175	-3.561478	-4.676558	0.113041
43	-0.314841	5.24391	0.001296	2.099338	5.334111	2.005555	-2.16903	-5.249338	2.077251
44	0.335022	6.861149	-0.319547	3.575435	5.572277	1.075572	-1.366991	-3.95145	2.991989
45	0.174433	5.674884	-1.632628	2.388057	6.890378	1.206555	-0.872123	-4.319075	1.331809
46	2.630218	7.138615	0.383177	0.927177	7.133009	-0.721483	-3.726525	-1.377467	1.728048
47	3.805232	5.70362	0.342698	0.601788	5.686164	-1.829299	-2.594288	-1.891175	3.089481
48	3.817537	3.88715	-1.528986	3.706636	3.898402	-1.48508	-5.081893	-2.831678	0.680352
49	-5.692102	-3.173676	-0.874391	-5.676723	-3.348357	-0.06332	6.347829	-1.864603	-0.091008
50	-7.851026	1.505475	1.300824	-6.635492	2.775873	0.259419	6.741185	3.629576	0.866493
51	2.767563	-2.586604	-1.289857	2.698062	-2.534579	-1.419233	-1.754163	2.590234	-1.40796
52	3.897303	-1.233504	-1.318603	3.817202	-1.172137	-1.440855	-3.222834	1.71842	-1.835911
53	4.218283	-4.42463	-1.280062	3.770901	-5.000439	0.053895	-3.419312	5.682166	0.201824
54	3.742973	-4.989318	0.338763	5.397831	-5.209937	-0.622964	-2.41434	4.759938	-0.928264
55	5.39986	-5.242277	-0.24394	4.158418	-4.359212	-1.559817	-2.070749	4.706128	0.816426
56	3.025821	-2.838022	1.135985	3.075092	-2.89261	0.977296	-2.250151	2.320818	1.003072
57	4.360798	-0.411682	0.934413	4.380398	-0.447139	0.821522	-4.198291	0.626405	0.046624
58	6.984612	-3.195801	-0.460958	6.955526	-3.13953	-0.821323	-5.532775	4.477695	-0.712402
59	5.798409	-2.407426	-1.517682	5.712469	-2.321119	-1.785683	-5.570309	2.704068	-0.732329
60	6.371227	-1.583135	-0.051829	6.347422	-1.552392	-0.315348	-4.51021	3.584644	-1.85356
61	5.500311	-2.644462	2.119817	5.592537	-2.71819	1.849137	-4.802001	2.826893	1.708266
	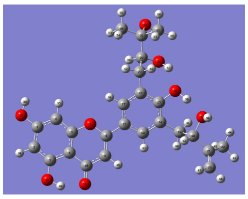 Conformer 10			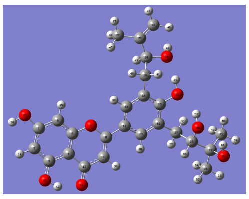 Conformer 11			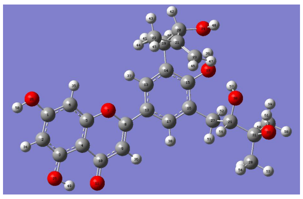 Conformer 12		
1	-6.237961	0.904032	0.155416	6.284698	0.925529	0.007118	-5.904198	1.534295	0.788434
2	-6.786185	-0.386112	0.114505	6.822208	-0.369781	0.003348	-6.693742	0.472989	0.322566
3	-5.94241	-1.48753	0.030449	5.968447	-1.466635	-0.006626	-6.080541	-0.667082	-0.183834
4	-4.531043	-1.308167	-0.015192	4.557463	-1.277709	-0.01107	-4.660694	-0.758375	-0.22816
5	-4.031861	0.002495	0.029142	4.069362	0.038066	-0.007963	-3.915362	0.330268	0.249557
6	-4.855337	1.117904	0.113222	4.903014	1.148934	0.001439	-4.505935	1.479466	0.759265
7	-2.685062	0.235977	-0.017415	2.723242	0.280952	-0.005446	-2.548351	0.296927	0.236204
8	-1.796392	-0.794233	-0.086812	1.827079	-0.744479	-0.027257	-1.877091	-0.782748	-0.252989
9	-2.217086	-2.09094	-0.117878	2.23639	-2.044694	-0.050498	-2.543127	-1.86615	-0.744612
10	-3.616517	-2.43224	-0.095581	3.63345	-2.396844	-0.029846	-3.982515	-1.92808	-0.755314
11	-4.026713	-3.622649	-0.132893	4.034051	-3.590967	-0.039348	-4.616881	-2.922226	-1.197953
12	-0.408795	-0.329425	-0.111116	0.442143	-0.271182	-0.03457	-0.424494	-0.61053	-0.185142

13	-0.096843	1.001035	0.206329	0.143305	1.061028	-0.363246	0.44465	-1.692938	-0.401501
14	1.215034	1.472833	0.20531	-1.168394	1.521548	-0.402614	1.824383	-1.535393	-0.361094
15	2.24256	0.577267	-0.151162	-2.208613	0.624635	-0.097065	2.352074	-0.257214	-0.092232
16	1.960985	-0.768447	-0.464345	-1.939559	-0.70714	0.278704	1.509321	0.840534	0.166863
17	0.642333	-1.201131	-0.441439	-0.614944	-1.133989	0.294327	0.13137	0.644452	0.107497
18	3.551859	0.948953	-0.188848	-3.482376	1.112606	-0.140798	3.711595	-0.144528	-0.056739
19	1.492763	2.897734	0.639878	-1.485122	2.952181	-0.761152	2.752965	-2.703054	-0.584884
20	1.996219	3.836569	-0.469459	-1.70484	3.855895	0.47525	3.258007	-3.342586	0.730246
21	1.819652	5.301524	-0.137726	-2.064747	5.272393	0.05857	4.226454	-4.479184	0.44734
22	2.505937	5.808413	1.103339	-3.461407	5.490285	-0.461064	5.609333	-4.092383	-0.006944
23	1.082869	6.078924	-0.936544	-1.166297	6.256577	0.155447	3.843345	-5.750094	0.599481
24	3.407656	3.53654	-0.634947	-2.681254	3.319161	1.37157	3.83871	-2.378977	1.612952
25	-6.461499	-2.727249	-0.007381	6.477494	-2.711076	-0.01262	-6.831431	-1.687464	-0.634126
26	-7.118712	1.933321	0.237518	7.174571	1.950116	0.016958	-6.567189	2.616568	1.269042
27	3.089228	-1.705652	-0.822038	-3.046047	-1.634365	0.736055	2.069396	2.18914	0.568809
28	4.54733	-4.522887	-0.549928	-4.311067	-4.570822	0.437366	1.920213	5.352887	0.011842
29	3.79618	-2.289743	0.411471	-3.897101	-2.206766	-0.398146	2.661654	2.996718	-0.586743
30	4.235613	-1.272466	1.321897	-4.561147	-1.109561	-1.039347	3.790375	2.269873	-1.089891
31	5.000328	-3.214154	0.091395	-4.957391	-3.242739	0.043337	3.115849	4.428001	-0.215448
32	6.061627	-2.511503	-0.765154	-5.874643	-2.707421	1.144844	4.08182	4.446012	0.970839
33	5.58026	-3.562483	1.359597	-5.718398	-3.420628	-1.176643	3.816258	4.834551	-1.416832
34	-7.860873	-0.52067	0.149107	7.896485	-0.51182	0.007542	-7.774474	0.54423	0.357469
35	-4.427204	2.113841	0.142817	4.483119	2.148782	0.005467	-3.89157	2.297096	1.119577
36	-1.498944	-2.899488	-0.145427	1.510209	-2.845033	-0.102926	-1.997505	-2.706343	-1.152689
37	-0.894181	1.680712	0.484019	0.947918	1.743079	-0.614339	0.046893	-2.682265	-0.602184
38	0.434264	-2.230508	-0.714211	-0.406315	-2.150974	0.610288	-0.524755	1.484346	0.30496
39	3.630385	1.933429	-0.319295	-4.105815	0.381992	-0.384684	3.973481	0.770264	-0.333086
40	2.229955	2.911329	1.453077	-0.662922	3.380414	-1.34261	2.240158	-3.482333	-1.156944
41	0.568272	3.318998	1.044344	-2.380939	2.980253	-1.390728	3.612787	-2.374083	-1.178147
42	1.468602	3.606852	-1.401949	-0.762523	3.885988	1.036431	2.386502	-3.749791	1.257857
43	2.098891	5.334264	2.005516	-4.198313	5.184784	0.290466	6.087437	-3.446374	0.738256
44	2.388309	6.890482	1.206687	-3.633859	6.54021	-0.713788	6.239131	-4.972844	-0.162139
45	3.575501	5.572169	1.076301	-3.657855	4.888333	-1.357858	5.582675	-3.524152	-0.945828
46	0.928326	7.133316	-0.722066	-1.39145	7.272436	-0.160282	2.844338	-6.005172	0.946519
47	0.603248	5.6865	-1.830004	-0.168711	6.078618	0.551356	4.509772	-6.580603	0.379253
48	3.707485	3.898423	-1.484773	-3.282543	2.746245	0.862512	4.168825	-1.634181	1.078903
49	-5.676507	-3.348106	-0.064027	5.686962	-3.327337	-0.02259	-6.182249	-2.384138	-0.947261
50	-6.635547	2.77585	0.262023	6.698033	2.796822	0.019442	-5.928355	3.288161	1.560249
51	2.698126	-2.534435	-1.419519	-2.597765	-2.463588	1.289599	1.268756	2.773282	1.030103
52	3.817642	-1.17233	-1.440618	-3.70465	-1.100302	1.430933	2.846638	2.04913	1.329359
53	4.156458	-4.360106	-1.558723	-3.673573	-4.941936	-0.371329	2.26832	6.381145	0.157813
54	3.769441	-5.000933	0.055261	-3.705828	-4.471786	1.343987	1.35029	5.064541	0.900722
55	5.395996	-5.211068	-0.622277	-5.086848	-5.31842	0.635036	1.251748	5.335722	-0.854723
56	3.074017	-2.892448	0.977307	-3.241915	-2.696086	-1.133077	1.915665	3.084055	-1.389763

57	4.380199	-0.447443	0.821822	-5.319984	-1.500442	-1.514405	4.330261	2.922713	-1.576357
58	6.347396	-1.554128	-0.315356	-6.706023	-3.402447	1.310181	3.573302	4.185045	1.9035
59	6.954502	-3.141749	-0.82091	-6.285119	-1.732731	0.865818	4.50136	5.451061	1.096262
60	5.711854	-2.322902	-1.785435	-5.340831	-2.604298	2.09402	4.903541	3.742049	0.810707
61	5.591484	-2.718794	1.84961	-6.562739	-3.840604	-0.947201	4.373534	5.597942	-1.196702
	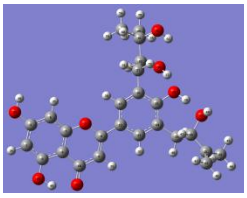 Conformer 13			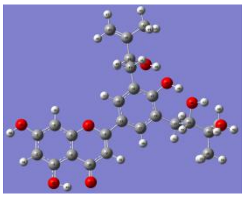 Conformer 14			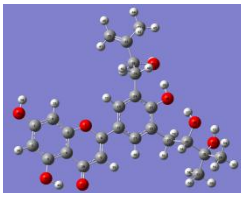 Conformer 15		
1	5.958126	1.431584	-0.627337	6.281714	0.933248	-0.059529	-6.24308	0.875637	0.138948
2	6.700685	0.318318	-0.207638	6.821007	-0.360977	-0.02657	-6.780424	-0.419395	0.10953
3	6.038213	-0.830119	0.209904	5.967534	-1.460155	0.005652	-5.927292	-1.514515	0.038763
4	4.615589	-0.877082	0.211028	4.558485	-1.272077	0.007456	-4.517295	-1.323829	-0.004907
5	3.918332	0.262706	-0.217105	4.069066	0.044573	-0.026105	-4.029234	-0.008595	0.02755
6	4.558489	1.42122	-0.638196	4.899662	1.154657	-0.059293	-4.862184	1.100631	0.098033
7	2.551179	0.273878	-0.239989	2.721598	0.283638	-0.019357	-2.684262	0.235677	-0.017661
8	1.832074	-0.811762	0.159803	1.827323	-0.74311	-0.003336	-1.787045	-0.787809	-0.072845
9	2.450586	-1.946814	0.59411	2.238833	-2.043082	0.010718	-2.196465	-2.088189	-0.091511
10	3.886286	-2.055126	0.64216	3.636358	-2.392267	0.03094	-3.593259	-2.440977	-0.070689
11	4.476189	-3.09633	1.035571	4.039271	-3.585687	0.055666	-3.993181	-3.635089	-0.096984
12	0.388463	-0.583863	0.064258	0.441548	-0.271671	-0.015222	-0.403051	-0.311542	-0.096505
13	-0.526203	-1.625126	0.284618	0.1426	1.060467	-0.344667	-0.103365	1.023451	0.213262
14	-1.902522	-1.428183	0.206756	-1.169101	1.52078	-0.387309	1.204866	1.504705	0.213986
15	-2.371129	-0.140352	-0.12701	-2.20984	0.623268	-0.086147	2.239295	0.613672	-0.133202
16	-1.481429	0.928658	-0.343287	-1.941248	-0.708944	0.288761	1.972828	-0.735505	-0.441288
17	-0.115077	0.689902	-0.245413	-0.616666	-1.135131	0.309321	0.655541	-1.176093	-0.419606
18	-3.698507	0.148298	-0.224787	-3.483971	1.109735	-0.13396	3.547544	0.991473	-0.160744
19	-2.836317	-2.57628	0.533601	-1.485211	2.951806	-0.744742	1.470383	2.933737	0.643134
20	-3.67862	-3.09902	-0.642547	-1.709662	3.853353	0.492433	1.994277	3.867242	-0.461142
21	-4.205859	-4.499278	-0.423126	-2.07078	5.269878	0.076881	1.800845	5.333419	-0.145036
22	-5.087542	-4.71713	0.77825	-3.467021	5.486556	-0.444403	2.452122	5.853576	1.109334
23	-3.883129	-5.471391	-1.281086	-1.173748	6.255122	0.176074	1.08032	6.100277	-0.968489
24	-4.788545	-2.17125	-0.779116	-2.687468	3.313627	1.385455	3.41181	3.575568	-0.589961
25	6.743721	-1.900853	0.614384	6.479774	-2.702302	0.034415	-6.435944	-2.758806	0.01211
26	6.66835	2.518515	-1.022307	7.073214	2.035023	-0.093075	-7.132442	1.898357	0.208673
27	-1.999654	2.308257	-0.680868	-3.050934	-1.637255	0.735586	3.097109	-1.679467	-0.801849
28	-3.156516	5.339572	1.500072	-4.355065	-4.583442	0.380529	5.483894	-3.92988	1.324333
29	-2.438681	3.122356	0.547511	-3.894605	-2.206859	-0.406982	3.825192	-2.266063	0.419083
30	-3.507315	2.48266	1.26171	-4.544933	-1.109237	-1.06081	4.416719	-1.242778	1.234145
31	-2.937101	4.551842	0.19974	-4.974759	-3.235993	0.018024	4.967456	-3.250871	0.04706
32	-1.986989	5.310283	-0.723086	-5.877406	-2.709215	1.131248	4.543571	-4.301469	-0.975535
33	-4.184667	4.421163	-0.498161	-5.84818	-3.38397	-1.130249	6.025092	-2.486738	-0.551189

34	7.783666	0.355956	-0.209791	7.894862	-0.519098	-0.026319	-7.854041	-0.562581	0.142772
35	3.980277	2.279456	-0.962155	4.494425	2.158387	-0.08351	-4.442505	2.100373	0.118803
36	1.870272	-2.797973	0.923848	1.514542	-2.846465	-0.009156	-1.471355	-2.890829	-0.107246
37	-0.169463	-2.619247	0.530961	0.94742	1.743209	-0.592699	-0.906918	1.69845	0.484234
38	0.573059	1.508597	-0.421116	-0.410089	-2.152908	0.62379	0.455014	-2.207717	-0.689505
39	-4.231889	-0.677459	-0.394541	-4.104979	0.378327	-0.382735	3.628721	1.978546	-0.278639
40	-2.236158	-3.407015	0.914648	-0.661218	3.381395	-1.32264	2.190221	2.955261	1.471639
41	-3.525067	-2.288759	1.338599	-2.378903	2.980445	-1.377332	0.536534	3.354776	1.025643
42	-3.075413	-3.074711	-1.55688	-0.768942	3.884026	1.056194	1.491332	3.627194	-1.404517
43	-4.53838	-4.543384	1.712412	-3.640075	6.536339	-0.697309	2.02491	5.383641	2.004377
44	-5.480415	-5.737181	0.800842	-3.661945	4.884431	-1.341418	2.325392	6.935625	1.201445
45	-5.92984	-4.016424	0.770761	-4.204586	5.180447	0.306242	3.523444	5.623796	1.111463
46	-4.236817	-6.490536	-1.147617	-0.176479	6.07807	0.573168	0.914291	7.155325	-0.766224
47	-3.248542	-5.283271	-2.143721	-1.399799	7.271009	-0.138926	0.626091	5.698213	-1.870875
48	-5.199367	-2.299653	-1.649818	-3.288136	2.742661	0.873515	3.729829	3.932669	-1.435318
49	6.064365	-2.594207	0.864542	5.689795	-3.320105	0.050757	-5.645781	-3.373735	-0.036082
50	6.059977	3.226503	-1.291693	8.006761	1.76596	-0.091081	-6.656212	2.745016	0.226583
51	-1.203057	2.85658	-1.189385	-2.607735	-2.46852	1.29076	2.678125	-2.505523	-1.381459
52	-2.84648	2.243073	-1.372441	-3.715048	-1.104759	1.426178	3.834918	-1.17499	-1.434818
53	-2.207758	5.513609	2.02101	-3.738252	-4.966636	-0.441271	4.71702	-4.577842	1.764588
54	-3.822249	4.79141	2.173236	-5.145693	-5.310557	0.588882	5.769047	-3.180736	2.068906
55	-3.609987	6.309431	1.271555	-3.720852	-4.50461	1.268671	6.360961	-4.540951	1.088077
56	-1.582206	3.208225	1.2315	-3.227558	-2.698498	-1.132152	3.090457	-2.801216	1.037302
57	-3.778997	1.681277	0.775195	-5.329233	-1.495437	-1.499787	4.307348	-0.380965	0.789125
58	-2.324177	6.346008	-0.835268	-5.33344	-2.635889	2.076644	4.336847	-3.846859	-1.948041
59	-0.972795	5.325762	-0.30924	-6.719344	-3.393268	1.269933	5.344491	-5.036118	-1.109036
60	-1.95365	4.856626	-1.717172	-6.269532	-1.720271	0.876019	3.647216	-4.833069	-0.637902
61	-4.682577	3.779627	0.042613	-5.368918	-3.895466	-1.804999	6.131898	-1.729962	0.055113
	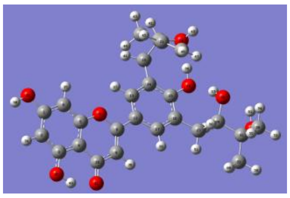 Conformer 16			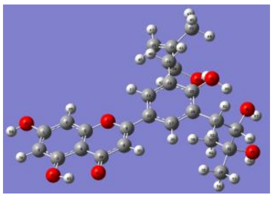 Conformer 17			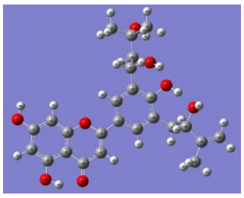 Conformer 18		
1	-6.131322	0.952491	0.457033	5.745887	1.759382	0.660945	5.928017	1.649091	-0.021787
2	-6.661888	-0.345775	0.464423	6.540739	0.61041	0.537359	6.68481	0.468853	0.013063
3	-5.823699	-1.431872	0.22884	5.95282	-0.594434	0.162649	6.037447	-0.760925	0.035945
4	-4.438582	-1.226634	-0.016315	4.5558	-0.660208	-0.091586	4.615347	-0.823311	0.025783
5	-3.957163	0.093357	-0.013629	3.80428	0.518921	0.045844	3.903477	0.385502	-0.010413
6	-4.772722	1.190632	0.218697	4.367739	1.730268	0.417607	4.528482	1.625731	-0.034034
7	-2.63169	0.348029	-0.239338	2.455042	0.511459	-0.182393	2.535614	0.387562	-0.016224
8	-1.755697	-0.663928	-0.490023	1.813672	-0.627768	-0.562951	1.8336	-0.779625	-0.00849
9	-2.163215	-1.964488	-0.52105	2.489184	-1.800748	-0.72704	2.465737	-1.987564	0.008679
10	-3.534451	-2.331862	-0.274065	3.907306	-1.894993	-0.491321	3.902579	-2.08752	0.040431

11	-3.931478	-3.527238	-0.289067	4.549508	-2.970005	-0.629072	4.507063	-3.192327	0.067001
12	-0.394367	-0.1742	-0.71465	0.379228	-0.413372	-0.763577	0.38742	-0.557092	-0.033773
13	-0.157142	1.188986	-0.94308	-0.150388	0.88588	-0.812132	-0.50657	-1.58802	0.292671
14	1.124823	1.686382	-1.166871	-1.508008	1.109482	-1.010799	-1.887026	-1.405518	0.259613
15	2.201663	0.780138	-1.147508	-2.366391	0.00343	-1.154344	-2.381305	-0.144411	-0.133352
16	1.998201	-0.595214	-0.923667	-1.867679	-1.314748	-1.111023	-1.508536	0.917752	-0.439395
17	0.702567	-1.049948	-0.708645	-0.500529	-1.495304	-0.916838	-0.137806	0.697836	-0.382904
18	3.493886	1.180202	-1.342881	-3.690364	0.277256	-1.329922	-3.715118	0.122126	-0.203261
19	1.314016	3.17039	-1.399256	-2.065069	2.513173	-1.018054	-2.7978	-2.532317	0.702177
20	2.090146	3.91535	-0.288008	-2.630172	2.943838	0.355585	-3.651387	-3.173496	-0.409301
21	1.665087	3.496447	1.10317	-3.122497	4.380404	0.322144	-4.133267	-4.559781	-0.027203
22	0.346186	4.062133	1.558509	-4.38696	4.646334	-0.45143	-3.110077	-5.657057	-0.164116
23	2.38436	2.651064	1.847169	-2.445061	5.341843	0.956169	-5.380861	-4.773515	0.397859
24	3.487661	3.683219	-0.550773	-3.670046	2.068571	0.807246	-4.746463	-2.265184	-0.633889
25	-6.32815	-2.677431	0.232978	6.710631	-1.697582	0.040854	6.757335	-1.89608	0.067191
26	-6.908573	2.042174	0.680513	6.272821	2.956447	1.02259	6.625064	2.813324	-0.042886
27	3.164698	-1.556643	-0.861558	-2.753286	-2.53237	-1.285084	-2.07271	2.265016	-0.821203
28	5.538526	-2.640179	2.052213	-2.511479	-3.909712	1.574702	-2.092655	5.438917	-0.584973
29	3.880925	-1.556666	0.500099	-3.856979	-2.798555	-0.254012	-2.459551	3.119745	0.396373
30	4.467451	-0.276243	0.786659	-4.929133	-1.86902	-0.497235	-3.331236	2.424089	1.298121
31	5.0288	-2.597081	0.603775	-3.492064	-2.783125	1.248285	-3.103624	4.487857	0.049515
32	4.614699	-3.993445	0.147588	-2.987521	-1.440322	1.787375	-4.353408	4.336583	-0.827437
33	6.087732	-2.168857	-0.265438	-4.786052	-3.06597	1.839891	-3.483184	5.076847	1.304471
34	-7.717574	-0.517411	0.649926	7.608713	0.645847	0.728363	7.767298	0.518284	0.02148
35	-4.373592	2.197106	0.216219	3.763753	2.623354	0.517001	3.938207	2.534959	-0.060024
36	-1.459418	-2.753826	-0.748887	1.970229	-2.691894	-1.053595	1.892772	-2.904875	-0.018475
37	-0.994113	1.877405	-0.957435	0.509643	1.737644	-0.694469	-0.125814	-2.550921	0.617156
38	0.556897	-2.10625	-0.507724	-0.123504	-2.511265	-0.868581	0.536117	1.509137	-0.634223
39	3.59223	2.1512	-1.13942	-4.251231	-0.510797	-1.08013	-4.229392	-0.719387	-0.332756
40	1.842761	3.360828	-2.341657	-1.273786	3.219609	-1.287107	-2.179599	-3.308218	1.159618
41	0.329238	3.634736	-1.495064	-2.850751	2.601875	-1.775692	-3.49522	-2.189825	1.477953
42	1.900006	4.989921	-0.412591	-1.81413	2.872211	1.086548	-3.04632	-3.243915	-1.323806
43	0.34071	5.157895	1.501682	-4.690281	5.694107	-0.372646	-3.522796	-6.622796	0.139676
44	-0.473557	3.707551	0.919864	-5.200469	4.014931	-0.077025	-2.76325	-5.740365	-1.202567
45	0.122131	3.765007	2.586335	-4.266257	4.407296	-1.516043	-2.219668	-5.462137	0.447304
46	2.048886	2.359381	2.838303	-1.542249	5.122654	1.522124	-5.718754	-5.768496	0.674101
47	3.30616	2.194186	1.500928	-2.763706	6.381147	0.929739	-6.09858	-3.963565	0.475388
48	3.996373	3.909434	0.246362	-3.966119	1.539844	0.042741	-5.182569	-2.490915	-1.471677
49	-5.552755	-3.283866	0.041045	6.081798	-2.428302	-0.235788	6.087321	-2.641683	0.075933
50	-7.82696	1.76272	0.830417	7.229147	2.862503	1.166028	6.00871	3.563969	-0.064456
51	2.78572	-2.563117	-1.053746	-3.259549	-2.484806	-2.258566	-2.950815	2.120041	-1.458143
52	3.900888	-1.333822	-1.641218	-2.113542	-3.418248	-1.321092	-1.331729	2.816474	-1.407402
53	6.418697	-3.288163	2.113587	-2.888785	-4.871006	1.211603	-1.191635	5.514348	0.033444
54	5.816959	-1.638048	2.391441	-2.373514	-3.98098	2.658888	-1.80386	5.106158	-1.586405

55	4.770596	-3.031734	2.729475	-1.531338	-3.722383	1.124273	-2.531939	6.438097	-0.67305
56	3.140376	-1.773478	1.282907	-4.244934	-3.8056	-0.457678	-1.553233	3.333168	0.977718
57	4.311619	0.30537	0.016458	-5.47591	-1.888372	0.312923	-3.83139	1.754856	0.794686
58	5.417388	-4.70753	0.359014	-1.96368	-1.237491	1.470486	-5.051821	3.619359	-0.382033
59	4.417185	-4.016528	-0.927401	-2.99088	-1.474928	2.883839	-4.110337	3.998486	-1.840061
60	3.715552	-4.325538	0.677922	-3.617371	-0.604447	1.473002	-4.859878	5.303713	-0.902119
61	6.186169	-1.220872	-0.057097	-4.736005	-2.847983	2.784344	-3.886417	4.33682	1.796175

Table S4. Conformational analysis of (2″*S*,2‴*R*)-**2**

Conformers	Gibbs Free Energy (Hartree)	Relative Gibbs Free Energy (kcal/mol)	Population (%)
1	-1571.022908	1.43888043	1.48
2	-1571.02303	1.36232421	1.68
3	-1571.023628	0.98707323	3.17
4	-1571.023295	1.19603406	2.23
5	-1571.025201	0	16.76
6	-1571.022971	1.3993473	1.58
7	-1571.022895	1.44703806	1.46
8	-1571.024844	0.22402107	11.48
9	-1571.023923	0.258320938	4.33
10	-1571.022883	1.45456818	1.44
11	-1571.024454	0.46874997	7.60
12	-1571.023139	1.29392562	1.89
13	-1571.024051	0.7216365	4.96
14	-1571.024122	0.67708329	5.34
15	-1571.023727	0.92494974	3.52
16	-1571.023216	1.24560735	2.05
17	-1571.023219	1.24372482	2.05
18	-1571.024715	0.30496986	10.02
19	-1571.023153	1.28514048	1.92
20	-1571.024654	0.34324797	9.39
21	-1571.023467	1.08810234	2.67
22	-1571.02358	1.01719371	3.01

Table S5. Coordinates of (2″*S*,2‴*R*)-**2**

	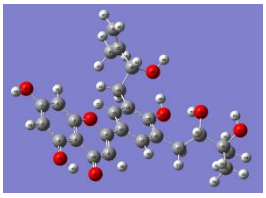 Conformer 1	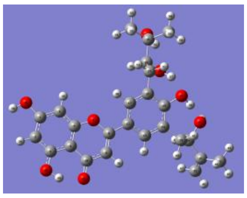 Conformer 2	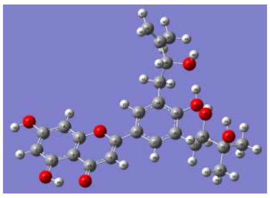 Conformer 3

1	-6.123441	1.060825	0.614529	5.891265	1.592405	0.456436	-6.257795	1.005784	-0.131615
2	-6.741304	-0.188477	0.458552	6.665744	0.425342	0.383614	-6.803414	-0.253359	0.157855
3	-5.971732	-1.298032	0.120571	6.044691	-0.797043	0.143004	-5.960543	-1.353536	0.288183
4	-4.568471	-1.16602	-0.063913	4.634886	-0.862251	-0.026844	-4.556272	-1.201963	0.127574
5	-3.99826	0.107184	0.103318	3.90506	0.335629	0.0535	-4.060114	0.080601	-0.160367
6	-4.74463	1.226707	0.439418	4.501525	1.564626	0.292093	-4.880002	1.190988	-0.294524
7	-2.651431	0.290035	-0.056906	2.545013	0.330153	-0.097657	-2.717276	0.282504	-0.329887
8	-1.838404	-0.747595	-0.398682	1.871084	-0.826964	-0.345064	-1.831893	-0.744705	-0.206017
9	-2.333107	-2.003794	-0.589515	2.523413	-2.019822	-0.448366	-2.249247	-2.009291	0.087152
10	-3.733697	-2.297117	-0.423413	3.951551	-2.116003	-0.285002	-3.644441	-2.32264	0.262369
11	-4.211069	-3.451471	-0.586952	4.573827	-3.20818	-0.368785	-4.052848	-3.485225	0.524462
12	-0.440013	-0.335635	-0.532092	0.430317	-0.607238	-0.485309	-0.447754	-0.312774	-0.408042
13	-0.101581	1.023344	-0.608936	-0.471305	-1.68156	-0.464959	-0.122731	1.051344	-0.434249
14	1.218474	1.448889	-0.750956	-1.84451	-1.496598	-0.609594	1.187265	1.495908	-0.608548
15	2.232054	0.470874	-0.785917	-2.326504	-0.178794	-0.753375	2.204569	0.530095	-0.739648
16	1.920379	-0.902977	-0.736733	-1.443645	0.917015	-0.804525	1.903011	-0.846907	-0.759096
17	0.592211	-1.285244	-0.605858	-0.080457	0.688709	-0.662718	0.584611	-1.247635	-0.590954
18	3.552817	0.796563	-0.89545	-3.656946	0.098318	-0.877188	3.51747	0.871025	-0.888083
19	1.508472	2.928705	-0.902768	-2.756388	-2.706907	-0.638915	1.460275	2.984828	-0.684523
20	2.231803	3.576842	0.289957	-3.689466	-2.854456	0.575106	2.241545	3.567567	0.505566
21	2.099276	5.083021	0.323009	-4.22188	-4.258846	0.752344	2.092553	5.066242	0.642641
22	2.626312	5.841491	-0.86657	-5.019446	-4.835897	-0.387457	2.541097	5.906753	-0.523778
23	1.536054	5.676816	1.379165	-3.977353	-4.923345	1.88542	1.583892	5.582857	1.764988
24	3.630007	3.207844	0.154322	-4.790853	-1.936278	0.343084	3.634313	3.230487	0.268272
25	-6.559789	-2.496796	-0.031819	6.782717	-1.917885	0.071074	-6.478423	-2.561832	0.56722
26	-6.831816	2.170705	0.943074	6.450172	2.806669	0.690229	-7.038675	2.107091	-0.268612
27	3.02487	-1.932841	-0.789134	-1.982949	2.316799	-0.986103	3.009018	-1.860424	-0.939415
28	4.304013	-4.636657	0.322876	-1.944767	5.398432	-0.161473	4.392985	-4.607886	-0.099544
29	3.672816	-2.185419	0.580543	-2.447145	2.953598	0.332546	3.726831	-2.198613	0.376255
30	4.161236	-0.976161	1.176646	-3.412071	2.139846	1.013072	4.219375	-1.025831	1.038327
31	4.825226	-3.223864	0.570768	-3.029201	4.384579	0.192203	4.897026	-3.207273	0.236922
32	5.932461	-2.854108	-0.424387	-4.194249	4.442732	-0.803896	5.953161	-2.736338	-0.771044
33	5.372592	-3.220852	1.899941	-3.51227	4.745791	1.497095	5.500712	-3.294924	1.538533
34	-7.811642	-0.303867	0.596675	7.743097	0.459761	0.511776	-7.873846	-0.383525	0.282746
35	-4.278162	2.195913	0.563229	3.913609	2.471896	0.351135	-4.47014	2.167718	-0.518788
36	-1.67764	-2.811067	-0.887557	1.976351	-2.925676	-0.673014	-1.530743	-2.808685	0.209378
37	-0.888402	1.76825	-0.579478	-0.105415	-2.693723	-0.329056	-0.911037	1.786593	-0.321783
38	0.367279	-2.344722	-0.539036	0.599423	1.532504	-0.695777	0.363226	-2.309551	-0.617813
39	3.696555	1.727621	-0.569666	-4.194896	-0.645969	-0.489259	3.670809	1.787298	-0.526628
40	2.109753	3.111126	-1.802723	-2.133465	-3.60373	-0.697046	2.012412	3.227111	-1.601746
41	0.558737	3.451294	-1.04639	-3.37838	-2.694847	-1.543176	0.500918	3.505768	-0.747018
42	1.837849	3.154371	1.220915	-3.152616	-2.549562	1.480173	1.906548	3.080707	1.428174
43	2.062878	5.597706	-1.776287	-5.844798	-4.167778	-0.6575	2.471218	6.973197	-0.293069
44	3.67162	5.576467	-1.060441	-4.401078	-4.956128	-1.286173	3.577301	5.672362	-0.791837

45	2.562061	6.921285	-0.707464	-5.431439	-5.81444	-0.126471	1.930619	5.710703	-1.414481
46	1.169023	5.103811	2.227244	-4.337791	-5.936947	2.041304	1.273436	4.951456	2.593976
47	1.416366	6.756032	1.430154	-3.40271	-4.479094	2.694502	1.45421	6.654629	1.891903
48	4.073994	3.338684	1.007917	-5.252177	-1.780571	1.183321	4.12599	3.322792	1.100542
49	-5.821448	-3.131414	-0.272877	6.131525	-2.659184	-0.108758	-5.695535	-3.186945	0.615302
50	-7.771047	1.941413	1.039296	7.411624	2.709838	0.790485	-7.970336	1.863931	-0.139331
51	3.791053	-1.610952	-1.501202	-2.816099	2.298796	-1.695574	2.589507	-2.782785	-1.352003
52	2.617971	-2.878423	-1.159501	-1.202853	2.949074	-1.42037	3.73585	-1.483407	-1.665457
53	5.113083	-5.359359	0.471735	-2.354617	6.412152	-0.102889	3.62029	-4.921498	0.610738
54	3.930372	-4.752274	-0.698928	-1.105588	5.327261	0.538971	3.978046	-4.651722	-1.11095
55	3.495817	-4.878447	1.021618	-1.568035	5.243629	-1.176924	5.221491	-5.321563	-0.041441
56	2.905757	-2.566992	1.267485	-1.586339	3.024528	1.010675	3.000798	-2.646331	1.0677
57	4.295742	-0.321366	0.465042	-3.82342	1.546056	0.356092	4.321555	-0.322111	0.3692
58	5.603767	-2.951958	-1.464243	-4.943707	3.680845	-0.562642	6.829051	-3.389108	-0.706457
59	6.265181	-1.822817	-0.26264	-3.863852	4.287803	-1.836226	6.271789	-1.713087	-0.543389
60	6.789491	-3.517403	-0.272649	-4.673058	5.424775	-0.74206	5.582711	-2.761678	-1.801126
61	5.434926	-2.273345	2.123931	-3.981632	3.946389	1.801108	5.554879	-2.366381	1.832965
	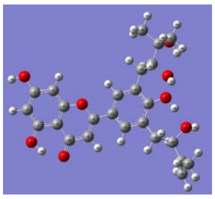 Conformer 4			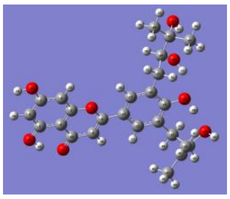 Conformer 5			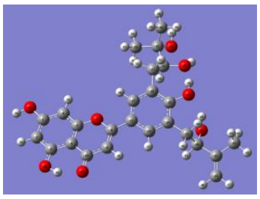 Conformer 6		
1	6.03516	1.488801	-0.352182	-5.517668	2.079139	0.95517	5.838236	1.637873	0.489651
2	6.733433	0.349872	0.074519	-6.439491	1.180191	0.399075	6.637972	0.489253	0.39893
3	6.034251	-0.82243	0.347467	-5.97977	0.026431	-0.229698	6.04395	-0.742058	0.136295
4	4.621718	-0.865406	0.193345	-4.585514	-0.23898	-0.307121	4.636187	-0.834915	-0.037361
5	3.969515	0.303016	-0.235305	-3.703794	0.693676	0.264183	3.880302	0.345102	0.062612
6	4.644757	1.482045	-0.513097	-4.138217	1.849507	0.895592	4.449589	1.582565	0.323119
7	2.611524	0.316273	-0.405314	-2.352025	0.48587	0.221689	2.521012	0.312652	-0.090983
8	1.861759	-0.790006	-0.144192	-1.828947	-0.615811	-0.384411	1.872548	-0.853977	-0.361551
9	2.432767	-1.949132	0.291092	-2.631991	-1.551377	-0.967567	2.5512	-2.030422	-0.484288
10	3.856782	-2.06574	0.476098	-4.066788	-1.427349	-0.958343	3.980599	-2.098449	-0.319104
11	4.407343	-3.129153	0.867316	-4.827055	-2.283421	-1.484438	4.626822	-3.175254	-0.420128
12	0.434512	-0.55859	-0.371768	-0.366527	-0.63663	-0.325613	0.427855	-0.664728	-0.500994
13	-0.462364	-1.631843	-0.479648	0.356948	-1.782538	-0.689592	-0.450318	-1.760419	-0.527339
14	-1.82687	-1.435762	-0.679595	1.748177	-1.826114	-0.647276	-1.8213	-1.587661	-0.662205
15	-2.30516	-0.110705	-0.750578	2.434846	-0.674931	-0.20836	-2.337575	-0.278958	-0.763418
16	-1.423288	0.98529	-0.689124	1.737204	0.492799	0.156597	-1.48148	0.840581	-0.776425
17	-0.067594	0.746456	-0.499466	0.34937	0.495369	0.095952	-0.112423	0.623125	-0.629235
18	-3.629444	0.173844	-0.917124	3.792222	-0.614888	-0.123858	-3.692787	-0.169959	-0.873668
19	-2.732064	-2.637753	-0.859389	2.460198	-3.08361	-1.103132	-2.754198	-2.775344	-0.660857
20	-3.713803	-2.891541	0.297269	3.319945	-3.806755	-0.042737	-3.352549	-3.077294	0.732514
21	-4.258093	-4.302313	0.320617	2.647587	-3.873157	1.311716	-4.290208	-4.271748	0.678997

22	-5.01489	-4.76283	-0.897274	1.481973	-4.822623	1.393921	-5.626696	-4.054172	0.019683
23	-4.05757	-5.073137	1.393404	3.041799	-3.11935	2.341621	-3.920882	-5.446585	1.197293
24	-4.800617	-1.94654	0.109516	4.587478	-3.123418	-0.020419	-4.011203	-1.937673	1.291398
25	6.699025	-1.915671	0.758576	-6.858893	-0.837876	-0.764801	6.806216	-1.845228	0.046403
26	6.673106	2.653702	-0.630488	-5.917119	3.216191	1.579088	6.370407	2.86014	0.743657
27	-1.957624	2.394681	-0.795195	2.502888	1.716087	0.600695	-1.985574	2.25668	-0.97816
28	-1.969191	5.411667	0.242464	2.985941	4.862259	0.512667	-1.617455	4.929006	0.746702
29	-2.479337	2.937065	0.543961	3.014532	2.562534	-0.575529	-2.859613	2.795459	0.158054
30	-3.467701	2.075673	1.125304	3.779112	1.790797	-1.511903	-4.183279	2.281324	-0.062665
31	-3.061967	4.373312	0.480376	3.850805	3.803221	-0.165325	-2.941656	4.332677	0.265196
32	-4.186539	4.498768	-0.555352	5.058269	3.428584	0.703812	-3.419166	4.976962	-1.036846
33	-3.598819	4.641156	1.786777	4.321934	4.387307	-1.39134	-3.942488	4.500825	1.296931
34	7.811814	0.36863	0.196645	-7.506989	1.370112	0.450388	7.714168	0.544803	0.5296
35	4.116075	2.367161	-0.843737	-3.435891	2.550419	1.328877	3.842038	2.475774	0.396293
36	1.817367	-2.807815	0.524549	-2.202324	-2.410369	-1.465135	2.024452	-2.944121	-0.724912
37	-0.092805	-2.651214	-0.442392	-0.169457	-2.673322	-1.015041	-0.068713	-2.770694	-0.421437
38	0.610848	1.589848	-0.436569	-0.187142	1.391777	0.385051	0.5529	1.478753	-0.632985
39	-4.180228	-0.594909	-0.602001	4.201505	-1.522212	-0.13411	-3.996615	0.742145	-0.616388
40	-2.104941	-3.526524	-0.971429	1.709756	-3.789504	-1.467999	-3.567379	-2.608012	-1.374785
41	-3.315844	-2.543274	-1.783936	3.12155	-2.86825	-1.95214	-2.213185	-3.667676	-0.990559
42	-3.212079	-2.677318	1.24732	3.497086	-4.829297	-0.403008	-2.519368	-3.318481	1.405491
43	-4.364437	-4.799692	-1.780476	0.66421	-4.497799	0.737707	-5.515695	-3.774482	-1.036083
44	-5.438384	-5.759494	-0.746585	1.766813	-5.83177	1.070719	-6.161097	-3.231825	0.50844
45	-5.828335	-4.067157	-1.131466	1.091039	-4.880834	2.413064	-6.246687	-4.953794	0.067613
46	-4.42658	-6.094596	1.438929	2.541784	-3.18005	3.303839	-2.956547	-5.575147	1.68415
47	-3.511768	-4.711488	2.261521	3.856705	-2.406258	2.264068	-4.564112	-6.322078	1.150803
48	-5.29161	-1.861744	0.942981	5.09096	-3.425475	0.754679	-4.216174	-1.32544	0.560374
49	6.000021	-2.622151	0.893681	-6.306064	-1.577512	-1.156576	6.171345	-2.597241	-0.147251
50	7.627739	2.545127	-0.486113	-6.88691	3.272156	1.565729	7.333975	2.782947	0.840517
51	-1.161264	3.055527	-1.150175	3.34881	1.406613	1.222274	-2.566582	2.3272	-1.906328
52	-2.759608	2.427224	-1.539163	1.855532	2.343562	1.220341	-1.115649	2.905417	-1.101978
53	-1.158328	5.293309	0.969369	3.567544	5.779538	0.652082	-0.823699	4.817223	0.001516
54	-2.38608	6.417896	0.355325	2.112811	5.100812	-0.104295	-1.742508	5.999797	0.940379
55	-1.551341	5.329358	-0.765316	2.640089	4.527689	1.495248	-1.295709	4.447762	1.675863
56	-1.647939	2.961776	1.260868	2.150878	2.934244	-1.142148	-2.485835	2.421795	1.121316
57	-3.852063	1.531955	0.411221	4.157983	1.022251	-1.045473	-4.750047	2.682925	0.621371
58	-4.941617	3.720622	-0.398115	5.645912	2.637445	0.224993	-2.675274	4.863301	-1.831479
59	-4.671927	5.473223	-0.444401	4.760593	3.083051	1.699249	-4.356597	4.521453	-1.366591
60	-3.814537	4.416852	-1.581905	5.70145	4.305647	0.824328	-3.581883	6.050992	-0.888907
61	-4.077273	3.821916	2.014179	4.615889	3.619414	-1.916657	-4.261198	5.416766	1.260807

	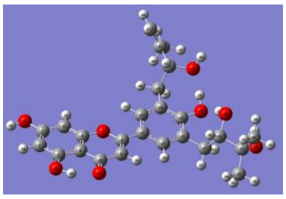 Conformer 7			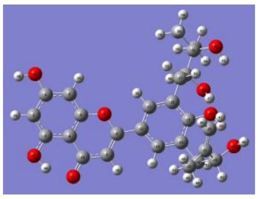 Conformer 8			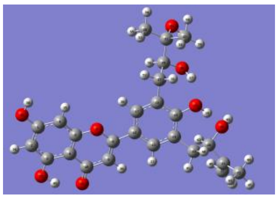 Conformer 9		
1	-6.264733	0.964425	-0.157451	-5.510999	2.060085	0.950148	6.031637	1.490647	-0.319929
2	-6.79788	-0.296969	0.145226	-6.433575	1.160553	0.39624	6.732501	0.346543	0.088503
3	-5.943502	-1.385663	0.295259	-5.974788	0.005929	-0.231595	6.037385	-0.830452	0.340854
4	-4.540004	-1.220238	0.141152	-4.580606	-0.259806	-0.310263	4.623164	-0.875983	0.184758
5	-4.05669	0.064027	-0.16105	-3.698105	0.673586	0.258787	3.968238	0.295202	-0.225582
6	-4.88816	1.163148	-0.314649	-4.131616	1.830244	0.889196	4.641602	1.482196	-0.48392
7	-2.71518	0.278922	-0.325686	-2.346334	0.465523	0.215049	2.611377	0.309848	-0.39724
8	-1.819503	-0.736762	-0.183857	-1.824453	-0.636936	-0.390334	1.862141	-0.801109	-0.153333
9	-2.223738	-2.001714	0.124599	-2.627885	-1.573073	-0.971569	2.435733	-1.965462	0.264027
10	-3.616508	-2.32857	0.296345	-4.062886	-1.448982	-0.960656	3.860072	-2.082223	0.447931
11	-4.012989	-3.492078	0.571753	-4.823603	-2.305675	-1.484847	4.412058	-3.150695	0.822341
12	-0.438929	-0.292486	-0.385908	-0.361608	-0.658039	-0.334033	0.434879	-0.566688	-0.377742
13	-0.126833	1.074364	-0.418288	0.36007	-1.807916	-0.688545	-0.465443	-1.637486	-0.482237
14	1.179739	1.528049	-0.593006	1.751345	-1.850214	-0.650088	-1.829908	-1.437827	-0.678827
15	2.204261	0.569487	-0.717655	2.437569	-0.693902	-0.224918	-2.304741	-0.111529	-0.750816
16	1.918353	-0.810106	-0.734796	1.744501	0.477644	0.133425	-1.419583	0.981924	-0.693075
17	0.601052	-1.218623	-0.565935	0.355311	0.476902	0.076102	-0.064248	0.739532	-0.505613
18	3.516191	0.922993	-0.858026	3.796174	-0.628264	-0.153309	-3.628528	0.176556	-0.914652
19	1.442823	3.018388	-0.675448	2.462063	-3.11103	-1.099374	-2.739075	-2.637605	-0.853322
20	2.20671	3.613383	0.519772	3.347511	-3.813168	-0.046535	-3.718506	-2.885572	0.306581
21	2.053422	5.112951	0.641086	2.704982	-3.861038	1.323035	-4.266923	-4.294633	0.335075
22	2.515699	5.943596	-0.527034	1.536819	-4.803689	1.440734	-5.028137	-4.756194	-0.879674
23	1.529219	5.638694	1.751968	3.125406	-3.099053	2.336384	-4.066224	-5.063072	1.409543
24	3.60325	3.276675	0.304706	4.612615	-3.124823	-0.063342	-4.803175	-1.937943	0.119317
25	-6.449487	-2.595905	0.587251	-6.854707	-0.858796	-0.764622	6.703268	-1.930011	0.734979
26	-7.057091	2.05486	-0.313405	-5.90954	3.197931	1.573075	6.772849	2.604528	-0.547583
27	3.018417	-1.831278	-0.924901	2.495196	1.713232	0.57594	-1.94977	2.392748	-0.800769
28	5.417415	-3.653745	1.566347	4.060425	4.726188	-1.361411	-1.947104	5.411538	0.231553
29	3.770674	-2.17245	0.371667	3.018278	2.567947	-0.590281	-2.46652	2.939581	0.53847
30	4.420358	-1.017453	0.923141	3.930624	1.837181	-1.424139	-3.456928	2.083	1.12331
31	4.873127	-3.250421	0.18769	3.768383	3.849495	-0.134407	-3.043598	4.378014	0.473477
32	4.386425	-4.482583	-0.56996	3.004187	4.649939	0.916642	-4.169702	4.506193	-0.560242
33	5.927079	-2.666667	-0.592258	5.005251	3.439589	0.467813	-3.576692	4.65043	1.780442
34	-7.867599	-0.437558	0.265061	-7.500986	1.350691	0.448576	7.809012	0.383067	0.207146
35	-4.488004	2.141395	-0.549536	-3.428716	2.531538	1.320931	4.094283	2.362895	-0.801185
36	-1.49665	-2.790924	0.261544	-2.198646	-2.43227	-1.469172	1.822734	-2.82959	0.483251
37	-0.921485	1.80337	-0.309949	-0.167381	-2.701804	-1.003691	-0.099281	-2.657999	-0.443823
38	0.386627	-2.281941	-0.592684	-0.181414	1.374787	0.360197	0.616307	1.581345	-0.445239

39	3.660931	1.838146	-0.487757	4.214813	-1.532193	-0.174917	-4.180763	-0.589945	-0.596645
40	0.480595	3.532183	-0.751303	1.70828	-3.826617	-1.43744	-3.324997	-2.543799	-1.776588
41	2.003083	3.258662	-1.58817	3.1042	-2.905726	-1.965492	-2.114858	-3.528504	-0.964644
42	1.860021	3.134164	1.442025	3.521247	-4.840579	-0.394318	-3.21365	-2.670353	1.254752
43	3.555217	5.707632	-0.780465	0.704096	-4.477452	0.804367	-4.379915	-4.797836	-1.764305
44	1.916153	5.739515	-1.423335	1.807526	-5.816218	1.115992	-5.839845	-4.058525	-1.113958
45	2.442387	7.011929	-0.306405	1.172334	-4.854034	2.47003	-5.454558	-5.751007	-0.725026
46	1.396093	6.711322	1.867483	2.646269	-3.14795	3.309829	-3.51718	-4.700699	2.275316
47	1.208966	5.01422	2.582471	3.941558	-2.390547	2.234408	-4.438343	-6.083229	1.458844
48	4.083514	3.375479	1.142838	5.136338	-3.415352	0.702735	-5.291694	-1.849748	0.953905
49	-5.660151	-3.211571	0.648465	-6.302733	-1.59908	-1.15627	6.005999	-2.639585	0.857816
50	-7.986688	1.80286	-0.186294	-6.879368	3.253793	1.561295	6.19273	3.334664	-0.820357
51	2.567334	-2.746928	-1.314866	3.342967	1.441638	1.213946	-2.753529	2.426273	-1.542782
52	3.746608	-1.486404	-1.666701	1.818364	2.327164	1.174948	-1.152246	3.050309	-1.159231
53	6.268789	-4.330768	1.442803	4.686243	5.574967	-1.06737	-1.531434	5.325656	-0.776834
54	5.749364	-2.771241	2.121273	4.588124	4.150696	-2.127708	-2.359723	6.419658	0.343255
55	4.650239	-4.164771	2.159722	3.1331	5.113245	-1.799616	-1.135444	5.291287	0.95727
56	3.043005	-2.538588	1.110215	2.162564	2.866775	-1.212441	-1.63355	2.962349	1.25364
57	4.268866	-0.264603	0.318276	4.086512	0.959196	-1.02694	-3.84468	1.539318	0.411039
58	5.163055	-5.254466	-0.564075	2.936885	4.102194	1.860371	-3.800111	4.420845	-1.587391
59	4.157608	-4.241975	-1.611561	1.990665	4.878974	0.569671	-4.92765	3.73145	-0.400038
60	3.489074	-4.899295	-0.099586	3.519057	5.596637	1.110904	-4.650857	5.482841	-0.450107
61	6.060857	-1.794061	-0.176345	5.361113	2.786813	-0.164011	-4.057798	3.83346	2.010448
	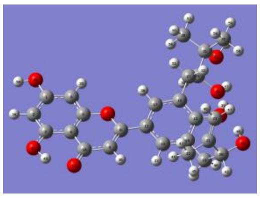 Conformer 10			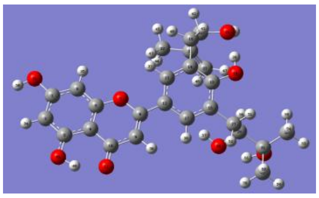 Conformer 11			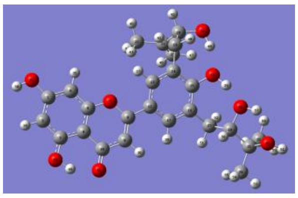 Conformer 12		
1	-5.495153	2.330274	0.264249	6.077825	1.061748	0.549716	6.263566	0.696698	-0.221691
2	-6.428922	1.28765	0.175619	6.640367	-0.221864	0.496436	6.718838	-0.616557	-0.034455
3	-5.984651	-0.01945	-0.002238	5.832736	-1.313393	0.18907	5.796863	-1.650593	0.101295
4	-4.593309	-0.29566	-0.095213	4.44702	-1.128547	-0.067793	4.402701	-1.377167	0.052081
5	-3.698999	0.783949	-0.000056	3.933227	0.177412	-0.003699	3.998739	-0.044834	-0.138064
6	-4.118227	2.093934	0.177494	4.717918	1.279816	0.300384	4.898486	1.001399	-0.275658
7	-2.349144	0.575891	-0.08939	2.605599	0.412316	-0.237243	2.66919	0.276077	-0.187212
8	-1.842658	-0.6781	-0.248011	1.757527	-0.605765	-0.553988	1.711947	-0.686013	-0.077432
9	-2.656728	-1.768728	-0.329032	2.198625	-1.893455	-0.64647	2.038863	-1.998781	0.090765
10	-4.091038	-1.64557	-0.271238	3.573876	-2.240135	-0.397795	3.411116	-2.428897	0.180711
11	-4.862862	-2.637599	-0.355016	4.002154	-3.423115	-0.469733	3.736893	-3.634264	0.347422
12	-0.379818	-0.682687	-0.300844	0.389756	-0.139063	-0.773357	0.359213	-0.135155	-0.172003
13	0.322744	-1.807953	-0.760552	0.119936	1.227113	-0.943539	0.150427	1.161216	-0.6678
14	1.713066	-1.842209	-0.805089	-1.169467	1.708294	-1.159683	-1.124152	1.710144	-0.784586
15	2.416741	-0.694964	-0.390349	-2.232426	0.780743	-1.196752	-2.222015	0.918842	-0.40094

16	1.745334	0.442032	0.099949	-1.988775	-0.600947	-1.032389	-2.045799	-0.37109	0.141012
17	0.353306	0.433529	0.126782	-0.688565	-1.038936	-0.816707	-0.755159	-0.880199	0.24086
18	3.779565	-0.729304	-0.452077	-3.522671	1.139317	-1.384398	-3.470341	1.454431	-0.532504
19	2.422924	-3.076595	-1.31882	-1.376916	3.196975	-1.346317	-1.295475	3.110189	-1.334226
20	3.320206	-3.837138	-0.293797	-2.258725	3.891156	-0.282286	-1.887627	4.171413	-0.356232
21	2.669023	-3.914969	1.078304	-1.992313	3.384531	1.119742	-1.313853	4.032081	1.045347
22	1.379126	-4.690797	1.150065	-0.66911	3.803296	1.703549	0.169574	4.265146	1.174455
23	3.214575	-3.326863	2.145254	-2.853708	2.597753	1.771084	-2.086314	3.722626	2.089144
24	4.642993	-3.323775	-0.265652	-3.620314	3.703737	-0.709316	-3.306534	4.187762	-0.389153
25	-6.87594	-1.021764	-0.085405	6.367506	-2.545186	0.135127	6.228782	-2.910769	0.278706
26	-5.880376	3.619797	0.438126	6.823575	2.156414	0.845794	7.123896	1.736971	-0.359503
27	2.51067	1.626007	0.653137	-3.152045	-1.559426	-0.969472	-3.221792	-1.161098	0.675995
28	2.780636	4.831312	0.639406	-6.216538	-1.922222	-0.249705	-4.685259	-4.024649	0.716718
29	3.105897	2.543194	-0.416182	-3.810563	-1.510667	0.419193	-4.069551	-1.830354	-0.406652
30	4.061624	1.777322	-1.16259	-2.878613	-1.924756	1.432015	-4.627573	-0.792969	-1.224848
31	3.800456	3.813519	0.128618	-5.049474	-2.410302	0.60501	-5.218183	-2.721032	0.122344
32	4.862097	3.493396	1.182862	-4.740342	-3.888035	0.331793	-6.130268	-1.980668	1.102501
33	4.442324	4.331524	-1.062686	-5.464224	-2.257379	1.974296	-5.949739	-3.01005	-1.094563
34	-7.494165	1.484405	0.244268	7.696828	-0.378259	0.690651	7.780314	-0.83989	0.005539
35	-3.406217	2.906718	0.246132	4.294131	2.275177	0.344922	4.558009	2.019101	-0.419378
36	-2.235308	-2.760759	-0.42211	1.517448	-2.685619	-0.927351	1.264202	-2.752199	0.143876
37	-0.222802	-2.671675	-1.125754	0.941395	1.934099	-0.919709	1.003517	1.746921	-0.990396
38	-0.170119	1.301021	0.512519	-0.524846	-2.098786	-0.652427	-0.620655	-1.861051	0.685059
39	4.124716	0.180208	-0.647999	-3.651314	2.109216	-1.22151	-4.119693	0.732113	-0.735765
40	3.069755	-2.822141	-2.166699	-0.397741	3.682585	-1.345732	-1.95079	3.100166	-2.213222
41	1.663468	-3.768353	-1.695517	-1.831546	3.413526	-2.321418	-0.315435	3.459425	-1.672901
42	3.419271	-4.862911	-0.675717	-2.038717	4.967055	-0.319175	-1.581064	5.152	-0.74671
43	1.037875	-4.798165	2.183781	0.164002	3.367032	1.137536	0.746632	3.48931	0.657005
44	0.580058	-4.197729	0.5834	-0.57512	3.47785	2.742899	0.48031	4.268426	2.223296
45	1.498228	-5.694155	0.720024	-0.543774	4.892707	1.666033	0.457987	5.225339	0.726394
46	2.742883	-3.377419	3.123273	-2.63962	2.243458	2.775231	-1.672661	3.617322	3.088831
47	4.151637	-2.785255	2.070461	-3.788514	2.26547	1.330414	-3.154585	3.573573	1.972593
48	4.564027	-2.350486	-0.253166	-4.212248	3.967789	0.015333	-3.596955	3.255595	-0.369903
49	-6.333428	-1.856583	-0.206764	5.609523	-3.158234	-0.101872	5.401186	-3.473163	0.346788
50	-6.849671	3.667015	0.486013	7.745311	1.891219	1.000383	8.038377	1.412862	-0.308267
51	1.835686	2.213129	1.281405	-3.893509	-1.303965	-1.731228	-2.844654	-1.933207	1.351745
52	3.321874	1.266349	1.296711	-2.800585	-2.576947	-1.16364	-3.865648	-0.499372	1.266721
53	3.288375	5.762326	0.913433	-7.121005	-2.477603	0.01907	-5.520358	-4.682009	0.982044
54	2.042931	5.057541	-0.136926	-6.025501	-2.078838	-1.315148	-4.053516	-4.546644	-0.008873
55	2.254967	4.464932	1.526824	-6.403117	-0.856672	-0.079397	-4.101296	-3.844513	1.624887
56	2.309436	2.868018	-1.100884	-4.129583	-0.480245	0.622488	-3.424171	-2.460215	-1.035542
57	4.652333	2.430998	-1.58514	-2.19378	-1.240231	1.495033	-5.400751	-1.193634	-1.668153
58	4.407394	3.144931	2.11463	-3.885351	-4.218387	0.929498	-5.623504	-1.789135	2.052673
59	5.439974	4.394947	1.417262	-5.610362	-4.494386	0.602485	-7.015858	-2.589078	1.320047

60	5.548959	2.723901	0.818703	-4.51262	-4.067793	-0.724184	-6.457901	-1.025893	0.681086
61	5.12861	4.959774	-0.786547	-4.632954	-2.278491	2.482312	-6.836959	-3.31565	-0.847164
	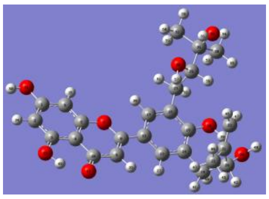 Conformer 13			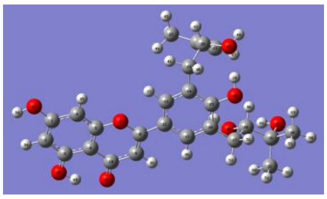 Conformer 14			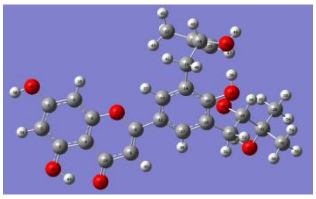 Conformer 15		
1	-5.639151	2.107567	-0.223914	6.24953	0.967421	-0.108444	6.058278	1.070507	0.586293
2	-6.471546	1.055732	0.18568	6.721832	-0.315278	0.20527	6.620974	-0.212954	0.531133
3	-5.933301	-0.215319	0.366714	5.822469	-1.373643	0.301267	5.816947	-1.301966	0.206028
4	-4.549873	-0.446239	0.136067	4.435178	-1.155815	0.081709	4.434502	-1.114792	-0.066843
5	-3.759117	0.640965	-0.272095	4.013328	0.147616	-0.228771	3.920479	0.191056	0.000188
6	-4.272637	1.915744	-0.458261	4.890227	1.217437	-0.329306	4.701626	1.290914	0.32163
7	-2.422554	0.474089	-0.514379	2.690179	0.412726	-0.454579	2.595702	0.428199	-0.247688
8	-1.825507	-0.738584	-0.346472	1.751474	-0.570983	-0.370778	1.751962	-0.587618	-0.581665
9	-2.5353	-1.828198	0.066309	2.097002	-1.854038	-0.06053	2.193185	-1.874389	-0.679777
10	-3.950179	-1.754818	0.322217	3.465688	-2.231653	0.177553	3.565509	-2.223664	-0.416072
11	-4.628016	-2.750103	0.693887	3.807844	-3.411598	0.458521	3.993809	-3.406134	-0.491416
12	-0.394389	-0.696874	-0.641529	0.401888	-0.073526	-0.633751	0.386111	-0.119712	-0.812636
13	0.33985	-1.877637	-0.829812	0.150397	1.304654	-0.711776	0.117819	1.246394	-0.984788
14	1.707402	-1.868044	-1.089414	-1.125212	1.815935	-0.941131	-1.169677	1.725849	-1.212378
15	2.366629	-0.620438	-1.160878	-2.192796	0.906258	-1.097066	-2.229905	0.797136	-1.276978
16	1.64884	0.583319	-0.996687	-1.964479	-0.48677	-1.045778	-1.993713	-0.58122	-1.077559
17	0.284387	0.529371	-0.736477	-0.678611	-0.954789	-0.809397	-0.693862	-1.01709	-0.847
18	3.692839	-0.497769	-1.394455	-3.471247	1.292092	-1.308788	-3.513489	1.155522	-1.506688
19	2.4277	-3.184472	-1.295135	-1.31276	3.317222	-1.014873	-1.391103	3.21798	-1.334258
20	3.533536	-3.513503	-0.26516	-2.235514	3.935427	0.060917	-2.286311	3.835147	-0.231052
21	3.147865	-3.133354	1.149061	-2.032542	3.319668	1.429349	-2.033894	3.223989	1.132437
22	2.09691	-4.010206	1.776126	-0.735053	3.684174	2.100573	-0.731026	3.623351	1.772198
23	3.677939	-2.078303	1.774474	-2.923256	2.489436	1.979361	-2.883438	2.361559	1.699193
24	4.723091	-2.850611	-0.731196	-3.578682	3.795261	-0.436858	-3.642534	3.675786	-0.686694
25	-6.726505	-1.225853	0.761748	6.269717	-2.604607	0.602877	6.352163	-2.533434	0.150192
26	-6.116889	3.363604	-0.413962	7.08722	2.030061	-0.213866	6.800562	2.162684	0.899287
27	2.388768	1.898036	-0.991797	-3.130855	-1.438	-1.146073	-3.157067	-1.537938	-0.983627
28	5.139567	3.354136	-0.404106	-6.231611	-1.846864	-0.635112	-5.788671	-2.137239	1.853206
29	3.066439	2.126143	0.369575	-3.864051	-1.532434	0.202283	-3.976447	-1.317427	0.305698
30	2.082632	2.206135	1.414029	-2.990053	-2.061554	1.213122	-3.114347	-1.213714	1.450077
31	3.905348	3.414472	0.492486	-5.113868	-2.436165	0.221753	-4.926024	-2.492094	0.632833
32	3.077163	4.67556	0.213967	-4.794759	-3.877668	-0.195637	-5.811796	-2.861069	-0.55429
33	4.394964	3.456741	1.845044	-5.602058	-2.430726	1.57519	-4.123197	-3.645711	0.93072
34	-7.529832	1.216855	0.365705	7.778622	-0.496265	0.375142	7.674822	-0.37111	0.737643
35	-3.639693	2.734979	-0.775317	4.537158	2.211806	-0.571957	4.277631	2.286094	0.368008
36	-2.037948	-2.775498	0.226801	1.338229	-2.620283	0.025259	1.51505	-2.66344	-0.97654

37	-0.166961	-2.836625	-0.801333	0.973531	1.999086	-0.589026	0.937184	1.954988	-0.94351
38	-0.258454	1.456087	-0.589566	-0.524217	-2.027813	-0.77186	-0.533007	-2.073217	-0.657268
39	4.164871	-1.355746	-1.236789	-3.599285	2.247758	-1.075952	-3.655491	2.117437	-1.311038
40	2.897743	-3.226659	-2.285867	-1.720977	3.61617	-1.988779	-1.848961	3.480377	-2.296231
41	1.688419	-3.989104	-1.267892	-0.330759	3.789898	-0.931977	-0.418464	3.715505	-1.304868
42	3.713302	-4.596592	-0.305954	-2.007167	5.008763	0.11666	-2.069051	4.911021	-0.186159
43	1.143538	-3.92533	1.238783	-0.685692	3.275832	3.113397	0.121247	3.264764	1.180705
44	1.920254	-3.731931	2.818391	0.120873	3.291379	1.536791	-0.640403	3.205999	2.778452
45	2.388235	-5.067521	1.741258	-0.607129	4.772532	2.156071	-0.637244	4.714666	1.836424
46	3.383042	-1.821278	2.787698	-2.753594	2.056874	2.961092	-2.676443	1.929118	2.673719
47	4.406565	-1.426429	1.302555	-3.838507	2.197181	1.474027	-3.803306	2.045876	1.217253
48	5.392153	-2.881793	-0.02641	-4.199598	4.009609	0.279909	-4.243392	3.881166	0.049659
49	-6.128489	-2.028805	0.826574	5.455816	-3.191037	0.618366	5.597541	-3.144853	-0.100629
50	-7.070095	3.381763	-0.22722	8.000042	1.742887	-0.046407	7.720332	1.896129	1.062999
51	1.686515	2.714583	-1.183396	-3.829695	-1.099367	-1.915646	-2.784558	-2.566591	-0.984139
52	3.146649	1.908375	-1.779772	-2.770212	-2.431885	-1.426746	-3.820963	-1.415283	-1.844255
53	4.868851	3.405697	-1.46251	-6.423843	-0.804771	-0.358808	-6.441706	-1.283441	1.638238
54	5.794126	4.202349	-0.178649	-7.150892	-2.420213	-0.477297	-6.4148	-2.993607	2.123478
55	5.700803	2.430039	-0.230042	-5.984289	-1.887448	-1.6997	-5.158091	-1.879281	2.708496
56	3.741197	1.284352	0.573113	-4.191067	-0.52716	0.498286	-4.564002	-0.397389	0.202522
57	1.679233	1.327923	1.501973	-2.307868	-1.393914	1.387685	-2.360596	-0.653515	1.2008
58	3.681525	5.560778	0.435349	-5.681206	-4.501626	-0.044926	-6.509437	-3.652798	-0.262246
59	2.759731	4.730707	-0.832667	-4.506272	-3.941839	-1.250131	-6.395693	-1.996004	-0.886196
60	2.184233	4.695776	0.846094	-3.977478	-4.280966	0.410002	-5.216514	-3.226236	-1.39521
61	3.629942	3.192862	2.387739	-4.799768	-2.514161	2.121893	-3.4078	-3.300228	1.496086
	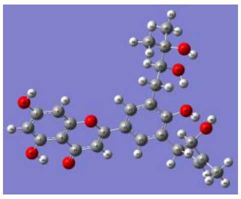 Conformer 16			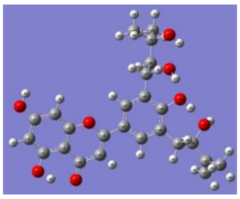 Conformer 17			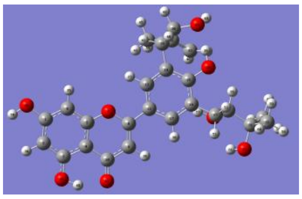 Conformer 18		
1	-6.131322	0.952491	0.457033	5.745887	1.759382	0.660945	5.928017	1.649091	-0.021787
2	-6.661888	-0.345775	0.464423	6.540739	0.61041	0.537359	6.68481	0.468853	0.013063
3	-5.823699	-1.431872	0.22884	5.95282	-0.594434	0.162649	6.037447	-0.760925	0.035945
4	-4.438582	-1.226634	-0.016315	4.5558	-0.660208	-0.091586	4.615347	-0.823311	0.025783
5	-3.957163	0.093357	-0.013629	3.80428	0.518921	0.045844	3.903477	0.385502	-0.010413
6	-4.772722	1.190632	0.218697	4.367739	1.730268	0.417607	4.528482	1.625731	-0.034034
7	-2.63169	0.348029	-0.239338	2.455042	0.511459	-0.182393	2.535614	0.387562	-0.016224
8	-1.755697	-0.663928	-0.490023	1.813672	-0.627768	-0.562951	1.8336	-0.779625	-0.00849
9	-2.163215	-1.964488	-0.52105	2.489184	-1.800748	-0.72704	2.465737	-1.987564	0.008679
10	-3.534451	-2.331862	-0.274065	3.907306	-1.894993	-0.491321	3.902579	-2.08752	0.040431
11	-3.931478	-3.527238	-0.289067	4.549508	-2.970005	-0.629072	4.507063	-3.192327	0.067001
12	-0.394367	-0.1742	-0.71465	0.379228	-0.413372	-0.763577	0.38742	-0.557092	-0.033773
13	-0.157142	1.188986	-0.94308	-0.150388	0.88588	-0.812132	-0.50657	-1.58802	0.292671

14	1.124823	1.686382	-1.166871	-1.508008	1.109482	-1.010799	-1.887026	-1.405518	0.259613
15	2.201663	0.780138	-1.147508	-2.366391	0.00343	-1.154344	-2.381305	-0.144411	-0.133352
16	1.998201	-0.595214	-0.923667	-1.867679	-1.314748	-1.111023	-1.508536	0.917752	-0.439395
17	0.702567	-1.049948	-0.708645	-0.500529	-1.495304	-0.916838	-0.137806	0.697836	-0.382904
18	3.493886	1.180202	-1.342881	-3.690364	0.277256	-1.329922	-3.715118	0.122126	-0.203261
19	1.314016	3.17039	-1.399256	-2.065069	2.513173	-1.018054	-2.7978	-2.532317	0.702177
20	2.090146	3.91535	-0.288008	-2.630172	2.943838	0.355585	-3.651387	-3.173496	-0.409301
21	1.665087	3.496447	1.10317	-3.122497	4.380404	0.322144	-4.133267	-4.559781	-0.027203
22	0.346186	4.062133	1.558509	-4.38696	4.646334	-0.45143	-3.110077	-5.657057	-0.164116
23	2.38436	2.651064	1.847169	-2.445061	5.341843	0.956169	-5.380861	-4.773515	0.397859
24	3.487661	3.683219	-0.550773	-3.670046	2.068571	0.807246	-4.746463	-2.265184	-0.633889
25	-6.32815	-2.677431	0.232978	6.710631	-1.697582	0.040854	6.757335	-1.89608	0.067191
26	-6.908573	2.042174	0.680513	6.272821	2.956447	1.02259	6.625064	2.813324	-0.042886
27	3.164698	-1.556643	-0.861558	-2.753286	-2.53237	-1.285084	-2.07271	2.265016	-0.821203
28	5.538526	-2.640179	2.052213	-2.511479	-3.909712	1.574702	-2.092655	5.438917	-0.584973
29	3.880925	-1.556666	0.500099	-3.856979	-2.798555	-0.254012	-2.459551	3.119745	0.396373
30	4.467451	-0.276243	0.786659	-4.929133	-1.86902	-0.497235	-3.331236	2.424089	1.298121
31	5.0288	-2.597081	0.603775	-3.492064	-2.783125	1.248285	-3.103624	4.487857	0.049515
32	4.614699	-3.993445	0.147588	-2.987521	-1.440322	1.787375	-4.353408	4.336583	-0.827437
33	6.087732	-2.168857	-0.265438	-4.786052	-3.06597	1.839891	-3.483184	5.076847	1.304471
34	-7.717574	-0.517411	0.649926	7.608713	0.645847	0.728363	7.767298	0.518284	0.02148
35	-4.373592	2.197106	0.216219	3.763753	2.623354	0.517001	3.938207	2.534959	-0.060024
36	-1.459418	-2.753826	-0.748887	1.970229	-2.691894	-1.053595	1.892772	-2.904875	-0.018475
37	-0.994113	1.877405	-0.957435	0.509643	1.737644	-0.694469	-0.125814	-2.550921	0.617156
38	0.556897	-2.10625	-0.507724	-0.123504	-2.511265	-0.868581	0.536117	1.509137	-0.634223
39	3.59223	2.1512	-1.13942	-4.251231	-0.510797	-1.08013	-4.229392	-0.719387	-0.332756
40	1.842761	3.360828	-2.341657	-1.273786	3.219609	-1.287107	-2.179599	-3.308218	1.159618
41	0.329238	3.634736	-1.495064	-2.850751	2.601875	-1.775692	-3.49522	-2.189825	1.477953
42	1.900006	4.989921	-0.412591	-1.81413	2.872211	1.086548	-3.04632	-3.243915	-1.323806
43	0.34071	5.157895	1.501682	-4.690281	5.694107	-0.372646	-3.522796	-6.622796	0.139676
44	-0.473557	3.707551	0.919864	-5.200469	4.014931	-0.077025	-2.76325	-5.740365	-1.202567
45	0.122131	3.765007	2.586335	-4.266257	4.407296	-1.516043	-2.219668	-5.462137	0.447304
46	2.048886	2.359381	2.838303	-1.542249	5.122654	1.522124	-5.718754	-5.768496	0.674101
47	3.30616	2.194186	1.500928	-2.763706	6.381147	0.929739	-6.09858	-3.963565	0.475388
48	3.996373	3.909434	0.246362	-3.966119	1.539844	0.042741	-5.182569	-2.490915	-1.471677
49	-5.552755	-3.283866	0.041045	6.081798	-2.428302	-0.235788	6.087321	-2.641683	0.075933
50	-7.82696	1.76272	0.830417	7.229147	2.862503	1.166028	6.00871	3.563969	-0.064456
51	2.78572	-2.563117	-1.053746	-3.259549	-2.484806	-2.258566	-2.950815	2.120041	-1.458143
52	3.900888	-1.333822	-1.641218	-2.113542	-3.418248	-1.321092	-1.331729	2.816474	-1.407402
53	6.418697	-3.288163	2.113587	-2.888785	-4.871006	1.211603	-1.191635	5.514348	0.033444
54	5.816959	-1.638048	2.391441	-2.373514	-3.98098	2.658888	-1.80386	5.106158	-1.586405
55	4.770596	-3.031734	2.729475	-1.531338	-3.722383	1.124273	-2.531939	6.438097	-0.67305
56	3.140376	-1.773478	1.282907	-4.244934	-3.8056	-0.457678	-1.553233	3.333168	0.977718
57	4.311619	0.30537	0.016458	-5.47591	-1.888372	0.312923	-3.83139	1.754856	0.794686

58	5.417388	-4.70753	0.359014	-1.96368	-1.237491	1.470486	-5.051821	3.619359	-0.382033
59	4.417185	-4.016528	-0.927401	-2.99088	-1.474928	2.883839	-4.110337	3.998486	-1.840061
60	3.715552	-4.325538	0.677922	-3.617371	-0.604447	1.473002	-4.859878	5.303713	-0.902119
61	6.186169	-1.220872	-0.057097	-4.736005	-2.847983	2.784344	-3.886417	4.33682	1.796175
		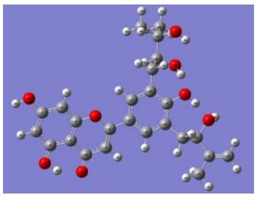 Conformer 19			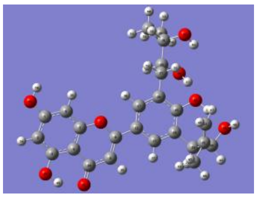 Conformer 20			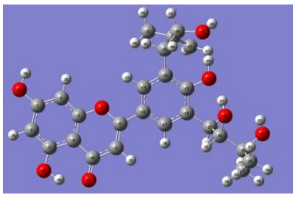 Conformer 21	
1	5.983595	1.543062	-0.320038	-5.511651	2.086641	0.896067	6.250409	0.690716	-0.294597
2	6.691695	0.40562	0.094331	-6.436014	1.167964	0.377838	6.711503	-0.616429	-0.081218
3	6.003261	-0.776857	0.350071	-5.980229	-0.004387	-0.213634	5.795377	-1.646556	0.096926
4	4.591634	-0.831618	0.191066	-4.584456	-0.272023	-0.292603	4.39794	-1.377322	0.064798
5	3.929304	0.335936	-0.224414	-3.700155	0.678336	0.239919	3.987317	-0.053214	-0.15267
6	4.593738	1.524904	-0.485043	-4.132212	1.856512	0.835457	4.884062	0.991975	-0.333402
7	2.571676	0.338154	-0.398269	-2.348872	0.47268	0.195599	2.657826	0.266055	-0.187619
8	1.832375	-0.778728	-0.154102	-1.827064	-0.647944	-0.376501	1.702653	-0.692637	-0.032422
9	2.413043	-1.93786	0.267455	-2.632894	-1.603505	-0.921508	2.035719	-1.999772	0.165549
10	3.837805	-2.042946	0.45568	-4.068043	-1.480881	-0.907135	3.409965	-2.42617	0.239301
11	4.397237	-3.10597	0.834789	-4.829682	-2.355597	-1.398761	3.740207	-3.626383	0.431567
12	0.403044	-0.558438	-0.382252	-0.363897	-0.664096	-0.326679	0.348427	-0.144178	-0.113829
13	-0.48248	-1.63887	-0.50809	0.360595	-1.810351	-0.687633	0.131336	1.146717	-0.617788
14	-1.848261	-1.451783	-0.706657	1.752018	-1.848235	-0.654562	-1.145001	1.697646	-0.715218
15	-2.337684	-0.130375	-0.756333	2.436204	-0.691483	-0.22722	-2.235363	0.923877	-0.272162
16	-1.469738	0.974934	-0.682028	1.740625	0.476711	0.136914	-2.052082	-0.377673	0.23616
17	-0.111309	0.742823	-0.494171	0.351273	0.471982	0.083608	-0.76304	-0.890512	0.30865
18	-3.667577	0.143131	-0.908675	3.794723	-0.622191	-0.159541	-3.52191	1.368348	-0.319057
19	-2.74263	-2.658752	-0.903795	2.465327	-3.104145	-1.113391	-1.307412	3.074964	-1.32576
20	-3.722715	-2.950674	0.249275	3.355794	-3.809846	-0.067244	-1.918534	4.159692	-0.411582
21	-4.208568	-4.386998	0.233538	2.716613	-3.869053	1.303449	-1.375685	4.109724	1.000411
22	-3.23688	-5.401165	0.778678	1.552974	-4.817686	1.417969	0.052049	4.565094	1.146185
23	-5.416848	-4.710763	-0.233333	3.136037	-3.11148	2.320521	-2.100562	3.659673	2.028151
24	-4.804539	-2.013454	0.086793	4.61825	-3.116632	-0.082413	-3.346759	3.985354	-0.483219
25	6.677522	-1.86871	0.749101	-6.860941	-0.888915	-0.713421	6.230931	-2.902242	0.300028
26	6.610817	2.717491	-0.581838	-6.026377	3.209164	1.458989	7.198876	1.647079	-0.46072
27	-1.996744	2.389638	-0.782137	2.488877	1.712908	0.581834	-3.233267	-1.192189	0.712792
28	-3.38002	4.908568	1.868499	4.040598	4.737685	-1.347827	-5.721556	-3.605651	-1.093343
29	-2.543926	2.937295	0.545705	3.006516	2.573467	-0.582509	-3.982837	-1.914896	-0.418741
30	-3.653967	2.159241	1.018363	3.918474	1.848424	-1.421721	-4.512027	-0.994886	-1.385536
31	-3.043197	4.404978	0.456887	3.754417	3.855242	-0.123582	-5.180526	-2.770869	0.07698
32	-2.0396	5.339819	-0.212603	2.991026	4.649376	0.932835	-4.823733	-3.677133	1.252
33	-4.229387	4.41323	-0.351069	4.99404	3.446321	0.473575	-6.200211	-1.870852	0.536168
34	7.76945	0.433433	0.220176	-7.498183	1.373704	0.439054	7.77569	-0.819372	-0.056609

35	4.05746	2.408956	-0.806164	-3.413066	2.562721	1.235607	4.522459	2.001131	-0.496999
36	1.805392	-2.805576	0.487445	-2.2065	-2.479838	-1.390774	1.264515	-2.752838	0.258024
37	-0.103476	-2.655244	-0.484836	-0.164181	-2.704429	-1.006392	0.978204	1.729337	-0.961833
38	0.560252	1.59092	-0.421835	-0.186843	1.367458	0.372392	-0.626787	-1.879952	0.732789
39	-4.208263	-0.634849	-0.600368	4.21581	-1.524924	-0.185679	-3.569231	2.356173	-0.441973
40	-3.346721	-2.556459	-1.814705	1.712904	-3.819993	-1.453888	-1.934445	3.03027	-2.225431
41	-2.104017	-3.532852	-1.050637	3.104476	-2.891439	-1.979929	-0.323772	3.422527	-1.65145
42	-3.214898	-2.754223	1.203521	3.532587	-4.834284	-0.422158	-1.678445	5.136996	-0.851928
43	-3.650986	-6.411749	0.728625	0.717775	-4.492136	0.784499	0.35443	4.585696	2.196452
44	-2.289385	-5.396067	0.224714	1.827917	-5.827232	1.087546	0.734325	3.890506	0.612805
45	-2.987717	-5.180852	1.825098	1.190274	-4.875116	2.447522	0.197126	5.566129	0.721059
46	-6.098746	-3.955524	-0.609787	2.659417	-3.16852	3.294757	-1.688652	3.62831	3.032585
47	-5.757775	-5.7423	-0.244962	3.948899	-2.398825	2.221078	-3.114159	3.289418	1.909687
48	-5.323557	-1.977218	0.906724	5.144543	-3.409669	0.680929	-3.762131	4.521285	0.213789
49	5.985323	-2.583796	0.872809	-6.310054	-1.640152	-1.083628	5.405754	-3.463042	0.397141
50	7.56627	2.61624	-0.437433	-5.305135	3.774054	1.782354	6.773197	2.509008	-0.601317
51	-2.787431	2.458898	-1.536818	3.33906	1.44144	1.216677	-3.945812	-0.557884	1.250765
52	-1.177465	3.033246	-1.111371	1.811871	2.322723	1.184833	-2.86762	-1.943481	1.416898
53	-3.83799	5.90093	1.805931	4.665077	5.586875	-1.052149	-5.959527	-2.962633	-1.945867
54	-4.082077	4.229347	2.361113	4.56751	4.166723	-2.11803	-6.63227	-4.12819	-0.783533
55	-2.478455	4.980215	2.488045	3.11099	5.124119	-1.781723	-4.986819	-4.351159	-1.419154
56	-1.74538	2.884399	1.299745	2.148318	2.872442	-1.201174	-3.273586	-2.574957	-0.938207
57	-3.839749	1.461503	0.359572	4.078163	0.969464	-1.028212	-4.34499	-0.083531	-1.078131
58	-1.060995	5.273765	0.275344	2.928048	4.097701	1.874571	-5.66457	-4.340671	1.479444
59	-2.387591	6.375328	-0.137907	1.975863	4.876932	0.589685	-3.954298	-4.299256	1.013145
60	-1.92077	5.099285	-1.272412	3.503789	5.596746	1.129353	-4.602072	-3.093309	2.149316
61	-4.748323	3.66487	-0.000342	5.350062	2.797159	-0.161866	-6.254935	-1.205556	-0.175392
	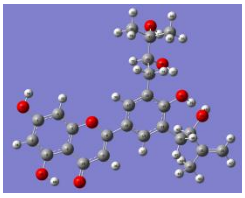 Conformer 22								
1	5.869693	1.66155	0.3549						
2	6.647435	0.494462	0.349365						
3	6.031612	-0.739246	0.173127						
4	4.620941	-0.818673	0.000083						
5	3.88745	0.37732	0.013505						
6	4.48064	1.620886	0.188586						
7	2.529319	0.36214	-0.144508						
8	1.856718	-0.807091	-0.334033						
9	2.513006	-2.001844	-0.368174						
10	3.940805	-2.085724	-0.194528						
11	4.564871	-3.179714	-0.216454						

12	0.416449	-0.595034	-0.491934		
13	-0.479392	-1.674609	-0.50682		
14	-1.85172	-1.495049	-0.664391		
15	-2.340717	-0.177484	-0.784804		
16	-1.464228	0.924277	-0.801474		
17	-0.101418	0.700957	-0.649394		
18	-3.671022	0.095059	-0.917572		
19	-2.754466	-2.71052	-0.728422		
20	-3.715052	-2.890074	0.463957		
21	-4.179677	-4.326236	0.608044		
22	-3.190215	-5.259463	1.255843		
23	-5.384635	-4.716837	0.18589		
24	-4.812845	-1.99	0.217938		
25	6.771321	-1.862034	0.165046		
26	6.534551	2.831683	0.529783		
27	-2.010109	2.324952	-0.954115		
28	-1.993473	5.384957	-0.048776		
29	-2.489003	2.92589	0.376082		
30	-3.455836	2.090395	1.027038		
31	-3.076538	4.357411	0.267664		
32	-4.232214	4.436552	-0.737857		
33	-3.573617	4.682102	1.576844		
34	7.721312	0.556961	0.480413		
35	3.874967	2.520239	0.19433		
36	1.970549	-2.921958	-0.538941		
37	-0.110645	-2.68845	-0.393401		
38	0.572369	1.550014	-0.655246		
39	-4.209736	-0.664145	-0.564151		
40	-3.375798	-2.691417	-1.63346		
41	-2.121825	-3.597833	-0.805844		
42	-3.198037	-2.586495	1.384582		
43	-2.953295	-4.929879	2.276107		
44	-2.239087	-5.287878	0.708838		
45	-3.581791	-6.279017	1.305482		
46	-6.079956	-4.017101	-0.265791		
47	-5.709623	-5.748529	0.287959		
48	-5.325385	-1.881141	1.035624		
49	6.122804	-2.612852	0.022194		
50	5.904678	3.571337	0.512385		
51	-2.837171	2.319871	-1.670857		
52	-1.230065	2.972106	-1.366015		
53	-2.408677	6.394963	0.031983		
54	-1.605928	5.258289	-1.064056		
55	-1.160993	5.299347	0.658032		

56	-1.634579	2.983155	1.06353						
57	-3.85964	1.512435	0.351661						
58	-3.89117	4.309841	-1.770579						
59	-4.980667	3.665594	-0.523791						
60	-4.715911	5.414658	-0.655221						
61	-4.042003	3.872871	1.855365						

Table S6. Conformational analysis of (2″*S*,2‴*S*)-**3**

Conformers	Gibbs Free Energy (Hartree)	Relative Gibbs Free Energy (kcal/mol)	Population (%)
1	-1494.613246	0.91553709	3.19
2	-1494.612866	1.15399089	2.14
3	-1494.612699	1.25878506	1.79
4	-1494.614438	0.16754517	11.29
5	-1494.612449	1.41566256	1.37
6	-1494.613649	0.66265056	4.90
7	-1494.613418	0.80760537	3.83
8	-1494.613322	0.86784633	3.46
9	-1494.612875	1.1483433	2.16
10	-1494.613797	0.56977908	5.73
11	-1494.613111	1.00025094	2.77
12	-1494.613301	0.88102404	3.39
13	-1494.61421	0.31061745	8.87
14	-1494.612685	1.2675702	1.76
15	-1494.613319	0.86972886	3.45
16	-1494.614705	0	14.98
17	-1494.613266	0.90298689	3.26
18	-1494.612742	1.23180213	1.87
19	-1494.613099	1.00778106	2.73
20	-1494.613676	0.64570779	5.04
21	-1494.613718	0.61935237	5.27
22	-1494.613952	0.47251503	6.75

Table S7. Coordinates of (2″*S*,2‴*S*)-**3**

	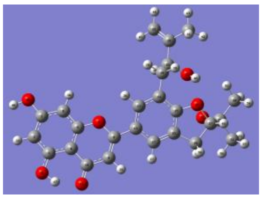 Conformer 1			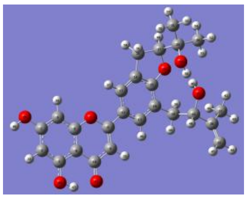 Conformer 2			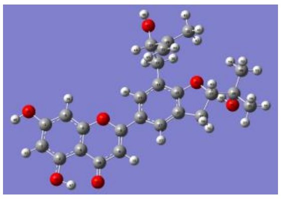 Conformer 3		
1	5.740102	1.917546	0.442626	6.264306	-1.577467	-0.065546	-6.00207	1.590236	-0.26915
2	6.617316	0.823575	0.41783	6.882285	-0.352736	0.226047	-6.761787	0.430437	-0.057377
3	6.112303	-0.457371	0.213425	6.107399	0.798046	0.339238	-6.116487	-0.790741	0.116814

4	4.716336	-0.655592	0.032607	4.698983	0.732546	0.158668	-4.697274	-0.861387	0.082246
5	3.880395	0.47331	0.064798	4.128612	-0.518357	-0.130846	-3.982719	0.328721	-0.133404
6	4.360597	1.758593	0.266448	4.880146	-1.678015	-0.248177	-4.603372	1.556323	-0.310039
7	2.528069	0.339555	-0.098154	2.778298	-0.637409	-0.319723	-2.614972	0.319186	-0.170432
8	1.965026	-0.881368	-0.314133	1.956098	0.442861	-0.210899	-1.915373	-0.839833	-0.020076
9	2.724093	-2.01261	-0.37321	2.446979	1.680385	0.084313	-2.552468	-2.030046	0.176508
10	4.152677	-1.973863	-0.190741	3.856387	1.907902	0.276121	-3.988038	-2.115952	0.251774
11	4.871981	-3.007259	-0.232067	4.332134	3.044318	0.539651	-4.595913	-3.203272	0.441797
12	0.511625	-0.805142	-0.476575	0.551241	0.091586	-0.43146	-0.468524	-0.632272	-0.104329
13	-0.1071	0.433973	-0.722357	-0.410438	1.094722	-0.651023	0.041998	0.589228	-0.58109
14	-1.48716	0.559008	-0.903216	-1.761499	0.805956	-0.852602	1.412892	0.839988	-0.686246
15	-2.223892	-0.624502	-0.834377	-2.107586	-0.547403	-0.841055	2.255754	-0.197851	-0.291003
16	-1.647521	-1.864215	-0.560542	-1.185598	-1.565382	-0.608989	1.785616	-1.422053	0.194643
17	-0.282118	-1.968414	-0.380865	0.147823	-1.260725	-0.409155	0.42758	-1.650551	0.291344
18	-2.147163	1.901936	-1.08664	-2.794265	1.893436	-1.003544	1.946725	2.179565	-1.117189
19	-2.802857	2.419629	0.213095	-3.586519	2.137235	0.300344	2.060134	3.160176	0.068806
20	-3.380899	3.811225	0.041236	-4.554517	3.297269	0.168769	3.062237	2.715441	1.117528
21	-4.619927	3.935635	-0.805967	-5.760808	3.083751	-0.707362	4.520966	2.823561	0.759765
22	-2.789998	4.858771	0.622633	-4.315956	4.444695	0.809902	2.63646	2.241004	2.292194
23	-3.819041	1.50461	0.673681	-4.284669	0.941125	0.705601	2.414616	4.423237	-0.506107
24	6.949375	-1.508393	0.186798	6.694889	1.97347	0.62084	-6.839848	-1.905492	0.317511
25	6.185078	3.184391	0.638921	6.97757	-2.725723	-0.185436	-6.584644	2.803379	-0.444602
26	-3.578275	-0.691911	-0.998106	-3.377027	-1.011893	-1.037722	3.61344	-0.126799	-0.323738
27	-4.033188	-2.028907	-0.569392	-3.409271	-2.440616	-0.668284	4.146791	-1.283443	0.409012
28	-2.746446	-2.889579	-0.472553	-1.923095	-2.878167	-0.595166	2.980099	-2.293347	0.494063
29	-4.816213	-1.852759	0.753561	-4.20582	-2.561193	0.653186	5.397036	-1.763292	-0.342201
30	-6.081112	-1.012033	0.519879	-5.666727	-2.136287	0.437591	6.443792	-0.644071	-0.410516
31	-5.223617	-3.21534	1.318836	-4.178774	-4.003036	1.165281	5.972352	-3.005229	0.334925
32	-3.966082	-1.245356	1.729905	-3.579446	-1.760646	1.658967	5.017621	-2.159485	-1.667062
33	7.685332	0.960144	0.555343	7.95658	-0.288368	0.366628	-7.846236	0.469305	-0.028361
34	3.692661	2.610447	0.288475	4.413889	-2.628787	-0.473807	-4.02718	2.458374	-0.473182
35	2.264069	-2.97027	-0.57715	1.778158	2.522952	0.197397	-1.985211	-2.946584	0.267278
36	0.504957	1.327163	-0.774491	-0.106496	2.135816	-0.677464	-0.650539	1.369682	-0.875406
37	0.164221	-2.927481	-0.142342	0.874562	-2.042258	-0.222622	0.060861	-2.592535	0.683879
38	-1.406011	2.639484	-1.408615	-2.308048	2.832169	-1.2854	2.926742	2.073087	-1.591745
39	-2.908445	1.848171	-1.874979	-3.49722	1.642816	-1.807745	1.273053	2.635837	-1.850073
40	-2.040808	2.441852	0.998596	-2.873446	2.35415	1.101955	1.067698	3.227119	0.538351
41	-4.443814	3.594265	-1.834235	-5.474821	2.866123	-1.744498	5.154479	2.489355	1.5864
42	-5.426421	3.314216	-0.399623	-6.345244	2.226514	-0.353279	4.753745	2.208347	-0.115819
43	-4.96826	4.971138	-0.848863	-6.407374	3.965433	-0.713201	4.786891	3.856196	0.508226
44	-3.165843	5.870844	0.494958	-4.978092	5.301572	0.714137	3.330142	1.888367	3.051595
45	-1.904808	4.741317	1.243476	-3.445483	4.569226	1.449891	1.577389	2.177988	2.530477
46	-4.148049	1.023913	-0.10527	-4.450187	0.417861	-0.097569	2.477941	5.061555	0.222788
47	6.370189	-2.311244	0.025839	5.951311	2.645751	0.655253	-6.171422	-2.647597	0.411596

48	7.150371	3.177642	0.748979	7.920441	-2.540978	-0.040818	-7.550625	2.711806	-0.39669
49	-4.705469	-2.380118	-1.354563	-3.948092	-2.94405	-1.473198	4.421522	-0.915267	1.403718
50	-2.695016	-3.614949	-1.291254	-1.655069	-3.505896	-1.451606	2.933685	-2.777523	1.472497
51	-2.699321	-3.446486	0.46528	-1.710299	-3.448601	0.311003	3.093505	-3.070555	-0.270353
52	-6.582217	-0.84123	1.477305	-6.195675	-2.165123	1.394866	6.753312	-0.330844	0.592541
53	-5.862438	-0.03804	0.074504	-5.754518	-1.124356	0.033793	7.325451	-0.999169	-0.952651
54	-6.776119	-1.533117	-0.146964	-6.170494	-2.816099	-0.257612	6.043381	0.23096	-0.931243
55	-5.871972	-3.068224	2.187766	-4.842103	-4.093144	2.030678	6.862906	-3.338284	-0.206775
56	-4.35319	-3.791028	1.643263	-3.174561	-4.297412	1.480183	6.258877	-2.78484	1.368061
57	-5.772411	-3.800475	0.573718	-4.52175	-4.699509	0.393248	5.247674	-3.824458	0.339426
58	-3.817588	-0.312061	1.474592	-3.726283	-0.817771	1.440247	4.585646	-1.384029	-2.06547
	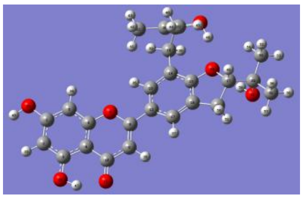 Conformer 4			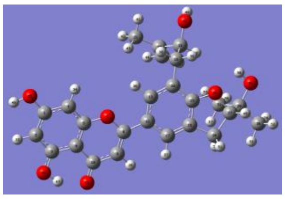 Conformer 5			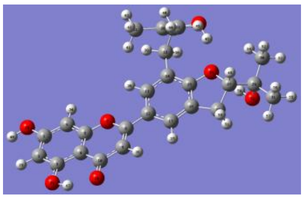 Conformer 6		
1	-5.974485	1.483754	-0.347003	5.810275	1.409373	-0.43703	-5.998856	1.271408	-1.028707
2	-6.709536	0.311865	-0.115564	6.515853	0.303663	0.059317	-6.72789	0.245883	-0.409067
3	-6.038704	-0.890695	0.08832	5.828982	-0.866472	0.370261	-6.050599	-0.805186	0.202693
4	-4.617896	-0.930475	0.06411	4.421764	-0.940729	0.184087	-4.629455	-0.839816	0.197899
5	-3.928769	0.271015	-0.171802	3.761966	0.194371	-0.315877	-3.946515	0.211728	-0.43611
6	-4.575038	1.480419	-0.377868	4.424717	1.370798	-0.632249	-4.599269	1.26901	-1.051594
7	-2.560611	0.291373	-0.199574	2.408841	0.175901	-0.519269	-2.578518	0.225753	-0.475442
8	-1.838307	-0.849168	-0.02277	1.668356	-0.92755	-0.221359	-1.849434	-0.760658	0.115409
9	-2.447922	-2.049101	0.193307	2.248121	-2.054882	0.281834	-2.452334	-1.799378	0.760408
10	-3.882758	-2.165307	0.263369	3.668607	-2.138541	0.50579	-3.887264	-1.912815	0.832557
11	-4.466448	-3.261719	0.472139	4.225909	-3.173063	0.960228	-4.465029	-2.868834	1.414467
12	-0.394671	-0.611506	-0.103605	0.242516	-0.730607	-0.490395	-0.406609	-0.546393	-0.023908
13	0.089151	0.595564	-0.640352	-0.255668	0.563451	-0.731152	0.081489	0.710309	-0.426511
14	1.453544	0.886501	-0.743297	-1.609162	0.814976	-0.965625	1.446736	0.988446	-0.55219
15	2.311768	-0.110723	-0.279973	-2.447835	-0.300689	-0.95399	2.300482	-0.075313	-0.260197
16	1.873059	-1.319849	0.261072	-1.986738	-1.601726	-0.739745	1.857346	-1.338858	0.132671
17	0.519711	-1.582339	0.355391	-0.644587	-1.828725	-0.505641	0.503494	-1.588464	0.250507
18	1.932862	2.205438	-1.306278	-2.152455	2.202226	-1.181347	1.933003	2.36029	-0.96227
19	2.82466	3.066398	-0.361443	-2.859668	2.76525	0.064097	2.88621	3.076129	0.041632
20	2.290457	3.084406	1.061967	-1.945361	2.916396	1.263993	2.413087	2.923956	1.478405
21	0.898386	3.636308	1.233982	-0.764841	3.840839	1.11559	1.046753	3.483424	1.781524
22	3.016931	2.63735	2.089187	-2.215144	2.266987	2.400431	3.167729	2.329928	2.405963
23	4.194414	2.700976	-0.450236	-3.395537	4.032216	-0.340634	4.241144	2.697501	-0.15428
24	-6.738889	-2.016528	0.307699	6.500486	-1.927772	0.848584	-6.744737	-1.78887	0.799798
25	-6.582648	2.679243	-0.552276	6.436036	2.571386	-0.752754	-6.613523	2.317001	-1.636983
26	3.676339	-0.010995	-0.291877	-3.791535	-0.236001	-1.161652	3.66493	-0.001353	-0.32986
27	4.233816	-1.127082	0.494162	-4.362636	-1.574029	-0.9321	4.230679	-1.228312	0.260528

28	3.082144	-2.147094	0.621323	-3.159207	-2.545361	-0.853232	3.060264	-2.233967	0.300508
29	5.489703	-1.616428	-0.241669	-5.253366	-1.480714	0.32195	5.43704	-1.629542	-0.600734
30	6.503883	-0.474891	-0.39135	-6.084209	-2.753428	0.46594	6.473104	-0.498149	-0.634138
31	6.103756	-2.795377	0.510221	-4.446599	-1.192655	1.594305	6.054235	-2.917384	-0.059397
32	5.112381	-2.109305	-1.533172	-6.192284	-0.417776	0.09878	4.986524	-1.921479	-1.929261
33	-7.794697	0.326999	-0.094335	7.59045	0.346422	0.20601	-7.813284	0.259362	-0.397986
34	-4.017794	2.39148	-0.555971	3.890259	2.23039	-1.016628	-4.046932	2.065677	-1.533772
35	-1.860012	-2.950573	0.30267	1.643365	-2.9137	0.540397	-1.858468	-2.559297	1.250337
36	-0.622835	1.333977	-0.990353	0.434633	1.398477	-0.728256	-0.627309	1.502728	-0.637239
37	0.173806	-2.510335	0.796735	-0.28191	-2.839507	-0.355759	0.153522	-2.574995	0.533109
38	2.516489	2.045266	-2.220913	-1.346622	2.882306	-1.473466	2.471716	2.307927	-1.916197
39	1.053128	2.789801	-1.592441	-2.886518	2.199221	-1.994441	1.056288	2.993019	-1.130442
40	2.781782	4.092097	-0.751827	-3.678592	2.082873	0.332856	2.853244	4.143725	-0.214171
41	0.147385	2.987268	0.767521	-1.082535	4.804968	0.703741	0.258279	2.921695	1.266127
42	0.641596	3.737802	2.292309	-0.271552	4.012384	2.076226	0.834858	3.450189	2.854028
43	0.803775	4.622314	0.760283	-0.01861	3.431416	0.423384	0.964384	4.525614	1.445878
44	2.627293	2.647771	3.103712	-1.581491	2.36166	3.278848	2.821122	2.218561	3.429993
45	4.024649	2.260426	1.94927	-3.080384	1.615414	2.492611	4.156915	1.949601	2.173529
46	4.239575	1.741069	-0.282584	-3.840517	4.411838	0.434504	4.268946	1.723387	-0.113197
47	-6.056056	-2.742073	0.421414	5.809422	-2.638776	1.000132	-6.057792	-2.420746	1.16648
48	-7.546763	2.567201	-0.509642	7.387776	2.487725	-0.576093	-7.577034	2.21874	-1.560547
49	4.503069	-0.707309	1.468663	-5.003743	-1.769319	-1.793649	4.557168	-0.957945	1.269846
50	3.031568	-2.575079	1.625306	-3.236721	-3.229242	-0.003673	3.050296	-2.803803	1.232581
51	3.21829	-2.963815	-0.096248	-3.090336	-3.159512	-1.757321	3.137127	-2.938742	-0.534952
52	6.8029	-0.080512	0.585829	-6.670545	-2.934679	-0.440703	6.828515	-0.256935	0.373532
53	7.396158	-0.843451	-0.905994	-5.44959	-3.624476	0.653726	7.329848	-0.804627	-1.241462
54	6.084417	0.348251	-0.978753	-6.77476	-2.645373	1.307955	6.048673	0.410426	-1.073372
55	6.995819	-3.141733	-0.020151	-3.857542	-0.277252	1.485802	6.912152	-3.198965	-0.677228
56	6.395977	-2.49901	1.522407	-5.13767	-1.056921	2.431307	6.399478	-2.77859	0.969842
57	5.399802	-3.629781	0.578439	-3.76192	-2.008816	1.844599	5.332718	-3.739248	-0.079369
58	4.689914	-1.366312	-1.997586	-5.661756	0.360042	-0.145508	4.558681	-1.11217	-2.25814
	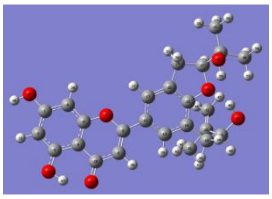 Conformer 7			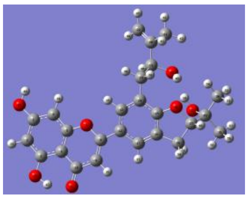 Conformer 8			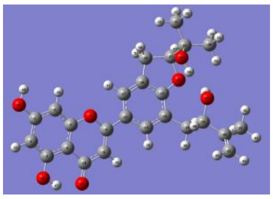 Conformer 9		
1	-5.867661	-1.88652	0.984674	5.746446	1.917636	0.423903	6.085161	-1.644369	0.69527
2	-6.690131	-0.907281	0.408881	6.622736	0.822777	0.409872	6.796865	-0.445554	0.543493
3	-6.112793	0.171597	-0.254549	6.117473	-0.457906	0.218511	6.120562	0.711899	0.176111
4	-4.698459	0.281396	-0.346312	4.719568	-0.658142	0.039305	4.714299	0.682163	-0.042982
5	-3.919142	-0.726605	0.246307	3.884542	0.469447	0.061139	4.048048	-0.542115	0.120147
6	-4.47177	-1.810515	0.91169	4.366581	1.758133	0.250962	4.702883	-1.711309	0.485296
7	-2.552381	-0.667495	0.193468	2.532701	0.337492	-0.100394	2.696196	-0.62953	-0.070426

8	-1.920188	0.352357	-0.449999	1.967289	-0.884314	-0.307293	1.969344	0.461556	-0.439814
9	-2.619503	1.349813	-1.061547	2.725589	-2.016417	-0.356286	2.559687	1.67514	-0.633362
10	-4.059912	1.386104	-1.036853	4.154217	-1.977753	-0.173003	3.973544	1.867502	-0.433576
11	-4.726265	2.303648	-1.585036	4.871833	-3.012404	-0.204831	4.537765	2.981815	-0.596126
12	-0.462071	0.216925	-0.402531	0.514276	-0.806572	-0.472015	0.547494	0.151291	-0.6013
13	0.363486	1.322546	-0.67703	-0.103237	0.433612	-0.715632	-0.40853	1.183044	-0.615622
14	1.759374	1.250346	-0.654463	-1.482866	0.560213	-0.898633	-1.772694	0.937301	-0.7834
15	2.285649	-0.005116	-0.343117	-2.220556	-0.622959	-0.835067	-2.138815	-0.401085	-0.946947
16	1.503067	-1.122615	-0.055547	-1.645546	-1.863825	-0.563577	-1.222732	-1.449842	-0.905414
17	0.123799	-1.024549	-0.08084	-0.280658	-1.969476	-0.381111	0.123647	-1.188157	-0.733253
18	2.61047	2.46948	-0.930829	-2.141542	1.904261	-1.078527	-2.795415	2.041875	-0.702728
19	3.619614	2.872424	0.186347	-2.799615	2.417431	0.221813	-3.505228	2.091154	0.668506
20	3.002212	2.77726	1.57249	-3.375451	3.8105	0.054698	-4.453401	3.269959	0.775886
21	1.78852	3.63859	1.810973	-4.611275	3.940553	-0.796311	-5.711183	3.212767	-0.050561
22	3.512311	1.979671	2.514108	-2.785738	4.854394	0.643822	-4.153613	4.29978	1.572194
23	4.850275	2.174372	0.062882	-3.8181	1.501899	0.676255	-4.204814	0.85566	0.926666
24	-6.89738	1.11074	-0.809811	6.952496	-1.51156	0.201617	6.797314	1.864049	0.02716
25	-6.383802	-2.955914	1.641369	6.307468	3.138624	0.614215	6.807561	-2.734881	1.056603
26	3.62517	-0.276504	-0.276993	-3.574499	-0.689021	-1.002347	-3.42401	-0.822685	-1.138155
27	3.801666	-1.617226	0.310717	-4.031313	-2.027278	-0.579547	-3.459446	-2.289631	-0.977248
28	2.419891	-2.295073	0.192245	-2.745251	-2.88884	-0.481463	-1.978336	-2.744826	-1.043804
29	4.95328	-2.294719	-0.446276	-4.818341	-1.855115	0.741519	-4.191295	-2.596207	0.351417
30	6.236048	-1.460176	-0.333439	-6.081839	-1.012561	0.506863	-5.653827	-2.130718	0.276237
31	5.171563	-3.70506	0.097593	-5.228623	-3.219354	1.300685	-4.162787	-4.098424	0.642618
32	4.584621	-2.44258	-1.823082	-3.970709	-1.251744	1.722521	-3.502556	-1.959774	1.430863
33	-7.77138	-0.977424	0.471559	7.686574	0.97725	0.546429	7.867076	-0.42453	0.712281
34	-3.846475	-2.571779	1.360838	3.684853	2.601413	0.263159	4.148376	-2.63588	0.601953
35	-2.100355	2.132548	-1.598297	2.265239	-2.975601	-0.552116	1.972401	2.522479	-0.961339
36	-0.089204	2.284792	-0.891764	0.50933	1.326631	-0.764068	-0.092367	2.209609	-0.461925
37	-0.500407	-1.883562	0.134243	0.164205	-2.929775	-0.144917	0.847647	-1.993718	-0.70223
38	3.202157	2.325294	-1.843012	-1.399163	2.642492	-1.396114	-2.31198	3.009127	-0.869121
39	1.939484	3.312459	-1.122186	-2.901155	1.854064	-1.868701	-3.54832	1.92099	-1.491645
40	3.874163	3.924682	0.001257	-2.039363	2.435307	1.009177	-2.742345	2.172565	1.449252
41	1.985428	4.682708	1.534189	-4.431799	3.60452	-1.825746	-6.295588	2.319866	0.200334
42	0.934782	3.306773	1.20785	-5.419724	3.317675	-0.396135	-5.48834	3.154868	-1.123798
43	1.486018	3.61311	2.861738	-4.958581	4.976569	-0.834897	-6.336092	4.094074	0.117474
44	3.06352	1.913021	3.501676	-1.903105	4.732758	1.267499	-4.800664	5.169926	1.649848
45	4.398552	1.38112	2.331204	-3.160146	5.867493	0.520138	-3.246068	4.311122	2.171359
46	4.631183	1.22389	0.054935	-4.145502	1.024318	-0.105286	-4.423027	0.462127	0.064326
47	-6.271554	1.774212	-1.226774	6.372634	-2.314654	0.048143	6.113833	2.547644	-0.238222
48	-7.354165	-2.907387	1.632697	5.616807	3.821819	0.603015	6.220298	-3.50466	1.137146
49	4.07681	-1.456148	1.35804	-4.70123	-2.375577	-1.368026	-4.044917	-2.66661	-1.818033
50	2.406706	-2.983069	-0.660462	-2.691875	-3.612017	-1.301967	-1.729727	-3.442339	-0.241709
51	2.168709	-2.858711	1.093875	-2.701034	-3.448259	0.455017	-1.7623	-3.243628	-1.99455

52	6.10001	-0.468818	-0.7775	-6.585921	-0.844918	1.463285	-5.74488	-1.070238	0.027581
53	6.53294	-1.328686	0.712747	-6.775061	-1.530627	-0.164168	-6.134245	-2.293077	1.245665
54	7.048338	-1.964669	-0.864941	-5.860959	-0.037089	0.065832	-6.203512	-2.697722	-0.482432
55	4.27395	-4.318098	-0.024494	-4.359705	-3.797229	1.625271	-3.149648	-4.444308	0.862184
56	5.990589	-4.183671	-0.447522	-5.776024	-3.801068	0.551875	-4.78685	-4.311049	1.515749
57	5.433875	-3.674555	1.159697	-5.87916	-3.074781	2.168405	-4.551398	-4.669726	-0.206674
58	4.408515	-1.546487	-2.158129	-3.820831	-0.317679	1.470862	-3.642403	-0.993302	1.360848
	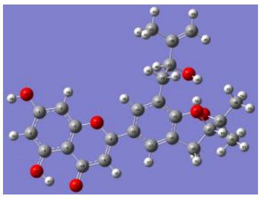 Conformer 10			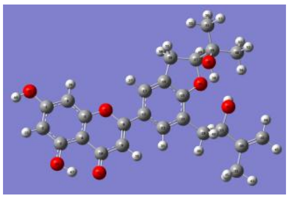 Conformer 11			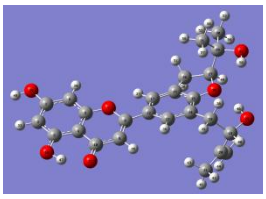 Conformer 12		
1	5.721531	1.872185	0.466008	6.241652	-1.595017	-0.049763	-5.866328	-2.009208	0.391321
2	6.592247	0.772909	0.447285	6.858022	-0.37214	0.252783	-6.64204	-0.871408	0.125657
3	6.080444	-0.505249	0.242182	6.082438	0.777852	0.369308	-6.013407	0.337574	-0.158517
4	4.6843	-0.695314	0.054498	4.675001	0.713412	0.181156	-4.594206	0.417956	-0.181629
5	3.855062	0.438589	0.080903	4.106212	-0.535597	-0.119296	-3.86327	-0.750694	0.091323
6	4.342017	1.721261	0.283175	4.85847	-1.694471	-0.240142	-4.467303	-1.965621	0.37748
7	2.502886	0.312865	-0.089035	2.756934	-0.653515	-0.315888	-2.494486	-0.731904	0.075641
8	1.933282	-0.904792	-0.307121	1.93384	0.425942	-0.20447	-1.81307	0.418884	-0.179213
9	2.68607	-2.040724	-0.36026	2.423419	1.661615	0.10132	-2.463339	1.590127	-0.430555
10	4.113677	-2.010465	-0.170065	3.831591	1.887923	0.301912	-3.902469	1.661934	-0.463026
11	4.827052	-3.048234	-0.206007	4.306085	3.022708	0.575035	-4.524336	2.72996	-0.705646
12	0.481731	-0.81924	-0.478783	0.530542	0.076158	-0.435121	-0.361765	0.224956	-0.128593
13	-0.128644	0.424963	-0.719279	-0.431362	1.080573	-0.647343	0.50297	1.174296	-0.70416
14	-1.506622	0.560052	-0.908801	-1.781526	0.793749	-0.857779	1.894153	1.049883	-0.66832
15	-2.251843	-0.619052	-0.855234	-2.128023	-0.559721	-0.863549	2.374721	-0.091397	-0.02278
16	-1.682972	-1.864638	-0.588483	-1.204406	-1.579476	-0.641241	1.552907	-1.060788	0.548447
17	-0.319892	-1.978538	-0.399507	0.127678	-1.276653	-0.43191	0.178234	-0.914951	0.501038
18	-2.152006	1.909562	-1.089611	-2.809646	1.885224	-1.004304	2.795218	2.080372	-1.309167
19	-2.807173	2.435822	0.209812	-3.605078	2.131107	0.300271	3.813788	2.781101	-0.360827
20	-3.367643	3.835718	0.025413	-4.571089	3.294703	0.155757	3.17597	3.172029	0.962605
21	-2.353788	4.91788	-0.249918	-3.941915	4.646708	-0.07043	2.013702	4.12761	0.873072
22	-4.676732	4.082222	0.114309	-5.892317	3.121011	0.237436	3.623214	2.693096	2.125794
23	-3.796216	1.51151	0.6795	-4.26102	0.927756	0.718103	5.010619	2.029517	-0.218757
24	6.91128	-1.561442	0.221395	6.668372	1.951575	0.661289	-6.752767	1.430952	-0.410785
25	6.173079	3.136728	0.662571	6.955703	-2.742446	-0.173314	-6.433426	-3.208853	0.67467
26	-3.603992	-0.678242	-1.029306	-3.394889	-1.023297	-1.069634	3.702866	-0.384501	0.108626
27	-4.068017	-2.014951	-0.612751	-3.425828	-2.454907	-0.715325	3.840758	-1.735102	0.683178
28	-2.787721	-2.885295	-0.520828	-1.939844	-2.893454	-0.650129	2.422843	-2.131973	1.160767
29	-4.849752	-1.841661	0.711225	-4.217513	-2.586323	0.607889	4.484433	-2.622248	-0.399283
30	-6.106973	-0.988618	0.48123	-5.677716	-2.155137	0.400747	4.873473	-3.970002	0.203219
31	-5.268835	-3.203202	1.269892	-4.192929	-4.032024	1.108607	3.584684	-2.791297	-1.62933

32	-3.994789	-1.246473	1.690736	-3.585677	-1.795647	1.617973	5.715786	-1.994971	-0.788565
33	7.660364	0.90314	0.590096	7.931584	-0.308642	0.39926	-7.726431	-0.918613	0.138957
34	3.679139	2.577171	0.300446	4.393452	-2.643809	-0.474231	-3.878575	-2.850771	0.582783
35	2.221501	-2.99598	-0.565101	1.754137	2.503601	0.215963	-1.904836	2.502061	-0.59437
36	0.488908	1.315014	-0.760104	-0.128169	2.122217	-0.660355	0.084767	2.028548	-1.226046
37	0.118782	-2.942704	-0.167362	0.854154	-2.060202	-0.252846	-0.476473	-1.654407	0.94632
38	-1.399744	2.627618	-1.426086	-2.312942	2.812824	-1.30035	3.38461	1.62491	-2.114321
39	-2.926317	1.871674	-1.867164	-3.528273	1.641433	-1.797706	2.16243	2.842924	-1.77315
40	-2.038859	2.466037	0.993393	-2.889656	2.366364	1.098994	4.122733	3.702288	-0.872738
41	-1.536506	4.88792	0.48266	-3.154604	4.843941	0.668978	1.697471	4.459332	1.866124
42	-1.8932	4.804951	-1.23941	-3.470224	4.718474	-1.058494	2.276548	5.014018	0.280841
43	-2.815572	5.908238	-0.20844	-4.687372	5.44352	0.001467	1.148287	3.665054	0.38308
44	-5.070492	5.086718	-0.01604	-6.324443	2.142962	0.419486	3.15811	2.971711	3.067739
45	-5.387682	3.292533	0.331985	-6.577396	3.958598	0.136651	4.472094	2.019102	2.172997
46	-4.205416	1.108337	-0.104826	-4.529762	0.454466	-0.087617	4.755892	1.134941	0.073439
47	6.32796	-2.360972	0.058734	5.92443	2.623509	0.696127	-6.095469	2.168878	-0.581137
48	7.137861	3.124478	0.776515	7.897775	-2.558461	-0.022663	-7.401142	-3.124146	0.658566
49	-4.742754	-2.355794	-1.400439	-3.967798	-2.95109	-1.522703	4.547637	-1.628845	1.507393
50	-2.749486	-3.456736	0.408611	-1.671426	-3.504794	-1.51834	2.359756	-2.121247	2.253807
51	-2.735704	-3.598461	-1.350262	-1.726343	-3.480651	0.245081	2.145433	-3.136937	0.830942
52	-5.879365	-0.020305	0.027764	-6.201285	-2.17928	1.361057	3.992238	-4.530059	0.529196
53	-6.811504	-1.504996	-0.179299	-6.189064	-2.833211	-0.290646	5.400823	-4.566269	-0.547389
54	-6.600649	-0.806927	1.440455	-5.761965	-1.143969	-0.00597	5.537531	-3.829755	1.062329
55	-4.403329	-3.787737	1.591697	-3.188294	-4.332008	1.416839	4.112991	-3.387413	-2.378956
56	-5.916184	-3.054944	2.139444	-4.541508	-4.721253	0.3326	2.645601	-3.298622	-1.388194
57	-5.822391	-3.78	0.521814	-4.852861	-4.127007	1.976139	3.343967	-1.819662	-2.07301
58	-3.824273	-0.318492	1.430352	-3.714252	-0.850956	1.396446	5.476735	-1.119404	-1.137677
	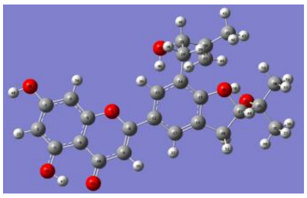 Conformer 13			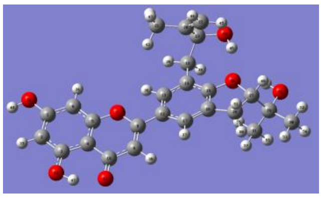 Conformer 14			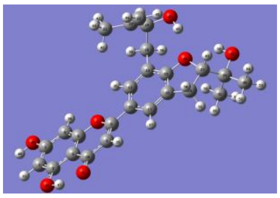 Conformer 15		
1	5.905009	1.702551	0.155331	-5.878521	1.426954	-0.554247	-5.846	1.176588	-1.267434
2	6.690292	0.546499	0.272984	-6.609234	0.26107	-0.28263	-6.606346	0.216674	-0.583612
3	6.080671	-0.704459	0.237276	-5.93447	-0.915321	0.031024	-5.961887	-0.76292	0.166541
4	4.671359	-0.809582	0.083248	-4.513772	-0.934487	0.077849	-4.542366	-0.791515	0.237302
5	3.930649	0.378789	-0.032865	-3.828999	0.260037	-0.20241	-3.827279	0.192286	-0.466135
6	4.515495	1.635628	0.000332	-4.479296	1.443464	-0.517713	-4.447036	1.178065	-1.219046
7	2.570231	0.336534	-0.177854	-2.461328	0.300109	-0.165073	-2.45906	0.207944	-0.437702
8	1.908199	-0.852072	-0.232524	-1.735229	-0.815443	0.121224	-1.761446	-0.71008	0.286278
9	2.570989	-2.040048	-0.141607	-2.339165	-2.008603	0.384714	-2.397245	-1.679279	1.003733
10	4.000068	-2.095199	0.034651	-3.774258	-2.142403	0.392765	-3.834163	-1.790368	1.015383
11	4.632466	-3.180502	0.129109	-4.353861	-3.232059	0.643614	-4.441273	-2.684058	1.66275

12	0.464867	-0.677779	-0.403859	-0.292577	-0.561377	0.096187	-0.313048	-0.506171	0.195864
13	-0.04975	0.557832	-0.836755	0.205386	0.599153	-0.524234	0.196009	0.698066	-0.324828
14	-1.418795	0.776528	-1.034571	1.570302	0.895138	-0.591587	1.565843	0.960448	-0.426213
15	-2.255933	-0.312895	-0.78054	2.414309	-0.04372	0.002372	2.403716	-0.060773	0.022601
16	-1.777274	-1.548407	-0.335557	1.960834	-1.203046	0.631356	1.94005	-1.270861	0.538264
17	-0.42393	-1.740371	-0.137526	0.606437	-1.471949	0.688488	0.581514	-1.50679	0.628864
18	-1.942946	2.130773	-1.43373	2.071662	2.148446	-1.271375	2.077766	2.263703	-0.995649
19	-2.207326	3.043454	-0.2004	2.888989	3.128132	-0.376966	2.988641	3.11108	-0.057619
20	-3.441446	2.636585	0.589159	2.251759	3.309198	0.991387	2.445606	3.163665	1.361381
21	-4.757224	2.851246	-0.112029	0.851501	3.867421	0.994364	1.070981	3.762587	1.515369
22	-3.361191	2.168554	1.839518	2.898963	2.989918	2.114588	3.14873	2.705425	2.399624
23	-1.034131	3.156152	0.594022	4.264236	2.77637	-0.324797	4.349073	2.709553	-0.134346
24	6.829271	-1.814962	0.348491	-6.630718	-2.03527	0.289502	-6.686293	-1.68315	0.825631
25	6.453153	2.943398	0.186076	-6.491438	2.596795	-0.865577	-6.42821	2.151399	-2.010232
26	-3.60506	-0.284423	-0.93138	3.775583	0.073208	0.037211	3.768124	0.008208	-0.005176
27	-4.143328	-1.62465	-0.636561	4.348492	-1.16515	0.595615	4.322901	-1.30469	0.370679
28	-2.958076	-2.446218	-0.074476	3.150978	-1.949721	1.183465	3.128049	-2.11961	0.921779
29	-5.322752	-1.429421	0.331425	5.139554	-1.848916	-0.535819	5.033738	-1.868208	-0.87449
30	-6.423008	-0.58623	-0.325351	5.951532	-3.010311	0.032364	5.830784	-3.113417	-0.493522
31	-5.884811	-2.780999	0.76929	4.238157	-2.301617	-1.690821	4.061909	-2.150481	-2.026574
32	-4.835661	-0.783032	1.513303	6.099822	-0.898364	-1.020686	6.002256	-0.893837	-1.291236
33	7.767546	0.611578	0.39035	-7.69424	0.261444	-0.313044	-7.690819	0.225811	-0.629559
34	3.919033	2.534828	-0.088815	-3.925229	2.349645	-0.727751	-3.870296	1.923468	-1.751964
35	2.03097	-2.974287	-0.219415	-1.745284	-2.891815	0.578704	-1.830324	-2.383515	1.597666
36	0.637596	1.370035	-1.043887	-0.495118	1.287593	-0.98261	-0.500375	1.455964	-0.664239
37	-0.057183	-2.68925	0.238478	0.246704	-2.357429	1.200602	0.217963	-2.454322	1.010271
38	-1.200713	2.636417	-2.058829	2.717423	1.891699	-2.120004	1.213775	2.867272	-1.289656
39	-2.858146	2.031295	-2.023444	1.207871	2.680658	-1.681038	2.659428	2.079139	-1.907237
40	-2.385519	4.055009	-0.586294	2.869187	4.098828	-0.890318	2.976734	4.132043	-0.462149
41	-4.868442	3.901546	-0.41145	0.792552	4.788373	0.399472	0.302896	3.133076	1.049978
42	-4.828454	2.251277	-1.02577	0.135067	3.161436	0.55708	1.012272	4.745527	1.02964
43	-5.599679	2.58812	0.53327	0.518913	4.093668	2.011454	0.809144	3.884611	2.570258
44	-4.253797	1.917584	2.407292	2.434985	3.116116	3.089283	2.751428	2.740796	3.41062
45	-2.410275	2.060527	2.350549	3.912733	2.604044	2.094909	4.143649	2.291809	2.272971
46	-0.74449	2.253488	0.812027	4.313319	1.845224	-0.040232	4.377682	1.753422	0.053768
47	6.185311	-2.581841	0.293809	-5.945529	-2.741438	0.482798	-6.019476	-2.27092	1.289746
48	7.415403	2.871899	0.299144	-7.454848	2.470909	-0.865111	-7.394436	2.05744	-1.972508
49	-4.497676	-2.02862	-1.589586	5.05381	-0.83877	1.361197	5.075316	-1.094749	1.13227
50	-3.079282	-2.609521	1.001355	3.163169	-3.001366	0.884593	3.200474	-2.235566	2.008116
51	-2.876088	-3.422094	-0.559925	3.164886	-1.92399	2.278052	3.083368	-3.123915	0.491885
52	-6.035412	0.38206	-0.649337	6.605359	-2.663965	0.839369	5.172405	-3.923663	-0.167183
53	-6.840367	-1.097696	-1.199703	5.300981	-3.798634	0.421862	6.399262	-3.461442	-1.361149
54	-7.22939	-0.415181	0.394346	6.575353	-3.439262	-0.757494	6.535358	-2.887182	0.313354
55	-5.132783	-3.376702	1.293778	3.689662	-1.453648	-2.113956	3.325163	-2.917016	-1.767341

56	-6.727538	-2.621321	1.448946	4.859769	-2.733946	-2.480188	3.523006	-1.24193	-2.314793
57	-6.242266	-3.34977	-0.094984	3.51066	-3.056045	-1.376098	4.628954	-2.500465	-2.893942
58	-4.389363	0.034631	1.224789	5.58947	-0.136829	-1.344848	5.503982	-0.08471	-1.497561
		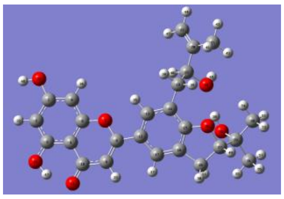 Conformer 16			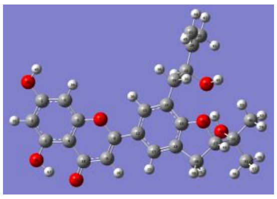 Conformer 17			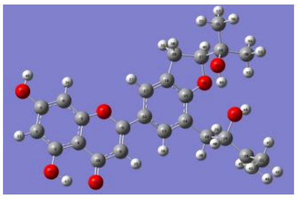 Conformer 18	
1	5.665874	1.910179	0.383424	5.839086	1.783662	-0.251862	6.238544	-1.56905	0.096298
2	6.546686	0.819111	0.405612	6.635754	0.713894	0.181238	6.844656	-0.324838	0.322767
3	6.047761	-0.469934	0.240212	6.049786	-0.523269	0.42331	6.062973	0.824319	0.348058
4	4.654658	-0.67906	0.051363	4.651079	-0.704208	0.231959	4.656981	0.739925	0.143488
5	3.814863	0.447039	0.035066	3.897509	0.396515	-0.202332	4.098188	-0.527676	-0.078705
6	4.28903	1.740387	0.197449	4.460962	1.641737	-0.450617	4.85909	-1.68963	-0.107836
7	2.465087	0.303079	-0.137724	2.550778	0.280193	-0.408338	2.7539	-0.668695	-0.2869
8	1.906806	-0.926874	-0.316435	1.902958	-0.896941	-0.180257	1.921829	0.410427	-0.263605
9	2.671872	-2.05688	-0.327415	2.578028	-1.999673	0.256248	2.402187	1.667062	-0.035825
10	4.097167	-2.006591	-0.131722	3.999805	-1.977168	0.481369	3.805156	1.914834	0.170873
11	4.821734	-3.037861	-0.129613	4.642542	-2.986513	0.876489	4.269984	3.068511	0.373451
12	0.456677	-0.862024	-0.496311	0.46757	-0.803489	-0.448302	0.52574	0.036525	-0.493216
13	-0.165024	0.366017	-0.789425	-0.131838	0.453079	-0.653541	-0.441556	1.019588	-0.773051
14	-1.543343	0.474553	-0.990858	-1.498317	0.59987	-0.905996	-1.785693	0.709021	-0.987635
15	-2.272809	-0.710402	-0.906828	-2.241774	-0.578147	-0.969459	-2.120119	-0.645272	-0.935012
16	-1.696593	-1.937316	-0.565961	-1.686154	-1.839513	-0.73861	-1.193065	-1.643759	-0.631285
17	-0.334606	-2.026437	-0.363537	-0.335881	-1.965831	-0.484771	0.131862	-1.318134	-0.415978
18	-2.231576	1.80137	-1.175404	-2.150679	1.954127	-0.993762	-2.830538	1.778391	-1.165766
19	-2.571235	2.460716	0.172777	-2.655857	2.420239	0.383607	-3.459371	2.173402	0.18268
20	-3.17631	3.840357	0.01667	-3.1914	3.839703	0.440065	-4.412668	3.354148	0.140806
21	-4.473498	3.940396	-0.743522	-3.481927	4.345039	1.830555	-4.857857	3.847879	1.494059
22	-2.564941	4.899535	0.555822	-3.420595	4.57623	-0.651324	-4.850581	3.898092	-0.998649
23	-3.492926	1.589301	0.863966	-3.662253	1.521142	0.907225	-4.133608	1.05009	0.797686
24	6.888102	-1.518663	0.259241	6.806988	-1.553079	0.840628	6.637002	2.020357	0.567362
25	6.104856	3.184774	0.541646	6.474806	2.963233	-0.46853	7.060725	-2.649006	0.085333
26	-3.612366	-0.786491	-1.102943	-3.570802	-0.622609	-1.241965	-3.371222	-1.125051	-1.153453
27	-4.07912	-2.073513	-0.574304	-4.061962	-1.968738	-0.915875	-3.409277	-2.525813	-0.709762
28	-2.805526	-2.951281	-0.440941	-2.79527	-2.857494	-0.804644	-1.92616	-2.959005	-0.570003
29	-4.815087	-1.764698	0.74994	-4.897226	-1.831896	0.377946	-4.237931	-2.551582	0.595215
30	-5.989165	-0.811402	0.484512	-6.093773	-0.903206	0.122869	-5.669861	-2.077042	0.305811
31	-5.318407	-3.051762	1.402278	-5.397722	-3.196329	0.852259	-4.273481	-3.958174	1.192979
32	-3.893803	-1.165167	1.665008	-4.066836	-1.314164	1.419599	-3.610914	-1.70912	1.564762
33	7.612676	0.964104	0.549712	7.700472	0.853729	0.326558	7.915329	-0.262787	0.478036
34	3.618166	2.590124	0.18285	3.841572	2.465745	-0.787346	4.386025	-2.649584	-0.283788
35	2.217067	-3.022595	-0.503213	2.050107	-2.922309	0.456175	1.727833	2.511614	0.00907

36	0.443194	1.261233	-0.8565	0.482559	1.34459	-0.596642	-0.147536	2.062452	-0.83251
37	0.114697	-2.971124	-0.077122	0.097064	-2.947173	-0.325455	0.86098	-2.083196	-0.177191
38	-1.584514	2.493178	-1.724696	-1.428266	2.688667	-1.363396	-2.37418	2.669342	-1.608468
39	-3.150698	1.672298	-1.753188	-2.987795	1.930348	-1.699728	-3.61681	1.437061	-1.847709
40	-1.645442	2.53888	0.758562	-1.830109	2.327451	1.098544	-2.648001	2.401959	0.883468
41	-4.333401	3.699064	-1.804787	-4.170597	3.670258	2.351124	-4.000711	4.215668	2.073919
42	-4.893636	4.947837	-0.678979	-2.562392	4.381248	2.429983	-5.304704	3.03342	2.074985
43	-5.207043	3.227338	-0.351164	-3.91962	5.346988	1.810836	-5.587022	4.658094	1.406194
44	-2.972416	5.903162	0.462904	-3.82623	5.581572	-0.571999	-5.549807	4.730275	-0.988005
45	-1.63147	4.800033	1.104668	-3.213879	4.219827	-1.65514	-4.534786	3.549179	-1.976356
46	-3.713834	2.019587	1.706454	-4.462202	1.660746	0.373193	-4.925174	0.8755	0.261378
47	6.312015	-2.328253	0.11971	6.176709	-2.325377	0.949618	5.888856	2.687696	0.54483
48	7.068759	3.184846	0.663612	5.832622	3.631489	-0.760021	6.541238	-3.453744	-0.076679
49	-4.782428	-2.469391	-1.310061	-4.706327	-2.267222	-1.745577	-3.93068	-3.079723	-1.493359
50	-2.76104	-3.706757	-1.232961	-2.696405	-3.515186	-1.674944	-1.741891	-3.480265	0.371926
51	-2.769825	-3.47328	0.517745	-2.817821	-3.488861	0.086153	-1.631853	-3.630832	-1.383486
52	-6.498556	-0.59155	1.428251	-6.613148	-0.709125	1.066081	-5.670358	-1.112087	-0.208752
53	-6.715245	-1.257242	-0.204455	-5.770508	0.049799	-0.305074	-6.218796	-1.976018	1.246829
54	-5.630083	0.127187	0.055477	-6.802787	-1.358044	-0.577363	-6.200528	-2.792833	-0.331369
55	-4.492079	-3.706665	1.692441	-6.069894	-3.062318	1.705413	-3.284216	-4.27056	1.537895
56	-5.97616	-3.602156	0.721476	-4.570997	-3.835737	1.173054	-4.949687	-3.972314	2.053338
57	-5.885186	-2.804864	2.30524	-5.94785	-3.709025	0.056423	-4.63354	-4.686177	0.45863
58	-3.673917	-0.275684	1.316599	-3.839034	-0.386852	1.198234	-3.691191	-0.780669	1.260996
	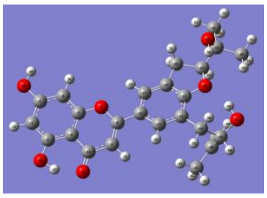 Conformer 19			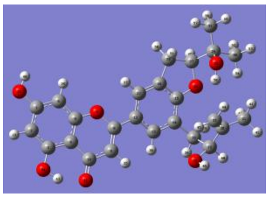 Conformer 20			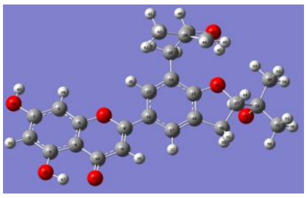 Conformer 21		
1	-5.924775	-2.112359	0.276789	-6.00311	2.020847	0.141503	-6.004426	1.257501	-1.052863
2	-6.718896	-0.961999	0.163171	-6.744163	0.840522	0.297834	-6.733124	0.239345	-0.421068
3	-6.112298	0.274495	-0.024601	-6.092578	-0.387141	0.270294	-6.056732	-0.800576	0.205849
4	-4.69428	0.373559	-0.103037	-4.682882	-0.447319	0.082551	-4.633553	-0.833894	0.205541
5	-3.944286	-0.806429	0.01754	-3.986442	0.761449	-0.067277	-3.950566	0.208821	-0.439132
6	-4.528199	-2.05227	0.206115	-4.615834	1.999548	-0.042262	-4.604801	1.25838	-1.07096
7	-2.578606	-0.773571	-0.056188	-2.631801	0.765554	-0.256662	-2.583235	0.226288	-0.476855
8	-1.915061	0.403534	-0.225723	-1.924071	-0.398704	-0.282499	-1.853163	-0.752866	0.125565
9	-2.583449	1.586668	-0.33028	-2.539922	-1.605574	-0.123832	-2.455731	-1.784094	0.7823
10	-4.023117	1.646643	-0.289535	-3.964875	-1.7088	0.055602	-3.890725	-1.898211	0.854496
11	-4.661216	2.727114	-0.39472	-4.55581	-2.813092	0.193814	-4.467595	-2.847549	1.447622
12	-0.461949	0.218899	-0.259792	-0.491545	-0.172099	-0.476259	-0.410542	-0.540048	-0.016218
13	0.371244	1.229217	-0.77425	0.346935	-1.227378	-0.878284	0.079015	0.717302	-0.414724
14	1.764295	1.119084	-0.80453	1.721685	-1.063939	-1.05765	1.44461	0.992913	-0.542556
15	2.278829	-0.075311	-0.29626	2.220441	0.21922	-0.828386	2.296692	-0.074219	-0.25787

16	1.488146	-1.105448	0.210184	1.420004	1.288875	-0.422554	1.851833	-1.338415	0.130827
17	0.111566	-0.972463	0.229737	0.06201	1.106316	-0.24418	0.49772	-1.585348	0.251541
18	2.628854	2.231847	-1.352332	2.622984	-2.211548	-1.425502	1.933098	2.365648	-0.946955
19	3.678954	2.826504	-0.365837	3.06788	-3.043288	-0.199599	2.892413	3.073255	0.057039
20	3.096577	3.032452	1.023406	3.894635	-2.242972	0.791889	2.425664	2.91332	1.495066
21	1.920978	3.971885	1.109184	5.350975	-2.089612	0.442869	1.061349	3.472509	1.807646
22	3.600819	2.41442	2.094163	3.352244	-1.732443	1.903383	3.18389	2.31335	2.415847
23	4.889241	2.083172	-0.37117	1.8803	-3.586024	0.367952	4.24562	2.693064	-0.147493
24	-6.867861	1.381319	-0.132248	-6.796717	-1.522604	0.421739	-6.749385	-1.778774	0.814934
25	-6.581408	-3.285925	0.458415	-6.702147	3.183826	0.177592	-6.727981	2.240691	-1.645486
26	3.614317	-0.367378	-0.242403	3.531503	0.552819	-0.968451	3.661094	-0.003287	-0.331804
27	3.79243	-1.598443	0.549851	3.684672	1.992651	-0.701821	4.225871	-1.234485	0.250699
28	2.391285	-2.243678	0.616715	2.311492	2.479166	-0.176065	3.053023	-2.237294	0.290366
29	4.884119	-2.426964	-0.143166	4.859235	2.131061	0.281914	5.427893	-1.634793	-0.617042
30	6.189584	-1.625943	-0.2358	6.151129	1.599964	-0.353212	6.466983	-0.506144	-0.648792
31	5.10039	-3.735349	0.614199	5.038275	3.586039	0.710094	6.043528	-2.926976	-0.084383
32	4.435393	-2.785308	-1.455976	4.544757	1.385428	1.465642	4.971551	-1.918907	-1.945281
33	-7.798171	-1.039301	0.222134	-7.817509	0.887911	0.439246	-7.816428	0.265807	-0.423083
34	-3.910073	-2.93894	0.292586	-4.038205	2.909123	-0.163439	-4.037273	2.043655	-1.558039
35	-2.038625	2.516135	-0.428785	-1.957731	-2.517375	-0.110164	-1.861174	-2.537152	1.281957
36	-0.07389	2.130241	-1.182885	-0.073822	-2.20757	-1.069213	-0.628667	1.512291	-0.619847
37	-0.519	-1.760467	0.623689	-0.571811	1.922818	0.081339	0.145624	-2.572274	0.530214
38	3.189262	1.887714	-2.229994	2.102605	-2.892981	-2.106448	2.467851	2.317158	-1.903313
39	1.969966	3.035682	-1.694315	3.513846	-1.845067	-1.943296	1.057504	3.001769	-1.108088
40	3.9524	3.811203	-0.768106	3.70354	-3.857574	-0.5843	2.860533	4.14248	-0.192011
41	1.643304	4.163688	2.149544	5.864996	-1.423987	1.14195	0.978354	4.516485	1.477746
42	2.148904	4.933268	0.630252	5.858783	-3.062673	0.454422	0.269917	2.914052	1.293225
43	1.041022	3.563988	0.597044	5.473268	-1.681674	-0.567465	0.854618	3.433973	2.880977
44	3.175232	2.560697	3.083443	3.949399	-1.186712	2.629589	2.84182	2.196658	3.440804
45	4.459365	1.755535	2.016918	2.298153	-1.860857	2.126847	4.171577	1.933253	2.176807
46	4.649367	1.154112	-0.195564	2.136073	-4.042922	1.185596	4.271607	1.718693	-0.11145
47	-6.22359	2.138752	-0.256889	-6.125448	-2.265808	0.370788	-6.062377	-2.404185	1.190901
48	-5.940134	-4.013224	0.521208	-6.096394	3.933418	0.054511	-6.128849	2.8875	-2.053657
49	4.130021	-1.276393	1.540153	3.936838	2.459064	-1.658919	4.556882	-0.969751	1.260019
50	2.174883	-2.63398	1.614022	1.980854	3.384913	-0.69113	3.125021	-2.938495	-0.548526
51	2.318414	-3.069022	-0.100246	2.364559	2.698147	0.895591	3.044897	-2.811309	1.219884
52	6.549972	-1.339277	0.758056	6.434431	2.198213	-1.226234	6.043517	0.405733	-1.08207
53	6.957758	-2.234036	-0.722613	6.96328	1.647143	0.378644	6.8268	-0.27083	0.358704
54	6.049272	-0.71399	-0.825077	6.03181	0.562387	-0.674254	7.320606	-0.81206	-1.260773
55	5.877288	-4.322326	0.115231	5.882202	3.661284	1.402826	5.319731	-3.746789	-0.105882
56	5.421453	-3.538621	1.641796	5.247186	4.221059	-0.156726	6.898453	-3.207761	-0.706704
57	4.183966	-4.331949	0.641065	4.147036	3.966592	1.21638	6.392807	-2.79424	0.944295
58	4.262201	-1.950178	-1.923808	4.276345	0.496823	1.169549	4.544794	-1.106748	-2.2685

	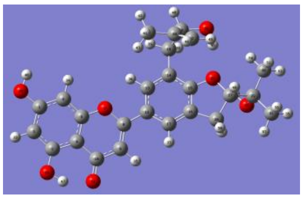<br>Conformer 22		
1	-5.973692	1.487389	-0.293528
2	-6.709332	0.310996	-0.088747
3	-6.040607	-0.895366	0.082977
4	-4.617956	-0.937629	0.053167
5	-3.92804	0.266232	-0.156643
6	-4.574494	1.482907	-0.331551
7	-2.560673	0.288111	-0.188614
8	-1.838381	-0.856538	-0.037534
9	-2.448134	-2.060441	0.152575
10	-3.883171	-2.177483	0.222665
11	-4.466762	-3.277966	0.407091
12	-0.394902	-0.617843	-0.114423
13	0.089755	0.584674	-0.660238
14	1.454347	0.875852	-0.759688
15	2.311211	-0.115781	-0.281888
16	1.871365	-1.320234	0.268415
17	0.517724	-1.583544	0.35817
18	1.935456	2.189034	-1.334459
19	2.817864	3.062846	-0.392815
20	2.270351	3.098418	1.025242
21	0.876884	3.652595	1.177608
22	2.987043	2.663555	2.064489
23	4.188667	2.697753	-0.464241
24	-6.740289	-2.026778	0.277914
25	-6.690315	2.628745	-0.452191
26	3.675741	-0.013797	-0.286818
27	4.230356	-1.121083	0.513641
28	3.079639	-2.141773	0.644032
29	5.491563	-1.61555	-0.209492
30	6.504548	-0.473672	-0.364586
31	6.103102	-2.785486	0.558395
32	5.123177	-2.122409	-1.498157
33	-7.792169	0.345209	-0.064909
34	-4.000418	2.389246	-0.488968
35	-1.85966	-2.964127	0.239187
36	-0.621629	1.318533	-1.021156
37	0.169386	-2.506648	0.807974
38	2.526438	2.019106	-2.242596

39	1.056903	2.768245	-1.634414
40	2.777595	4.083449	-0.796669
41	0.610687	3.767874	2.232189
42	0.786696	4.632358	0.690259
43	0.129901	2.997581	0.713005
44	2.587996	2.686224	3.075145
45	3.995856	2.28473	1.938384
46	4.233222	1.739589	-0.286597
47	-6.058143	-2.754866	0.372244
48	-6.087117	3.379188	-0.582513
49	4.493037	-0.691164	1.485499
50	3.023755	-2.559853	1.651902
51	3.221376	-2.965364	-0.064542
52	6.797235	-0.069228	0.610389
53	7.400413	-0.845644	-0.870462
54	6.08696	0.342921	-0.962378
55	6.999567	-3.135137	0.03771
56	6.387655	-2.478203	1.569513
57	5.400539	-3.620758	0.630248
58	4.702557	-1.384984	-1.973013

Table S8. Conformational analysis of (1''*S*,2''*S*,2'''*S*)-**4**

Conformers	Gibbs Free Energy (Hartree)	Relative Gibbs Free Energy (kcal/mol)	Population (%)
1	-1609.107226	1.41942762	2.85
2	-1609.106514	1.86621474	1.34
3	-1609.106517	1.86433221	1.34
4	-1609.107308	1.3679718	3.10
5	-1609.109488	0	31.23
6	-1609.107348	1.3428714	3.24
7	-1609.107299	1.37361939	3.07
8	-1609.107838	1.0353915	5.44
9	-1609.106605	1.80911133	1.47
10	-1609.1094	0.05522088	28.45
11	-1609.10684	1.66164648	1.89
12	-1609.106813	1.67858925	1.84
13	-1609.107776	1.07429712	5.10
14	-1609.107	1.56124488	2.24
15	-1609.106406	1.93398582	1.19
16	-1609.107111	1.49159127	2.52
17	-1609.106557	1.83923181	1.40
18	-1609.107016	1.55120472	2.28

Table S9. Coordinates of (1''*S*,2''*S*,2'''*S*)-**4**

	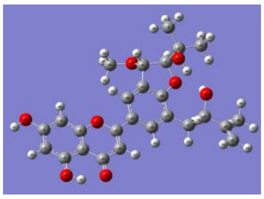 Conformer 1			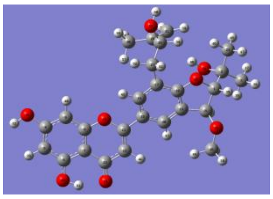 Conformer 2			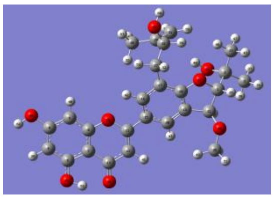 Conformer 3		
1	-3.386994	-2.172264	-0.281324	-4.166527	-1.234642	-0.353374	-4.166542	-1.234589	-0.353318
2	-4.092091	-2.131043	1.094957	-4.91637	-0.837316	0.930893	-4.916411	-0.837247	0.930926
3	-4.021979	-3.500364	1.775568	-5.597043	-2.057985	1.550534	-5.597128	-2.057911	1.550534
4	6.287522	-1.406414	0.152918	5.978712	1.684748	0.126762	5.978681	1.684727	0.127034
5	6.922279	-0.168381	0.330109	6.723305	0.515099	0.337375	6.723282	0.515057	0.337489
6	6.162796	0.998087	0.339149	6.075361	-0.716614	0.363404	6.07535	-0.716666	0.363354
7	4.752915	0.935094	0.168511	4.667468	-0.788403	0.180139	4.66746	-0.788444	0.180049
8	4.165702	-0.329698	-0.004083	3.967894	0.412489	-0.027013	3.967876	0.412471	-0.02695
9	4.901472	-1.504964	-0.016502	4.591525	1.65057	-0.05762	4.591497	1.650563	-0.057378
10	2.813216	-0.447742	-0.178765	2.610204	0.40253	-0.201947	2.610188	0.402524	-0.201908
11	2.006641	0.649154	-0.168535	1.913089	-0.766886	-0.202243	1.913082	-0.766898	-0.202367
12	2.512702	1.901771	0.011368	2.535653	-1.966768	-0.026689	2.535653	-1.9668	-0.026969
13	3.92628	2.127292	0.179911	3.957606	-2.053941	0.190875	3.957608	-2.053991	0.190582
14	4.416625	3.276518	0.337906	4.552595	-3.149959	0.367146	4.5526	-3.150027	0.36673
15	0.596894	0.296315	-0.356292	0.479325	-0.560071	-0.414688	0.479317	-0.560074	-0.414779
16	-0.351962	1.28291	-0.684307	0.009878	0.665417	-0.923837	0.009867	0.665402	-0.923951
17	-1.708153	0.997757	-0.850737	-1.345581	0.931581	-1.132921	-1.345598	0.931567	-1.133012
18	-2.0739	-0.342074	-0.698282	-2.222167	-0.11193	-0.822385	-2.222184	-0.111918	-0.822402
19	-1.161805	-1.344154	-0.374511	-1.789871	-1.346816	-0.333714	-1.789887	-1.34679	-0.333692
20	0.175773	-1.039315	-0.19347	-0.445626	-1.576714	-0.104484	-0.445635	-1.576696	-0.104505
21	-1.917264	-2.647458	-0.283879	-3.001142	-2.228214	-0.147061	-3.001165	-2.228156	-0.146929
22	-3.354559	-0.800923	-0.810824	-3.573698	-0.022082	-0.928791	-3.573714	-0.022044	-0.92876
23	-5.565972	-1.734236	0.916708	-5.959739	0.244974	0.626367	-5.95974	0.245079	0.626402
24	-3.411639	-1.214425	1.954841	-3.968311	-0.369903	1.900836	-3.968363	-0.369873	1.900906
25	-1.758178	-3.478202	-1.434222	-3.137796	-3.23659	-1.147021	-3.137888	-3.236662	-1.146756
26	-0.472124	-4.069492	-1.534701	-2.202713	-4.296217	-1.021706	-2.202888	-4.296346	-1.021295
27	-2.72574	2.080804	-1.100135	-1.812642	2.267793	-1.651437	-1.812663	2.267765	-1.651546
28	-3.479081	2.484717	0.187237	-2.783976	3.02244	-0.727162	-2.783912	3.022488	-0.727227
29	-4.438368	3.634373	-0.054093	-2.334124	3.113175	0.719658	-2.333952	3.113232	0.719555
30	-5.679085	3.332297	-0.852819	-0.935617	3.604339	0.980949	-0.935449	3.604485	0.980729
31	-4.161658	4.84944	0.42697	-3.199445	2.839337	1.704439	-3.199153	2.83926	1.704401
32	-4.174393	1.352382	0.749891	-2.886776	4.336093	-1.299093	-2.886703	4.336114	-1.2992
33	6.766771	2.186246	0.51059	6.785536	-1.840056	0.561901	6.785539	-1.840121	0.561725
34	6.985013	-2.57016	0.136634	6.565896	2.907458	0.091747	6.565863	2.907442	0.09218
35	-3.963658	-2.769106	-0.989882	-4.859599	-1.630549	-1.098247	-4.859589	-1.630526	-1.0982
36	-3.004427	-3.729172	2.101778	-6.295762	-2.513147	0.841945	-6.295973	-2.512934	0.84198
37	-4.361121	-4.293422	1.101101	-6.156724	-1.747805	2.437915	-6.156677	-1.747761	2.438009

38	-4.666333	-3.500541	2.659704	-4.869515	-2.814247	1.856984	-4.869643	-2.814281	1.856822
39	7.997788	-0.105289	0.461753	7.798674	0.555076	0.479774	7.798649	0.555031	0.479899
40	4.422106	-2.466056	-0.15351	4.026235	2.559999	-0.217838	4.026202	2.56001	-0.217473
41	1.854617	2.759573	0.047639	1.968916	-2.887631	-0.064174	1.968925	-2.887662	-0.064594
42	-0.032066	2.311042	-0.817763	0.729335	1.441259	-1.159391	0.729326	1.441225	-1.159564
43	0.889721	-1.802053	0.091121	-0.115105	-2.509405	0.337571	-0.115106	-2.509376	0.337569
44	-1.653818	-3.215838	0.61509	-3.019087	-2.690598	0.846808	-3.019109	-2.690404	0.847002
45	-6.111381	-2.50533	0.36263	-6.441878	0.557366	1.557509	-6.729603	-0.133872	-0.054146
46	-5.684304	-0.791749	0.375411	-5.498937	1.118875	0.158594	-5.498888	1.119007	0.15873
47	-6.028907	-1.621028	1.901398	-6.729652	-0.134043	-0.054084	-6.441951	0.557415	1.557525
48	-3.581173	-0.303769	1.636407	-3.573965	0.454124	1.563074	-3.573942	0.454116	1.563135
49	-0.515606	-4.777481	-2.36539	-1.180883	-3.97161	-1.258341	-1.181044	-3.971874	-1.258043
50	-0.207459	-4.608366	-0.612793	-2.502371	-5.066489	-1.735942	-2.212898	-4.719604	-0.005912
51	0.306722	-3.324197	-1.743041	-2.212742	-4.719659	-0.0064	-2.502654	-5.06673	-1.735368
52	-3.452626	1.754114	-1.853947	-0.941358	2.908223	-1.820193	-2.312082	2.149669	-2.620766
53	-2.231737	2.972279	-1.49781	-2.311966	2.149739	-2.620709	-0.941382	2.908167	-1.820417
54	-2.742557	2.782678	0.939697	-3.761714	2.527139	-0.757752	-3.761669	2.527214	-0.757733
55	-5.434996	2.971219	-1.860255	-0.746164	4.520246	0.409722	-0.187906	2.867141	0.66641
56	-6.266929	2.544372	-0.367388	-0.778602	3.814529	2.042409	-0.778502	3.815128	2.042109
57	-6.309374	4.219685	-0.955778	-0.188066	2.867173	0.666241	-0.745913	4.520127	0.409112
58	-4.816224	5.698377	0.246325	-2.927576	2.940106	2.752195	-4.224853	2.545559	1.495923
59	-3.266409	5.039251	1.014564	-4.225156	2.545735	1.495883	-2.927215	2.940025	2.752142
60	-4.415631	0.766899	0.012047	-3.533921	4.824819	-0.765017	-3.53381	4.824884	-0.765116
61	6.033047	2.869251	0.485113	6.119389	-2.589201	0.537077	6.119405	-2.589278	0.536855
62	7.931092	-2.38582	0.258948	7.523583	2.813379	0.22579	7.52353	2.813352	0.226361
	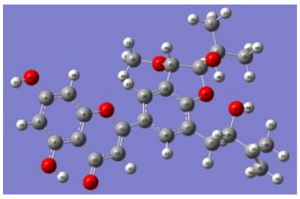 Conformer 4			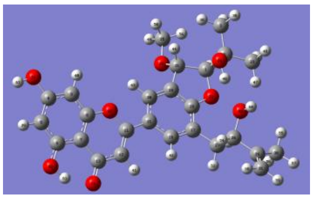 Conformer 5			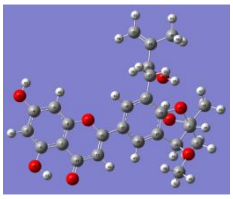 Conformer 6		
1	-3.459046	-2.14567	-0.506246	-3.455428	-2.009193	-0.530723	-3.985036	-1.580009	-0.217367
2	-4.130315	-2.136647	0.884778	-4.187551	-1.989372	0.829088	-4.636428	-1.244954	1.144842
3	-4.146664	-3.540114	1.491895	-4.336465	-3.404967	1.385974	-5.036734	-2.527472	1.878455
4	6.0625	-1.368826	1.073896	6.125912	-1.528798	0.938574	5.95529	1.93518	0.321614
5	6.789698	-0.215452	0.745521	6.875718	-0.385555	0.626103	6.780226	0.802646	0.381495
6	6.130443	0.877402	0.189865	6.233044	0.739989	0.118378	6.215123	-0.463567	0.284452
7	4.728808	0.825566	-0.0412	4.825569	0.731362	-0.080183	4.807906	-0.611192	0.126859
8	4.046729	-0.352903	0.305196	4.120501	-0.438783	0.247837	4.026129	0.553085	0.071608
9	4.682013	-1.454216	0.859004	4.739031	-1.572039	0.754362	4.568388	1.827992	0.165979
10	2.69592	-0.454852	0.10985	2.76327	-0.499705	0.082102	2.667884	0.473165	-0.072748
11	1.987455	0.569458	-0.441093	2.069799	0.558154	-0.422962	2.046913	-0.733017	-0.187065
12	2.594602	1.73149	-0.816293	2.699236	1.715115	-0.777606	2.749912	-1.900109	-0.159555
13	4.007353	1.940069	-0.627178	4.1207	1.881272	-0.615761	4.181143	-1.91513	0.013114

14	4.58717	3.009233	-0.955533	4.721459	2.94435	-0.926358	4.849417	-2.981579	0.051351
15	0.56535	0.248845	-0.573699	0.637682	0.278017	-0.534627	0.597033	-0.601551	-0.348479
16	-0.379341	1.267648	-0.802315	-0.284067	1.329385	-0.718534	0.034913	0.634831	-0.717051
17	-1.744488	1.012001	-0.938019	-1.654176	1.110551	-0.855804	-1.339359	0.814886	-0.892096
18	-2.127263	-0.328887	-0.861848	-2.068369	-0.223428	-0.820911	-2.13325	-0.316762	-0.696559
19	-1.221315	-1.361099	-0.612143	-1.191607	-1.278285	-0.578687	-1.610702	-1.55279	-0.317187
20	0.124868	-1.086167	-0.453732	0.163983	-1.047447	-0.442207	-0.249809	-1.706852	-0.127426
21	-2.000561	-2.653204	-0.555865	-1.992217	-2.548055	-0.498044	-2.760097	-2.519889	-0.174945
22	-3.411126	-0.75607	-0.968501	-3.355498	-0.617673	-0.972574	-3.494192	-0.323811	-0.803217
23	-5.568521	-1.613921	0.752612	-5.567183	-1.334466	0.673733	-5.887818	-0.379705	0.929081
24	-3.369905	-1.313973	1.77175	-3.395988	-1.251698	1.765405	-3.68573	-0.579692	1.979113
25	-1.881039	-3.448324	-1.73617	-1.67967	-3.379195	-1.610952	-2.890494	-3.413608	-1.280636
26	-0.609388	-4.059731	-1.882299	-2.163179	-4.706134	-1.461539	-1.877198	-4.405736	-1.33144
27	-2.757051	2.122897	-1.026431	-2.655797	2.233586	-0.907434	-1.932199	2.168932	-1.185989
28	-3.238248	2.547857	0.372847	-3.075917	2.688335	0.501777	-2.499637	2.847001	0.081127
29	-4.149132	3.761283	0.413074	-4.014216	3.877031	0.479324	-3.008736	4.247103	-0.202968
30	-4.439707	4.280713	1.798384	-5.335655	3.697872	-0.222349	-4.280908	4.362832	-1.000986
31	-4.679529	4.311311	-0.683342	-3.657354	5.016644	1.079474	-2.331573	5.308781	0.242828
32	-3.888161	1.452348	1.060344	-3.710814	1.565534	1.152679	-3.539146	2.038734	0.671637
33	6.82377	1.983674	-0.128877	6.94783	1.836971	-0.184792	7.001	-1.553024	0.33924
34	6.661141	-2.459102	1.616594	6.707924	-2.649633	1.435331	6.572015	3.139561	0.422733
35	-4.065588	-2.701127	-1.224095	-4.047279	-2.535132	-1.283644	-4.727563	-1.980471	-0.909559
36	-4.589108	-4.262049	0.797925	-4.895709	-3.366907	2.325648	-5.64487	-2.272309	2.751321
37	-4.742723	-3.533288	2.409531	-4.884437	-4.043448	0.685374	-5.623309	-3.185901	1.229572
38	-3.138198	-3.87479	1.748603	-3.366442	-3.865917	1.590962	-4.158104	-3.073813	2.230049
39	7.859993	-0.16207	0.917987	7.950847	-0.36513	0.773816	7.851055	0.916741	0.502164
40	4.130402	-2.349278	1.117579	4.169975	-2.459824	0.999899	3.926562	2.700802	0.120425
41	2.022383	2.522009	-1.283211	2.138149	2.533415	-1.208785	2.242458	-2.847129	-0.286946
42	-0.052077	2.302035	-0.834032	0.069646	2.355541	-0.720873	0.690144	1.486248	-0.862778
43	0.835563	-1.873116	-0.234147	0.853543	-1.86527	-0.270858	0.152864	-2.650638	0.221683
44	-1.733029	-3.257467	0.318875	-1.791174	-3.091873	0.432521	-2.700971	-3.09682	0.754673
45	-5.594502	-0.653808	0.22979	-6.192367	-1.888763	-0.034974	-6.660545	-0.941868	0.394416
46	-6.001897	-1.485986	1.748942	-6.072901	-1.321518	1.64454	-6.287805	-0.07783	1.901494
47	-6.19173	-2.316332	0.188871	-5.463955	-0.305801	0.320363	-5.678165	0.525131	0.352583
48	-3.460176	-0.381713	1.481701	-3.412154	-0.31236	1.485094	-3.537514	0.321777	1.625632
49	0.179026	-3.323083	-2.085416	-1.838497	-5.262979	-2.343244	-2.157788	-5.101514	-2.125325
50	-0.334971	-4.630713	-0.98267	-1.750919	-5.180968	-0.559315	-0.894338	-3.976206	-1.566317
51	-0.683349	-4.741904	-2.732366	-3.260452	-4.742917	-1.403477	-1.805196	-4.951888	-0.379158
52	-2.309048	2.992578	-1.517214	-2.228794	3.102298	-1.418865	-1.166586	2.826537	-1.608224
53	-3.616011	1.809726	-1.6293	-3.543302	1.920288	-1.463738	-2.728909	2.082823	-1.93518
54	-2.35503	2.751216	0.989423	-2.168486	2.957499	1.058891	-1.703791	2.898792	0.830462
55	-4.855874	3.487665	2.429593	-5.84673	2.798668	0.139166	-5.100896	3.839866	-0.495031
56	-3.516577	4.620677	2.28667	-5.199385	3.571756	-1.303832	-4.179549	3.907755	-1.994702
57	-5.144322	5.116606	1.774172	-5.988388	4.56046	-0.062868	-4.569379	5.408664	-1.136439

58	-4.474995	3.943802	-1.683555	-2.699632	5.119179	1.584125	-1.423688	5.199517	0.831565
59	-5.345082	5.167757	-0.612635	-4.306902	5.888402	1.082495	-2.65682	6.324279	0.031258
60	-4.745067	1.319245	0.621516	-3.993779	1.869603	2.030842	-3.960505	1.539661	-0.048621
61	6.151574	2.623536	-0.509352	6.283875	2.50487	-0.530263	6.384646	-2.337685	0.246415
62	7.612734	-2.291787	1.718951	7.665592	-2.509526	1.520731	5.911574	3.850283	0.370737
	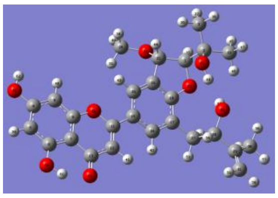			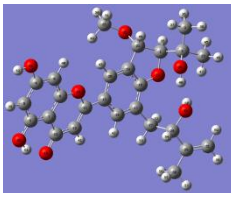			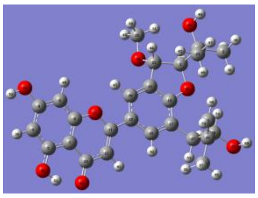		
	Conformer 7			Conformer 8			Conformer 9		
1	-3.383815	-2.17146	-0.285385	3.460311	-2.109954	0.513248	-3.772407	-1.686856	-0.513944
2	-4.088937	-2.133243	1.090944	4.105198	-2.197583	-0.888931	-4.599406	-1.784003	0.779859
3	-4.01829	-3.503889	1.768846	4.059022	-3.631626	-1.420843	-5.97712	-1.148642	0.572055
4	6.287676	-1.405185	0.168336	-6.07948	-1.442371	-0.982356	5.810185	-1.954744	0.940533
5	6.924377	-0.166921	0.336131	-6.807709	-0.276473	-0.704557	6.655133	-0.890157	0.594611
6	6.167864	0.999102	0.336511	-6.146591	0.846619	-0.215608	6.110676	0.270673	0.052649
7	4.755857	0.937976	0.166003	-4.741786	0.812641	-0.000935	4.707278	0.376865	-0.147084
8	4.166427	-0.324996	0.002327	-4.058717	-0.37973	-0.29415	3.904759	-0.717669	0.216008
9	4.901626	-1.503231	-0.001019	-4.695844	-1.510934	-0.781431	4.424346	-1.884065	0.756963
10	2.814856	-0.444358	-0.172077	-2.704844	-0.465689	-0.111355	2.547162	-0.666695	0.050858
11	2.008406	0.653133	-0.170187	-1.994748	0.58927	0.37534	1.94548	0.431294	-0.484747
12	2.516298	1.906043	0.001354	-2.601748	1.767226	0.695754	2.670864	1.519025	-0.872012
13	3.930199	2.131198	0.168805	-4.01829	1.959532	0.515298	4.102433	1.566355	-0.71664
14	4.421219	3.280999	0.31864	-4.598389	3.042149	0.794709	4.790572	2.564705	-1.058378
15	0.598765	0.299716	-0.357212	-0.569317	0.28112	0.508335	0.492985	0.269987	-0.582581
16	-0.350873	1.286204	-0.683451	0.373031	1.313026	0.677317	-0.33485	1.380644	-0.847178
17	-1.706961	1.000572	-0.849646	1.740942	1.071057	0.812194	-1.720588	1.280533	-0.956198
18	-2.071913	-0.339729	-0.69932	2.130858	-0.270748	0.7903	-2.254271	-0.001066	-0.779832
19	-1.159144	-1.341722	-0.377268	1.225245	-1.31648	0.610869	-1.465703	-1.119011	-0.517604
20	0.178261	-1.036305	-0.19586	-0.124496	-1.055985	0.455725	-0.091362	-1.001073	-0.416035
21	-1.913753	-2.645674	-0.288686	2.003407	-2.609361	0.625835	-2.356197	-2.329155	-0.44814
22	-3.352276	-0.799137	-0.812389	3.421207	-0.691947	0.892345	-3.582439	-0.277513	-0.844804
23	-5.562982	-1.736697	0.913483	5.568414	-1.734257	-0.815741	-3.880979	-1.148914	1.975203
24	-3.408803	-1.218055	1.952577	3.356785	-1.399417	-1.807923	-4.730831	-3.202346	0.965077
25	-1.754175	-3.474553	-1.440321	1.907649	-3.319291	1.861275	-2.085918	-3.172037	-1.562587
26	-0.467803	-4.06485	-1.541868	0.639982	-3.919641	2.074656	-2.65719	-4.467259	-1.421471
27	-2.725289	2.08357	-1.096181	2.739369	2.196738	0.867956	-2.60846	2.470326	-1.202616
28	-3.477716	2.484492	0.192682	3.35583	2.500891	-0.518791	-3.325926	2.948994	0.072565
29	-4.437295	3.634602	-0.045175	4.265648	3.717037	-0.481621	-2.37617	3.42095	1.156097
30	-5.678556	3.334334	-0.843729	3.612687	5.01642	-0.080551	-1.504621	4.607582	0.835625
31	-4.160402	4.848498	0.438731	5.559104	3.634344	-0.802769	-2.349114	2.805446	2.341858
32	-4.17249	1.350754	0.753226	4.015688	1.340107	-1.037201	-4.195824	4.006834	-0.352731
33	6.772075	2.189055	0.499107	-6.841004	1.965124	0.053998	6.916359	1.292716	-0.282536

34	7.086411	-2.502066	0.1786	-6.680006	-2.561581	-1.459897	6.294574	-3.106076	1.470751
35	-3.960008	-2.76739	-0.995085	4.079347	-2.619275	1.254002	-4.341786	-2.130304	-1.334477
36	-3.000608	-3.733124	2.094358	4.466758	-4.33592	-0.688578	-5.887208	-0.080558	0.353865
37	-4.357443	-4.295677	1.092895	3.037062	-3.932141	-1.665049	-6.497863	-1.635008	-0.259232
38	-4.662412	-3.505917	2.65314	4.656843	-3.699601	-2.334727	-6.585786	-1.262519	1.476525
39	7.999358	-0.122501	0.465377	-7.880513	-0.236516	-0.864598	7.728386	-0.957895	0.742436
40	4.404753	-2.458272	-0.131991	-4.143421	-2.415996	-1.000417	3.781643	-2.711928	1.0287
41	1.859752	2.765279	0.030688	-2.027553	2.584007	1.112279	2.182729	2.373247	-1.321491
42	-0.031934	2.314836	-0.815174	0.041501	2.346336	0.665779	0.111088	2.36298	-0.957095
43	0.892524	-1.799139	0.0876	-0.833699	-1.857218	0.289431	0.527088	-1.869261	-0.223497
44	-1.649967	-3.215323	0.609375	1.7177	-3.272973	-0.198133	-2.231024	-2.904541	0.476707
45	-6.108045	-2.506863	0.357778	6.170851	-2.439463	-0.233503	-2.918098	-1.630336	2.167261
46	-5.681701	-0.793122	0.374168	5.980419	-1.676734	-1.827412	-3.709695	-0.082527	1.805357
47	-6.025966	-1.625746	1.898402	5.668385	-0.752069	-0.345448	-4.496882	-1.250679	2.875732
48	-3.578754	-0.306832	1.635997	3.480642	-0.456467	-1.573406	-5.264701	-3.342374	1.763801
49	-0.510995	-4.771758	-2.373491	-0.145584	-3.170902	2.241132	-2.398907	-5.024152	-2.324855
50	-0.20258	-4.604789	-0.620722	0.732095	-4.540448	2.968762	-2.247633	-4.98617	-0.542715
51	0.310502	-3.318745	-1.749381	0.349402	-4.552301	1.222708	-3.74907	-4.425865	-1.317751
52	-3.452622	1.757993	-1.850038	3.556513	1.961508	1.561579	-3.384411	2.217061	-1.933155
53	-2.232028	2.976075	-1.492452	2.248806	3.096928	1.247142	-2.026398	3.297562	-1.619845
54	-2.740629	2.780738	0.945264	2.536453	2.696705	-1.22188	-3.920669	2.112488	0.465351
55	-5.435147	2.975457	-1.852112	2.684637	5.182386	-0.643108	-2.107397	5.428509	0.43228
56	-6.266093	2.545363	-0.359626	3.343024	5.027215	0.982838	-0.755621	4.362188	0.072175
57	-6.308876	4.22197	-0.944306	4.280486	5.862686	-0.263573	-0.973863	4.961836	1.723505
58	-4.815195	5.6978	0.260661	6.200826	4.510989	-0.779618	-2.994004	1.957595	2.558801
59	-3.264782	5.036955	1.026195	6.011512	2.697964	-1.111108	-1.679657	3.123338	3.137402
60	-4.414365	0.767074	0.014162	4.56964	0.987068	-0.321355	-4.661039	4.32557	0.437735
61	6.0394	2.872119	0.468657	-6.167301	2.627122	0.391152	6.312027	2.004635	-0.648388
62	6.547164	-3.300808	0.055758	-7.633454	-2.403192	-1.559761	7.26086	-3.045955	1.551988
	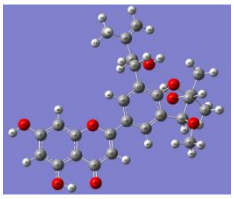 Conformer 10			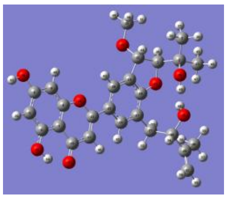 Conformer 11			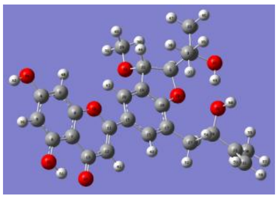 Conformer 12		
1	-4.04564	-1.552987	-0.268659	-3.379916	-2.055088	-0.333538	-3.425731	-2.025582	-0.440013
2	-4.705386	-1.188053	1.080563	-4.160961	-2.028082	1.001071	-4.03459	-2.043813	0.985073
3	-5.15234	-2.446337	1.82722	-4.178973	-3.416266	1.645332	-3.055988	-1.573565	2.07295
4	5.923918	1.853802	0.348164	6.314927	-1.542868	0.11348	6.142292	-1.508225	0.892806
5	6.735969	0.712819	0.422648	6.985821	-0.323128	0.283958	6.880456	-0.351273	0.604174
6	6.157004	-0.54949	0.327908	6.260015	0.864481	0.296611	6.225804	0.779755	0.124802
7	4.751722	-0.681486	0.158068	4.847771	0.841293	0.136267	4.817735	0.762767	-0.069098
8	3.983373	0.492738	0.087624	4.223491	-0.406429	-0.030369	4.124555	-0.421075	0.23428
9	4.537637	1.760302	0.179222	4.925469	-1.602244	-0.046107	4.755125	-1.559932	0.712425

10	2.62533	0.424528	-0.069549	2.866908	-0.486015	-0.194421	2.767153	-0.490415	0.072174
11	1.99284	-0.77583	-0.18083	2.091738	0.633155	-0.180015	2.062411	0.572558	-0.405622
12	2.683429	-1.950457	-0.138238	2.635019	1.87111	-0.006616	2.679725	1.74291	-0.735619
13	4.112177	-1.979398	0.047353	4.055492	2.056427	0.151749	4.100555	1.918192	-0.575249
14	4.769817	-3.052381	0.098672	4.579446	3.191524	0.3044	4.69008	2.99346	-0.863928
15	0.546654	-0.630477	-0.356177	0.670679	0.320303	-0.354463	0.631737	0.281574	-0.516984
16	0.000291	0.609742	-0.735241	-0.256308	1.338013	-0.656001	-0.299591	1.329864	-0.675265
17	-1.370245	0.804198	-0.922502	-1.619368	1.092054	-0.822233	-1.66949	1.103936	-0.800996
18	-2.179861	-0.316711	-0.731382	-2.020654	-0.240542	-0.684209	-2.070023	-0.23354	-0.766724
19	-1.671983	-1.557075	-0.339555	-1.134385	-1.262654	-0.364233	-1.181512	-1.292366	-0.582835
20	-0.31516	-1.725014	-0.136531	0.214793	-1.004391	-0.207165	0.174469	-1.051802	-0.462089
21	-2.834469	-2.508976	-0.198284	-1.904084	-2.546632	-0.247431	-1.978013	-2.573394	-0.583662
22	-3.534408	-0.308727	-0.854591	-3.306619	-0.672289	-0.828502	-3.359735	-0.631806	-0.870686
23	-5.924373	-0.286564	0.831587	-5.608222	-1.570741	0.76241	-4.552746	-3.448253	1.287778
24	-3.746501	-0.53728	1.916679	-3.490125	-1.161782	1.920076	-5.173127	-1.177812	0.975854
25	-2.963809	-3.417905	-1.292326	-1.544801	-3.406684	-1.323195	-1.760632	-3.249943	-1.817779
26	-1.961361	-4.421548	-1.318298	-1.984209	-4.74463	-1.135505	-2.259311	-4.580302	-1.810913
27	-1.940249	2.166865	-1.211781	-2.608243	2.202849	-1.063823	-2.683263	2.215593	-0.891127
28	-2.410671	2.883224	0.077411	-3.369044	2.600632	0.221292	-3.217679	2.647992	0.485638
29	-2.850696	4.312391	-0.19043	-4.295186	3.77918	-0.010815	-4.14603	3.842616	0.403303
30	-1.80863	5.22171	-0.793327	-5.533609	3.523511	-0.829099	-5.416219	3.669179	-0.388209
31	-4.080994	4.735812	0.110015	-3.992874	4.978191	0.494499	-3.827935	4.978895	1.030712
32	-3.422264	2.113921	0.737981	-4.100429	1.47613	0.752951	-3.89956	1.516274	1.063588
33	6.931374	-1.645761	0.397037	6.898719	2.035264	0.461701	6.929534	1.889906	-0.155073
34	6.441176	3.105186	0.436281	6.979462	-2.725738	0.094634	6.736238	-2.6347	1.361704
35	-4.786346	-1.952192	-0.964019	-3.929073	-2.622511	-1.088126	-4.113026	-2.518926	-1.129522
36	-5.764479	-3.087688	1.185084	-4.840541	-3.401839	2.516479	-3.579121	-1.585172	3.034472
37	-4.294947	-3.023098	2.183003	-4.551224	-4.169892	0.943981	-2.72264	-0.550805	1.881031
38	-5.749307	-2.159623	2.698183	-3.184531	-3.715246	1.985184	-2.177942	-2.221426	2.156747
39	7.81032	0.798599	0.552576	8.063601	-0.290621	0.407445	7.95591	-0.32433	0.74839
40	3.920469	2.648129	0.123998	4.417962	-2.549501	-0.177998	4.195136	-2.458347	0.939425
41	2.166564	-2.892603	-0.263406	2.002928	2.748075	0.031768	2.110111	2.566301	-1.145293
42	0.665882	1.453964	-0.877048	0.089736	2.359233	-0.776242	0.0483	2.357891	-0.671467
43	0.073896	-2.670967	0.221965	0.907666	-1.800534	0.036508	0.876738	-1.866272	-0.33139
44	-2.794309	-3.073057	0.740547	-1.706258	-3.051308	0.704829	-1.715764	-3.242931	0.245518
45	-6.72167	-0.840601	0.325119	-6.149954	-2.299421	0.150307	-5.304077	-3.747932	0.549696
46	-6.307315	0.07752	1.789335	-6.119698	-1.477328	1.724948	-5.015839	-3.465448	2.279097
47	-5.67612	0.57532	0.206051	-5.664038	-0.605178	0.253267	-3.740464	-4.182176	1.277007
48	-3.539914	0.338261	1.529011	-3.603865	-0.237777	1.616338	-4.827621	-0.28787	0.760495
49	-1.904212	-4.954049	-0.357214	-1.616605	-5.320109	-1.987757	-1.792267	-5.175664	-1.01269
50	-2.241694	-5.126561	-2.104141	-1.578499	-5.170267	-0.206523	-3.34985	-4.612843	-1.67694
51	-0.971497	-4.006681	-1.549998	-3.080543	-4.819982	-1.104236	-2.009932	-5.016713	-2.780459
52	-2.793353	2.099242	-1.898595	-2.088822	3.089808	-1.438442	-2.23292	3.096208	-1.360201
53	-1.1803	2.779146	-1.704055	-3.332645	1.907531	-1.833003	-3.524342	1.900245	-1.515733

54	-1.569964	2.902098	0.78232	-2.635651	2.865007	0.989161	-2.358013	2.90538	1.118618
55	-2.131528	6.265648	-0.755183	-6.149353	2.745809	-0.362124	-5.206349	3.545856	-1.45833
56	-1.607726	4.971953	-1.842513	-5.287054	3.169677	-1.838502	-5.950511	2.769052	-0.064706
57	-0.852885	5.137244	-0.259918	-6.137472	4.429646	-0.926965	-6.077537	4.532086	-0.271245
58	-4.816961	4.080875	0.563777	-3.100334	5.134494	1.095919	-4.472894	5.853274	0.992014
59	-4.382525	5.762707	-0.077813	-4.622847	5.846959	0.320821	-2.9073	5.075954	1.601381
60	-4.104213	1.916733	0.074605	-4.353873	0.915886	0.00005	-4.159403	1.766562	1.965093
61	6.306508	-2.424763	0.303288	6.18437	2.738655	0.44037	6.258644	2.559871	-0.482219
62	7.404159	3.052603	0.554288	7.931055	-2.567549	0.211113	7.692918	-2.487793	1.447156
	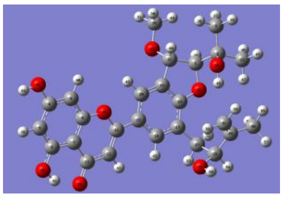 Conformer 13			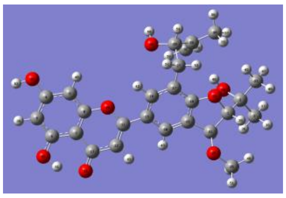 Conformer 14			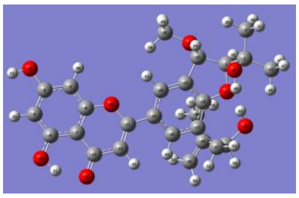 Conformer 15		
1	3.69723	1.549744	-0.643382	-4.114915	-1.102558	-0.464551	3.853072	-1.447897	0.044673
2	4.576123	1.612978	0.620089	-5.004229	-0.685909	0.722189	4.568234	-2.073193	-1.164492
3	4.836184	3.057315	1.045599	-5.69643	-1.894423	1.350731	4.887066	-3.541727	-0.886477
4	-5.861209	1.914202	0.980289	6.132472	1.538984	0.062408	-5.843181	-1.846303	0.783786
5	-6.721442	0.852867	0.663225	6.836302	0.34867	0.296195	-6.672253	-0.870685	0.211378
6	-6.19599	-0.322296	0.133415	6.145078	-0.858103	0.355105	-6.102491	0.23412	-0.414825
7	-4.796703	-0.446063	-0.083731	4.734588	-0.883587	0.181369	-4.688888	0.373758	-0.472382
8	-3.978397	0.646073	0.24981	4.076567	0.335903	-0.050268	-3.902921	-0.632161	0.115122
9	-4.478926	1.826466	0.778598	4.743935	1.55008	-0.113799	-4.447772	-1.741502	0.743644
10	-2.624037	0.578955	0.066083	2.718852	0.36993	-0.21813	-2.536821	-0.54488	0.094415
11	-2.040381	-0.534081	-0.458892	1.978503	-0.772356	-0.183762	-1.911651	0.502511	-0.510029
12	-2.782247	-1.621327	-0.816289	2.560522	-1.988848	0.020094	-2.615903	1.500571	-1.113395
13	-4.211428	-1.651376	-0.640897	3.979399	-2.121817	0.228212	-4.057724	1.506889	-1.122142
14	-4.914919	-2.64816	-0.955999	4.536862	-3.233959	0.428151	-4.729301	2.4236	-1.664432
15	-0.588573	-0.388783	-0.582835	0.551655	-0.518022	-0.392469	-0.452207	0.394068	-0.42968
16	0.222456	-1.508759	-0.851359	0.121402	0.726771	-0.891374	0.361577	1.518686	-0.667931
17	1.608639	-1.423239	-0.987675	-1.224186	1.027477	-1.115606	1.755205	1.460765	-0.633744
18	2.158383	-0.146145	-0.841868	-2.133968	0.011116	-0.816012	2.295847	0.210467	-0.3259
19	1.383545	0.979186	-0.570506	-1.738556	-1.22944	-0.317141	1.527907	-0.914535	-0.0426
20	0.012193	0.87802	-0.437433	-0.405095	-1.510751	-0.098773	0.145612	-0.840517	-0.109388
21	2.27909	2.182516	-0.508461	-2.955493	-2.086413	-0.121539	2.45846	-2.03294	0.36573
22	3.485304	0.127756	-0.94784	-3.479611	0.120662	-0.973418	3.638347	-0.022144	-0.241637
23	5.909301	0.895161	0.375474	-6.053759	0.33542	0.2665	5.849813	-1.296748	-1.487586
24	3.860962	0.995622	1.6983	-4.163782	-0.12576	1.739593	3.679223	-2.057826	-2.291315
25	1.954683	3.056032	-1.584647	-2.885097	-3.197441	-1.008411	2.444576	-2.296684	1.767322
26	2.540318	4.343724	-1.460084	-3.82993	-4.215472	-0.712303	1.283706	-2.980969	2.213974
27	2.460552	-2.64023	-1.234698	-1.663122	2.377037	-1.619195	2.612382	2.679362	-0.872113
28	2.730766	-3.470223	0.041153	-1.706792	3.459459	-0.51613	3.388603	3.212207	0.364253
29	3.543555	-2.715771	1.078514	-2.739346	3.169834	0.559138	2.50256	3.288597	1.605723

30	5.032935	-2.734488	0.864956	-4.146854	3.585584	0.226851	2.554011	2.133531	2.571046
31	2.960608	-2.11032	2.120354	-2.397067	2.613454	1.727565	1.716064	4.351234	1.800042
32	1.4611	-3.896539	0.523467	-0.382471	3.573766	-0.006706	4.601341	2.49866	0.594942
33	-7.016267	-1.341686	-0.173525	6.815641	-2.001565	0.576666	-6.893604	1.169892	-0.966256
34	-6.326606	3.078917	1.498588	6.763161	2.738861	-0.003904	-6.35225	-2.939712	1.405137
35	4.221552	1.976707	-1.502452	-4.720366	-1.502513	-1.282273	4.482203	-1.494737	0.935293
36	3.91142	3.579372	1.303664	-6.32659	-2.406866	0.617376	3.978541	-4.127457	-0.721531
37	5.34204	3.612261	0.249652	-6.334171	-1.554607	2.172306	5.412835	-3.965948	-1.746786
38	5.482079	3.059752	1.928752	-4.977758	-2.610599	1.756936	5.528429	-3.638267	-0.005262
39	-7.791882	0.933807	0.82414	7.913117	0.353691	0.432094	-7.752963	-0.963553	0.248426
40	-3.824232	2.652011	1.027869	4.210222	2.475068	-0.292304	-3.81716	-2.500003	1.190033
41	-2.307405	-2.487847	-1.25627	1.961853	-2.889898	0.015954	-2.100712	2.307007	-1.617867
42	-0.225458	-2.491918	-0.927128	0.857643	1.490372	-1.10542	-0.099326	2.478782	-0.873105
43	-0.591787	1.754836	-0.238131	-0.112346	-2.474263	0.303128	-0.472689	-1.710767	0.069604
44	2.174795	2.71788	0.443045	-3.036741	-2.445492	0.911538	2.260066	-2.957898	-0.188346
45	6.490153	0.880766	1.302737	-5.581222	1.198745	-0.206001	6.551136	-1.328322	-0.647024
46	6.495216	1.40929	-0.394174	-6.747491	-0.111054	-0.454169	5.626719	-0.247577	-1.704071
47	5.746374	-0.133603	0.048331	-6.626585	0.679563	1.133046	6.334065	-1.741186	-2.362056
48	3.698925	0.067814	1.44764	-3.724329	0.65567	1.357128	3.577678	-1.131043	-2.564824
49	3.637702	4.306029	-1.507664	-4.864985	-3.872353	-0.849089	1.100302	-3.886161	1.616874
50	2.247968	4.8257	-0.516026	-3.715535	-4.578585	0.31923	0.391241	-2.343378	2.174245
51	2.172361	4.939624	-2.298174	-3.636788	-5.035949	-1.406848	1.467722	-3.265417	3.252387
52	3.418919	-2.348141	-1.672884	-0.967492	2.728675	-2.388399	1.967029	3.48577	-1.232674
53	1.961883	-3.302191	-1.950428	-2.652107	2.306651	-2.080532	3.352307	2.477552	-1.656979
54	3.322796	-4.344971	-0.273118	-1.997794	4.400141	-1.011123	3.705508	4.228806	0.108247
55	5.554989	-2.121197	1.604072	-4.481021	3.136553	-0.716469	2.237038	1.192835	2.105296
56	5.295032	-2.365736	-0.134285	-4.206123	4.673576	0.093754	3.579302	1.977996	2.924596
57	5.418916	-3.759938	0.93132	-4.850019	3.295703	1.012027	1.90823	2.316056	3.434622
58	1.883423	-2.126167	2.251147	-1.369784	2.335954	1.940712	1.048036	4.420199	2.6548
59	3.546559	-1.604699	2.883851	-3.130675	2.44389	2.511791	1.708223	5.189965	1.106787
60	1.611819	-4.351214	1.368002	-0.414733	4.192142	0.741149	4.419487	1.547145	0.48995
61	-6.423461	-2.065608	-0.535293	6.121857	-2.726084	0.573943	-6.273074	1.855299	-1.354004
62	-7.291962	3.029285	1.596199	7.717427	2.613373	0.129163	-7.323017	-2.908473	1.377826
	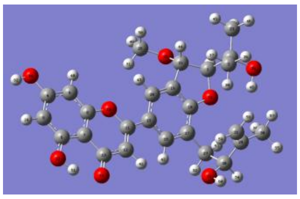 Conformer 16			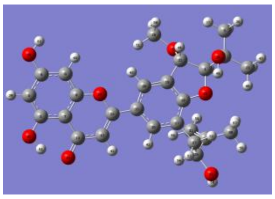 Conformer 17			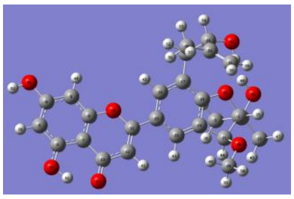 Conformer 18		
1	-3.724283	-1.789827	-0.35914	3.643544	-1.474433	0.128105	4.170976	-0.992741	-0.170093
2	-4.509121	-1.703802	0.963222	4.588472	-2.23158	-0.813743	4.758655	-1.366983	-1.543168
3	-3.715704	-0.999526	2.069949	4.810889	-3.653795	-0.303748	3.68101	-1.515082	-2.624008
4	5.98321	-1.836611	0.347465	-6.075136	-1.913557	0.044414	-6.101168	1.312303	-1.089459
5	6.712062	-0.644623	0.469158	-6.872575	-0.759997	0.033818	-6.80483	0.236517	-0.528649

6	6.051847	0.577198	0.373767	-6.269657	0.489921	-0.045787	-6.102943	-0.811833	0.059306
7	4.648168	0.61688	0.152496	-4.852163	0.599251	-0.117425	-4.681653	-0.793025	0.089714
8	3.964253	-0.605334	0.039425	-4.098743	-0.584428	-0.102999	-4.024752	0.308232	-0.485182
9	4.600177	-1.834218	0.131714	-4.678813	-1.843873	-0.024163	-4.702298	1.363661	-1.076034
10	2.61328	-0.625931	-0.180608	-2.73347	-0.540782	-0.174729	-2.657245	0.375114	-0.488716
11	1.901738	0.531519	-0.272591	-2.071894	0.648157	-0.239966	-1.907365	-0.606589	0.082033
12	2.50296	1.74912	-0.151803	-2.745043	1.833697	-0.240922	-2.483451	-1.691346	0.671647
13	3.923557	1.870651	0.055577	-4.184256	1.885413	-0.192209	-3.915068	-1.861786	0.701669
14	4.503386	2.984103	0.159651	-4.825759	2.969258	-0.202042	-4.468662	-2.860849	1.231368
15	0.474504	0.288439	-0.48937	-0.618866	0.469735	-0.28941	-0.469922	-0.33472	-0.011334
16	-0.354526	1.31442	-0.979577	0.221981	1.539199	-0.654094	-0.016187	0.962046	-0.320211
17	-1.729967	1.151878	-1.155767	1.610061	1.4175	-0.706982	1.338851	1.290014	-0.422228
18	-2.241528	-0.104937	-0.824136	2.126486	0.162173	-0.375281	2.224268	0.236215	-0.191527
19	-1.437495	-1.164619	-0.396433	1.328185	-0.916971	0.005617	1.816118	-1.057384	0.131653
20	-0.07959	-0.977175	-0.207718	-0.049854	-0.779917	0.031005	0.468304	-1.36151	0.209747
21	-2.308237	-2.393971	-0.276209	2.234821	-2.093819	0.2964	3.050647	-1.894859	0.382866
22	-3.562404	-0.429023	-0.884758	3.451889	-0.131345	-0.41111	3.582335	0.348139	-0.28905
23	-4.9498	-3.101302	1.393467	5.921751	-1.485987	-0.947791	5.601731	-2.633642	-1.413606
24	-5.716636	-0.974898	0.698545	3.960076	-2.350733	-2.098148	5.671569	-0.324278	-1.913328
25	-2.174729	-3.286252	-1.382144	2.173102	-2.613384	1.618464	3.324158	-2.084732	1.76889
26	-0.949631	-4.002567	-1.392661	1.096383	-3.511595	1.840601	2.426713	-2.976971	2.41236
27	-2.606332	2.264572	-1.669953	2.504314	2.581275	-1.039027	1.787327	2.691813	-0.764702
28	-2.907067	3.360122	-0.624095	2.858072	3.409679	0.213892	2.727417	3.377782	0.272163
29	-3.602087	2.814538	0.608942	3.669159	2.633472	1.234485	2.268465	3.12779	1.699783
30	-5.063842	2.494838	0.437942	5.108547	2.349733	0.894135	0.892052	3.636997	2.043555
31	-2.951711	2.648821	1.764398	3.097369	2.222711	2.370483	3.040381	2.492751	2.584769
32	-1.66575	3.996535	-0.337761	3.56563	4.556705	-0.269965	4.09202	3.05607	0.044621
33	6.746267	1.721625	0.492039	-7.02818	1.59993	-0.054386	-6.773392	-1.843352	0.599753
34	6.582039	-3.051448	0.43218	-6.728616	-3.100192	0.124419	-6.740982	2.356561	-1.673005
35	-4.318546	-2.333381	-1.095542	4.09208	-1.362516	1.120067	4.977811	-0.908453	0.558712
36	-4.352777	-0.898191	2.953778	5.481195	-4.184079	-0.986799	2.9816	-2.326624	-2.399746
37	-2.820764	-1.561643	2.355471	3.870792	-4.209335	-0.24587	3.10607	-0.589753	-2.733992
38	-3.405812	0.000406	1.75425	5.267081	-3.639856	0.690579	4.164294	-1.732192	-3.580878
39	7.784446	-0.65901	0.636376	-7.951466	-0.845394	0.088351	-7.889836	0.209406	-0.545034
40	4.047505	-2.760537	0.038076	-4.058384	-2.73319	-0.018282	-4.169456	2.198901	-1.512687
41	1.912429	2.654988	-0.189337	-2.205287	2.770922	-0.262364	-1.872074	-2.445152	1.14954
42	0.074084	2.27572	-1.234798	-0.212879	2.495607	-0.925474	-0.748869	1.741589	-0.495128
43	0.550353	-1.775757	0.164344	-0.692749	-1.610273	0.291847	0.144828	-2.376557	0.407917
44	-2.139443	-2.94856	0.656136	2.068714	-2.903938	-0.426503	3.01058	-2.871053	-0.117838
45	-4.091084	-3.737113	1.62768	6.579323	-2.031726	-1.631077	4.98782	-3.497133	-1.142103
46	-5.573117	-3.024803	2.28955	5.765123	-0.477985	-1.343974	6.086177	-2.844892	-2.371485
47	-5.536745	-3.580785	0.603601	6.422569	-1.396425	0.022176	6.378908	-2.502633	-0.654018
48	-5.440387	-0.132146	0.301707	3.814646	-1.443462	-2.416856	5.150322	0.495688	-1.9516
49	-1.023587	-4.738998	-2.195955	1.269957	-3.975716	2.814231	1.416225	-2.555206	2.490377

50	-0.783643	-4.523216	-0.43781	1.063242	-4.293016	1.067902	2.818974	-3.144194	3.417741
51	-0.092406	-3.345677	-1.589835	0.127774	-2.996189	1.864074	2.369923	-3.937855	1.880144
52	-2.118736	2.756677	-2.518361	3.426946	2.237112	-1.515774	0.894516	3.309899	-0.898211
53	-3.552282	1.85566	-2.034074	2.001364	3.25471	-1.740756	2.323267	2.698792	-1.721685
54	-3.589826	4.077874	-1.108454	1.91263	3.721516	0.68163	2.660373	4.456491	0.078232
55	-5.649838	3.408291	0.272697	5.604338	1.808318	1.704936	0.785239	4.696346	1.775422
56	-5.229682	1.862825	-0.44373	5.656437	3.279566	0.705882	0.112283	3.092215	1.497358
57	-5.469091	1.984639	1.316409	5.182293	1.742	-0.014094	0.687288	3.530751	3.112641
58	-3.456034	2.27014	2.648778	2.057413	2.445086	2.597204	2.701405	2.309905	3.601018
59	-1.897227	2.887027	1.859314	3.644202	1.639907	3.107634	4.034754	2.145184	2.325338
60	-1.828786	4.639268	0.371572	3.78799	5.101981	0.502011	4.163847	2.084245	0.045488
61	6.07234	2.458055	0.394448	-6.38572	2.367059	-0.113039	-6.072792	-2.463452	0.959813
62	7.535686	-2.936348	0.578149	-6.085191	-3.828116	0.125864	-7.701553	2.218482	-1.625998

Table S10. Conformational analysis of (1"*S*,2"*S*,2"'*R*)-**4**

Conformers	Gibbs Free Energy (Hartree)	Relative Gibbs Free Energy (kcal/mol)	Population (%)
1	-1609.105691	1.62399588	1.35
2	-1609.105927	1.47590352	1.74
3	-1609.107004	0.80007525	5.43
4	-1609.106065	1.38930714	2.01
5	-1609.106833	0.90737946	4.53
6	-1609.105893	1.49723886	1.67
7	-1609.106916	0.85529613	4.95
8	-1609.106189	1.3114959	2.29
9	-1609.106825	0.91239954	4.49
10	-1609.107498	0.49008531	9.16
11	-1609.108279	0	20.96
12	-1609.106034	1.40875995	1.94
13	-1609.106651	1.02158628	3.74
14	-1609.107388	0.55911141	8.16
15	-1609.107413	0.54342366	8.38
16	-1609.106911	0.85843368	4.92
17	-1609.107111	0.73293168	6.08
18	-1609.10574	1.59324789	1.42
19	-1609.106256	1.26945273	2.46
20	-1609.106475	1.13202804	3.10
21	-1609.105591	1.68674688	1.22

Table S11. Coordinates of (1''*S*,2''*S*,2'''*R*)-**4**

	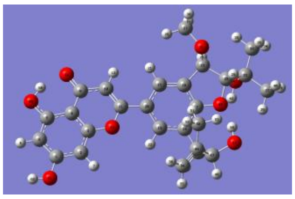			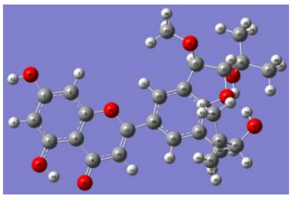			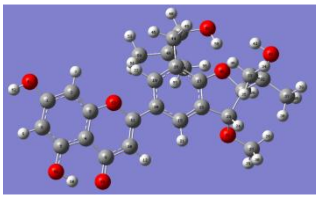		
	Conformer 1			Conformer 2			Conformer 3		
1	6.16706	1.360217	-0.192207	5.843177	-1.949491	0.740072	6.197028	1.217899	-1.005288
2	4.770432	1.441698	-0.242314	4.450441	-1.826377	0.67087	4.799145	1.292257	-1.005788
3	4.054106	0.255333	-0.200127	3.933471	-0.673307	0.100222	4.097982	0.236636	-0.443393
4	4.670242	-1.004411	-0.109677	4.74457	0.358633	-0.401608	4.730371	-0.886308	0.116506
5	6.090392	-1.050078	-0.062458	6.154756	0.199979	-0.31565	6.151378	-0.928272	0.100944
6	6.830742	0.127881	-0.102811	6.696792	-0.948582	0.253848	6.876779	0.119792	-0.458313
7	4.268308	2.398549	-0.309819	3.800831	-2.604675	1.051017	4.284893	2.144706	-1.431358
8	3.864225	-2.210027	-0.077011	4.141775	1.536251	-0.996635	3.939226	-1.954736	0.696794
9	7.914303	0.07801	-0.064413	7.775184	-1.056316	0.313243	7.961301	0.074973	-0.464032
10	2.438916	-2.00305	-0.147674	2.70011	1.544999	-1.027236	2.51099	-1.761362	0.652439
11	1.903184	-0.752389	-0.217004	1.970286	0.51971	-0.505581	1.958943	-0.654396	0.081269
12	1.799488	-2.875598	-0.160353	2.207475	2.387873	-1.493051	1.883095	-2.518441	1.102803
13	2.689622	0.357914	-0.242308	2.569444	-0.569456	0.049079	2.732091	0.326385	-0.459442
14	4.380749	-3.355863	-0.002402	4.836067	2.477815	-1.462142	4.470389	-2.973726	1.211489
15	-0.494471	-1.377501	0.080614	-0.108173	-0.818608	-0.216624	-0.434935	-1.355862	0.205222
16	-1.83373	-1.027968	0.01959	-1.492723	-0.883686	-0.175924	-1.772251	-1.021509	0.100834
17	-2.192816	0.243659	-0.420232	-2.241315	0.264915	-0.413024	-2.153867	0.279278	-0.213762
18	-1.275105	1.223694	-0.799318	-1.683144	1.518214	-0.674266	-1.242799	1.311029	-0.450472
19	0.068458	0.856008	-0.708242	-0.289132	1.563584	-0.685248	0.10303	0.949282	-0.350195
20	0.475694	-0.42258	-0.2808	0.50772	0.422694	-0.46815	0.52733	-0.354733	-0.023454
21	-0.196309	-2.360729	0.422427	0.50143	-1.6996	-0.065891	-0.145914	-2.372851	0.444246
22	0.823159	1.583507	-0.982631	0.18393	2.525736	-0.848168	0.854021	1.709345	-0.531947
23	-3.097529	-1.791241	0.357755	-2.44694	-2.031965	0.078569	-3.00582	-1.851404	0.335397
24	-3.248963	-2.621695	-0.344489	-2.391012	-2.771105	-0.731782	-3.005539	-2.781627	-0.24777
25	-4.196142	-0.715714	0.183689	-3.824539	-1.327641	0.091314	-4.136971	-0.892534	-0.131333
26	-4.546583	-0.413519	1.174686	-4.183485	-1.285006	1.123454	-4.897174	-0.779381	0.643716
27	-2.577086	-3.526022	1.911343	-1.298708	-3.679729	1.366928	-4.054265	-3.164853	2.00903
28	-2.911726	-4.266254	1.171015	-1.399834	-4.377601	0.523776	-5.073827	-2.830821	1.771484
29	-1.482209	-3.461758	1.877484	-0.291207	-3.245464	1.348738	-3.989245	-3.364531	3.08048
30	-2.87794	-3.85303	2.909142	-1.428085	-4.225801	2.304058	-3.850213	-4.091004	1.452596
31	-3.194499	-2.265237	1.692747	-2.31025	-2.682756	1.333396	-3.084064	-2.166984	1.719213
32	-5.372899	-1.108216	-0.718515	-4.888214	-1.930319	-0.834953	-4.823519	-1.216015	-1.47152
33	-6.083996	-2.33267	-0.145872	-5.164255	-3.379469	-0.439303	-3.817765	-1.377215	-2.617962
34	-6.922372	-2.601342	-0.795285	-5.922873	-3.798916	-1.10667	-4.364398	-1.551251	-3.549141
35	-5.410669	-3.192049	-0.082844	-4.262456	-3.993069	-0.516996	-3.138861	-2.220394	-2.456333
36	-6.472224	-2.121551	0.854901	-5.534838	-3.435882	0.588412	-3.21487	-0.471442	-2.739877
37	-6.344775	0.068546	-0.869141	-6.173213	-1.094692	-0.784544	-5.703006	-2.454618	-1.318289

38	-7.162645	-0.217039	-1.537187	-6.912545	-1.519953	-1.469577	-5.107882	-3.344681	-1.095264
39	-5.83814	0.942558	-1.290849	-5.977872	-0.058868	-1.080062	-6.245097	-2.630907	-2.251981
40	-6.769029	0.355395	0.098972	-6.597289	-1.085947	0.225261	-6.435055	-2.310463	-0.517162
41	-4.861832	-1.495434	-2.001121	-4.370636	-1.963703	-2.171915	-5.719102	-0.134587	-1.76647
42	-4.432691	-0.708836	-2.378738	-4.221178	-1.039894	-2.436007	-5.170777	0.665922	-1.82961
43	-3.544241	0.452927	-0.416013	-3.590286	0.054792	-0.338907	-3.509802	0.428586	-0.273804
44	-1.698396	2.600576	-1.250152	-2.525366	2.751683	-0.892266	-1.657706	2.723322	-0.792399
45	-2.446442	2.525212	-2.049413	-3.274143	2.565174	-1.672422	-2.209646	2.740403	-1.740093
46	-0.823761	3.107248	-1.668416	-1.872573	3.552328	-1.252247	-0.750368	3.315469	-0.944327
47	-2.292904	3.521154	-0.150075	-3.283322	3.288234	0.35227	-2.559965	3.44144	0.256011
48	-2.299059	4.5319	-0.572287	-3.61631	4.298309	0.090231	-2.474216	4.515984	0.046808
49	-1.429982	3.538656	1.108899	-2.376144	3.391806	1.575422	-2.075304	3.199404	1.676437
50	-1.786893	2.577304	2.211661	-2.347325	2.222378	2.523769	-0.681309	3.683348	1.983984
51	-1.697436	1.534241	1.885812	-1.967244	1.315155	2.039449	0.074936	3.115302	1.428615
52	-1.136677	2.716789	3.07995	-1.712569	2.434179	3.388854	-0.558144	4.736703	1.699709
53	-2.827174	2.722628	2.522895	-3.358645	1.992002	2.876389	-0.455771	3.586421	3.049767
54	-0.396012	4.380566	1.197304	-1.639692	4.490137	1.767686	-2.841438	2.593123	2.586244
55	-0.164047	5.084891	0.400907	-1.688708	5.336334	1.085288	-2.484501	2.415885	3.597319
56	0.259553	4.39309	2.064345	-0.957525	4.582048	2.609054	-3.848807	2.264757	2.353069
57	-3.661427	3.227026	0.125257	-4.481829	2.561076	0.616395	-3.935795	3.146372	0.062334
58	-3.771379	2.258927	0.124253	-4.301275	1.614082	0.475405	-4.028331	2.176332	0.0779
59	6.722648	-2.232883	0.021011	6.96924	1.159851	-0.785589	6.798608	-1.981525	0.627571
60	5.999174	-2.926759	0.032935	6.366012	1.875078	-1.145657	6.083746	-2.597134	0.966867
61	6.845081	2.534592	-0.233933	6.325116	-3.086447	1.301925	6.860307	2.26263	-1.561254
62	7.800388	2.362311	-0.192261	7.296525	-3.065684	1.298221	7.817906	2.107547	-1.507251
	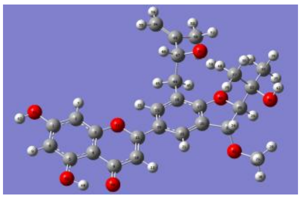 Conformer 4			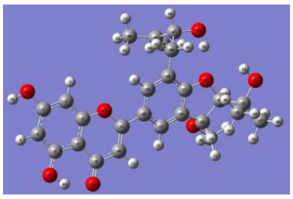 Conformer 5			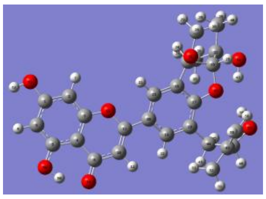 Conformer 6		
1	-6.121032	1.738416	0.297026	6.201431	1.36286	-0.257102	5.937439	-2.105471	0.138904
2	-4.727996	1.685445	0.170426	4.804103	1.443741	-0.282614	4.542825	-1.997303	0.084393
3	-4.137628	0.435172	0.067291	4.088474	0.260923	-0.177039	4.000165	-0.730656	-0.067751
4	-4.876042	-0.760087	0.086128	4.705818	-0.994814	-0.046724	4.787737	0.428747	-0.169091
5	-6.288901	-0.669165	0.215492	6.126684	-1.039975	-0.026494	6.200647	0.28208	-0.109592
6	-6.90412	0.575057	0.32097	6.866314	0.134527	-0.130548	6.768493	-0.979711	0.042459
7	-4.133586	2.590232	0.154072	4.300939	2.397553	-0.379199	3.911229	-2.873506	0.158965
8	-4.198606	-2.037261	-0.034753	3.90059	-2.197586	0.051886	4.158471	1.727534	-0.316319
9	-7.983812	0.629507	0.419674	7.950384	0.085131	-0.112654	7.848854	-1.075937	0.086344
10	-2.765613	-1.966687	-0.175249	2.474134	-1.992293	0.000711	2.71657	1.717122	-0.34004
11	-2.106242	-0.774005	-0.170742	1.93812	-0.744773	-0.108695	2.011515	0.554473	-0.255724
12	-2.222209	-2.893543	-0.301359	1.83246	-2.862737	0.034624	2.202534	2.66659	-0.408369
13	-2.774124	0.405736	-0.049509	2.723295	0.363357	-0.195641	2.634791	-0.648387	-0.124986

14	-4.827361	-3.128382	-0.027398	4.418609	-3.339676	0.163326	4.831009	2.788284	-0.404156
15	0.225695	-1.666972	-0.140062	-0.45181	-1.34486	0.287595	-0.053042	-0.76525	0.186391
16	1.583442	-1.442792	-0.274711	-1.787659	-0.997255	0.206637	-1.433965	-0.850186	0.16672
17	2.060137	-0.167699	-0.563492	-2.165452	0.248649	-0.285153	-2.200927	0.216822	-0.28773
18	1.225863	0.93801	-0.742462	-1.253049	1.210224	-0.725578	-1.652253	1.414053	-0.757024
19	-0.140585	0.695449	-0.595073	0.090053	0.834016	-0.646873	-0.257663	1.474452	-0.738863
20	-0.658532	-0.582127	-0.298877	0.509855	-0.418974	-0.156851	0.552028	0.419201	-0.272346
21	-0.139757	-2.659269	0.09865	-0.160762	-2.299801	0.710421	0.549866	-1.58508	0.55727
22	-0.829087	1.52255	-0.725138	0.841874	1.53592	-0.989218	0.212968	2.371238	-1.127737
23	2.749113	-2.390714	-0.203267	-3.017074	-1.736472	0.66262	-2.350792	-1.942567	0.647823
24	2.775805	-2.973465	0.724532	-3.076741	-2.752071	0.249919	-2.106851	-2.920113	0.211587
25	3.961094	-1.417764	-0.275679	-4.159354	-0.835618	0.115247	-3.739681	-1.437775	0.16501
26	4.633995	-1.718504	-1.081583	-4.864401	-0.575432	0.906817	-4.454345	-1.414209	0.989666
27	3.56729	-4.391882	-1.169186	-3.96827	-2.711413	2.604592	-2.865298	-3.194383	2.59531
28	4.615208	-4.081801	-1.067001	-4.995795	-2.396729	2.374543	-2.685918	-3.180579	3.672234
29	3.298275	-4.992509	-0.288489	-3.81599	-3.726578	2.210805	-2.423678	-4.104135	2.163728
30	3.454162	-4.997702	-2.070772	-3.836468	-2.7201	3.688416	-3.949402	-3.212358	2.416216
31	2.693939	-3.277689	-1.313887	-3.003034	-1.807734	2.082408	-2.255153	-2.02562	2.063726
32	4.766449	-1.284134	1.030017	-4.939716	-1.354999	-1.106725	-4.374341	-2.165861	-1.034498
33	5.958539	-0.341317	0.832596	-4.016872	-1.730132	-2.272739	-3.43322	-2.228453	-2.243696
34	6.578225	-0.331631	1.736481	-4.628562	-2.036974	-3.125921	-2.528369	-2.807294	-2.033333
35	6.575941	-0.677181	-0.006557	-3.344795	-2.555809	-2.018179	-3.131177	-1.223783	-2.55712
36	5.637063	0.686785	0.642587	-3.406233	-0.874904	-2.580183	-3.95723	-2.703243	-3.0781
37	3.900295	-0.830253	2.210666	-5.827793	-2.526897	-0.695241	-4.836611	-3.55787	-0.610403
38	4.513935	-0.789794	3.117916	-6.44199	-2.831027	-1.547884	-5.356221	-4.036357	-1.445713
39	3.076361	-1.525997	2.392907	-5.2344	-3.388587	-0.376999	-3.992558	-4.191809	-0.324627
40	3.489025	0.168483	2.040462	-6.494409	-2.236585	0.123196	-5.529687	-3.492827	0.234528
41	5.238605	-2.626059	1.233069	-5.83556	-0.312447	-1.518007	-5.565922	-1.449679	-1.389553
42	5.738345	-2.635362	2.065453	-5.280737	0.453439	-1.743959	-5.284534	-0.549767	-1.626974
43	3.422015	-0.109146	-0.649243	-3.5194	0.42631	-0.281347	-3.541327	-0.036896	-0.232604
44	1.777768	2.302425	-1.070851	-1.663933	2.566769	-1.24893	-2.488125	2.567433	-1.260993
45	0.99615	2.916243	-1.528374	-2.281015	2.459765	-2.149282	-3.071516	2.26293	-2.13836
46	2.58174	2.202616	-1.810225	-0.758505	3.103525	-1.547351	-1.809847	3.359822	-1.591218
47	2.319162	3.062541	0.159999	-2.476337	3.456863	-0.260371	-3.504728	3.164733	-0.241319
48	1.492144	3.190005	0.868567	-2.382734	4.48685	-0.629387	-3.752133	4.168982	-0.609981
49	2.847792	4.432769	-0.225634	-1.900895	3.406869	1.145877	-2.894258	3.309381	1.143509
50	4.145775	4.469322	-0.988686	-0.478657	3.885065	1.290296	-1.694377	4.216657	1.238691
51	4.062573	3.954349	-1.95461	-0.18561	3.939438	2.34261	-1.908285	5.200312	0.800289
52	4.465361	5.497261	-1.180738	0.225977	3.216435	0.780931	-1.391183	4.362693	2.279295
53	4.933145	3.956507	-0.424743	-0.349984	4.880199	0.844598	-0.834751	3.807899	0.693681
54	2.171314	5.534932	0.109596	-2.61686	2.962265	2.181253	-3.393674	2.666926	2.201887
55	1.244347	5.483376	0.676388	-3.64468	2.635849	2.062923	-2.945798	2.7683	3.18688
56	2.515566	6.526632	-0.173395	-2.196352	2.921059	3.182547	-4.268131	2.030041	2.120014
57	3.327431	2.309499	0.849061	-3.868007	3.178927	-0.318985	-4.739804	2.463515	-0.252117

58	3.68241	1.638501	0.241614	-3.978799	2.223909	-0.15904	-4.540164	1.523907	-0.087602
59	-7.035167	-1.786476	0.235628	6.760405	-2.218865	0.092798	6.993084	1.363588	-0.199337
60	-6.387506	-2.54639	0.143511	6.037385	-2.911105	0.148017	6.374134	2.145999	-0.298233
61	-6.674258	2.973342	0.395385	6.879182	2.533555	-0.360465	6.442331	-3.35567	0.28947
62	-7.63905	2.893418	0.476417	7.834953	2.36169	-0.328019	7.412526	-3.315564	0.318874
	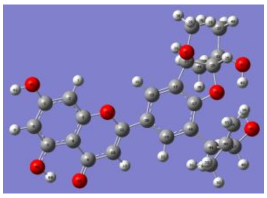 Conformer 7			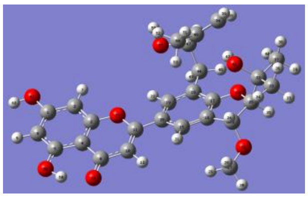 Conformer 8			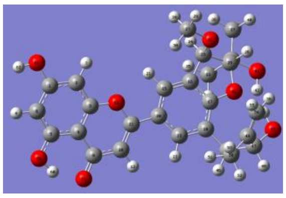 Conformer 9		
1	5.907641	-1.90007	0.814913	-5.972073	1.566346	0.058744	5.896345	-1.768744	0.803637
2	4.513626	-1.778533	0.773614	-4.586816	1.566303	-0.142227	4.49958	-1.677937	0.777696
3	3.983023	-0.652438	0.162978	-3.924704	0.350154	-0.066164	3.937162	-0.578192	0.147893
4	4.782008	0.354151	-0.405604	-4.584554	-0.861135	0.201686	4.706839	0.431567	-0.454449
5	6.193788	0.1974	-0.347183	-5.991759	-0.82439	0.399422	6.122295	0.306325	-0.410907
6	6.749298	-0.924408	0.261259	-6.677708	0.384741	0.328612	6.709584	-0.788698	0.216243
7	3.873394	-2.537265	1.205653	-4.051504	2.484561	-0.348504	3.881331	-2.439719	1.235633
8	4.165457	1.505196	-1.037385	-3.835606	-2.102696	0.258834	4.057644	1.554156	-1.104554
9	7.828763	-1.030521	0.299012	-7.751908	0.398233	0.483587	7.791496	-0.870515	0.242533
10	2.723512	1.51554	-1.031404	-2.419833	-1.981738	0.020687	2.615968	1.533756	-1.08033
11	2.00647	0.51541	-0.44707	-1.836181	-0.772161	-0.214657	1.92825	0.532611	-0.463457
12	2.219908	2.337597	-1.522104	-1.825359	-2.885697	0.015872	2.088186	2.332448	-1.584121
13	2.618026	-0.549357	0.140084	-2.569726	0.373867	-0.257744	2.570047	-0.504581	0.140949
14	4.848409	2.423217	-1.562797	-4.394853	-3.207635	0.489701	4.714009	2.474039	-1.659721
15	-0.069268	-0.806271	-0.06452	0.545365	-1.505687	-0.096173	-0.110793	-0.82899	-0.031186
16	-1.451422	-0.860443	-0.028302	1.880168	-1.260183	-0.361734	-1.491245	-0.916369	0.037751
17	-2.210313	0.279106	-0.270794	2.267703	-0.045843	-0.932554	-2.278049	0.198744	-0.242499
18	-1.651551	1.527856	-0.559738	1.362553	0.966462	-1.25458	-1.753478	1.453983	-0.560193
19	-0.256505	1.557075	-0.605005	0.014584	0.68254	-1.021913	-0.359079	1.52016	-0.609227
20	0.54533	0.421298	-0.372373	-0.41177	-0.532238	-0.453702	0.470834	0.408564	-0.367284
21	0.52765	-1.688019	0.13367	0.242722	-2.415371	0.409151	0.515592	-1.693153	0.151029
22	0.22011	2.508527	-0.815763	-0.718539	1.444686	-1.252205	0.091999	2.479357	-0.840325
23	-2.373545	-1.999228	0.31529	3.123681	-2.079981	-0.111986	-2.406685	-2.052837	0.436313
24	-2.174394	-2.898394	-0.282369	3.174017	-2.428069	0.926927	-2.145864	-3.000715	-0.05237
25	-3.769078	-1.398653	-0.013717	4.25546	-1.081088	-0.454395	-3.786613	-1.522462	0.002009
26	-4.445731	-1.495127	0.837238	4.916013	-1.529917	-1.199536	-4.505216	-1.639541	0.813889
27	-2.830912	-3.537649	2.062107	2.39053	-4.263653	-0.72383	-1.291973	-2.815091	2.400817
28	-3.921822	-3.50935	1.93256	2.435544	-4.564442	0.333423	-1.007803	-3.738816	1.875635
29	-2.605458	-3.697399	3.118423	1.352355	-4.00844	-0.974052	-1.517163	-3.048137	3.443753
30	-2.429673	-4.37506	1.473205	2.708058	-5.100725	-1.349819	-0.447463	-2.114748	2.366842
31	-2.216715	-2.309677	1.693838	3.279478	-3.191369	-0.992849	-2.470741	-2.249919	1.846946
32	-4.474207	-1.914687	-1.281968	5.067904	-0.548318	0.740824	-4.383601	-2.097868	-1.295362
33	-3.591261	-1.794715	-2.529976	5.741356	-1.692312	1.49618	-3.428203	-1.976216	-2.488601

34	-4.162	-2.124124	-3.402876	5.006933	-2.359479	1.9553	-3.927305	-2.356873	-3.384333
35	-2.68907	-2.41022	-2.457901	6.373791	-2.280659	0.823924	-2.507202	-2.548225	-2.338395
36	-3.284302	-0.756622	-2.69413	6.369929	-1.282274	2.292246	-3.155172	-0.931185	-2.667961
37	-4.946544	-3.350388	-1.064734	6.116828	0.45957	0.253342	-4.812395	-3.545396	-1.06529
38	-4.104251	-4.035115	-0.931873	6.651387	0.87248	1.114204	-3.952548	-4.189904	-0.861855
39	-5.518929	-3.677837	-1.937555	6.84402	-0.018654	-0.411546	-5.317171	-3.919565	-1.960853
40	-5.594518	-3.413915	-0.18454	5.642303	1.281188	-0.290717	-5.507933	-3.611552	-0.222679
41	-5.665838	-1.135401	-1.458873	4.166792	0.085414	1.65956	-5.589814	-1.370563	-1.567796
42	-5.377939	-0.213369	-1.56909	3.852558	0.896417	1.221708	-5.331977	-0.438554	-1.669301
43	-3.553103	0.044189	-0.191754	3.611924	0.070699	-1.087471	-3.615385	-0.075468	-0.189645
44	-2.475938	2.771898	-0.796588	1.83881	2.317437	-1.718861	-2.610935	2.66902	-0.828659
45	-3.108449	2.647889	-1.683977	1.241251	2.666061	-2.568563	-3.226169	2.512858	-1.72314
46	-1.790315	3.597334	-1.010206	2.877783	2.246626	-2.049735	-1.946957	3.511211	-1.045406
47	-3.425715	3.191609	0.366203	1.758801	3.414617	-0.635508	-3.589871	3.077121	0.314079
48	-3.659041	4.251883	0.20154	2.28015	4.292052	-1.043579	-3.846123	4.129722	0.134944
49	-2.748198	3.06417	1.721176	2.432769	3.032722	0.681102	-2.931519	2.979751	1.681034
50	-1.518749	3.912807	1.921284	1.598664	2.353622	1.739261	-1.72567	3.85933	1.890936
51	-0.696873	3.592697	1.269405	2.189317	2.170982	2.641596	-0.886706	3.551961	1.254774
52	-1.722254	4.964609	1.680872	0.734759	2.969286	2.011642	-1.949949	4.903557	1.636444
53	-1.165476	3.8586	2.954917	1.197152	1.398773	1.386252	-1.38708	3.823983	2.930296
54	-3.217102	2.246621	2.666748	3.72645	3.320485	0.871648	-3.395418	2.160453	2.627581
55	-2.721561	2.154203	3.62954	4.31544	3.830291	0.112622	-2.913095	2.089768	3.598907
56	-4.112501	1.653603	2.513241	4.242315	3.062339	1.793215	-4.273286	1.543888	2.465953
57	-4.680718	2.531301	0.289514	0.372515	3.717235	-0.464964	-4.827117	2.385784	0.223682
58	-4.501649	1.573511	0.270915	0.305233	4.45929	0.156856	-4.624781	1.432496	0.216246
59	6.996944	1.133353	-0.880639	-6.664393	-1.959369	0.655308	6.898262	1.24626	-0.976593
60	6.385484	1.832499	-1.257874	-5.97521	-2.688031	0.654494	6.267108	1.923036	-1.362132
61	6.402741	-3.009825	1.418243	-6.597308	2.768149	-0.020238	6.422583	-2.853114	1.426401
62	7.373875	-2.988921	1.393871	-7.549688	2.651115	0.132456	7.392661	-2.813051	1.388275
	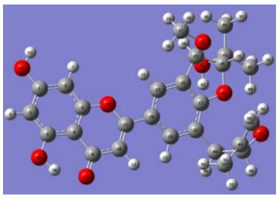 Conformer 10			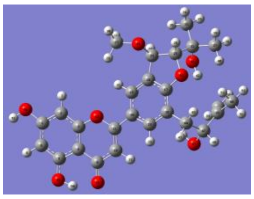 Conformer 11			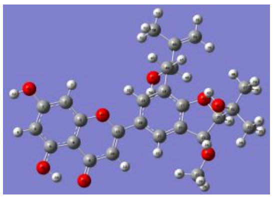 Conformer 12		
1	5.945561	-2.015778	0.050554	6.074473	1.767398	0.305278	-6.028128	1.453419	0.986465
2	4.549161	-1.92795	0.009603	4.688957	1.761768	0.105535	-4.628897	1.441104	0.948964
3	3.985527	-0.665201	-0.116977	4.054234	0.531487	0.027571	-4.008544	0.317818	0.423706
4	4.755013	0.505112	-0.205776	4.741206	-0.689195	0.139703	-4.723005	-0.790066	-0.062857
5	6.172236	0.37738	-0.160179	6.147352	-0.646155	0.34452	-6.142969	-0.742615	-0.011469
6	6.758841	-0.876354	-0.03349	6.806523	0.577193	0.425383	-6.787934	0.374527	0.511544
7	3.916412	-2.806327	0.071973	4.133774	2.686674	0.012929	-4.05243	2.279532	1.319037
8	4.104632	1.796482	-0.327924	4.017766	-1.944271	0.056934	-4.014561	-1.932901	-0.608005
9	7.837375	-0.975441	0.001175	7.880741	0.594279	0.579995	-7.872626	0.398133	0.544625

10	2.663093	1.764478	-0.340519	2.594087	-1.825869	-0.136174	-2.577033	-1.826157	-0.609084
11	1.975573	0.590505	-0.267851	1.990343	-0.610051	-0.255878	-1.943125	-0.729891	-0.10506
12	2.134313	2.707005	-0.390418	2.007005	-2.734218	-0.165657	-2.007029	-2.637915	-1.041158
13	2.61937	-0.604436	-0.160551	2.700679	0.548714	-0.177116	-2.639683	0.323114	0.403661
14	4.760219	2.868571	-0.40449	4.599301	-3.056479	0.159607	-4.62147	-2.940066	-1.058797
15	-0.063709	-0.768109	0.172846	0.012932	0.903366	-0.192949	0.392571	-1.603988	-0.190608
16	-1.445174	-0.874175	0.186165	-1.342195	1.102062	-0.390759	1.756188	-1.365537	-0.161004
17	-2.228041	0.179907	-0.277231	-2.16058	0.051123	-0.808974	2.233248	-0.072272	0.051924
18	-1.703236	1.38911	-0.74275	-1.65936	-1.222742	-1.101719	1.394069	1.034456	0.219197
19	-0.310274	1.480117	-0.721736	-0.282223	-1.393307	-0.923269	0.022627	0.777081	0.15147
20	0.518603	0.433787	-0.273125	0.560866	-0.3663	-0.460618	-0.494802	-0.518381	-0.048578
21	0.565808	-1.586541	0.49762	0.647614	1.699097	0.176251	0.013008	-2.614035	-0.289996
22	0.141796	2.389938	-1.102362	0.141501	-2.360084	-1.17356	-0.666469	1.605341	0.271837
23	-2.360192	-1.987714	0.643782	-2.198432	2.334171	-0.236411	2.945171	-2.285477	-0.31762
24	-2.105842	-2.939272	0.161089	-2.055546	2.804589	0.74342	2.890452	-3.136101	0.37251
25	-3.755992	-1.478806	0.215144	-3.626748	1.772283	-0.416255	4.1359	-1.350592	0.006351
26	-4.405568	-1.421171	1.0908	-4.136603	2.327188	-1.206568	4.830355	-1.344463	-0.836537
27	-1.25811	-2.792414	2.599653	-0.787655	4.022751	-1.156178	2.222021	-3.758784	-2.046653
28	-1.490166	-3.027101	3.640936	-0.657794	4.443157	-0.147906	2.171902	-4.57395	-1.309876
29	-1.020391	-3.72241	2.062728	0.079967	3.392027	-1.389882	2.58019	-4.15582	-2.999195
30	-0.380526	-2.13379	2.572601	-0.836585	4.837617	-1.8821	1.213227	-3.350278	-2.190942
31	-2.409231	-2.164297	2.056737	-2.008151	3.306827	-1.262696	3.146588	-2.759341	-1.646804
32	-4.428546	-2.273539	-0.915556	-4.48267	1.733523	0.863051	4.875877	-1.670746	1.316129
33	-4.721775	-3.699429	-0.451523	-4.637663	3.135653	1.453035	5.533385	-3.047271	1.227494
34	-5.205889	-4.251271	-1.26247	-5.053986	3.824816	0.711564	4.795014	-3.838543	1.070889
35	-5.391711	-3.693745	0.413531	-5.319521	3.094766	2.30765	6.255076	-3.077573	0.405606
36	-3.805641	-4.230405	-0.178251	-3.682546	3.535627	1.802832	6.062299	-3.256421	2.161966
37	-5.717915	-1.577042	-1.366485	-5.863907	1.142331	0.559627	5.923681	-0.593795	1.619117
38	-6.174046	-2.146705	-2.181396	-6.421574	1.784201	-0.130817	6.677162	-0.543544	0.825892
39	-6.437172	-1.509347	-0.543384	-6.433685	1.05464	1.489707	6.426018	-0.829104	2.562101
40	-5.511993	-0.562442	-1.721667	-5.776505	0.152777	0.108877	5.455738	0.391416	1.70586
41	-3.512696	-2.383675	-2.01494	-3.800869	0.946058	1.846388	3.919428	-1.745911	2.384487
42	-3.409091	-1.493728	-2.39126	-3.786563	0.025933	1.521481	3.575134	-0.847673	2.521778
43	-3.561764	-0.102942	-0.256038	-3.470984	0.393198	-0.898861	3.586231	0.004242	0.111503
44	-2.558803	2.521803	-1.262258	-2.526792	-2.372896	-1.541632	1.955469	2.419321	0.398694
45	-3.098341	2.209999	-2.164916	-1.949337	-3.016344	-2.212217	2.81498	2.39388	1.07689
46	-1.894217	3.338697	-1.559032	-3.393744	-2.011464	-2.100489	1.195157	3.071495	0.838604
47	-3.632095	3.07657	-0.277978	-2.998906	-3.254606	-0.348159	2.422901	3.018643	-0.951593
48	-3.889107	4.080437	-0.641379	-3.396596	-4.181468	-0.77996	3.214488	2.367193	-1.34857
49	-3.083145	3.215116	1.133084	-4.121436	-2.620594	0.456263	2.979669	4.414512	-0.776203
50	-1.907132	4.14511	1.289087	-5.42691	-2.460304	-0.27819	1.989518	5.519926	-0.517585
51	-1.650416	4.284031	2.343107	-5.726236	-3.407568	-0.744754	1.281601	5.605066	-1.349031
52	-1.017469	3.760568	0.775348	-6.226261	-2.144298	0.396871	1.392969	5.324086	0.382336
53	-2.122939	5.129841	0.853991	-5.347565	-1.720671	-1.082942	2.496531	6.480215	-0.387547

54	-3.612969	2.549301	2.161823	-3.972365	-2.287754	1.743842	4.298747	4.620946	-0.826013
55	-3.209258	2.647334	3.166098	-3.040778	-2.459306	2.273447	4.728485	5.60543	-0.658951
56	-4.46964	1.895909	2.034608	-4.797927	-1.878713	2.320726	4.995704	3.811251	-1.028791
57	-4.8472	2.344772	-0.351399	-1.891771	-3.666735	0.440672	1.334976	3.088656	-1.876453
58	-4.625584	1.407077	-0.20241	-1.421382	-2.863764	0.72328	0.942388	2.200647	-1.92071
59	6.946463	1.473583	-0.239152	6.845098	-1.788649	0.461091	-6.86718	-1.777875	-0.469178
60	6.314894	2.247249	-0.320531	6.172167	-2.526983	0.375111	-6.200187	-2.452388	-0.794096
61	6.583329	-3.20705	0.173374	6.672291	2.983379	0.37594	-6.611819	2.56254	1.506036
62	5.929707	-3.924195	0.223245	7.627837	2.8706	0.511061	-7.577962	2.461576	1.487476
	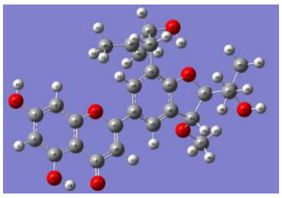 Conformer 13			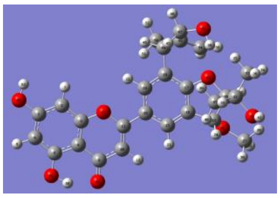 Conformer 14			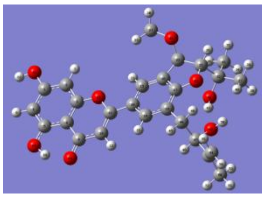 Conformer 15		
1	6.226091	1.382392	-0.213084	6.229498	1.279463	-0.877699	-6.2673	-1.377702	0.243789
2	4.828818	1.460122	-0.245479	4.831599	1.353626	-0.883039	-4.885463	-1.49803	0.055878
3	4.114642	0.271616	-0.169147	4.125948	0.258603	-0.401682	-4.135861	-0.331869	0.02036
4	4.735065	-0.982774	-0.062925	4.755857	-0.898667	0.081035	-4.705557	0.944464	0.16311
5	6.157921	-1.023498	-0.036584	6.178806	-0.938227	0.072081	-6.111368	1.029543	0.35574
6	6.893794	0.153331	-0.110495	6.906284	0.14552	-0.405537	-6.884517	-0.127403	0.395098
7	4.307026	2.407421	-0.324186	4.303991	2.228507	-1.246675	-4.419709	-2.468502	-0.060471
8	3.931703	-2.189246	0.006968	3.960421	-2.006486	0.5759	-3.865147	2.126726	0.121469
9	7.976813	0.124314	-0.088467	7.98951	0.117437	-0.413774	-7.95686	-0.047287	0.542846
10	2.505202	-1.985944	-0.045562	2.532833	-1.812182	0.531504	-2.458292	1.879388	-0.065451
11	1.966004	-0.737799	-0.131444	1.982396	-0.666618	0.039444	-1.968714	0.615409	-0.213613
12	1.865234	-2.858226	-0.03234	1.90401	-2.604657	0.914219	-1.789343	2.729587	-0.068087
13	2.75035	0.373277	-0.192586	2.761264	0.350681	-0.421316	-2.788169	-0.471755	-0.173099
14	4.451865	-3.332292	0.096027	4.488138	-3.060043	1.019838	-4.340488	3.286067	0.252097
15	-0.42305	-1.348404	0.250033	-0.414559	-1.357094	0.176138	-0.153629	-1.089819	-0.1929
16	-1.759575	-1.00228	0.17605	-1.750958	-1.018124	0.081349	1.175574	-1.416794	-0.390885
17	-2.13932	0.251096	-0.296319	-2.129975	0.284738	-0.230661	2.089193	-0.440631	-0.791424
18	-1.227957	1.219221	-0.72604	-1.215166	1.312034	-0.474417	1.732685	0.894963	-0.988377
19	0.115928	0.845117	-0.650881	0.129825	0.945369	-0.380335	0.383173	1.201679	-0.807364
20	0.53784	-0.413803	-0.178153	0.551993	-0.360387	-0.057081	-0.567691	0.24057	-0.414644
21	-0.130441	-2.30946	0.657871	-0.13087	-2.374678	0.418598	-0.867602	-1.828533	0.149565
22	0.866468	1.553852	-0.982127	0.881591	1.703906	-0.564667	0.069273	2.226716	-0.976774
23	-2.987937	-1.757197	0.606441	-2.985348	-1.845959	0.315607	1.928902	-2.716342	-0.236421
24	-3.075734	-2.739868	0.128103	-3.020844	-2.747818	-0.306882	1.69902	-3.214641	0.712728
25	-4.131265	-0.812814	0.138014	-4.119785	-0.858513	-0.080154	3.398735	-2.247383	-0.322021
26	-4.773815	-0.562056	0.985025	-4.817809	-0.744148	0.752198	3.949617	-2.892024	-1.009551
27	-3.913544	-2.877058	2.476484	-3.99412	-3.245303	1.938576	0.430617	-4.21183	-1.328891
28	-4.940037	-2.592643	2.211979	-5.010189	-2.939204	1.658645	0.186659	-4.669466	-0.358423
29	-3.816132	-2.918774	3.563393	-3.958148	-3.449006	3.010881	-0.346214	-3.476269	-1.575937

30	-3.711038	-3.870964	2.052336	-3.744526	-4.160897	1.38348	0.441673	-4.986626	-2.098948
31	-2.962203	-1.92273	2.018221	-3.040623	-2.219228	1.686387	1.724786	-3.630981	-1.314084
32	-4.996308	-1.332598	-1.022326	-4.900757	-1.213304	-1.356571	4.150724	-2.10941	1.02012
33	-6.103632	-0.322673	-1.339957	-6.00806	-0.181972	-1.59526	4.169403	-3.438007	1.777059
34	-6.717493	-0.140353	-0.451929	-6.677332	-0.135542	-0.730062	4.816353	-3.347386	2.654944
35	-5.68193	0.628652	-1.678335	-5.587465	0.813033	-1.769465	4.555667	-4.244182	1.144987
36	-6.750889	-0.709092	-2.135509	-6.598404	-0.459889	-2.475878	3.169737	-3.713501	2.123133
37	-4.163853	-1.638945	-2.271991	-3.986808	-1.323683	-2.581874	5.590752	-1.647279	0.751451
38	-3.682292	-0.733733	-2.653118	-3.501225	-0.367048	-2.795613	6.083884	-1.423086	1.701921
39	-4.814993	-2.029969	-3.061705	-4.58003	-1.601991	-3.460042	6.164078	-2.425333	0.236205
40	-3.39343	-2.38776	-2.067224	-3.215339	-2.085832	-2.43978	5.608796	-0.751837	0.123932
41	-5.559912	-2.535909	-0.479217	-5.471086	-2.489648	-1.031017	3.463631	-1.172516	1.85195
42	-6.133847	-2.922472	-1.160221	-6.001727	-2.774288	-1.792559	3.559128	-0.280634	1.456074
43	-3.491872	0.430505	-0.286717	-3.484207	0.442305	-0.278111	3.35491	-0.912788	-0.924917
44	-1.63889	2.57967	-1.240563	-1.622156	2.726331	-0.819428	2.761815	1.957366	-1.268759
45	-2.230021	2.478564	-2.158939	-2.152819	2.747356	-1.779222	2.301536	2.78139	-1.822952
46	-0.730559	3.127541	-1.508708	-0.710527	3.317046	-0.950524	3.569756	1.553403	-1.887913
47	-2.485978	3.452327	-0.266144	-2.546014	3.44515	0.209117	3.348429	2.518871	0.039319
48	-2.382709	4.488078	-0.616347	-2.444295	4.520436	0.010415	2.515011	2.821858	0.683694
49	-1.955995	3.384086	1.157275	-2.102256	3.189665	1.640672	4.293898	3.695851	-0.118601
50	-0.53877	3.85928	1.352241	-0.709967	3.652122	1.986877	4.694491	4.356127	1.176308
51	0.180558	3.196027	0.856469	0.051952	3.07507	1.448666	5.415787	5.161058	1.010107
52	-0.395578	4.859772	0.923184	-0.56399	4.704798	1.71079	3.816887	4.777606	1.684509
53	-0.278534	3.900319	2.41377	-0.513847	3.547554	3.057772	5.135159	3.625138	1.863346
54	-2.705166	2.928515	2.163919	-2.901146	2.591549	2.527366	4.76229	4.100592	-1.303032
55	-2.317427	2.87576	3.177841	-2.574259	2.405736	3.547063	4.478927	3.630961	-2.239245
56	-3.728285	2.60357	2.007452	-3.905975	2.278318	2.26461	5.454686	4.935438	-1.374286
57	-3.874053	3.171936	-0.374412	-3.918427	3.16397	-0.02411	4.01153	1.484773	0.804858
58	-3.983694	2.212014	-0.242899	-4.015405	2.193781	-0.02872	4.82938	1.267514	0.326847
59	6.792585	-2.204696	0.060425	6.821468	-2.027768	0.527411	-6.698402	2.229726	0.501011
60	6.0708	-2.898809	0.097414	6.104252	-2.665531	0.815747	-5.956817	2.902391	0.44133
61	7.006303	2.490571	-0.278591	7.001472	2.30002	-1.328985	-6.978569	-2.533087	0.271663
62	6.446861	3.282571	-0.339972	6.435432	3.029342	-1.631564	-7.921157	-2.333572	0.396661
	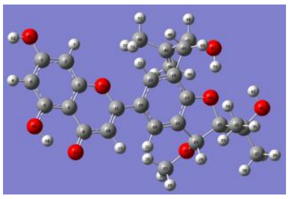 Conformer 16			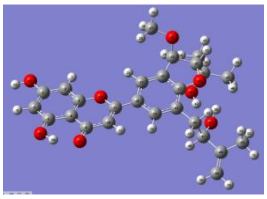 Conformer 17			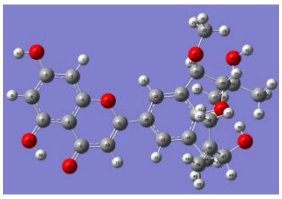 Conformer 18		
1	6.112775	1.452447	-0.361159	6.122067	-1.410045	-0.943118	5.841388	-1.970463	0.811387
2	4.714177	1.513679	-0.36189	4.7366	-1.478826	-0.755065	4.448935	-1.83474	0.76286
3	4.017388	0.32324	-0.221617	4.094357	-0.34653	-0.27736	3.932778	-0.707121	0.142626
4	4.654735	-0.921206	-0.080077	4.773863	0.847293	0.018453	4.744839	0.287544	-0.428331
5	6.076165	-0.94656	-0.086043	6.18067	0.881454	-0.183102	6.154611	0.11652	-0.362498

6	6.79707	0.235832	-0.225539	6.846964	-0.242819	-0.662136	6.695692	-1.007144	0.255491
7	4.19595	2.458561	-0.46617	4.186825	-2.384951	-0.976295	3.798888	-2.584235	1.196376
8	3.868499	-2.132665	0.056312	4.044991	1.995212	0.524316	4.143185	1.440762	-1.070518
9	7.881829	0.201552	-0.227982	7.921227	-0.202767	-0.81204	7.773835	-1.124215	0.298659
10	2.438695	-1.948058	0.028583	2.626628	1.802231	0.692879	2.701661	1.465358	-1.072737
11	1.882743	-0.710543	-0.095128	2.024082	0.622811	0.371031	1.971188	0.476031	-0.486186
12	1.81135	-2.827169	0.092138	2.04824	2.619871	1.10182	2.208536	2.288452	-1.572226
13	2.650729	0.406322	-0.216859	2.73888	-0.432795	-0.10668	2.569093	-0.590347	0.112184
14	4.40444	-3.265304	0.179195	4.621211	3.079186	0.805532	4.838434	2.348546	-1.597908
15	-0.492815	-1.344228	0.337068	0.156738	-1.02546	0.44085	-0.119766	-0.822729	-0.107371
16	-1.836657	-1.018558	0.280745	-1.193863	-1.289087	0.586761	-1.503143	-0.866359	-0.078084
17	-2.238121	0.208695	-0.246059	-2.100168	-0.245029	0.759194	-2.246524	0.278611	-0.340135
18	-1.347552	1.181323	-0.702412	-1.715452	1.098229	0.783829	-1.673113	1.516661	-0.641731
19	0.004334	0.835612	-0.620221	-0.347396	1.343376	0.655883	-0.278903	1.539317	-0.67194
20	0.449373	-0.405146	-0.125281	0.597529	0.312588	0.494379	0.508838	0.39674	-0.422358
21	-0.175007	-2.298354	0.740608	0.868851	-1.825233	0.280488	0.468693	-1.706102	0.108286
22	0.740215	1.548154	-0.974922	-0.018042	2.377205	0.646547	0.205434	2.488451	-0.873812
23	-3.069218	-1.748949	0.767126	-1.969397	-2.583601	0.599407	-2.441615	-1.980894	0.295388
24	-3.083221	-2.805134	0.467911	-1.673713	-3.249483	-0.218942	-2.302856	-2.885649	-0.30778
25	-4.203069	-0.934327	0.115154	-3.427405	-2.09073	0.471223	-3.832132	-1.344098	0.017535
26	-4.965667	-0.693518	0.856493	-4.049802	-2.598652	1.209796	-4.45188	-1.397102	0.916891
27	-2.334278	-2.423238	2.932793	-0.61654	-3.884705	2.066341	-2.856485	-3.530691	2.04055
28	-1.317216	-2.014435	2.875693	-0.715953	-4.500915	2.962774	-3.946762	-3.510436	1.907095
29	-2.315856	-3.469405	2.593812	0.166209	-3.133785	2.236016	-2.451791	-4.366349	1.452256
30	-2.672536	-2.386524	3.97055	-0.317663	-4.52124	1.22027	-2.629942	-3.685878	3.097432
31	-3.257204	-1.653288	2.176936	-1.883386	-3.287349	1.838645	-2.249645	-2.299911	1.66901
32	-4.874831	-1.539568	-1.131291	-4.061687	-2.190299	-0.936045	-4.603664	-1.937416	-1.180077
33	-3.86434	-1.919458	-2.220396	-3.99195	-3.628041	-1.45684	-5.962262	-1.248651	-1.324056
34	-3.277984	-1.049248	-2.533004	-4.584726	-3.711141	-2.372588	-6.52141	-1.694966	-2.151998
35	-3.169803	-2.695926	-1.884678	-4.392238	-4.333216	-0.721289	-6.553339	-1.35759	-0.406188
36	-4.4064	-2.296497	-3.092373	-2.964771	-3.91515	-1.694833	-5.836954	-0.179738	-1.519038
37	-5.73416	-2.73401	-0.722557	-5.532613	-1.749039	-0.880642	-3.806015	-1.842482	-2.479879
38	-5.122889	-3.550127	-0.327015	-5.943351	-1.740394	-1.894503	-4.389424	-2.279754	-3.295281
39	-6.277026	-3.104822	-1.597002	-6.12271	-2.44216	-0.272054	-2.859269	-2.385885	-2.411146
40	-6.463648	-2.440611	0.038942	-5.655177	-0.749084	-0.456259	-3.594022	-0.798673	-2.725441
41	-5.786587	-0.55702	-1.642109	-3.316252	-1.389162	-1.855264	-4.771166	-3.338065	-0.911719
42	-5.252825	0.226813	-1.857182	-3.480396	-0.445383	-1.650666	-5.469943	-3.43289	-0.243759
43	-3.597161	0.34457	-0.280715	-3.393941	-0.670408	0.851704	-3.593034	0.081813	-0.229497
44	-1.788247	2.516947	-1.254775	-2.72212	2.218206	0.844875	-2.499944	2.758232	-0.866499
45	-2.38916	2.37766	-2.161672	-2.232058	3.142409	1.165228	-3.267288	2.571238	-1.628217
46	-0.893773	3.073353	-1.550279	-3.496229	1.992688	1.588599	-1.842483	3.543634	-1.250712
47	-2.639015	3.400105	-0.292488	-3.393993	2.481411	-0.521263	-3.224655	3.321463	0.387145
48	-2.562968	4.427385	-0.672832	-2.610592	2.677551	-1.259719	-3.535631	4.338642	0.125838
49	-2.087367	3.381432	1.124079	-4.339069	3.666415	-0.47036	-2.295123	3.404914	1.595404

50	-0.679957	3.896312	1.286041	-5.626199	3.481862	0.290104	-2.307326	2.252824	2.565275
51	0.048875	3.239834	0.795132	-6.24137	4.384855	0.251635	-1.995969	1.314687	2.09081
52	-0.568466	4.889474	0.831424	-6.20521	2.650459	-0.128829	-1.637038	2.443068	3.408278
53	-0.405293	3.969097	2.342189	-5.44316	3.24123	1.345285	-3.319954	2.089262	2.95016
54	-2.809376	2.930844	2.152648	-4.011698	4.810313	-1.077584	-1.505269	4.470582	1.757075
55	-2.404986	2.912053	3.161227	-4.656372	5.68462	-1.035585	-1.524901	5.306387	1.060503
56	-3.826242	2.576451	2.021081	-3.083299	4.914477	-1.634348	-0.806873	4.544841	2.586772
57	-4.022573	3.088945	-0.372031	-4.083081	1.302631	-0.988119	-4.437988	2.628442	0.67247
58	-4.114633	2.13342	-0.204012	-4.374918	0.8095	-0.202625	-4.282001	1.674048	0.55309
59	6.728501	-2.114253	0.042629	6.871909	2.001152	0.089396	6.969753	1.040729	-0.898245
60	6.016426	-2.815149	0.124643	6.195032	2.664013	0.417988	6.366928	1.7436	-1.282747
61	6.772171	2.630171	-0.497859	6.727642	-2.530353	-1.411321	6.322156	-3.081514	1.423987
62	7.730699	2.472233	-0.477922	7.681863	-2.371679	-1.50306	7.293538	-3.071023	1.403637
	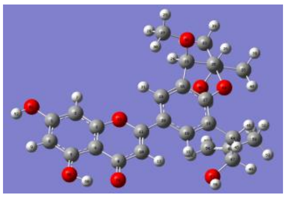 Conformer 19			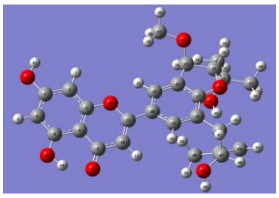 Conformer 20			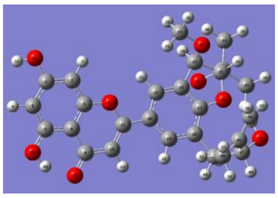 Conformer 21		
1	-5.811963	-1.818527	1.033622	5.910764	1.905841	0.392493	5.920636	-1.785538	0.757546
2	-4.430686	-1.756492	0.815723	4.530443	1.883017	0.162336	4.524132	-1.693028	0.729852
3	-3.915522	-0.587822	0.275346	3.919339	0.643613	0.023671	3.964328	-0.574058	0.131954
4	-4.718056	0.517024	-0.054665	4.62672	-0.565559	0.105044	4.736709	0.452824	-0.436638
5	-6.116739	0.419367	0.179154	6.028939	-0.503916	0.34304	6.15183	0.325372	-0.392404
6	-6.656777	-0.743595	0.721053	6.662406	0.725411	0.484072	6.736451	-0.788672	0.203053
7	-3.787933	-2.592251	1.062017	3.945015	2.792907	0.09097	3.903854	-2.467969	1.162267
8	-4.117908	1.708867	-0.624598	3.927554	-1.829339	-0.040034	4.090022	1.594977	-1.054924
9	-7.726506	-0.804739	0.894952	7.729964	0.773975	0.663688	7.818199	-0.872569	0.230075
10	-2.691293	1.652846	-0.81509	2.506758	-1.728687	-0.258255	2.649117	1.574813	-1.036397
11	-1.964871	0.554422	-0.460565	1.8822	-0.520138	-0.345781	1.957359	0.556158	-0.451949
12	-2.206816	2.509383	-1.264055	1.935789	-2.64485	-0.330934	2.123692	2.38872	-1.517775
13	-2.56233	-0.545732	0.075597	2.571441	0.646851	-0.209844	2.597515	-0.499257	0.122936
14	-4.806788	2.716404	-0.937289	4.527559	-2.934249	0.034239	4.749864	2.530576	-1.579891
15	0.062149	-0.89367	-0.449255	-0.124955	0.951138	-0.243297	-0.084394	-0.813881	-0.05921
16	1.4296	-1.029705	-0.611404	-1.480618	1.134126	-0.454101	-1.464178	-0.900714	0.012658
17	2.22264	0.090187	-0.86697	-2.259189	0.086253	-0.947377	-2.249891	0.222115	-0.239214
18	1.696091	1.376797	-1.012185	-1.736655	-1.173019	-1.250577	-1.722334	1.482478	-0.535488
19	0.3108	1.485628	-0.881305	-0.360421	-1.325087	-1.074133	-0.328029	1.548529	-0.581549
20	-0.517171	0.382958	-0.599485	0.452784	-0.294476	-0.568029	0.50059	0.431317	-0.362626
21	-0.560974	-1.746027	-0.210672	0.48779	1.735634	0.182991	0.541758	-1.683166	0.096719
22	-0.127592	2.472269	-0.966042	0.075075	-2.287235	-1.314482	0.12389	2.512199	-0.791937
23	2.325612	-2.24288	-0.560556	-2.385868	2.319473	-0.216225	-2.379303	-2.052461	0.360838
24	2.177133	-2.813688	0.363988	-2.276801	2.71079	0.802644	-2.146773	-2.952773	-0.219794
25	3.738599	-1.619317	-0.643003	-3.79081	1.721478	-0.469875	-3.774207	-1.48174	0.01688

26	4.272579	-2.050808	-1.492508	-4.308223	2.32649	-1.217711	-4.438458	-1.597	0.874567
27	1.009305	-3.886901	-1.679862	-1.021811	4.12214	-0.971012	-1.224808	-3.018102	2.209268
28	1.108763	-4.622281	-2.481652	-1.084806	4.990945	-1.630313	-0.987409	-3.895082	1.589105
29	0.8748	-4.411437	-0.722246	-0.133365	3.536288	-1.240775	-0.358511	-2.343918	2.21418
30	0.121227	-3.271057	-1.872521	-0.916916	4.464025	0.069416	-1.424143	-3.344141	3.232577
31	2.200131	-3.115802	-1.682287	-2.219131	3.383162	-1.152498	-2.400079	-2.367417	1.75197
32	4.582025	-1.723048	0.639061	-4.676173	1.523857	0.775271	-4.430637	-2.074285	-1.235355
33	4.793759	-3.184384	1.033734	-4.910581	2.849586	1.496526	-4.829924	-3.530157	-0.962211
34	5.441821	-3.230925	1.91412	-3.980786	3.256874	1.902483	-5.265838	-3.966186	-1.867816
35	5.273999	-3.739167	0.221723	-5.603059	2.693301	2.32902	-5.578435	-3.592359	-0.165418
36	3.850243	-3.677074	1.282361	-5.34694	3.588578	0.817173	-3.965201	-4.136096	-0.676698
37	5.937408	-1.034446	0.436908	-6.01453	0.893378	0.368781	-5.653237	-1.247026	-1.648206
38	6.4943	-1.045337	1.37875	-5.854534	-0.055887	-0.150434	-6.114165	-1.687058	-2.539653
39	6.530323	-1.553216	-0.324188	-6.615011	0.705793	1.264056	-6.406923	-1.231417	-0.853532
40	5.807648	0.001963	0.119063	-6.577423	1.557883	-0.295451	-5.360948	-0.21942	-1.875322
41	3.857818	-1.109015	1.712478	-3.986695	0.665111	1.694248	-3.42217	-2.011715	-2.255001
42	3.712438	-0.175965	1.470768	-3.967219	-0.217706	1.283803	-3.815068	-2.352882	-3.074601
43	3.549415	-0.194226	-0.936903	-3.575981	0.399193	-1.059484	-3.582912	-0.04332	-0.171797
44	2.5708	2.58158	-1.229334	-2.633908	-2.31874	-1.636478	-2.573572	2.70265	-0.804864
45	2.104081	3.252192	-1.961016	-2.213467	-2.869821	-2.485063	-3.146799	2.568321	-1.730504
46	3.535492	2.279773	-1.647903	-3.611495	-1.93612	-1.93928	-1.902606	3.55075	-0.97215
47	2.817579	3.402592	0.067834	-2.853345	-3.341785	-0.500931	-3.605373	3.085699	0.297362
48	3.428301	4.271503	-0.222383	-3.635651	-4.030385	-0.849939	-3.852719	4.142301	0.127965
49	3.592395	2.620224	1.106503	-3.322971	-2.714314	0.810446	-3.014558	2.962757	1.692978
50	5.083542	2.595565	0.904149	-2.277892	-2.294454	1.814504	-1.805664	3.819808	1.969361
51	5.577946	1.955914	1.639935	-1.642022	-3.141019	2.094826	-0.941551	3.500587	1.373944
52	5.345541	2.232486	-0.097121	-1.609972	-1.529534	1.40658	-1.998271	4.869384	1.710679
53	5.501118	3.607188	0.990324	-2.746255	-1.900218	2.720938	-1.520739	3.772834	3.024287
54	2.985962	2.016653	2.134693	-4.633191	-2.56762	1.043711	-3.539484	2.145547	2.608888
55	3.556611	1.482862	2.891071	-5.381107	-2.892751	0.324039	-4.418256	1.545975	2.396713
56	1.911292	2.070269	2.2674	-5.006252	-2.122728	1.962898	-3.107945	2.059402	3.602646
57	1.58841	3.845944	0.635523	-1.622019	-4.051582	-0.350083	-4.836436	2.397143	0.13271
58	1.157919	4.419491	-0.018348	-1.761746	-4.752718	0.306222	-4.628753	1.444584	0.10957
59	-6.922246	1.451621	-0.123656	6.743543	-1.639263	0.43183	6.92997	1.28173	-0.92692
60	-6.321158	2.163174	-0.495802	6.085603	-2.384509	0.303264	6.299538	1.969972	-1.293698
61	-6.29205	-2.972127	1.562965	6.591827	3.070615	0.538308	6.44477	-2.888752	1.348645
62	-7.255161	-2.904563	1.671538	5.979525	3.820239	0.452897	7.414933	-2.847189	1.314761

Table S12. Conformational analysis of (1″*S*,2″*S*)-**5**

Conformers	Gibbs Free Energy (Hartree)	Relative Gibbs Free Energy (kcal/mol)	Population (%)
1	-1533.897972	0	7.74
2	-1533.897232	0.4643574	3.53
3	-1533.897219	0.47251503	3.49

4	-1533.896544	0.89608428	1.71
5	-1533.897408	0.35391564	4.26
6	-1533.897249	0.45368973	3.60
7	-1533.89653	0.90486942	1.68
8	-1533.896551	0.89169171	1.72
9	-1533.89676	0.76054212	2.14
10	-1533.896476	0.93875496	1.59
11	-1533.896991	0.61558731	2.74
12	-1533.896719	0.78627003	2.05
13	-1533.897386	0.36772086	4.16
14	-1533.897923	0.03074799	7.35
15	-1533.896694	0.80195778	2.00
16	-1533.896509	0.91804713	1.64
17	-1533.896925	0.65700297	2.55
18	-1533.896553	0.89043669	1.72
19	-1533.896872	0.690261	2.41
20	-1533.897076	0.56224896	3.00
21	-1533.897629	0.21523593	5.38
22	-1533.897307	0.41729415	3.83
23	-1533.89633	1.03037142	1.36
24	-1533.897923	0.03074799	7.35
25	-1533.897142	0.5208333	3.21
26	-1533.896791	0.74108931	2.21
27	-1533.897497	0.29806725	4.68
28	-1533.897883	0.05584839	7.04
29	-1533.896159	1.13767563	1.13
30	-1533.896238	1.08810234	1.23
31	-1533.896428	0.96887544	1.51

Table S13. Coordinates of (1"*S*,2"*S*)-**5**

	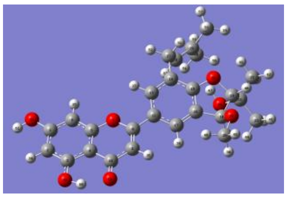 Conformer 1			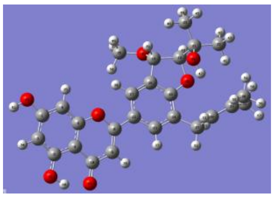 Conformer 2			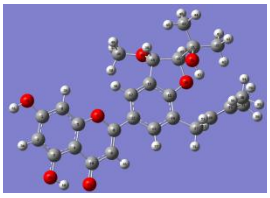 Conformer 3		
1	6.007448	2.056771	0.168894	-5.95357	1.560655	1.058123	-5.953595	1.560667	1.058163
2	6.80966	0.919325	0.341165	-6.762684	0.456179	0.754226	-6.762725	0.456225	0.754185
3	6.220693	-0.342037	0.346018	-6.183917	-0.695045	0.227448	-6.183971	-0.694998	0.227389
4	4.815364	-0.476427	0.179085	-4.781716	-0.751263	0.00029	-4.781753	-0.751255	0.000321
5	4.056995	0.694124	0.008873	-4.016168	0.382707	0.319934	-4.01619	0.382683	0.320044
6	4.621206	1.960781	0.000466	-4.570308	1.540337	0.84512	-4.570322	1.540314	0.845235
7	2.69929	0.623882	-0.150664	-2.661132	0.381229	0.126967	-2.661138	0.381166	0.127177

8	2.057677	-0.576776	-0.170281	-2.028374	-0.706015	-0.394685	-2.028404	-0.70603	-0.394604
9	2.739152	-1.749457	-0.029728	-2.717265	-1.830362	-0.741816	-2.717314	-1.830324	-0.741864
10	4.166015	-1.774182	0.168718	-4.142318	-1.930201	-0.553911	-4.142368	-1.930158	-0.553972
11	4.814836	-2.844459	0.311485	-4.797888	-2.961976	-0.858136	-4.797952	-2.961902	-0.858277
12	0.613788	-0.436518	-0.367133	-0.586654	-0.493002	-0.533832	-0.58668	-0.493045	-0.533729
13	0.076711	0.778221	-0.838532	0.284319	-1.587894	-0.706721	0.284277	-1.587968	-0.706508
14	-1.290063	0.959653	-1.052224	1.662139	-1.433404	-0.850916	1.662102	-1.433511	-0.850678
15	-2.102057	-0.148396	-0.801138	2.135347	-0.117997	-0.845835	2.13533	-0.118106	-0.8457
16	-1.609527	-1.35185	-0.295931	1.308802	0.9848	-0.637246	1.308794	0.984728	-0.637233
17	-0.254288	-1.505792	-0.064308	-0.054595	0.811092	-0.469302	-0.054608	0.811046	-0.469297
18	-2.784512	-2.272491	-0.069685	2.180454	2.217462	-0.623647	2.180468	2.217374	-0.623776
19	-3.986908	-1.321238	-0.272536	3.601329	1.60907	-0.576398	3.60133	1.60896	-0.576428
20	-3.452654	-0.137911	-0.950257	3.449391	0.20568	-0.968357	3.449374	0.205544	-0.968235
21	-4.71407	-0.806457	0.992441	4.33497	1.604281	0.786248	4.334936	1.604341	0.786243
22	-5.960273	-0.020831	0.563992	5.776828	1.125718	0.571946	5.776773	1.125673	0.572077
23	-5.105506	-1.952165	1.923238	4.327966	2.987195	1.434417	4.327957	2.987339	1.434223
24	-3.827309	0.0411	1.732658	3.651981	0.723836	1.686478	3.651867	0.724041	1.686574
25	-2.909191	-3.299484	-1.054402	2.109524	2.987778	-1.824485	2.109569	2.987535	-1.824727
26	-1.918457	-4.309499	-0.950372	0.886952	3.69122	-1.976649	0.887063	3.691089	-1.976923
27	-1.902084	2.304041	-1.373396	2.628034	-2.596126	-0.835147	2.627985	-2.596239	-0.834865
28	-2.57171	2.856492	-0.134408	3.321625	-2.655063	0.507961	3.321754	-2.655108	0.508144
29	-3.848096	3.247302	0.013637	4.640662	-2.633811	0.759935	4.64083	-2.633681	0.759894
30	-4.345187	3.76353	1.342034	5.153975	-2.690024	2.17821	5.154413	-2.689995	2.178064
31	-4.872541	3.260517	-1.092426	5.712721	-2.602001	-0.299931	5.712663	-2.601591	-0.300206
32	6.985665	-1.434946	0.508567	-6.955385	-1.755345	-0.066784	-6.95547	-1.755252	-0.06693
33	6.534611	3.307068	0.157024	-6.471692	2.703744	1.574072	-6.471708	2.703746	1.574141
34	7.884141	1.006437	0.46938	-7.834487	0.485315	0.9237	-7.834538	0.485394	0.923595
35	4.011714	2.845953	-0.130858	-3.95527	2.398648	1.084486	-3.95527	2.398599	1.084658
36	2.215839	-2.694838	-0.084055	-2.202828	-2.67138	-1.187348	-2.20288	-2.671293	-1.187496
37	0.745854	1.611209	-1.022937	-0.113745	-2.597074	-0.677832	-0.113814	-2.597136	-0.677529
38	0.128049	-2.41755	0.379971	-0.708573	1.653753	-0.282823	-0.70859	1.653724	-0.282902
39	-2.764983	-2.728207	0.927238	1.965461	2.865498	0.233983	1.965464	2.865533	0.233756
40	-4.699333	-1.790051	-0.953918	4.219202	2.091217	-1.336212	4.219225	2.091013	-1.336283
41	-5.696982	0.759967	-0.153764	5.792292	0.167486	0.046913	6.348372	1.848565	-0.019872
42	-6.700094	-0.679402	0.096398	6.348372	1.848739	-0.019895	5.79221	0.167349	0.047217
43	-6.415781	0.447163	1.442002	6.268268	1.003451	1.541973	6.268176	1.003559	1.542142
44	-4.226122	-2.397544	2.39558	4.672849	3.750209	0.728949	4.997265	2.990706	2.299791
45	-5.759268	-1.57427	2.715036	4.997233	2.990426	2.300017	3.326948	3.257389	1.780458
46	-5.642876	-2.733286	1.375713	3.326944	3.257219	1.780642	4.672794	3.750265	0.728639
47	-3.647628	0.819514	1.172856	3.727554	-0.174859	1.31458	3.727457	-0.174703	1.314794
48	-0.921044	-3.934195	-1.214971	1.004915	4.343518	-2.844958	0.666238	4.304582	-1.090689
49	-2.1972	-5.097699	-1.653456	0.66601	4.304565	-1.090339	1.005044	4.343247	-2.845336
50	-1.87975	-4.728046	0.066418	0.042648	3.012297	-2.154415	0.042676	3.01223	-2.154551
51	-2.611659	2.214139	-2.200255	3.350879	-2.502583	-1.64998	2.07004	-3.525727	-1.00531

52	-1.10871	2.986971	-1.702701	2.070131	-3.525606	-1.005766	3.350725	-2.502818	-1.649809
53	-1.913447	2.92396	0.732463	2.640026	-2.711618	1.357383	2.640296	-2.711821	1.357668
54	-3.549341	3.794929	2.092331	5.730708	-1.786521	2.418141	5.731128	-1.786479	2.417985
55	-4.762751	4.773952	1.238545	4.340342	-2.779186	2.904387	5.834898	-3.541011	2.314774
56	-5.154603	3.128377	1.726667	5.834369	-3.541074	2.31515	4.340927	-2.77931	2.904388
57	-5.045395	4.28932	-1.436764	6.1685	-3.595614	-0.408884	5.340186	-2.290245	-1.278237
58	-4.580787	2.659708	-1.956141	6.521331	-1.920181	-0.012374	6.167964	-3.595327	-0.410041
59	-5.838213	2.888989	-0.730362	5.340368	-2.291571	-1.278297	6.521644	-1.920344	-0.012352
60	6.354974	-2.214006	0.474581	-6.331006	-2.451807	-0.428769	-6.331131	-2.451728	-0.428961
61	7.496826	3.256662	0.280948	-7.432214	2.606109	1.682622	-7.432258	2.606184	1.682508
	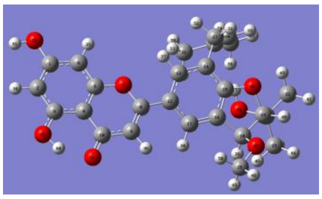 Conformer 4			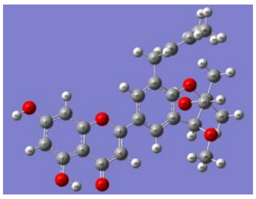 Conformer 5			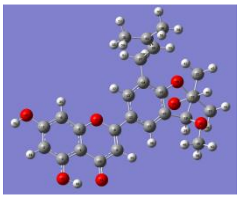 Conformer 6		
1	5.921578	1.813081	-0.007105	-6.175704	1.685895	-0.841063	-5.964428	1.063583	-1.102279
2	6.665632	0.668212	0.313267	-6.91731	0.587485	-0.382439	-6.638651	-0.025929	-0.531847
3	6.020422	-0.559618	0.431234	-6.253115	-0.536623	0.100131	-5.908612	-1.044588	0.074135
4	4.616235	-0.652391	0.230191	-4.832263	-0.571601	0.126283	-4.488965	-0.982069	0.112453
5	3.917215	0.523688	-0.090118	-4.136185	0.554982	-0.343235	-3.86204	0.130475	-0.473342
6	4.538232	1.757376	-0.21358	-4.776049	1.685795	-0.828046	-4.567983	1.157286	-1.081848
7	2.563064	0.493505	-0.287874	-2.767483	0.572785	-0.346617	-2.497622	0.238764	-0.470342
8	1.868481	-0.674145	-0.199155	-2.051637	-0.485974	0.123173	-1.719949	-0.711214	0.118536
9	2.491418	-1.852329	0.088913	-2.668541	-1.600704	0.609755	-2.268887	-1.804656	0.720853
10	3.909335	-1.915273	0.336647	-4.103826	-1.721283	0.629706	-3.693413	-2.018301	0.744151
11	4.504536	-2.989998	0.615127	-4.694784	-2.745543	1.064233	-4.221436	-3.026395	1.284523
12	0.438312	-0.495042	-0.454926	-0.607081	-0.260963	0.046806	-0.292522	-0.398046	0.027162
13	-0.027778	0.679205	-1.078799	-0.101572	1.041878	-0.14072	0.125113	0.903105	-0.318446
14	-1.379728	0.903982	-1.341201	1.26396	1.312114	-0.220572	1.470333	1.255707	-0.423706
15	-2.248245	-0.13041	-0.98793	2.114397	0.209293	-0.091658	2.390924	0.24106	-0.150526
16	-1.827886	-1.287842	-0.33193	1.651161	-1.091641	0.108743	2.015476	-1.053772	0.208558
17	-0.487093	-1.479543	-0.051229	0.290048	-1.342069	0.156716	0.675091	-1.391647	0.280403
18	-3.054949	-2.113359	-0.023202	2.847341	-2.00992	0.208116	3.271592	-1.857454	0.450541
19	-4.19941	-1.1248	-0.346512	4.031289	-1.048813	-0.062001	4.383648	-0.884915	-0.011113
20	-3.594255	-0.071921	-1.164915	3.464954	0.293969	-0.188182	3.736025	0.409082	-0.240509
21	-4.871687	-0.419173	0.85247	4.836328	-1.347546	-1.336589	5.108669	-1.294662	-1.304771
22	-5.993336	0.495772	0.34273	5.897211	-0.265314	-1.568438	6.067809	-0.187934	-1.757216
23	-5.424558	-1.429219	1.855454	5.488709	-2.725535	-1.238993	5.864928	-2.605911	-1.095959
24	-3.885662	0.357219	1.54793	3.93388	-1.401537	-2.451876	4.13134	-1.548984	-2.325772
25	-3.226619	-3.234471	-0.890272	3.063969	-2.570882	1.500367	3.523232	-2.145197	1.824701
26	-2.292211	-4.277146	-0.661535	2.164125	-3.61644	1.832205	2.665292	-3.135475	2.368868
27	-1.908374	2.218556	-1.875129	1.817797	2.708965	-0.40127	1.923506	2.655776	-0.779137
28	-2.976196	2.828901	-0.980776	2.416694	3.229155	0.883943	2.447392	3.388527	0.434468

29	-2.810694	3.257143	0.28136	3.709113	3.460284	1.156197	1.841834	4.36852	1.120222
30	-3.972129	3.806757	1.074051	4.1297	3.948224	2.520931	2.499041	4.97539	2.335601
31	-1.494815	3.216918	1.017664	4.845739	3.262632	0.187132	0.491702	4.949453	0.785406
32	6.729843	-1.659582	0.735976	-6.959581	-1.590652	0.542815	-6.550334	-2.089082	0.624798
33	6.505052	3.031785	-0.132337	-6.778608	2.803152	-1.320443	-6.632039	2.081102	-1.702581
34	7.73818	0.724114	0.470907	-8.002561	0.601311	-0.396972	-7.722225	-0.08667	-0.554362
35	3.973562	2.647933	-0.459224	-4.213729	2.539138	-1.185621	-4.058003	2.002698	-1.52623
36	1.927182	-2.774994	0.11919	-2.084286	-2.417825	1.01151	-1.638244	-2.532823	1.213215
37	0.68722	1.451479	-1.339689	-0.799286	1.866945	-0.230839	-0.626019	1.659226	-0.516812
38	-0.15953	-2.347967	0.508592	-0.0799	-2.356703	0.243277	0.376444	-2.410732	0.495965
39	-3.069591	-2.4534	1.019081	2.795407	-2.81431	-0.536392	3.273244	-2.790198	-0.126406
40	-4.947236	-1.624777	-0.964719	4.687013	-1.036939	0.812202	5.099334	-0.740697	0.801297
41	-6.418792	1.05122	1.183827	6.449985	-0.486555	-2.486403	5.527609	0.747696	-1.929879
42	-6.791811	-0.085193	-0.131205	6.606992	-0.224895	-0.73514	6.838562	-0.001105	-1.001882
43	-5.607873	1.210548	-0.389702	5.433085	0.720283	-1.665579	6.55809	-0.488486	-2.68796
44	-6.08963	-2.144268	1.360799	4.743513	-3.518057	-1.127506	5.188854	-3.425093	-0.83592
45	-5.995524	-0.902974	2.626273	6.060357	-2.921651	-2.150941	6.384758	-2.875191	-2.020111
46	-4.621684	-1.982876	2.349269	6.171598	-2.768439	-0.385126	6.60655	-2.500841	-0.298297
47	-3.605285	1.061878	0.935278	3.566626	-0.507858	-2.558027	3.717163	-0.696613	-2.540704
48	-2.602764	-5.1203	-1.282738	1.148526	-3.241689	2.014709	1.634875	-2.771737	2.475081
49	-2.289515	-4.586545	0.394298	2.539206	-4.075511	2.749846	3.055984	-3.381044	3.359032
50	-1.272868	-3.983367	-0.945018	2.123797	-4.375934	1.037587	2.657646	-4.042918	1.747055
51	-2.337056	2.06735	-2.87238	2.558999	2.703474	-1.205884	1.092823	3.191736	-1.244299
52	-1.064755	2.907351	-2.00164	1.001348	3.368055	-0.721383	2.722629	2.58923	-1.529037
53	-3.971052	2.898764	-1.415378	1.689991	3.392798	1.680331	3.413434	3.033723	0.792146
54	-3.763898	4.827892	1.419142	4.832414	3.24402	2.987616	3.471711	4.519425	2.544349
55	-4.895184	3.820858	0.486686	3.274554	4.073276	3.192321	2.646829	6.056181	2.204287
56	-4.150929	3.199844	1.97189	4.655872	4.910184	2.45083	1.865035	4.855048	3.224935
57	-0.650127	2.980891	0.368457	5.539498	2.500713	0.566097	0.563538	6.035597	0.639424
58	-1.299227	4.181297	1.502871	5.424447	4.189827	0.079638	0.040815	4.516961	-0.110204
59	-1.521564	2.462586	1.816125	4.519139	2.945429	-0.804239	-0.205841	4.794465	1.61976
60	6.065884	-2.410785	0.763015	-6.279977	-2.266477	0.83861	-5.832023	-2.681787	0.997261
61	7.459573	2.95533	0.031805	-7.743362	2.693373	-1.285764	-7.588313	1.91576	-1.657246
	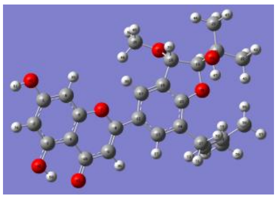 Conformer 7			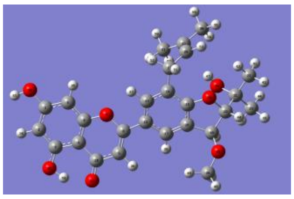 Conformer 8			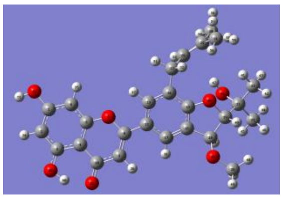 Conformer 9		
1	-6.061115	-1.768709	-0.007282	5.921598	1.81305	-0.007502	6.139778	1.925117	0.189628
2	-6.846494	-0.607185	-0.039483	6.665651	0.668264	0.313154	6.917268	0.765597	0.323358
3	-6.227875	0.638736	-0.093615	6.020443	-0.55954	0.431377	6.299825	-0.481823	0.299946
4	-4.809681	0.732985	-0.1172	4.616275	-0.652387	0.230265	4.890427	-0.579758	0.142413
5	-4.068938	-0.460246	-0.080959	3.917263	0.523613	-0.090347	4.157533	0.611691	0.010522

6	-4.662688	-1.712277	-0.026765	4.538265	1.757284	-0.214044	4.750359	1.865166	0.031108
7	-2.701039	-0.428043	-0.106874	2.56313	0.493372	-0.28817	2.797411	0.576194	-0.138847
8	-2.027303	0.754925	-0.148623	1.868498	-0.674222	-0.199101	2.127777	-0.608503	-0.183012
9	-2.688504	1.947448	-0.168948	2.491426	-1.852325	0.089338	2.783488	-1.799442	-0.077695
10	-4.127643	2.012874	-0.166696	3.909344	-1.915227	0.337015	4.211194	-1.861614	0.105053
11	-4.759109	3.102684	-0.194103	4.504494	-2.989899	0.615834	4.83661	-2.949716	0.214058
12	-0.575699	0.563701	-0.152157	0.438339	-0.495102	-0.454939	0.685672	-0.428907	-0.362627
13	0.285562	1.619377	-0.516315	-0.027724	0.679229	-1.078677	0.173921	0.816486	-0.784543
14	1.671898	1.483545	-0.536497	-1.379658	0.904034	-1.341123	-1.187102	1.035879	-0.994536
15	2.169666	0.230942	-0.158565	-2.248196	-0.130389	-0.987988	-2.024471	-0.061866	-0.777432
16	1.350301	-0.831742	0.219921	-1.82788	-1.287924	-0.332121	-1.557325	-1.283017	-0.299977
17	-0.027571	-0.68416	0.205548	-0.487097	-1.479665	-0.051445	-0.2057	-1.487116	-0.095583
18	2.233488	-2.004907	0.577613	-3.054982	-2.113374	-0.023428	-2.73541	-2.184444	-0.062937
19	3.647275	-1.47795	0.22929	-4.19938	-1.124721	-0.34657	-3.939491	-1.233385	-0.343158
20	3.490802	-0.076461	-0.160684	-3.594191	-0.071815	-1.164952	-3.370173	-0.030459	-0.957038
21	4.35353	-2.217004	-0.918527	-4.871535	-0.419142	0.852511	-4.751672	-0.74063	0.877826
22	5.678967	-1.529739	-1.266215	-5.993249	0.495788	0.342929	-5.949044	0.084535	0.389013
23	4.59055	-3.677892	-0.539478	-5.424246	-1.429199	1.855565	-5.23382	-1.899461	1.747665
24	3.488182	-2.228162	-2.064206	-3.885447	0.35726	1.547917	-3.899044	0.065419	1.701371
25	2.251706	-2.338465	1.963489	-3.226812	-3.234399	-0.890651	-2.679683	-3.282875	-0.966722
26	1.085562	-3.013945	2.407362	-2.292445	-4.27715	-0.662132	-3.581379	-4.327313	-0.629315
27	2.60506	2.610529	-0.922978	-1.908378	2.218603	-1.874979	-1.769974	2.399226	-1.288024
28	3.314921	3.170251	0.286796	-2.976229	2.828872	-0.980577	-2.467966	2.921799	-0.051685
29	4.617166	3.078299	0.592615	-2.810872	3.257032	0.281596	-3.740048	3.334743	0.069965
30	5.146871	3.670653	1.875569	-3.972414	3.80655	1.074201	-4.270033	3.811944	1.400317
31	5.659158	2.397917	-0.256666	-1.495029	3.216959	1.017965	-4.727239	3.411423	-1.066953
32	-6.975993	1.754755	-0.123223	6.729846	-1.65941	0.736459	7.040881	-1.59575	0.426362
33	-6.617919	-3.005157	0.043884	6.505064	3.031731	-0.132952	6.695887	3.162784	0.207497
34	-7.930432	-0.664346	-0.022405	7.738186	0.724213	0.470842	7.994512	0.825259	0.443526
35	-4.066576	-2.615793	-0.002395	3.973602	2.647782	-0.459902	4.160066	2.767102	-0.070587
36	-2.138183	2.878809	-0.169513	1.927186	-2.774969	0.119974	2.238351	-2.731325	-0.147613
37	-0.134816	2.570622	-0.826168	0.687284	1.451531	-1.339436	0.861869	1.639599	-0.941601
38	-0.682653	-1.507431	0.460113	-0.159554	-2.348209	0.508187	0.150603	-2.436543	0.288324
39	1.972851	-2.894287	-0.009584	-3.069623	-2.453592	1.018786	-2.753722	-2.562697	0.966075
40	4.264916	-1.489687	1.130654	-4.947272	-1.624572	-0.964786	-4.603393	-1.682327	-1.085876
41	5.511421	-0.491892	-1.56769	-6.418568	1.051268	1.18407	-5.620295	0.885664	-0.277082
42	6.358836	-1.527494	-0.407366	-6.791804	-0.085209	-0.130834	-6.663106	-0.54201	-0.156332
43	6.161327	-2.062829	-2.091008	-5.607911	1.210522	-0.389597	-6.461156	0.528781	1.248068
44	3.651034	-4.200997	-0.340596	-5.995246	-0.90297	2.626366	-5.897937	-1.515653	2.527879
45	5.096749	-4.188972	-1.363761	-4.621272	-1.982691	2.349397	-4.400679	-2.410727	2.236468
46	5.221887	-3.746781	0.351611	-6.089238	-2.144391	1.361011	-5.791663	-2.628564	1.151473
47	3.393744	-1.304693	-2.352398	-3.605086	1.061937	0.935282	-3.660363	0.850392	1.173855
48	0.854136	-3.875809	1.76424	-2.602908	-5.120073	-1.283687	-4.630607	-4.00564	-0.695364
49	0.212148	-2.349665	2.435451	-1.273057	-3.983284	-0.945331	-3.415125	-5.132639	-1.348035

50	1.292029	-3.366555	3.420582	-2.289928	-4.586942	0.393585	-3.394588	-4.702318	0.387243
51	2.015663	3.400836	-1.404157	-2.337164	2.067347	-2.872171	-0.95759	3.079835	-1.572837
52	3.321776	2.248315	-1.665934	-1.064828	2.907455	-2.001566	-2.45654	2.346835	-2.137517
53	2.657491	3.672641	0.997055	-3.971031	2.898809	-1.415293	-1.837091	2.943465	0.837594
54	4.356353	4.1476	2.46316	-4.151364	3.199521	1.971924	-3.497868	3.80171	2.175554
55	5.617321	2.896537	2.497598	-3.764198	4.827629	1.419455	-5.101749	3.177877	1.73628
56	5.924525	4.419724	1.672464	-4.895369	3.820756	0.486691	-4.66701	4.832802	1.321241
57	6.484004	3.088076	-0.478771	-1.522027	2.463318	1.817056	-4.867975	4.455953	-1.376933
58	5.267159	2.017395	-1.200993	-0.650415	2.980158	0.368955	-4.419311	2.837971	-1.943628
59	6.095688	1.549061	0.285776	-1.299157	4.181687	1.502369	-5.711572	3.047133	-0.750765
60	-6.324043	2.516244	-0.157563	6.065813	-2.410544	0.763709	6.391936	-2.358971	0.377945
61	-7.586196	-2.926384	0.048864	7.459543	2.955341	0.031422	7.657473	3.086291	0.323176
	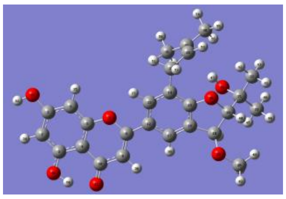 Conformer 10			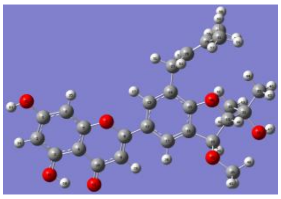 Conformer 11			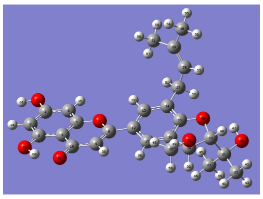 Conformer 12		
1	6.055486	1.687997	0.009524	6.274422	1.729876	-0.439483	-5.80358	-1.963497	0.137813
2	6.774279	0.518758	0.297804	6.983189	0.633432	0.072555	-6.586511	-0.808697	-0.005863
3	6.100987	-0.695938	0.392122	6.292945	-0.514178	0.452366	-5.965205	0.42589	-0.172276
4	4.693699	-0.750767	0.199349	4.878936	-0.574896	0.320503	-4.546707	0.51542	-0.19879
5	4.020532	0.448521	-0.088118	4.216047	0.550371	-0.197381	-3.808571	-0.670831	-0.050718
6	4.669786	1.669864	-0.187653	4.882216	1.704312	-0.582162	-4.404911	-1.911388	0.116827
7	2.664866	0.454524	-0.276935	2.855946	0.544221	-0.3505	-2.440364	-0.643318	-0.074343
8	1.942799	-0.697893	-0.209754	2.113551	-0.537597	0.01418	-1.76465	0.530314	-0.216806
9	2.539784	-1.896449	0.047797	2.696432	-1.653536	0.538868	-2.42266	1.717179	-0.346679
10	3.957532	-1.998568	0.282218	4.122757	-1.749218	0.714228	-3.862139	1.784586	-0.359675
11	4.528976	-3.093226	0.531597	4.683227	-2.77372	1.187275	-4.490634	2.86841	-0.489301
12	0.515599	-0.477947	-0.451844	0.681977	-0.334079	-0.215159	-0.312764	0.337182	-0.193928
13	0.073928	0.73205	-1.027102	0.180676	0.963355	-0.453839	0.552479	1.3591	-0.634845
14	-1.270569	0.99266	-1.290897	-1.173183	1.221938	-0.665804	1.939643	1.221445	-0.633196
15	-2.161867	-0.036377	-0.976909	-2.019872	0.108897	-0.634307	2.430743	0.004932	-0.152001
16	-1.76608	-1.214083	-0.348792	-1.556735	-1.184433	-0.392412	1.606606	-1.025909	0.299099
17	-0.430857	-1.456491	-0.087915	-0.210595	-1.423771	-0.190461	0.229697	-0.878278	0.269815
18	-2.99399	-2.021777	-0.03432	-2.726512	-2.128156	-0.444529	2.492567	-2.14749	0.793519
19	-4.141233	-1.043333	-0.434702	-3.923767	-1.138888	-0.530633	3.872804	-1.723104	0.250652
20	-3.500988	0.036795	-1.189133	-3.361809	0.173283	-0.826291	3.754863	-0.30638	-0.110504
21	-4.900422	-0.364833	0.726924	-4.773664	-1.032201	0.749274	4.395229	-2.478962	-0.985557
22	-5.972082	0.580085	0.167373	-3.948477	-0.572201	1.956645	4.782576	-3.904245	-0.597661
23	-5.53828	-1.393638	1.657648	-5.95423	-0.086453	0.515646	3.396766	-2.465741	-2.150128
24	-3.95572	0.377069	1.513321	-5.24272	-2.377769	0.934543	5.61178	-1.835384	-1.391336
25	-2.989236	-3.215184	-0.809358	-2.615046	-2.955667	-1.597705	2.60459	-2.196298	2.214717
26	-3.946049	-4.167048	-0.366241	-3.502939	-4.065915	-1.559647	1.434377	-2.670754	2.862879

27	-1.770665	2.329067	-1.796734	-1.722769	2.618896	-0.850716	2.873688	2.315356	-1.104645
28	-2.860273	2.923107	-0.917648	-2.535999	3.043726	0.34903	3.564924	2.986113	0.060214
29	-2.736018	3.295841	0.366518	-3.827956	3.401476	0.383827	3.30995	4.199081	0.571086
30	-3.916837	3.837327	1.136122	-4.487558	3.77344	1.68897	4.081192	4.707486	1.764353
31	-1.450464	3.200167	1.149826	-4.730156	3.489137	-0.820486	2.265856	5.153109	0.049696
32	6.786015	-1.819192	0.666153	6.967704	-1.566481	0.945785	-6.711171	1.535496	-0.307161
33	6.667324	2.894988	-0.092066	6.90352	2.869817	-0.822245	-6.361806	-3.189064	0.30405
34	7.848787	0.545927	0.449227	8.062925	0.666271	0.178333	-7.670587	-0.861612	0.012121
35	4.124773	2.57895	-0.408578	4.345356	2.556468	-0.979518	-3.810675	-2.809655	0.227602
36	1.95439	-2.806124	0.064792	2.089408	-2.492579	0.851667	-1.870752	2.644147	-0.426293
37	0.805393	1.498867	-1.256149	0.872511	1.798138	-0.458825	0.135097	2.281394	-1.025262
38	-0.129337	-2.368668	0.414741	0.143877	-2.436767	-0.035944	-0.426368	-1.67729	0.591326
39	-3.043246	-2.278743	1.030557	-2.809257	-2.759986	0.447483	2.178642	-3.131948	0.422299
40	-4.839715	-1.533429	-1.117006	-4.570933	-1.404116	-1.369925	4.618633	-1.777572	1.045241
41	-6.73372	0.025697	-0.391576	-3.555197	0.435971	1.796125	5.244213	-4.40048	-1.456665
42	-5.52527	1.319547	-0.502747	-3.113468	-1.250076	2.155786	3.908803	-4.488091	-0.29423
43	-6.460409	1.105703	0.993473	-4.585287	-0.548081	2.848303	5.503358	-3.897259	0.226163
44	-6.215203	-2.051236	1.102979	-5.598227	0.922005	0.292198	3.1539	-1.440062	-2.445786
45	-4.786893	-2.008865	2.15928	-6.566995	-0.439276	-0.320416	3.846547	-2.970921	-3.009861
46	-6.117819	-0.87518	2.427286	-6.584444	-0.03673	1.411312	2.46309	-2.979349	-1.899438
47	-3.62218	1.091641	0.940272	-5.810116	-2.380958	1.722391	5.387958	-0.897112	-1.518252
48	-4.975995	-3.796334	-0.467942	-4.552594	-3.751013	-1.490813	0.603108	-1.958573	2.780469
49	-3.826848	-5.051775	-0.995315	-3.352386	-4.621662	-2.487788	1.689054	-2.797028	3.917504
50	-3.776678	-4.4422	0.684798	-3.282901	-4.719205	-0.702868	1.113665	-3.638274	2.449001
51	-0.916962	3.012598	-1.876234	-0.879582	3.307493	-0.991179	3.631871	1.872396	-1.763861
52	-2.169664	2.214006	-2.811138	-2.321633	2.661822	-1.765206	2.309079	3.033055	-1.70445
53	-3.836033	3.031624	-1.386372	-1.984958	3.026859	1.289919	4.327971	2.376447	0.542821
54	-3.702784	4.839597	1.529646	-5.324547	3.095921	1.90741	4.605103	5.642936	1.524376
55	-4.815869	3.893753	0.514972	-3.786251	3.730698	2.527963	4.820777	3.979998	2.112663
56	-4.142613	3.199879	2.001668	-4.910615	4.78636	1.644175	3.404571	4.936483	2.59924
57	-0.58684	2.967984	0.524556	-4.283081	3.078014	-1.727309	1.739064	4.783391	-0.832466
58	-1.253304	4.142467	1.676149	-5.669886	2.953132	-0.637275	2.719251	6.120232	-0.205581
59	-1.52347	2.419517	1.919605	-5.004141	4.534614	-1.018154	1.516381	5.357512	0.826379
60	6.104269	-2.554719	0.680844	6.27338	-2.261378	1.148863	-6.058139	2.28987	-0.408046
61	7.621012	2.791506	0.061983	7.860043	2.778593	-0.678054	-7.330078	-3.11011	0.304904
	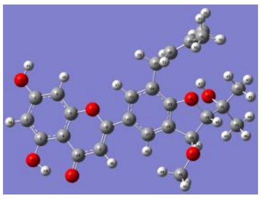 Conformer 13			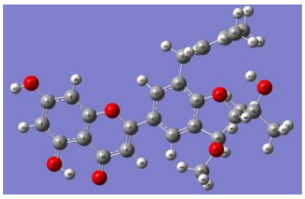 Conformer 14			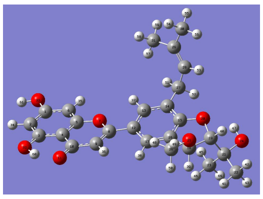 Conformer 15		
1	6.00702	2.057987	0.182955	6.166778	1.835561	-0.423032	-5.924952	1.059524	-1.117116
2	6.809857	0.92085	0.353092	6.886321	0.768048	0.133235	-6.605882	-0.020982	-0.537563
3	6.223656	-0.339488	0.348934	6.210906	-0.381424	0.533272	-5.882503	-1.035899	0.082459

4	4.817302	-0.476142	0.175593	4.800826	-0.473007	0.377837	-4.462717	-0.978687	0.125894
5	4.057957	0.69212	0.007943	4.126679	0.624745	-0.183165	-3.829007	0.124856	-0.469675
6	4.621751	1.961403	0.007034	4.777997	1.779728	-0.589025	-4.528185	1.147736	-1.092227
7	2.701383	0.623082	-0.155825	2.769704	0.58825	-0.360306	-2.464146	0.227707	-0.462158
8	2.059904	-0.577995	-0.179678	2.042169	-0.497763	0.020025	-1.692964	-0.71853	0.140524
9	2.742415	-1.750285	-0.042807	2.635169	-1.587641	0.585115	-2.248066	-1.803236	0.752076
10	4.169038	-1.774616	0.158109	4.060049	-1.650564	0.789857	-3.673975	-2.011001	0.772033
11	4.817996	-2.844972	0.297679	4.629858	-2.6507	1.301449	-4.20759	-3.011184	1.321019
12	0.615883	-0.438264	-0.375078	0.610685	-0.327549	-0.240737	-0.263516	-0.410693	0.052191
13	0.07731	0.775	-0.848376	0.087323	0.957248	-0.492688	0.158595	0.88413	-0.310501
14	-1.290107	0.955265	-1.059324	-1.271255	1.18353	-0.717543	1.505249	1.233637	-0.415375
15	-2.101096	-0.152381	-0.803117	-2.086213	0.050218	-0.702404	2.42162	0.222263	-0.122622
16	-1.606789	-1.354352	-0.296151	-1.604182	-1.237941	-0.460729	2.041204	-1.06836	0.25002
17	-0.250952	-1.507202	-0.06759	-0.256459	-1.439536	-0.22208	0.700292	-1.402388	0.32634
18	-2.780792	-2.274872	-0.06459	-2.778527	-2.190758	-0.543932	3.297889	-1.860118	0.534927
19	-3.983836	-1.323997	-0.264712	-3.969609	-1.213344	-0.484499	4.391291	-0.9369	-0.041177
20	-3.452188	-0.142753	-0.948235	-3.433402	0.091953	-0.873383	3.770658	0.381248	-0.207775
21	-4.705305	-0.805311	1.002039	-4.660868	-1.036301	0.884878	4.998391	-1.342809	-1.397416
22	-5.954463	-0.022688	0.576709	-5.525417	-2.255999	1.190005	5.883123	-2.574968	-1.221961
23	-5.090918	-1.947898	1.93911	-3.674186	-0.747878	2.024152	3.932131	-1.566237	-2.47737
24	-3.815939	0.045938	1.734825	-5.557703	0.077606	0.75436	5.874498	-0.281544	-1.803031
25	-2.908383	-3.303043	-1.047712	-2.863774	-2.873769	-1.792691	3.569959	-2.002324	1.927934
26	-1.917021	-4.312661	-0.945592	-1.860113	-3.861745	-1.969395	2.697693	-2.90488	2.590242
27	-1.9035	2.298445	-1.382958	-1.869165	2.565436	-0.87481	1.96168	2.62687	-0.792061
28	-2.570632	2.854236	-0.144114	-2.730828	2.912471	0.317355	2.469811	3.383117	0.413894
29	-3.847201	3.243743	0.005828	-4.051576	3.150787	0.344754	1.851105	4.370281	1.077246
30	-4.341728	3.763682	1.333731	-4.752663	3.439989	1.651062	2.492553	5.002162	2.288229
31	-4.874447	3.25166	-1.097702	-4.951206	3.160488	-0.866779	0.500202	4.935752	0.719906
32	6.988173	-1.433764	0.509363	6.896144	-1.406059	1.068473	-6.530811	-2.071668	0.641753
33	6.643811	3.256222	0.198447	6.781667	2.975274	-0.82809	-6.585896	2.073121	-1.731008
34	7.88002	1.026846	0.486445	7.963077	0.825215	0.257582	-7.689598	-0.077643	-0.563857
35	3.997234	2.838076	-0.124485	4.232799	2.609785	-1.020311	-4.012996	1.986204	-1.543698
36	2.219941	-2.695918	-0.101057	2.038519	-2.429565	0.909947	-1.622538	-2.52891	1.254527
37	0.745674	1.60781	-1.036613	0.759964	1.807493	-0.483313	-0.590178	1.637916	-0.525596
38	0.132963	-2.41748	0.378372	0.123683	-2.435624	-0.027584	0.399954	-2.416993	0.56065
39	-2.757917	-2.729416	0.932774	-2.792481	-2.92333	0.273996	3.292278	-2.852213	0.064288
40	-4.699425	-1.794496	-0.941582	-4.716131	-1.477454	-1.234674	5.193723	-0.810211	0.68726
41	-6.695993	-0.683948	0.115665	-6.074534	-2.091169	2.121971	5.296276	-3.450603	-0.929847
42	-5.695671	0.755353	-0.145663	-6.24914	-2.425938	0.386526	6.647888	-2.394677	-0.459809
43	-6.405955	0.448517	1.455072	-4.914304	-3.156014	1.307245	6.384302	-2.799122	-2.168438
44	-5.741912	-1.567868	2.732165	-4.240001	-0.595028	2.947965	4.428374	-1.800102	-3.423766
45	-4.208735	-2.389801	2.409501	-3.100859	0.162426	1.823305	3.327428	-0.665368	-2.622771
46	-5.629292	-2.732179	1.397116	-2.970292	-1.570669	2.183754	3.25917	-2.392936	-2.22836
47	-3.639501	0.822347	1.171213	-4.998772	0.816568	0.456269	5.335306	0.528287	-1.792886

48	-0.920491	-3.937279	-1.213406	-2.104507	-4.396554	-2.889819	1.672785	-2.515603	2.64842
49	-2.197537	-5.101612	-1.647119	-0.861491	-3.416997	-2.070485	3.08639	-3.03075	3.603159
50	-1.875253	-4.730215	0.071481	-1.847691	-4.572304	-1.129657	2.676741	-3.882202	2.085656
51	-2.614923	2.205783	-2.207915	-1.051843	3.29174	-0.964461	1.136479	3.152893	-1.277778
52	-1.111241	2.980799	-1.716128	-2.443537	2.616695	-1.80442	2.769961	2.54629	-1.530675
53	-1.910301	2.925424	0.72089	-2.190242	2.936499	1.263991	3.43472	3.040894	0.78669
54	-5.148968	3.128378	1.722648	-5.503048	2.667098	1.866768	1.850746	4.892619	3.173329
55	-4.761421	4.772969	1.22784	-4.051166	3.473931	2.49005	2.634329	6.081519	2.139774
56	-3.543996	3.799095	2.081843	-5.289596	4.396833	1.608646	3.466169	4.556464	2.513986
57	-5.048607	4.278845	-1.44618	-5.796364	2.472276	-0.731519	0.060867	4.48519	-0.172559
58	-4.584653	2.647079	-1.959441	-5.388204	4.157924	-1.007652	-0.204099	4.789513	1.550129
59	-5.839028	2.88137	-0.73152	-4.438967	2.878772	-1.788044	0.565769	6.019799	0.556547
60	6.358678	-2.212743	0.469092	6.212043	-2.108583	1.27868	-5.816938	-2.663473	1.023575
61	5.997643	3.971152	0.074927	7.737326	2.904709	-0.667615	-7.543065	1.912509	-1.687323
	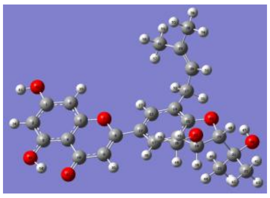 Conformer 16			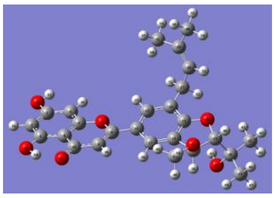 Conformer 17			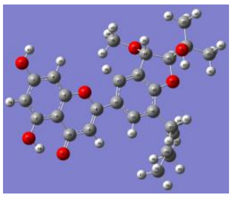 Conformer 18		
1	-5.961259	1.22207	-0.372625	-5.968752	1.047573	-1.1508	-5.814839	-2.005588	0.076949
2	-6.610716	-0.012842	-0.230811	-6.645711	-0.024482	-0.551432	-6.609196	-0.853861	-0.019246
3	-5.856374	-1.171587	-0.070193	-5.920176	-1.023334	0.087201	-6.001524	0.389873	-0.14597
4	-4.436672	-1.103534	-0.048749	-4.498707	-0.960855	0.131168	-4.582278	0.494826	-0.179499
5	-3.834806	0.157441	-0.196501	-3.86851	0.132999	-0.481687	-3.83208	-0.686683	-0.078332
6	-4.565277	1.325065	-0.357928	-4.572473	1.143518	-1.123936	-4.416953	-1.939945	0.049177
7	-2.471765	0.281204	-0.177935	-2.504785	0.242639	-0.477124	-2.465131	-0.647683	-0.109924
8	-1.670212	-0.810006	-0.035554	-1.729407	-0.693411	0.137286	-1.799868	0.536121	-0.218459
9	-2.192758	-2.062288	0.096576	-2.281011	-1.769416	0.76727	-2.470109	1.720346	-0.303678
10	-3.615592	-2.289167	0.109761	-3.706016	-1.980508	0.793322	-3.909943	1.775116	-0.302716
11	-4.119855	-3.436172	0.239165	-4.236231	-2.97268	1.359793	-4.548995	2.856969	-0.390144
12	-0.24752	-0.460946	-0.05428	-0.301161	-0.387012	0.039347	-0.346711	0.356277	-0.212383
13	0.161854	0.806952	-0.514017	0.121963	0.912516	-0.305316	0.504287	1.395666	-0.640309
14	1.503514	1.183288	-0.585396	1.468786	1.257442	-0.415713	1.891953	1.270977	-0.651647
15	2.427009	0.23165	-0.149844	2.384585	0.236095	-0.150639	2.399498	0.049547	-0.197512
16	2.057703	-1.024534	0.335152	2.003409	-1.057403	0.206798	1.590949	-0.996306	0.244408
17	0.723027	-1.387553	0.378106	0.661272	-1.3872	0.285209	0.211989	-0.862756	0.221528
18	3.314587	-1.737957	0.781513	3.255828	-1.870019	0.437687	2.487124	-2.131175	0.683187
19	4.415972	-0.851932	0.163987	4.371273	-0.902413	-0.026195	3.890917	-1.629388	0.265272
20	3.774303	0.422154	-0.176541	3.730205	0.396089	-0.248256	3.726095	-0.241499	-0.173421
21	5.104582	-1.384718	-1.106782	5.087358	-1.311703	-1.324926	4.549773	-2.418928	-0.878299
22	6.004574	-2.566412	-0.752826	6.052012	-0.209698	-1.777188	5.858559	-1.74787	-1.310145
23	4.104128	-1.751458	-2.210315	5.835482	-2.629035	-1.125558	4.805957	-3.861899	-0.445856
24	5.979387	-0.350115	-1.578805	4.103247	-1.554584	-2.342226	3.637695	-2.48252	-1.985484

25	3.508574	-1.711662	2.194376	3.513849	-2.166063	1.808839	2.529962	-2.338065	2.093481
26	2.621119	-2.558189	2.908807	2.653624	-3.154393	2.352967	1.366036	-2.956558	2.618552
27	1.946153	2.544775	-1.077225	1.929355	2.655834	-0.768373	2.811955	2.384281	-1.105647
28	2.379493	3.4282	0.070206	2.478365	3.375317	0.442125	3.503264	3.037597	0.068904
29	1.702416	4.450873	0.611568	1.892889	4.356447	1.143489	3.246846	4.241443	0.600253
30	2.27086	5.215009	1.782081	2.574172	4.948085	2.353114	4.019205	4.731515	1.800508
31	0.350993	4.93205	0.148522	0.543857	4.953086	0.832845	2.200305	5.202389	0.096643
32	-6.474646	-2.357166	0.064276	-6.563578	-2.052616	0.665366	-6.757232	1.498162	-0.236851
33	-6.653735	2.378237	-0.530859	-6.73969	1.985106	-1.757759	-6.472931	-3.186243	0.198014
34	-7.693927	-0.079636	-0.244802	-7.727726	-0.071433	-0.587736	-7.689451	-0.935811	0.005597
35	-4.074312	2.283868	-0.467278	-4.043687	1.970229	-1.585446	-3.798607	-2.827774	0.121958
36	-1.541941	-2.922359	0.179842	-1.651522	-2.48394	1.280807	-1.92683	2.654395	-0.35594
37	-0.591794	1.511299	-0.847215	-0.626021	1.673427	-0.497792	0.074409	2.321702	-1.007744
38	0.432527	-2.365213	0.744124	0.356386	-2.405016	0.498342	-0.435557	-1.674474	0.52747
39	3.362159	-2.776824	0.429259	3.248008	-2.799973	-0.143731	2.220979	-3.070334	0.182834
40	5.175091	-0.623854	0.913811	5.091738	-0.765212	0.783209	4.542072	-1.60862	1.142129
41	6.722237	-2.284524	0.024187	5.517562	0.730473	-1.94289	5.678219	-0.720713	-1.640973
42	5.421771	-3.421583	-0.398739	6.827817	-0.031764	-1.024891	6.576718	-1.717785	-0.483763
43	6.561811	-2.876512	-1.641984	6.535502	-0.509437	-2.711738	6.302002	-2.311119	-2.136647
44	3.436222	-2.564537	-1.908393	5.155096	-3.444628	-0.865505	3.8764	-4.378294	-0.190813
45	4.656421	-2.072073	-3.098453	6.348683	-2.897951	-2.053514	5.283361	-4.406556	-1.265765
46	3.489372	-0.888013	-2.484152	6.581912	-2.532513	-0.331311	5.469762	-3.891709	0.423528
47	5.423869	0.441421	-1.685686	3.693592	-0.698382	-2.550592	3.543641	-1.577422	-2.326673
48	2.644958	-3.585032	2.51486	2.637159	-4.058473	1.726466	1.11702	-3.873975	2.064943
49	2.959986	-2.56314	3.947153	1.625919	-2.785463	2.467315	0.498499	-2.284356	2.596208
50	1.587359	-2.190434	2.874181	3.048837	-3.407372	3.339462	1.586677	-3.212204	3.657521
51	2.789871	2.414535	-1.767471	1.096739	3.202537	-1.2173	2.236181	3.108674	-1.686556
52	1.13299	2.998797	-1.648396	2.716532	2.587284	-1.530711	3.5709	1.963931	-1.778546
53	3.337391	3.156059	0.512223	3.446053	3.00869	0.782879	4.268624	2.421679	0.539724
54	3.246979	4.82635	2.088168	3.545101	4.480817	2.5442	4.760773	3.999759	2.135465
55	1.594845	5.166287	2.646924	2.731038	6.028233	2.227153	4.540991	5.671823	1.575267
56	2.38566	6.280083	1.537935	1.952208	4.827809	3.250915	3.34365	4.945377	2.64028
57	-0.031694	4.388892	-0.718078	-0.143377	4.797605	1.675631	1.45236	5.393554	0.878177
58	-0.385153	4.832708	0.957826	0.076432	4.532638	-0.060015	1.671956	4.846217	-0.790141
59	0.387576	5.999289	-0.107883	0.624205	6.039699	0.695093	2.651939	6.174044	-0.144226
60	-5.74189	-3.034841	0.161865	-5.847469	-2.634335	1.057279	-6.111552	2.260779	-0.316243
61	-7.607251	2.191646	-0.522639	-6.174702	2.67515	-2.143016	-5.831516	-3.913865	0.253483
	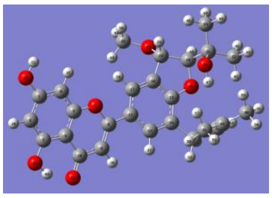			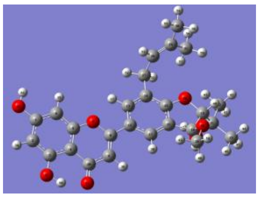			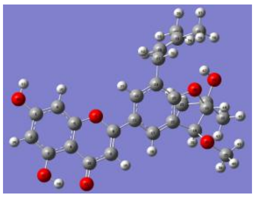		
	Conformer 19			Conformer 20			Conformer 21		
1	-5.698804	1.660193	1.224669	-6.170504	1.804977	-0.167276	6.144234	1.927931	0.215491

2	-6.554914	0.619849	0.835401	-6.909505	0.613597	-0.12958	6.921257	0.765744	0.326508
3	-6.031921	-0.492508	0.186349	-6.243678	-0.605684	-0.083316	6.303762	-0.478867	0.284592
4	-4.636257	-0.576908	-0.080752	-4.820874	-0.645997	-0.072581	4.891637	-0.574307	0.131843
5	-3.821886	0.490262	0.328056	-4.127296	0.573013	-0.113092	4.15891	0.617667	0.024691
6	-4.321771	1.611306	0.977975	-4.770743	1.803063	-0.1602	4.75392	1.871959	0.063298
7	-2.472816	0.461867	0.103133	-2.759682	0.596183	-0.099569	2.79857	0.587861	-0.117447
8	-1.89189	-0.588673	-0.540583	-2.038791	-0.559026	-0.065429	2.126395	-0.594821	-0.178982
9	-2.630306	-1.647369	-0.979676	-2.653225	-1.776226	-0.04069	2.781491	-1.787592	-0.098498
10	-4.053315	-1.716692	-0.764479	-4.088542	-1.898311	-0.031153	4.210533	-1.854513	0.075287
11	-4.752622	-2.690311	-1.151042	-4.676053	-3.012147	0.000175	4.834177	-2.945211	0.161926
12	-0.446737	-0.413381	-0.694394	-0.595165	-0.314105	-0.070043	0.682977	-0.408431	-0.346001
13	0.376381	-1.515095	-1.003878	-0.094666	0.955302	-0.42596	0.172556	0.848703	-0.734464
14	1.757004	-1.400813	-1.159178	1.270002	1.236268	-0.480203	-1.189578	1.081065	-0.924452
15	2.278634	-0.110267	-1.034218	2.124727	0.183769	-0.137952	-2.028402	-0.014866	-0.706431
16	1.502858	0.994071	-0.685337	1.665359	-1.076073	0.248824	-1.560644	-1.260662	-0.291862
17	0.137488	0.85553	-0.503291	0.30689	-1.342375	0.269951	-0.206961	-1.475971	-0.113428
18	2.426806	2.182438	-0.554742	2.864742	-1.930014	0.589935	-2.74104	-2.182451	-0.129804
19	3.820885	1.514052	-0.600127	4.048415	-1.023749	0.172846	-3.9295	-1.185833	-0.239831
20	3.601133	0.175941	-1.153328	3.477212	0.278427	-0.172443	-3.379377	0.031838	-0.837446
21	4.545311	1.319375	0.750303	4.860982	-1.525822	-1.032176	-4.61287	-0.770773	1.08083
22	5.912535	0.666034	0.507502	5.903694	-0.482444	-1.449113	-5.48428	-1.912042	1.5966
23	4.714358	2.645863	1.487612	5.536707	-2.854695	-0.697562	-3.61327	-0.299563	2.14562
24	3.743576	0.477291	1.591049	3.963341	-1.789209	-2.121567	-5.503416	0.312071	0.768819
25	2.369794	3.083399	-1.660695	3.025073	-2.194944	1.981938	-2.724904	-3.143048	-1.179028
26	1.170156	3.838736	-1.716647	2.105223	-3.144923	2.49597	-3.638413	-4.210251	-0.963952
27	2.670227	-2.595961	-1.334171	1.816602	2.596076	-0.858115	-1.762553	2.439113	-1.269
28	3.769893	-2.656615	-0.285487	2.357004	3.322113	0.350872	-2.585913	2.978633	-0.122372
29	3.603128	-2.818903	1.037192	3.634935	3.613694	0.633265	-3.898713	3.25747	-0.104039
30	4.786013	-2.821381	1.975379	3.994773	4.315316	1.920023	-4.562052	3.749294	1.160545
31	2.259306	-2.992482	1.699963	4.812244	3.28485	-0.247658	-4.825065	3.122472	-1.287642
32	-6.847665	-1.492312	-0.191313	-6.946248	-1.751553	-0.04877	7.043215	-1.596911	0.388325
33	-6.276352	2.715454	1.852869	-6.884859	2.958093	-0.21071	6.811182	3.108716	0.26381
34	-7.617086	0.685655	1.039721	-7.992638	0.646288	-0.136689	7.99601	0.840375	0.443212
35	-3.655399	2.412455	1.278052	-4.194055	2.720865	-0.188621	4.148936	2.767762	-0.022008
36	-2.158147	-2.457089	-1.519851	-2.067809	-2.685928	-0.043747	2.236079	-2.718282	-0.180429
37	-0.06243	-2.505326	-1.07069	-0.795143	1.740061	-0.688461	0.864202	1.669327	-0.887718
38	-0.47773	1.696403	-0.208328	-0.055435	-2.324162	0.55057	0.152217	-2.442201	0.222516
39	2.252638	2.736696	0.375242	2.855886	-2.876291	0.035328	-2.742642	-2.703652	0.836628
40	4.459436	2.052592	-1.302711	4.700465	-0.864571	1.034869	-4.688477	-1.547482	-0.936108
41	6.565989	1.323504	-0.075862	6.463089	-0.850853	-2.314252	-6.215516	-2.211761	0.838884
42	5.801226	-0.276712	-0.03544	6.609954	-0.2843	-0.635741	-4.880596	-2.783929	1.8657
43	6.392774	0.461836	1.469095	5.422418	0.462597	-1.71657	-6.026373	-1.583717	2.488502
44	5.330535	2.492154	2.378535	6.21453	-2.738786	0.15366	-2.916526	-1.089579	2.442727
45	3.751115	3.050419	1.809128	6.117818	-3.193391	-1.560365	-4.168729	0.018892	3.032676

46	5.208415	3.385027	0.848954	4.804638	-3.63012	-0.455423	-3.033009	0.554871	1.783797
47	3.718851	-0.398342	1.163156	3.599868	-0.93217	-2.400496	-4.942925	0.982267	0.340287
48	0.301754	3.211229	-1.956411	2.092541	-4.059788	1.885302	-3.516104	-4.903665	-1.798588
49	1.300985	4.579345	-2.508926	1.085673	-2.741545	2.549198	-4.681113	-3.86329	-0.940407
50	0.979281	4.35661	-0.764799	2.438874	-3.391809	3.50654	-3.42294	-4.73332	-0.020896
51	3.138246	-2.560007	-2.324547	2.590452	2.475724	-1.622027	-2.359686	2.367704	-2.182943
52	2.060131	-3.506814	-1.313153	1.007353	3.182651	-1.310353	-0.932719	3.123834	-1.483989
53	4.784161	-2.535224	-0.659477	1.596802	3.596579	1.08293	-2.022922	3.12146	0.800455
54	4.829665	-3.75627	2.549157	4.512852	5.263482	1.720785	-5.324699	3.034652	1.499111
55	4.703905	-2.005645	2.706299	3.111957	4.528548	2.530636	-3.840675	3.884115	1.972086
56	5.731604	-2.699785	1.438424	4.685746	3.704364	2.517357	-5.077655	4.704005	0.992154
57	1.997345	-2.099438	2.284002	4.52945	2.818363	-1.19246	-5.223334	4.105333	-1.572657
58	1.452623	-3.16904	0.986657	5.390288	4.191732	-0.470191	-5.695657	2.502443	-1.034746
59	2.28699	-3.832444	2.405141	5.49306	2.596384	0.26992	-4.346773	2.681158	-2.163472
60	-6.257831	-2.170808	-0.634588	-6.264875	-2.48634	-0.021474	6.393884	-2.358132	0.329495
61	-5.598537	3.374412	2.077185	-6.2799	3.718061	-0.23019	6.181649	3.843637	0.176772
	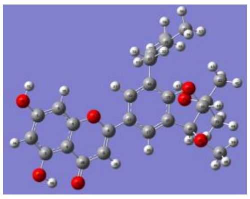 Conformer 22			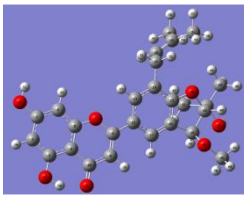 Conformer 23			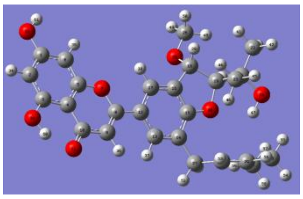 Conformer 24		
1	6.275719	1.724294	-0.46865	6.160485	1.904907	0.194608	-6.172999	1.696459	0.177521
2	6.985299	0.643636	0.075004	6.931221	0.739339	0.31352	-6.91847	0.518446	0.330715
3	6.298064	-0.495806	0.476955	6.306383	-0.502021	0.284459	-6.269844	-0.710831	0.314757
4	4.883314	-0.567037	0.336851	4.893387	-0.590663	0.13688	-4.858211	-0.774829	0.142593
5	4.219459	0.540514	-0.212121	4.167061	0.604288	0.021321	-4.157465	0.43188	-0.004947
6	4.884722	1.689303	-0.620577	4.769482	1.855544	0.046921	-4.78376	1.671408	0.008186
7	2.861475	0.526917	-0.375993	2.806309	0.581057	-0.116436	-2.800633	0.431731	-0.178559
8	2.119534	-0.548988	0.007647	2.126373	-0.598243	-0.164994	-2.095645	-0.733602	-0.194235
9	2.702851	-1.649709	0.562225	2.77596	-1.794013	-0.075332	-2.715237	-1.938139	-0.041093
10	4.128423	-1.734995	0.752006	4.204528	-1.867476	0.094058	-4.143194	-2.037415	0.126659
11	4.688314	-2.745782	1.253453	4.822999	-2.96083	0.188675	-4.735986	-3.140354	0.261657
12	0.689404	-0.35472	-0.240178	0.684358	-0.404984	-0.328315	-0.659593	-0.506307	-0.375052
13	0.188487	0.936313	-0.512487	0.176899	0.853991	-0.714094	0.197328	-1.575184	-0.714968
14	-1.162924	1.181954	-0.752442	-1.184095	1.093817	-0.901387	1.57081	-1.412762	-0.8855
15	-2.000774	0.063438	-0.733622	-2.032699	0.003847	-0.680988	2.055633	-0.113344	-0.705958
16	-1.545999	-1.213358	-0.417473	-1.564167	-1.246254	-0.276995	1.241926	0.960045	-0.352988
17	-0.203687	-1.443111	-0.18176	-0.211476	-1.468151	-0.100641	-0.121232	0.783404	-0.195582
18	-2.722832	-2.146671	-0.395203	-2.739654	-2.170887	-0.121975	2.103574	2.190002	-0.23243
19	-3.921739	-1.168296	-0.589814	-3.935929	-1.193685	-0.307755	3.530456	1.57475	-0.289115
20	-3.334896	0.116685	-0.980492	-3.382246	0.055743	-0.815748	3.364901	0.2249	-0.830633
21	-4.802571	-0.872565	0.647119	-4.734384	-0.891059	0.973869	4.280959	1.432233	1.05244
22	-5.983684	0.011495	0.226453	-3.863181	-0.250727	2.060596	4.776918	2.797579	1.51906

23	-5.313151	-2.153284	1.303653	-5.930143	0.006566	0.646914	3.441539	0.738343	2.133595
24	-4.004512	-0.197348	1.627931	-5.188185	-2.191659	1.383239	5.451408	0.640495	0.794618
25	-2.602017	-3.087081	-1.456973	-2.687771	-3.172955	-1.13213	1.830509	3.055622	-1.32795
26	-3.509284	-4.174105	-1.342173	-3.57936	-4.251151	-0.876993	2.394543	4.349645	-1.163733
27	-1.743833	2.572261	-0.871905	-1.735565	2.457123	-1.254631	2.516257	-2.558028	-1.177717
28	-2.505924	2.906154	0.391664	-2.484971	3.054491	-0.087247	3.441215	-2.797512	-0.006801
29	-3.787065	3.29093	0.51047	-3.771803	3.428708	-0.038902	4.777754	-2.680083	0.030671
30	-4.384954	3.564009	1.869325	-4.362742	3.986736	1.232364	5.533505	-2.914409	1.31716
31	-4.72064	3.530172	-0.64888	-4.73262	3.359504	-1.198258	5.644777	-2.319697	-1.150671
32	6.972201	-1.534451	1.000959	7.039593	-1.623547	0.395858	-6.978432	-1.843798	0.462718
33	7.009268	2.804347	-0.839187	6.834421	3.08236	0.230745	-6.869664	2.860726	0.202089
34	8.062312	0.699606	0.180803	8.006736	0.808786	0.426389	-7.993116	0.569087	0.460565
35	4.331325	2.52103	-1.042236	4.169279	2.75397	-0.044542	-4.20283	2.579148	-0.110961
36	2.097638	-2.485072	0.888057	2.22514	-2.722438	-0.145757	-2.139386	-2.853753	-0.026305
37	0.874819	1.775604	-0.510564	0.871304	1.671445	-0.872234	-0.216627	-2.567692	-0.858943
38	0.146626	-2.447068	0.03006	0.141754	-2.44006	0.225413	-0.762817	1.613759	0.073091
39	-2.793884	-2.683979	0.557903	-2.781016	-2.651839	0.862299	1.938038	2.733369	0.707389
40	-4.540459	-1.499301	-1.427407	-4.615782	-1.575287	-1.073189	4.165669	2.108666	-0.998379
41	-6.66123	-0.52855	-0.443683	-3.018594	-0.892105	2.32869	3.942767	3.466933	1.749764
42	-6.544218	0.318599	1.114736	-3.480205	0.718726	1.728526	5.378603	2.677546	2.424993
43	-5.631136	0.905652	-0.293133	-4.463395	-0.08599	2.962662	5.400175	3.265178	0.749927
44	-5.828272	-2.786947	0.574692	-5.592329	0.970749	0.259821	2.540475	1.304447	2.389519
45	-6.022563	-1.896896	2.096206	-6.52473	0.18927	1.549647	4.049218	0.633785	3.037292
46	-4.499361	-2.727681	1.753372	-6.572579	-0.46992	-0.100868	3.140228	-0.262715	1.809862
47	-3.748197	0.659482	1.238808	-5.732791	-2.071076	2.177894	5.11567	-0.180652	0.394741
48	-3.293513	-4.854539	-2.1687	-3.473158	-4.951013	-1.70882	3.492196	4.319133	-1.11442
49	-3.376086	-4.70531	-0.388745	-4.622994	-3.915976	-0.814777	2.096884	4.936659	-2.035084
50	-4.556646	-3.848155	-1.416748	-3.325087	-4.760616	0.0638	2.017372	4.834664	-0.251674
51	-0.925303	3.289252	-1.014186	-2.379658	2.378803	-2.135328	1.923287	-3.460199	-1.372909
52	-2.388171	2.642488	-1.752648	-0.897315	3.109442	-1.53155	3.082267	-2.346601	-2.08974
53	-1.918834	2.799123	1.304447	-1.887077	3.162014	0.818367	2.928401	-3.068094	0.916614
54	-4.788555	4.583933	1.922869	-5.189786	3.355295	1.585329	6.307892	-3.682003	1.187732
55	-5.224932	2.884334	2.067161	-3.619774	4.054546	2.032975	6.048784	-1.997492	1.634686
56	-3.650491	3.441654	2.671154	-4.784036	4.987811	1.06699	4.868622	-3.226816	2.128001
57	-5.711252	3.108737	-0.441993	-5.004918	4.370728	-1.530629	6.283901	-1.456904	-0.919335
58	-4.867956	4.608292	-0.800237	-5.667646	2.872184	-0.89483	6.325244	-3.147921	-1.388962
59	-4.360434	3.103976	-1.587383	-4.336153	2.815052	-2.057107	5.074101	-2.077998	-2.048673
60	6.279636	-2.226679	1.215312	6.385021	-2.381182	0.345762	-6.309821	-2.589287	0.421237
61	6.423177	3.491145	-1.198035	6.208747	3.820022	0.139435	-6.261282	3.608869	0.083059
	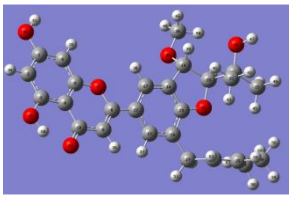			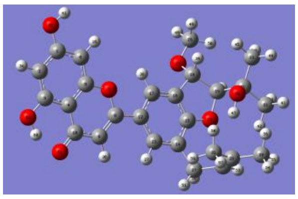			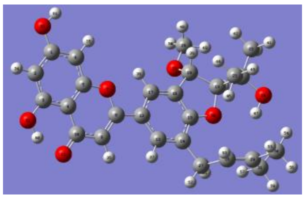		

	Conformer 25			Conformer 26			Conformer 27		
1	-6.176767	1.691195	0.179142	-5.760827	1.801533	1.118332	-6.020906	1.696563	0.894618
2	-6.922756	0.512454	0.323853	-6.63616	0.768563	0.752859	-6.846253	0.594089	0.629929
3	-6.273716	-0.716652	0.306097	-6.131605	-0.375836	0.146374	-6.281824	-0.592147	0.175905
4	-4.861309	-0.779687	0.140551	-4.735348	-0.500303	-0.101227	-4.874809	-0.689074	-0.017646
5	-4.160028	0.427618	0.001379	-3.901259	0.560886	0.282703	-4.092393	0.442381	0.259985
6	-4.786761	1.66705	0.016578	-4.382601	1.713592	0.88992	-4.634083	1.638198	0.713441
7	-2.802567	0.428492	-0.165884	-2.550705	0.494483	0.075567	-2.734312	0.405801	0.098801
8	-2.096787	-0.736694	-0.183246	-1.986884	-0.588848	-0.527743	-2.112992	-0.719132	-0.352505
9	-2.71759	-1.942097	-0.037276	-2.744666	-1.643965	-0.941903	-2.817649	-1.845291	-0.657747
10	-4.145751	-2.042224	0.123054	-4.17114	-1.674272	-0.74135	-4.248507	-1.907345	-0.496653
11	-4.739761	-3.145704	0.251112	-4.888216	-2.643984	-1.104645	-4.917805	-2.940525	-0.76225
12	-0.661036	-0.509068	-0.35761	-0.536326	-0.451486	-0.668965	-0.663674	-0.542065	-0.467066
13	0.197655	-1.575029	-0.701444	0.263672	-1.580682	-0.945317	0.186423	-1.664401	-0.566323
14	1.571525	-1.413412	-0.868058	1.6456	-1.5024	-1.105356	1.569426	-1.550936	-0.694283
15	2.062088	-0.115998	-0.676993	2.197387	-0.221045	-1.005311	2.071359	-0.245686	-0.720315
16	1.242213	0.955717	-0.328972	1.447355	0.899769	-0.664862	1.264915	0.882273	-0.591011
17	-0.121153	0.779071	-0.174974	0.076917	0.805998	-0.502949	-0.107185	0.752179	-0.469666
18	2.0934	2.189727	-0.210214	2.379791	2.069236	-0.51618	2.14497	2.103908	-0.642785
19	3.525939	1.597741	-0.343365	3.778486	1.393585	-0.662316	3.559799	1.470465	-0.522494
20	3.370246	0.222696	-0.802365	3.519634	0.041774	-1.164273	3.391848	0.05011	-0.834396
21	4.351176	1.58985	0.956814	4.595705	1.220728	0.636955	4.241655	1.55189	0.860257
22	3.677548	0.773297	2.065729	5.925457	0.520647	0.327157	4.74623	2.970392	1.108051
23	5.759524	1.058005	0.68054	4.85161	2.561492	1.32116	3.335731	1.068899	2.000902
24	4.406093	2.981875	1.309253	3.826887	0.42691	1.553422	5.404356	0.710637	0.803843
25	1.770807	3.088735	-1.266162	2.116553	3.022862	-1.538879	1.9403	2.771035	-1.882626
26	2.304442	4.390178	-1.057165	2.742036	4.276049	-1.301889	2.523209	4.066692	-1.915047
27	2.502046	-2.563934	-1.180536	2.529804	-2.71979	-1.273451	2.504683	-2.740965	-0.71415
28	3.378576	-2.887758	0.006108	3.660357	-2.779008	-0.257993	3.361543	-2.770905	0.530278
29	4.717778	-2.862564	0.071508	3.533236	-2.882353	1.074898	4.697165	-2.672463	0.619429
30	5.430874	-3.197578	1.358352	4.747311	-2.895194	1.972571	5.379417	-2.676232	1.966891
31	5.629844	-2.532365	-1.082382	2.207851	-2.977676	1.788808	5.633242	-2.555262	-0.558421
32	-6.982686	-1.850468	0.446021	-6.965762	-1.368795	-0.208533	-7.067906	-1.652076	-0.08125
33	-6.873837	2.855369	0.204924	-6.321192	2.889432	1.705038	-6.638256	2.821629	1.335585
34	-7.998052	0.562358	0.448614	-7.698756	0.865048	0.942226	-7.916911	0.669386	0.779178
35	-4.205397	2.575361	-0.096096	-3.701367	2.508963	1.171451	-3.991512	2.48754	0.91746
36	-2.141155	-2.857386	-0.023053	-2.285427	-2.481177	-1.450298	-2.312133	-2.718507	-1.048031
37	-0.21643	-2.565808	-0.857442	-0.197904	-2.561777	-0.99087	-0.237991	-2.6614	-0.508828
38	-0.762206	1.611394	0.089286	-0.51544	1.677967	-0.253436	-0.744217	1.624019	-0.382174
39	1.974068	2.703554	0.750898	2.2693	2.546963	0.464707	1.947597	2.803591	0.180072
40	4.081736	2.130824	-1.11834	4.371134	1.904342	-1.425009	4.239322	1.866765	-1.279276
41	2.678811	1.155061	2.296436	6.468314	0.342371	1.260296	5.293414	3.00375	2.054973
42	4.280634	0.827363	2.979327	5.752116	-0.439635	-0.166158	3.918286	3.683294	1.166847
43	3.596247	-0.278853	1.777321	6.551811	1.135511	-0.328136	5.42317	3.284152	0.306833

44	6.254088	1.664256	-0.085511	5.486509	2.405155	2.198326	2.438149	1.685435	2.110994
45	5.71773	0.021208	0.338433	3.922716	3.029576	1.656949	3.898399	1.111882	2.938127
46	6.363839	1.093087	1.594533	5.365581	3.250775	0.643704	3.025059	0.032095	1.839158
47	4.943427	3.055347	2.114508	3.747214	-0.457182	1.150591	5.06913	-0.162818	0.536227
48	2.012333	4.992643	-1.920163	2.439145	4.939559	-2.114731	2.119973	4.703743	-1.114444
49	1.89718	4.84247	-0.141249	2.421233	4.706212	-0.342056	3.617153	4.030548	-1.814739
50	3.399406	4.378049	-0.976511	3.838538	4.197109	-1.299184	2.272179	4.501683	-2.884681
51	3.106252	-2.322148	-2.059782	1.902109	-3.616801	-1.211006	3.122122	-2.709515	-1.616737
52	1.895237	-3.439876	-1.443964	2.968041	-2.71972	-2.278153	1.903518	-3.6569	-0.772261
53	2.827261	-3.150422	0.909544	4.665444	-2.712432	-0.668999	2.794814	-2.855358	1.457978
54	6.131226	-4.032135	1.216826	4.775857	-3.808891	2.580496	5.903018	-1.725499	2.136901
55	4.730966	-3.467805	2.155008	5.677394	-2.830913	1.399778	4.66558	-2.82005	2.783517
56	6.029937	-2.343617	1.703555	4.72213	-2.050194	2.673802	6.137541	-3.468376	2.024885
57	5.1019	-2.13313	-1.95015	2.225718	-3.795983	2.519395	5.116107	-2.466517	-1.515119
58	6.184678	-3.425835	-1.400307	2.004859	-2.056814	2.352892	6.290059	-1.681527	-0.45155
59	6.382031	-1.793532	-0.778937	1.369428	-3.140772	1.109788	6.295129	-3.430161	-0.606114
60	-6.313123	-2.59542	0.404856	-6.38663	-2.074328	-0.622821	-6.451169	-2.377904	-0.393282
61	-6.264823	3.603986	0.092348	-5.631205	3.540284	1.915318	-5.978538	3.517606	1.490978
	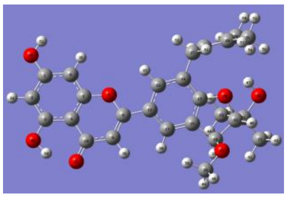			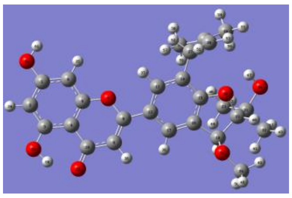			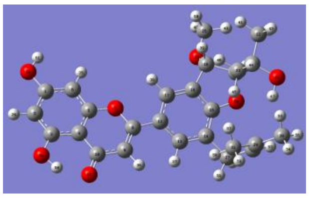		
	Conformer 28			Conformer 29			Conformer 30		
1	6.016307	2.059739	0.233817	6.037641	1.708803	0.037211	-5.984369	1.81322	0.41187
2	6.819363	0.91676	0.357645	6.766015	0.543547	0.317407	-6.742017	0.635132	0.482183
3	6.231327	-0.342143	0.319686	6.103979	-0.675688	0.402181	-6.113145	-0.594996	0.328659
4	4.8228	-0.47152	0.15831	4.695343	-0.742129	0.207834	-4.709574	-0.659696	0.099718
5	4.063338	0.702499	0.037716	4.012238	0.4515	-0.07106	-3.996101	0.547114	0.039627
6	4.628833	1.970455	0.071748	4.652257	1.680765	-0.161362	-4.602536	1.787248	0.191087
7	2.704789	0.64027	-0.112862	2.657394	0.449812	-0.260757	-2.646607	0.547193	-0.183837
8	2.061848	-0.558624	-0.170689	1.943491	-0.708323	-0.201951	-1.961134	-0.620041	-0.334562
9	2.743584	-1.73546	-0.07904	2.549843	-1.903482	0.047209	-2.592533	-1.826345	-0.270141
10	4.172538	-1.767876	0.105785	3.968805	-1.996261	0.281159	-4.015093	-1.92389	-0.059951
11	4.821006	-2.843008	0.203356	4.54768	-3.088262	0.523031	-4.61964	-3.027349	-0.006214
12	0.615879	-0.410047	-0.349231	0.514112	-0.495403	-0.441277	-0.529626	-0.393873	-0.548421
13	0.078923	0.816815	-0.789639	0.0654	0.715857	-1.008826	0.291708	-1.42848	-1.044227
14	-1.289469	1.009989	-0.984002	-1.281404	0.976551	-1.264016	1.662267	-1.272558	-1.246773
15	-2.101622	-0.096515	-0.728459	-2.169135	-0.047411	-0.925941	2.17877	-0.010795	-0.937615
16	-1.607773	-1.322122	-0.277238	-1.762733	-1.244268	-0.337432	1.40028	1.027245	-0.430788
17	-0.249543	-1.488615	-0.073154	-0.424104	-1.487888	-0.095183	0.040452	0.857583	-0.242693
18	-2.784914	-2.258944	-0.106257	-2.98542	-2.087292	-0.083105	2.292189	2.219271	-0.198094
19	-3.972687	-1.278266	-0.18316	-4.124267	-1.059267	-0.33069	3.701656	1.584969	-0.361655
20	-3.455957	-0.073433	-0.834317	-3.51565	0.034335	-1.089428	3.490177	0.31987	-1.066604

21	-4.603121	-0.840022	1.156404	-4.776974	-0.435078	0.922712	4.467966	1.256951	0.938795
22	-5.450694	-1.973973	1.725487	-5.719415	-1.445237	1.571583	5.027008	2.540855	1.545986
23	-3.566146	-0.347212	2.174458	-3.746861	0.094073	1.93016	3.614992	0.483354	1.953722
24	-5.507506	0.234503	0.85702	-5.596507	0.654628	0.476542	5.600534	0.455567	0.573903
25	-2.932403	-3.178787	-1.185826	-3.001954	-3.166444	-1.010195	2.011267	3.206994	-1.182968
26	-1.93908	-4.192496	-1.206207	-3.962167	-4.161521	-0.682311	2.604923	4.464025	-0.888129
27	-1.895325	2.345369	-1.359991	-1.769809	2.291534	-1.837014	2.553163	-2.39163	-1.746922
28	-2.697042	2.90948	-0.209735	-2.926629	2.905693	-1.067655	3.816751	-2.584914	-0.926396
29	-4.013931	3.165331	-0.163325	-2.881979	3.387957	0.182905	3.881711	-3.00126	0.346742
30	-4.649582	3.691732	1.101693	-4.119816	3.932486	0.850992	5.204638	-3.106077	1.063683
31	-4.971224	2.969935	-1.313396	-1.632241	3.420088	1.024647	2.675883	-3.381091	1.167169
32	6.996047	-1.441805	0.43571	6.796845	-1.796662	0.668654	-6.83345	-1.728212	0.396558
33	6.654971	3.256074	0.279853	6.747072	2.86364	-0.030391	-6.661118	2.978968	0.568696
34	7.891202	1.017189	0.481473	7.838132	0.596169	0.465965	-7.810599	0.686228	0.65481
35	4.004068	2.851556	-0.023844	4.084119	2.57874	-0.377262	-4.012685	2.695392	0.136093
36	2.219635	-2.678298	-0.163271	1.972329	-2.818251	0.059059	-2.029094	-2.745485	-0.359788
37	0.751547	1.647075	-0.973589	0.794722	1.481745	-1.247591	-0.149458	-2.390301	-1.284573
38	0.136825	-2.418794	0.327344	-0.113992	-2.412377	0.378693	-0.573008	1.660585	0.147492
39	-2.75516	-2.812237	0.84199	-3.020657	-2.487765	0.938367	2.162827	2.657924	0.79998
40	-4.75088	-1.674501	-0.836851	-4.904612	-1.473275	-0.971843	4.339179	2.188524	-1.010329
41	-4.834143	-2.841731	1.978619	-5.173069	-2.31552	1.947705	4.224823	3.217739	1.855336
42	-5.953526	-1.633756	2.635784	-6.234653	-0.975421	2.414841	5.666255	3.060675	0.825
43	-6.212901	-2.284357	1.003722	-6.472199	-1.786652	0.853684	5.62771	2.297587	2.427603
44	-2.854421	-1.129749	2.455151	-3.117691	-0.700974	2.342378	4.238019	0.234695	2.818005
45	-4.088978	-0.018069	3.077361	-4.279575	0.57281	2.7572	3.24665	-0.450226	1.519739
46	-3.004278	0.504358	1.778449	-3.097702	0.841121	1.465667	2.755481	1.062132	2.305889
47	-4.964421	0.900482	0.40014	-5.003922	1.231497	-0.037985	5.235256	-0.301051	0.081252
48	-2.226305	-4.898056	-1.988862	-3.865794	-4.951671	-1.429843	2.299448	5.151205	-1.679918
49	-1.888366	-4.720467	-0.242423	-4.988923	-3.770224	-0.709632	3.702519	4.40881	-0.869768
50	-0.945564	-3.78726	-1.439048	-3.774536	-4.580598	0.316996	2.258665	4.848958	0.082134
51	-2.515068	2.232686	-2.254332	-2.089975	2.132851	-2.873856	1.962205	-3.314514	-1.778161
52	-1.083838	3.036601	-1.619474	-0.920218	2.982834	-1.88336	2.843291	-2.180947	-2.783329
53	-2.111077	3.095595	0.69081	-3.879824	2.932138	-1.592756	4.748533	-2.339231	-1.432804
54	-5.389575	2.977674	1.488218	-3.960984	4.962045	1.198105	5.358638	-4.117205	1.463158
55	-3.907745	3.868285	1.886338	-4.98297	3.92646	0.177782	5.238364	-2.420488	1.921167
56	-5.186389	4.630999	0.913913	-4.377249	3.337571	1.737634	6.045348	-2.865213	0.405301
57	-4.507642	2.513182	-2.189154	-0.748754	3.064309	0.491366	2.570197	-2.713214	2.032873
58	-5.816746	2.335816	-1.014749	-1.435343	4.440916	1.377738	1.743807	-3.341254	0.600473
59	-5.403346	3.932918	-1.616208	-1.754106	2.798777	1.922296	2.792319	-4.395787	1.570061
60	6.365405	-2.218616	0.377459	6.121646	-2.537298	0.67759	-6.17841	-2.47462	0.261444
61	6.008593	3.975344	0.186197	6.149736	3.604027	-0.227651	-6.044971	3.726918	0.497341

	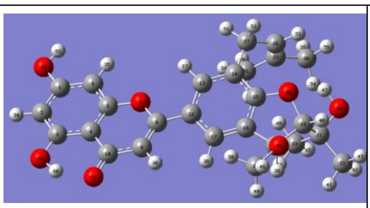<br>Conformer 31				
1	5.910664	1.843278	0.039614		
2	6.665125	0.699929	0.34008		
3	6.032313	-0.533868	0.436514		
4	4.62696	-0.637114	0.234234		
5	3.91709	0.53588	-0.065318		
6	4.527849	1.778912	-0.168227		
7	2.563729	0.499057	-0.262707		
8	1.878413	-0.675514	-0.192673		
9	2.51169	-1.852619	0.074445		
10	3.930869	-1.90771	0.319517		
11	4.534507	-2.981993	0.579163		
12	0.445808	-0.502269	-0.442081		
13	-0.028543	0.677183	-1.050462		
14	-1.382694	0.901187	-1.305653		
15	-2.245821	-0.131985	-0.937597		
16	-1.814437	-1.30903	-0.321502		
17	-0.470259	-1.503641	-0.059787		
18	-3.035643	-2.166595	-0.06273		
19	-4.173165	-1.147321	-0.270021		
20	-3.596824	-0.06945	-1.075492		
21	-4.770223	-0.504279	1.001864		
22	-5.691995	-1.501849	1.698317		
23	-3.700808	0.03033	1.964392		
24	-5.600265	0.583965	0.572908		
25	-3.212783	-3.195522	-1.03397		
26	-2.264114	-4.24557	-0.924671		
27	-1.903002	2.194957	-1.898764		
28	-3.052815	2.809402	-1.118702		
29	-2.989786	3.321193	0.119405		
30	-4.221751	3.862899	0.800727		
31	-1.723808	3.392343	0.933994		
32	6.750531	-1.633969	0.721993		
33	6.591957	3.01422	-0.038132		
34	7.734573	0.78062	0.49514		
35	3.940028	2.659973	-0.400712		
36	1.955892	-2.780736	0.090919		
37	0.683322	1.451347	-1.314296		
38	-0.134612	-2.389589	0.466878		

39	-3.042944	-2.609028	0.942204		
40	-4.97485	-1.584645	-0.866492		
41	-5.13512	-2.37323	2.056074		
42	-6.166842	-1.021826	2.559417		
43	-6.476523	-1.842957	1.015392		
44	-3.056784	-0.763561	2.355328		
45	-4.200453	0.514345	2.808891		
46	-3.069181	0.773988	1.471294		
47	-5.025043	1.1516	0.028994		
48	-2.250544	-4.666492	0.091672		
49	-2.569857	-5.020235	-1.631273		
50	-1.250631	-3.909495	-1.180614		
51	-1.065502	2.89783	-1.978688		
52	-2.242676	2.007807	-2.924499		
53	-4.016827	2.810123	-1.624365		
54	-4.458319	3.277651	1.69951		
55	-4.067375	4.898743	1.130508		
56	-5.096082	3.838828	0.142493		
57	-1.819548	2.795904	1.851415		
58	-0.846653	3.03239	0.393266		
59	-1.533133	4.425319	1.25363		
60	6.093826	-2.390898	0.736081		
61	5.977528	3.736991	-0.247765		

Table S14. Conformational analysis of (2''*S*,2'''*R*)-**6**

Conformers	Gibbs Free Energy (Hartree)	Relative Gibbs Free Energy (kcal/mol)	Population (%)
1	-1494.63764	1.35416658	2.09
2	-1494.637099	1.69364949	1.18
3	-1494.63772	1.30396578	2.27
4	-1494.637381	1.51669167	1.59
5	-1494.637517	1.43135031	1.83
6	-1494.637915	1.18160133	2.79
7	-1494.637151	1.66101897	1.24
8	-1494.638795	0.62939253	7.09
9	-1494.638095	1.06864953	3.38
10	-1494.637105	1.68988443	1.18
11	-1494.637672	1.33408626	2.16
12	-1494.639798	0	20.50
13	-1494.638477	0.82894071	5.06
14	-1494.639059	0.46372989	9.37
15	-1494.63721	1.62399588	1.32
16	-1494.639568	0.1443273	16.07

17	-1494.638056	1.09312242	3.24
18	-1494.637489	1.44892059	1.78
19	-1494.63759	1.38554208	1.98
20	-1494.637315	1.55810733	1.48
21	-1494.638073	1.08245475	3.30
22	-1494.637825	1.23807723	2.54
23	-1494.637125	1.67733423	1.21
24	-1494.637496	1.44452802	1.79
25	-1494.638151	1.03350897	3.58

Table S15. Coordinates of (2''*S*,2'''*R*)-**6**

	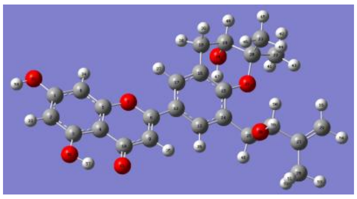 Conformer 1			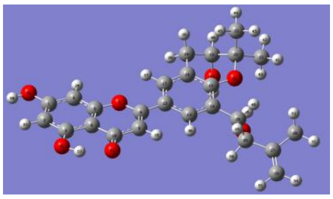 Conformer 2			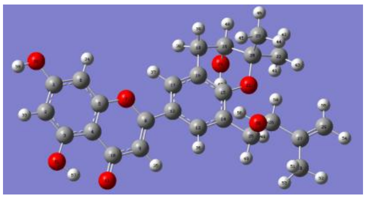 Conformer 3		
1	6.195942	-1.500813	0.011118	6.150494	-1.484031	-0.643111	6.064244	-1.410934	0.889283
2	6.805051	-0.238351	0.066467	6.816144	-0.286837	-0.341352	6.733157	-0.265667	0.431558
3	6.011873	0.90343	0.070372	6.080604	0.823534	0.055949	6.000367	0.776094	-0.126055
4	4.59591	0.796891	0.019155	4.665269	0.751498	0.154232	4.586135	0.685491	-0.232984
5	4.037156	-0.491735	-0.032733	4.047447	-0.47116	-0.160396	3.967005	-0.485451	0.237478
6	4.805151	-1.646069	-0.038724	4.757795	-1.593427	-0.558879	4.67391	-1.53858	0.796688
7	2.679519	-0.656262	-0.088675	2.686524	-0.598187	-0.086245	2.607161	-0.626546	0.16755
8	1.840965	0.416886	-0.080405	1.903606	0.442906	0.310344	1.826852	0.352798	-0.367405
9	2.321792	1.691824	-0.014842	2.445682	1.650021	0.641809	2.368369	1.507175	-0.85146
10	3.733077	1.960484	0.028038	3.862452	1.881394	0.573955	3.785025	1.748086	-0.805599
11	4.199327	3.134596	0.07947	4.382437	2.995202	0.869759	4.306566	2.812741	-1.244719
12	0.433088	0.022815	-0.139018	0.48228	0.093978	0.337201	0.407332	-0.000932	-0.358804
13	-0.561366	0.954358	-0.491997	-0.500624	1.091526	0.484328	-0.581098	0.986494	-0.532071
14	-1.900629	0.59537	-0.548411	-1.850209	0.775304	0.532946	-1.932211	0.664039	-0.543264
15	-2.254606	-0.7326	-0.232354	-2.224493	-0.580885	0.441764	-2.302493	-0.686321	-0.362093
16	-1.286261	-1.687988	0.113954	-1.275855	-1.594976	0.249768	-1.340578	-1.69404	-0.192853
17	0.049939	-1.294862	0.14989	0.071418	-1.2396	0.202353	0.005735	-1.3331	-0.190039
18	-1.698937	-3.104795	0.441096	-1.725538	-3.02379	0.052184	-1.769298	-3.133681	-0.025283
19	-3.115738	-3.405017	-0.053631	-3.201783	-3.077203	-0.346515	-3.221329	-3.339509	-0.461772
20	-4.070728	-2.255196	0.320576	-4.045549	-2.210044	0.606326	-4.119878	-2.231492	0.120165
21	-3.5824	-1.036741	-0.330891	-3.57082	-0.827062	0.485775	-3.640837	-0.947863	-0.402028
22	-5.465989	-2.457065	-0.251786	-5.506044	-2.145892	0.184267	-5.552874	-2.332101	-0.380685
23	-4.124164	-2.012861	1.828421	-3.90888	-2.637222	2.066361	-4.071033	-2.185257	1.646821
24	-3.131946	-3.654139	-1.462856	-3.386892	-2.677268	-1.708228	-3.330462	-3.399	-1.887427
25	-2.971907	1.595337	-0.901507	-2.911757	1.842192	0.611452	-2.993781	1.727031	-0.663829
26	-3.70317	2.120071	0.346049	-3.493195	2.209673	-0.77714	-3.588287	2.095735	0.716674

27	-4.914826	2.962336	0.000079	-4.668406	3.16103	-0.632642	-4.706735	3.106899	0.589964
28	-4.669242	4.238753	-0.759436	-5.981607	2.553612	-0.216533	-4.322161	4.525768	0.264547
29	-6.134643	2.5588	0.369623	-4.510491	4.469592	-0.852933	-5.976863	2.715756	0.731736
30	-2.734161	2.87994	1.089888	-3.854659	1.0501	-1.543968	-2.576908	2.654387	1.568051
31	6.594348	2.118978	0.124024	6.719137	1.974713	0.350297	6.639503	1.879374	-0.566318
32	6.92821	-2.644226	0.002683	6.827512	-2.595494	-1.031387	6.733484	-2.45271	1.446317
33	7.885898	-0.14483	0.107722	7.897433	-0.220337	-0.412244	7.812826	-0.184413	0.51214
34	4.344959	-2.625987	-0.081923	4.252601	-2.522472	-0.794792	4.167463	-2.427243	1.154023
35	1.639742	2.53169	0.020876	1.81291	2.460999	0.978291	1.734969	2.261504	-1.300285
36	-0.291049	1.973497	-0.747305	-0.215697	2.136728	0.54345	-0.299371	2.028525	-0.642547
37	0.80201	-2.030376	0.414919	0.809159	-2.020728	0.054843	0.752555	-2.110976	-0.070859
38	-1.006079	-3.818202	-0.017159	-1.128757	-3.499346	-0.732892	-1.126022	-3.792292	-0.618099
39	-1.647598	-3.268944	1.524717	-1.568266	-3.608582	0.966517	-1.653128	-3.440626	1.021529
40	-3.482893	-4.326303	0.405956	-3.566788	-4.10602	-0.294564	-3.584323	-4.306407	-0.104007
41	-5.427183	-2.619197	-1.331592	-6.044614	-1.425627	0.808504	-5.585164	-2.354339	-1.472516
42	-5.936652	-3.328023	0.215985	-5.970863	-3.129205	0.310353	-6.015914	-3.247619	0.001788
43	-6.08393	-1.576673	-0.047057	-5.59838	-1.848845	-0.863164	-6.133979	-1.474871	-0.025365
44	-4.870433	-1.24415	2.053627	-4.181987	-3.691949	2.178285	-4.782979	-1.441164	2.01815
45	-4.409332	-2.933338	2.349142	-4.579612	-2.037743	2.690129	-3.076862	-1.920655	2.017448
46	-3.160764	-1.678838	2.223156	-2.889372	-2.503934	2.438016	-4.34452	-3.161109	2.062348
47	-2.921091	-2.819617	-1.916683	-3.154012	-1.735062	-1.784677	-3.120968	-2.516786	-2.24061
48	-2.5235	2.442881	-1.42916	-3.723053	1.510578	1.267471	-3.809296	1.381341	-1.306426
49	-3.713609	1.141343	-1.565823	-2.49213	2.755918	1.042839	-2.562747	2.625426	-1.115827
50	-4.032903	1.262621	0.945419	-2.701928	2.701745	-1.353077	-3.988382	1.180727	1.171166
51	-3.913949	4.851922	-0.253751	-6.749249	3.321143	-0.079015	-3.689667	4.951072	1.052909
52	-4.285344	4.034344	-1.767649	-6.335667	1.843055	-0.973652	-5.208191	5.158557	0.154562
53	-5.58678	4.826637	-0.859286	-5.887527	1.990326	0.721307	-3.743188	4.58185	-0.66625
54	-7.026711	3.133389	0.128361	-5.326568	5.176692	-0.719094	-6.805697	3.407717	0.59821
55	-6.284533	1.63217	0.92083	-3.554665	4.881763	-1.171436	-6.232826	1.686852	0.977195
56	-3.159223	3.150812	1.921165	-4.190174	0.374352	-0.92663	-1.863083	1.996751	1.63465
57	5.844503	2.784601	0.118274	6.004477	2.626853	0.611551	5.92654	2.487429	-0.9226
58	7.876841	-2.428642	0.038135	7.784251	-2.413505	-1.048762	7.686825	-2.258112	1.473558
	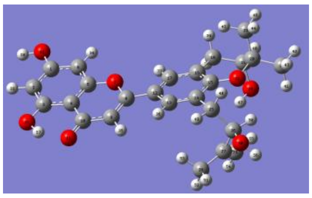 Conformer 4			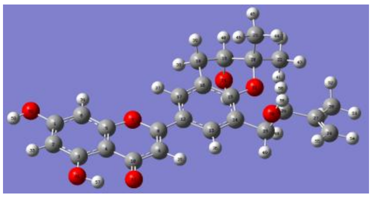 Conformer 5			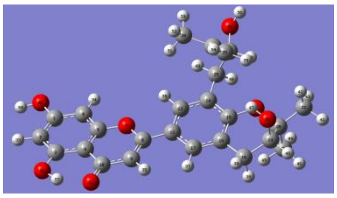 Conformer 6		
1	-5.928415	-1.820551	0.219362	6.186596	-1.522624	0.267058	-5.752389	1.524285	-0.790683
2	-6.626146	-0.604568	0.166532	6.822105	-0.27816	0.145343	-6.518898	0.482404	-0.247092
3	-5.919764	0.58309	0.015245	6.053828	0.868175	-0.022339	-5.881475	-0.657112	0.230631
4	-4.502675	0.569727	-0.086809	4.636557	0.783129	-0.073909	-4.46637	-0.769651	0.170265
5	-3.852802	-0.675126	-0.026739	4.050742	-0.488055	0.051229	-3.746889	0.304693	-0.380089
6	-4.533362	-1.873608	0.124146	4.793692	-1.646595	0.220736	-4.357129	1.452017	-0.863069

7	-2.489919	-0.750635	-0.127657	2.690741	-0.630831	0.005124	-2.382356	0.251764	-0.4676
8	-1.732049	0.372477	-0.26693	1.872454	0.446654	-0.15322	-1.688135	-0.829749	-0.0162
9	-2.302744	1.611047	-0.311463	2.381625	1.707753	-0.266431	-2.329408	-1.899593	0.537138
10	-3.727443	1.784328	-0.236756	3.797337	1.952427	-0.239041	-3.761007	-1.938039	0.657104
11	-4.276272	2.922322	-0.288181	4.287375	3.112924	-0.348762	-4.373428	-2.922395	1.162184
12	-0.30328	0.070637	-0.346671	0.458431	0.070201	-0.182373	-0.244508	-0.671823	-0.194674
13	0.614128	1.037282	-0.800076	-0.536384	1.019263	-0.487984	0.297604	0.584811	-0.524381
14	1.974732	0.77387	-0.87024	-1.8783	0.671923	-0.529707	1.663715	0.770483	-0.689157
15	2.428484	-0.508179	-0.493718	-2.238796	-0.662273	-0.246742	2.511962	-0.345987	-0.543698
16	1.541547	-1.488613	-0.018911	-1.272656	-1.633453	0.056803	2.004749	-1.610023	-0.193988
17	0.183761	-1.183299	0.046193	0.067016	-1.25084	0.081047	0.630552	-1.753428	-0.023638
18	2.067777	-2.827366	0.445528	-1.6907	-3.057364	0.343268	2.947061	-2.772774	0.016701
19	3.567236	-2.752041	0.734276	-3.115081	-3.330842	-0.145087	4.37225	-2.277033	0.261558
20	4.301209	-2.068246	-0.435941	-4.05909	-2.189703	0.280655	4.766733	-1.238443	-0.80667
21	3.773274	-0.709214	-0.576005	-3.570916	-0.950388	-0.33106	3.842654	-0.106923	-0.709652
22	5.78283	-1.874362	-0.148191	-5.461275	-2.36428	-0.283799	6.137927	-0.635719	-0.537703
23	4.090168	-2.802889	-1.759671	-4.09476	-2.001152	1.7967	4.682828	-1.804272	-2.224111
24	3.824563	-2.087437	1.975928	-3.148942	-3.529788	-1.561936	4.517827	-1.74965	1.584402
25	2.947179	1.838606	-1.311774	-2.953739	1.683958	-0.826481	2.228232	2.139052	-0.976854
26	3.933107	2.284779	-0.211393	-3.659848	2.174679	0.453187	3.121995	2.701038	0.148718
27	3.26357	2.535076	1.125188	-4.952036	2.900971	0.12941	2.517865	2.539895	1.529244
28	2.269753	3.662321	1.195764	-6.121439	2.010348	-0.19797	1.244097	3.287674	1.814733
29	3.558453	1.757649	2.172844	-5.031814	4.234515	0.14359	3.111973	1.745162	2.426012
30	4.555614	3.47632	-0.724718	-2.705319	2.986906	1.140523	3.321873	4.086333	-0.185951
31	-6.588255	1.753671	-0.034892	6.661706	2.067039	-0.135459	-6.615885	-1.66087	0.753651
32	-6.574324	-3.006055	0.364659	6.897006	-2.667249	0.436581	-6.328634	2.654734	-1.27481
33	-7.709051	-0.583895	0.242299	7.904512	-0.20294	0.184592	-7.600833	0.557804	-0.198403
34	-4.003758	-2.81793	0.164434	4.312862	-2.61294	0.314969	-3.77456	2.262311	-1.284849
35	-1.684666	2.496115	-0.390875	1.722118	2.558343	-0.378625	-1.763967	-2.743581	0.910625
36	0.263769	2.01217	-1.121415	-0.267228	2.046665	-0.707148	-0.360019	1.438179	-0.64092
37	-0.500204	-1.934048	0.427396	0.815938	-2.001503	0.308085	0.24885	-2.734748	0.239989
38	1.545413	-3.142553	1.354721	-1.00846	-3.759728	-0.146842	2.933798	-3.440332	-0.853252
39	1.87673	-3.598062	-0.310865	-1.627534	-3.257949	1.420086	2.624337	-3.368883	0.876453
40	3.977423	-3.757741	0.856574	-3.483221	-4.265509	0.285714	5.075821	-3.111613	0.205713
41	5.93368	-1.342817	0.794185	-5.435092	-2.476396	-1.370276	6.909642	-1.404172	-0.651776
42	6.278112	-2.848766	-0.082404	-5.926751	-3.256061	0.148604	6.195917	-0.231378	0.47542
43	6.249824	-1.30316	-0.957021	-6.077087	-1.494968	-0.031603	6.341046	0.167358	-1.253515
44	4.682898	-2.323722	-2.545474	-4.832881	-1.235795	2.057144	5.041524	-1.058229	-2.940499
45	3.041865	-2.791283	-2.070166	-4.381002	-2.937589	2.287517	3.65817	-2.07134	-2.496611
46	4.412224	-3.845994	-1.67041	-3.124714	-1.687938	2.192285	5.308533	-2.698828	-2.313433
47	3.429319	-1.19919	1.935321	-2.932021	-2.682712	-1.988854	3.865445	-1.037558	1.703562
48	3.541771	1.491306	-2.165304	-3.696054	1.251512	-1.50239	2.827269	2.125943	-1.895377
49	2.385019	2.718629	-1.63997	-2.524539	2.561335	-1.321771	1.401142	2.838402	-1.134833
50	4.691498	1.507234	-0.079135	-3.908927	1.299795	1.07134	4.084287	2.180717	0.129387

51	2.715696	4.597454	0.837064	-5.896526	1.338994	-1.036622	0.425326	2.931801	1.176083
52	1.911415	3.815441	2.218312	-6.369825	1.367303	0.657614	0.936613	3.168876	2.858186
53	1.399601	3.46237	0.557078	-7.009455	2.594725	-0.45794	1.365385	4.356941	1.6049
54	3.083303	1.895451	3.141985	-5.959392	4.750846	-0.09259	2.696224	1.5891	3.419377
55	4.285	0.9511	2.09449	-4.172924	4.850341	0.395313	4.036867	1.220263	2.195179
56	5.250123	3.728755	-0.093133	-3.116812	3.273634	1.973411	3.964062	4.443267	0.450577
57	-5.890938	2.465199	-0.144499	5.926483	2.739712	-0.241859	-5.960789	-2.367584	1.027816
58	-7.535033	-2.856064	0.414962	7.848543	-2.462012	0.456106	-7.296503	2.59613	-1.18818
	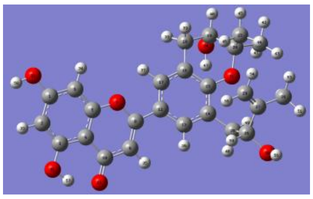			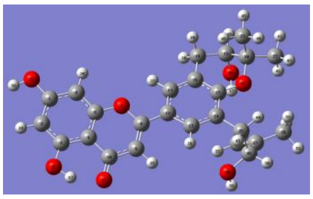			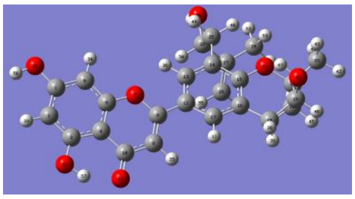		
	Conformer 7			Conformer 8			Conformer 9		
1	5.914668	-1.6114	0.957498	-5.928804	-1.861702	0.288304	5.832077	1.62016	-0.777562
2	6.66222	-0.512224	0.509417	-6.652565	-0.66103	0.236876	6.613239	0.607341	-0.20158
3	6.008687	0.566078	-0.075744	-5.973769	0.540189	0.068423	5.992743	-0.524328	0.314948
4	4.595231	0.558521	-0.221094	-4.558289	0.556334	-0.051866	4.579213	-0.658516	0.260186
5	3.894518	-0.568114	0.243235	-3.881103	-0.673862	0.008305	3.844113	0.3865	-0.325463
6	4.521925	-1.656211	0.830449	-4.534162	-1.885577	0.175912	4.437889	1.525643	-0.84726
7	2.530918	-0.627782	0.13795	-2.518167	-0.720886	-0.109269	2.480045	0.310429	-0.410571
8	1.825579	0.389488	-0.429768	-1.786771	0.417475	-0.267309	1.803269	-0.764047	0.081954
9	2.449769	1.501155	-0.915272	-2.385165	1.642873	-0.315426	2.459329	-1.802875	0.67513
10	3.875815	1.658302	-0.830947	-3.811717	1.786089	-0.222526	3.891743	-1.81845	0.790629
11	4.472759	2.684014	-1.267268	-4.385961	2.911626	-0.275471	4.518742	-2.775203	1.329584
12	0.387097	0.125456	-0.448627	-0.352483	0.147743	-0.360108	0.356963	-0.638794	-0.098074
13	-0.532913	1.164516	-0.672522	0.535885	1.129952	-0.832968	-0.216828	0.599597	-0.434665
14	-1.902458	0.934275	-0.699786	1.902507	0.897378	-0.915738	-1.589001	0.74972	-0.598738
15	-2.365797	-0.381717	-0.49051	2.389887	-0.371774	-0.540634	-2.413238	-0.379781	-0.420661
16	-1.46918	-1.445472	-0.284533	1.531831	-1.363364	-0.033617	-1.865246	-1.639183	-0.110651
17	-0.103976	-1.174078	-0.258797	0.169185	-1.08939	0.044967	-0.487705	-1.748934	0.047402
18	-1.985753	-2.856184	-0.119018	2.09485	-2.677849	0.455816	-2.762098	-2.848841	0.018736
19	-3.426421	-2.982072	-0.615456	3.605891	-2.582657	0.670454	-4.119555	-2.602067	-0.638911
20	-4.282667	-1.828296	-0.061057	4.272572	-1.917295	-0.547411	-4.685624	-1.240042	-0.194862
21	-3.715832	-0.567234	-0.551007	3.736386	-0.55816	-0.653675	-3.74904	-0.195871	-0.622226
22	-5.698273	-1.85083	-0.618504	5.76924	-1.729555	-0.345651	-5.98471	-0.902424	-0.911555
23	-4.291201	-1.807044	1.467242	3.984693	-2.657851	-1.852712	-4.854958	-1.15933	1.321977
24	-3.483944	-3.036176	-2.044426	3.918016	-1.876248	1.876121	-4.032056	-2.692891	-2.064243
25	-2.85604	2.085696	-0.899361	2.846295	1.997287	-1.327607	-2.16488	2.106946	-0.916578
26	-3.090455	2.884056	0.398549	3.274839	2.894691	-0.140619	-2.305622	2.991631	0.338161
27	-3.812155	2.088006	1.468191	4.024259	2.137486	0.94049	-3.32816	2.467702	1.327683
28	-5.291548	1.880715	1.282485	5.490966	1.928415	0.680785	-4.778967	2.660499	0.975807
29	-3.132991	1.594199	2.508596	3.411389	1.700443	2.046603	-2.926004	1.853892	2.445298
30	-3.835985	4.04719	0.008028	2.085723	3.52407	0.340845	-2.655585	4.29707	-0.146401

31	6.724674	1.625299	-0.506172	-6.667913	1.695923	0.018849	6.741675	-1.498969	0.870754
32	6.509349	-2.685296	1.537633	-6.547695	-3.059898	0.448623	6.394718	2.74197	-1.296096
33	7.742387	-0.496682	0.617051	-7.734592	-0.662385	0.326455	7.693983	0.699984	-0.156278
34	3.953709	-2.509624	1.180781	-3.98398	-2.81811	0.215344	3.843718	2.313189	-1.295172
35	1.875579	2.286254	-1.390225	-1.787213	2.540169	-0.409936	1.904519	-2.636155	1.086606
36	-0.181365	2.182296	-0.808837	0.160838	2.09571	-1.152085	0.41936	1.468525	-0.562648
37	0.587459	-1.996629	-0.109571	-0.492337	-1.849663	0.446689	-0.073725	-2.729468	0.260866
38	-1.355936	-3.556261	-0.677753	1.620103	-2.960212	1.401365	-2.292166	-3.719118	-0.450924
39	-1.933396	-3.158229	0.934222	1.874621	-3.480308	-0.258909	-2.907652	-3.101561	1.076187
40	-3.856926	-3.927208	-0.274856	4.028775	-3.582156	0.800868	-4.825567	-3.38251	-0.343974
41	-5.688574	-1.846554	-1.711067	5.977258	-1.201293	0.587033	-6.322077	0.099568	-0.62838
42	-6.214773	-2.75417	-0.277417	6.259943	-2.707854	-0.309599	-6.760545	-1.621165	-0.627624
43	-6.256939	-0.978969	-0.263968	6.193593	-1.161675	-1.179658	-5.85406	-0.940429	-1.995583
44	-4.996875	-1.053196	1.826361	4.530481	-2.181726	-2.673659	-3.894212	-1.186299	1.842397
45	-4.604314	-2.783754	1.852119	4.313353	-3.699909	-1.776861	-5.46239	-1.999735	1.675431
46	-3.30539	-1.574823	1.878282	2.920438	-2.64994	-2.102502	-5.363238	-0.229652	1.59195
47	-3.210507	-2.168824	-2.390074	3.693094	-0.93713	1.745778	-3.462038	-1.968965	-2.376673
48	-2.432735	2.785254	-1.628606	3.744833	1.586253	-1.793992	-3.135063	2.017734	-1.411173
49	-3.81442	1.739038	-1.293129	2.358476	2.648378	-2.060725	-1.49187	2.634761	-1.601323
50	-2.108718	3.18854	0.786574	3.95305	3.658622	-0.551878	-1.324653	3.032255	0.83142
51	-5.82392	2.839491	1.279947	5.658652	1.447948	-0.29013	-5.43187	2.211709	1.730109
52	-5.5086	1.398758	0.322007	6.018942	2.891504	0.651715	-5.024319	3.726507	0.897876
53	-5.707023	1.259173	2.080993	5.951215	1.307446	1.455061	-5.015565	2.211676	0.004057
54	-3.616388	0.992633	3.274941	3.958202	1.16992	2.823381	-3.63494	1.439603	3.158506
55	-2.066346	1.772662	2.627979	2.353376	1.873973	2.219755	-1.870505	1.741013	2.684116
56	-3.985547	4.576471	0.80965	2.327266	4.024548	1.138577	-2.730579	4.881091	0.627113
57	6.059695	2.270185	-0.889193	-5.987137	2.421066	-0.105247	6.096751	-2.204535	1.17102
58	7.472276	-2.546884	1.577899	-7.511128	-2.931273	0.508515	7.362813	2.697572	-1.202397
	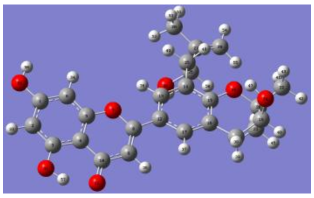 Conformer 10			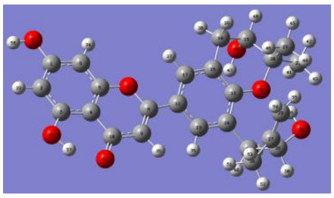 Conformer 11			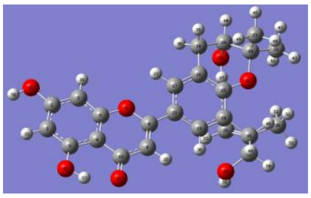 Conformer 12		
1	5.727249	1.805541	-0.65594	5.889378	-1.88082	0.307018	-5.817198	-1.798848	0.805617
2	6.574338	0.784545	-0.198842	6.60666	-0.679015	0.208996	-6.600671	-0.693645	0.440661
3	6.026246	-0.419082	0.223826	5.919826	0.514064	0.017216	-5.982924	0.443452	-0.06748
4	4.618467	-0.620516	0.19368	4.502337	0.520826	-0.080815	-4.570497	0.490385	-0.215602
5	3.81588	0.432353	-0.273842	3.83212	-0.710233	0.026658	-3.833441	-0.644478	0.163401
6	4.338489	1.645239	-0.701795	4.493112	-1.913684	0.218649	-4.424507	-1.791106	0.671625
7	2.455989	0.29574	-0.332604	2.467484	-0.766199	-0.066588	-2.470112	-0.65559	0.047348
8	1.845997	-0.851102	0.077433	1.729774	0.363842	-0.246687	-1.79603	0.420108	-0.446455
9	2.569818	-1.902764	0.558692	2.319469	1.589797	-0.339585	-2.456905	1.54654	-0.8425
10	4.003839	-1.855804	0.635852	3.747813	1.742008	-0.274008	-3.886183	1.653861	-0.742628

11	4.691939	-2.823942	1.069745	4.314481	2.867903	-0.36871	-4.515678	2.691764	-1.09759
12	0.390779	-0.785439	-0.059371	0.293912	0.086689	-0.309495	-0.351275	0.196764	-0.492397
13	-0.248569	0.444077	-0.305461	-0.599577	1.041804	-0.826204	0.537776	1.261624	-0.726172
14	-1.628842	0.530364	-0.431083	-1.969798	0.815004	-0.875314	1.911494	1.065994	-0.78161
15	-2.389489	-0.648392	-0.300446	-2.448769	-0.423631	-0.400135	2.409357	-0.245287	-0.62782
16	-1.781282	-1.89335	-0.068652	-1.583187	-1.410579	0.094637	1.550362	-1.321528	-0.344386
17	-0.394923	-1.942819	0.04606	-0.217121	-1.138683	0.135522	0.180348	-1.083502	-0.280618
18	-2.624832	-3.142542	0.046944	-2.12529	-2.73917	0.569492	2.118692	-2.694311	-0.069214
19	-4.042907	-2.923876	-0.486767	-3.538923	-2.993039	0.044321	3.610163	-2.607379	0.253597
20	-4.610727	-1.586628	0.026744	-4.414943	-1.743252	0.243757	4.327773	-1.739583	-0.796165
21	-3.739021	-0.512423	-0.457175	-3.799832	-0.63426	-0.505075	3.762502	-0.390178	-0.713802
22	-5.970869	-1.269274	-0.576703	-5.792792	-1.897405	-0.381797	5.804725	-1.564297	-0.473962
23	-4.658158	-1.527123	1.553206	-4.514462	-1.336324	1.71323	4.136089	-2.25994	-2.220129
24	-4.080831	-2.992071	-1.915661	-3.524606	-3.41764	-1.321223	3.826518	-2.098622	1.574319
25	-2.319897	1.851973	-0.65154	-2.891837	1.874217	-1.441563	2.849936	2.237722	-0.914288
26	-2.927191	2.409424	0.647256	-3.817251	2.590652	-0.416432	3.210635	2.878239	0.448802
27	-3.800368	3.624937	0.407158	-3.069374	2.978075	0.848362	3.940414	1.929735	1.383029
28	-3.131984	4.851369	-0.154847	-2.002455	4.027574	0.679861	5.420732	1.807902	1.147315
29	-5.105638	3.574473	0.691893	-3.343478	2.416777	2.028798	3.301316	1.266326	2.353697
30	-1.817748	2.724008	1.507413	-5.001601	1.83797	-0.160184	1.989274	3.374515	0.999145
31	6.836526	-1.403116	0.66734	6.607779	1.670567	-0.075917	-6.732545	1.507961	-0.420802
32	6.32724	2.957631	-1.052481	6.515654	-3.071273	0.491688	-6.373503	-2.931463	1.307663
33	7.647545	0.937762	-0.174642	7.68996	-0.673554	0.281196	-7.680277	-0.719052	0.552655
34	3.685282	2.434953	-1.057278	3.948026	-2.846946	0.294488	-3.82894	-2.650074	0.956955
35	2.069706	-2.797606	0.905848	1.715061	2.480796	-0.451309	-1.910608	2.385471	-1.253575
36	0.339901	1.350585	-0.388619	-0.220699	1.976885	-1.223525	0.161425	2.272323	-0.836109
37	0.071759	-2.910688	0.200472	0.453693	-1.897208	0.524427	-0.480904	-1.914861	-0.06179
38	-2.162817	-3.96603	-0.507569	-1.473064	-3.552998	0.236504	1.598136	-3.152132	0.778477
39	-2.675718	-3.460967	1.095582	-2.131932	-2.770368	1.665923	1.964757	-3.357349	-0.929196
40	-4.694807	-3.731892	-0.144637	-3.995829	-3.819438	0.594786	4.054911	-3.605834	0.248246
41	-6.296398	-0.27291	-0.260298	-5.715478	-2.165532	-1.4379	5.944199	-1.205456	0.547706
42	-6.709328	-2.001183	-0.233472	-6.348007	-2.683849	0.139752	6.318912	-2.525356	-0.579801
43	-5.931475	-1.29888	-1.668118	-6.352833	-0.960838	-0.292599	6.2603	-0.851547	-1.168576
44	-3.659486	-1.570873	1.99628	-5.213302	-0.500862	1.818507	4.723004	-1.651932	-2.916153
45	-5.245223	-2.364754	1.944877	-4.886744	-2.175506	2.31036	4.479209	-3.297556	-2.293138
46	-5.133461	-0.594145	1.872895	-3.547439	-1.026376	2.118001	3.089639	-2.220318	-2.533877
47	-3.610363	-2.216752	-2.26757	-3.179299	-2.688765	-1.865157	3.568953	-1.158299	1.584475
48	-3.124861	1.743841	-1.385088	-2.272137	2.632251	-1.928717	3.775495	1.941976	-1.413982
49	-1.603518	2.579265	-1.045983	-3.543314	1.448023	-2.213813	2.380593	3.019849	-1.520196
50	-3.53847	1.62619	1.112056	-4.15835	3.514886	-0.902595	3.883836	3.721956	0.231027
51	-3.819967	5.701744	-0.186229	-1.185353	3.677909	0.037061	5.637909	1.539208	0.107039
52	-2.259814	5.129574	0.448533	-2.412288	4.928654	0.203445	5.920848	2.767815	1.336164
53	-2.765579	4.673314	-1.174545	-1.571782	4.313946	1.644391	5.870209	1.052334	1.798274
54	-5.763748	4.423954	0.520541	-2.795595	2.694334	2.926569	3.835865	0.604808	3.032401

55	-5.564412	2.675823	1.100374	-4.129423	1.676816	2.138868	2.233958	1.3828	2.516841
56	-2.188758	2.993607	2.364454	-4.741746	0.897106	-0.158904	2.194098	3.73532	1.878364
57	6.234649	-2.163249	0.920012	5.922581	2.389396	-0.209022	-6.088074	2.200108	-0.753237
58	5.654051	3.599188	-1.341231	7.479543	-2.938281	0.530435	-7.340185	-2.829786	1.362858
	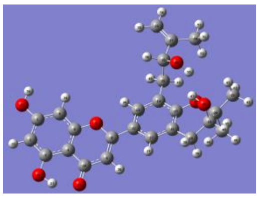 Conformer 13			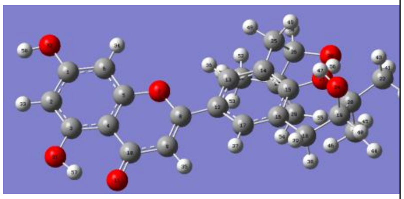 Conformer 14			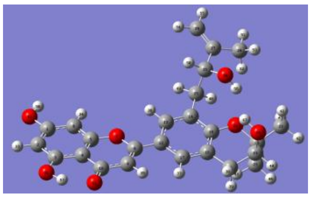 Conformer 15		
1	-5.739665	1.999006	0.144023	5.782037	1.401176	-0.948512	5.800744	1.866733	0.628031
2	-6.616776	0.904382	0.107595	6.531348	0.401597	-0.310626	6.646471	0.836218	0.190138
3	-6.100941	-0.381637	0.021509	5.876273	-0.673301	0.278728	6.09675	-0.373586	-0.212311
4	-4.695041	-0.592893	-0.028787	4.458865	-0.764158	0.234554	4.688433	-0.571837	-0.180926
5	-3.861422	0.536787	0.005236	3.756228	0.264886	-0.415966	3.887198	0.491164	0.265711
6	-4.351573	1.832806	0.09071	4.385101	1.348387	-1.010134	4.411428	1.710459	0.67254
7	-2.50049	0.397234	-0.037414	2.38995	0.228532	-0.49314	2.526821	0.358222	0.325102
8	-1.925675	-0.83403	-0.12696	1.681164	-0.788493	0.070154	1.916512	-0.797237	-0.058524
9	-2.680367	-1.968536	-0.175549	2.304372	-1.808577	0.726236	2.638102	-1.859666	-0.517224
10	-4.115917	-1.917871	-0.118601	3.736386	-1.864187	0.839635	4.072564	-1.814905	-0.598369
11	-4.833192	-2.957969	-0.156939	4.332923	-2.804384	1.438193	4.758827	-2.792063	-1.013495
12	-0.464716	-0.765851	-0.175757	0.237318	-0.625509	-0.10961	0.460885	-0.727684	0.08258
13	0.184394	0.436787	-0.512079	-0.293679	0.609629	-0.521266	-0.178642	0.512041	0.270578
14	1.568684	0.511257	-0.590847	-1.661518	0.807327	-0.677699	-1.557392	0.601066	0.409784
15	2.317451	-0.653085	-0.330775	-2.516058	-0.285172	-0.428211	-2.311474	-0.588062	0.378182
16	1.704477	-1.853884	0.05757	-2.01796	-1.540064	-0.041935	-1.708712	-1.837458	0.166698
17	0.31365	-1.892308	0.12504	-0.643102	-1.691495	0.11578	-0.324193	-1.887789	0.023587
18	2.550584	-3.042394	0.450882	-2.957849	-2.702716	0.179839	-2.558804	-3.082813	0.068075
19	3.980434	-2.605055	0.77473	-4.322437	-2.455234	-0.463878	-4.018165	-2.72747	-0.222941
20	4.530372	-1.704458	-0.34683	-4.829244	-1.04421	-0.115676	-4.502507	-1.622422	0.73447
21	3.677832	-0.512183	-0.413625	-3.854126	-0.073517	-0.641138	-3.666176	-0.437649	0.51146
22	5.914085	-1.16142	-0.021798	-6.123411	-0.701701	-0.83796	-5.913236	-1.156073	0.407106
23	4.508741	-2.386093	-1.71311	-4.960965	-0.828973	1.391108	-4.378349	-2.024889	2.202628
24	4.047996	-1.94605	2.043423	-4.282077	-2.665825	-1.87776	-4.197345	-2.345828	-1.590436
25	2.280066	1.805341	-0.890875	-2.18564	2.162287	-1.105012	-2.25536	1.930544	0.537953
26	2.766411	2.532923	0.388984	-2.962615	2.965749	-0.022735	-2.808173	2.449871	-0.81311
27	3.643612	3.719974	0.030068	-2.263741	2.924962	1.325862	-3.635813	3.707008	-0.607707
28	5.065966	3.41465	-0.358678	-0.926976	3.615773	1.388047	-5.037032	3.516427	-0.091036
29	3.146194	4.960119	0.050466	-2.806045	2.31463	2.382796	-3.1135	4.909862	-0.864897
30	3.437883	1.650268	1.301549	-4.332776	2.573538	0.042503	-3.553686	1.446657	-1.520439
31	-6.940732	-1.437296	-0.013663	6.594827	-1.633615	0.895892	6.905894	-1.366722	-0.636731
32	-6.310629	3.227665	0.234117	6.380064	2.468314	-1.537464	6.402448	3.023447	1.007808
33	-7.688465	1.064439	0.149139	7.614769	0.460907	-0.272875	7.720104	0.986471	0.166019
34	-3.674727	2.680072	0.118885	3.815417	2.125473	-1.505599	3.759317	2.507561	1.01325

35	-2.204373	-2.935232	-0.278904	1.724426	-2.594816	1.191979	2.136825	-2.762151	-0.842362
36	-0.401156	1.324459	-0.725413	0.375853	1.438477	-0.717826	0.408915	1.423013	0.297911
37	-0.160621	-2.81281	0.450942	-0.264552	-2.669643	0.395158	0.139056	-2.857621	-0.127828
38	2.121396	-3.532927	1.330472	-3.088	-2.87959	1.254551	-2.185904	-3.729924	-0.732387
39	2.565908	-3.789395	-0.351932	-2.529502	-3.618765	-0.23956	-2.498918	-3.663265	0.99661
40	4.62809	-3.480458	0.867347	-5.04753	-3.180999	-0.08687	-4.651377	-3.607332	-0.083722
41	5.925257	-0.679144	0.958551	-6.399278	0.339253	-0.640141	-5.997849	-0.871955	-0.644467
42	6.218722	-0.433701	-0.780817	-6.929318	-1.34835	-0.475863	-6.625378	-1.962964	0.608663
43	6.640479	-1.980653	-0.016027	-6.018742	-0.843256	-1.915993	-6.177796	-0.296506	1.03134
44	3.490628	-2.612562	-2.040953	-5.607571	-1.599189	1.824811	-4.933754	-2.95025	2.388921
45	4.969261	-1.73085	-2.459355	-5.410695	0.149267	1.586674	-4.795383	-1.236724	2.837606
46	5.076023	-3.322252	-1.676705	-3.992162	-0.86663	1.896187	-3.337075	-2.183006	2.496353
47	3.561822	-1.105025	1.977851	-3.701716	-1.991015	-2.270602	-3.70991	-1.517415	-1.742891
48	1.608333	2.488157	-1.419593	-1.33239	2.764968	-1.429168	-3.076623	1.849654	1.25764
49	3.13607	1.612418	-1.545545	-2.854584	2.063924	-1.968295	-1.557664	2.682809	0.917874
50	1.8827	2.889279	0.929295	-2.97492	4.009988	-0.363556	-1.954249	2.684649	-1.457876
51	5.603943	2.952491	0.478167	-0.186271	3.128724	0.742233	-5.531519	4.477067	0.081912
52	5.113767	2.702302	-1.192903	-1.008193	4.655661	1.043182	-5.048138	2.950588	0.849928
53	5.601318	4.32229	-0.653307	-0.530486	3.621707	2.408163	-5.637744	2.942801	-0.807588
54	3.743151	5.822722	-0.238431	-2.293068	2.284752	3.341541	-2.10375	5.019859	-1.256418
55	2.119259	5.15641	0.353532	-3.77925	1.838086	2.328947	-3.671567	5.827363	-0.689816
56	3.934688	0.996867	0.775139	-4.354401	1.607106	-0.091364	-4.024632	0.901887	-0.862758
57	-6.358898	-2.25063	-0.074478	5.928394	-2.295005	1.245388	6.303567	-2.130334	-0.876834
58	-5.620071	3.913275	0.268465	7.346379	2.398919	-1.438897	5.730768	3.670191	1.288678
	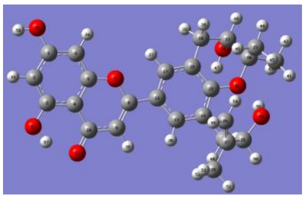 Conformer 16			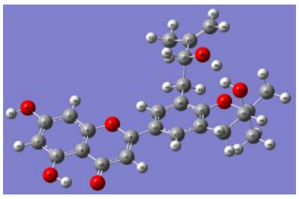 Conformer 17			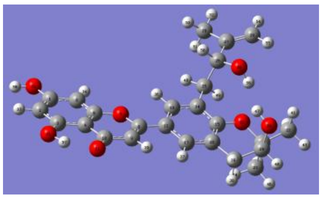 Conformer 18		
1	5.699359	-1.852253	0.880955	-5.709894	1.957125	0.128087	5.78189	1.814206	0.6501
2	6.472929	-0.777692	0.417805	-6.581447	0.857924	0.117396	6.619852	0.7897	0.184686
3	5.843756	0.318204	-0.16105	-6.059391	-0.428725	0.056096	6.060704	-0.408992	-0.243058
4	4.428687	0.353031	-0.284789	-4.654238	-0.633373	0.003738	4.652782	-0.599027	-0.210379
5	3.701123	-0.750037	0.194655	-3.825784	0.502076	0.012551	3.859264	0.460566	0.261727
6	4.304014	-1.854802	0.77635	-4.320933	1.795794	0.074533	4.391254	1.66592	0.693302
7	2.335234	-0.76909	0.108313	-2.464166	0.366261	-0.033994	2.497842	0.33285	0.322851
8	1.652396	0.26501	-0.455479	-1.884104	-0.863362	-0.108948	1.881067	-0.813161	-0.077706
9	2.302179	1.356644	-0.951419	-2.634342	-2.002019	-0.134425	2.594721	-1.87108	-0.558924
10	3.7337	1.47179	-0.887204	-4.069311	-1.956855	-0.066864	4.028867	-1.831087	-0.648057
11	4.353426	2.478935	-1.334218	-4.782359	-3.000799	-0.080865	4.708623	-2.802961	-1.086043
12	0.205859	0.039776	-0.458661	-0.424051	-0.78806	-0.169623	0.426731	-0.739603	0.072659
13	-0.685075	1.088483	-0.748675	0.216886	0.424341	-0.485309	-0.208636	0.502841	0.255454
14	-2.059895	0.893056	-0.763884	1.60014	0.509545	-0.572507	-1.586115	0.596603	0.40444

15	-2.55257	-0.393024	-0.46498	2.359015	-0.655394	-0.345111	-2.343322	-0.59119	0.392856
16	-1.692113	-1.465402	-0.186777	1.753336	-1.869565	0.01531	-1.744099	-1.843503	0.185795
17	-0.31861	-1.23034	-0.183442	0.363804	-1.916864	0.096261	-0.361182	-1.898258	0.029575
18	-2.248928	-2.840923	0.101235	2.605713	-3.068944	0.359285	-2.596584	-3.088596	0.105778
19	-3.710351	-2.960545	-0.334027	4.039351	-2.642931	0.679854	-4.060731	-2.734741	-0.162039
20	-4.510273	-1.730795	0.133668	4.573351	-1.698687	-0.413608	-4.526218	-1.621383	0.794821
21	-3.915854	-0.546402	-0.507336	3.716962	-0.507672	-0.433491	-3.69588	-0.438979	0.540787
22	-5.944786	-1.750913	-0.370466	5.958423	-1.163154	-0.082292	-5.94439	-1.160578	0.492823
23	-4.456081	-1.546937	1.649883	4.541519	-2.334099	-1.802015	-4.370943	-2.009523	2.263951
24	-3.821594	-3.14832	-1.7475	4.126015	-2.038673	1.974116	-4.264693	-2.362749	-1.52885
25	-2.995791	2.042195	-1.053143	2.291608	1.819258	-0.84991	-2.274895	1.931587	0.520903
26	-3.587509	2.708908	0.214662	2.761422	2.529468	0.44689	-2.828013	2.43607	-0.837885
27	-2.502244	3.247281	1.127379	3.555474	3.783686	0.120032	-3.610635	3.726635	-0.658301
28	-1.76842	4.460345	0.620137	2.809064	4.858773	-0.62727	-2.829179	4.900978	-0.127447
29	-2.208866	2.665751	2.293349	4.83509	3.917119	0.479343	-4.909901	3.806405	-0.958197
30	-4.451114	1.814701	0.919374	3.464696	1.638455	1.313184	-3.574151	1.423841	-1.514899
31	6.585049	1.353746	-0.605875	-6.895322	-1.487361	0.045419	6.863647	-1.396278	-0.690881
32	6.271083	-2.942096	1.454179	-6.174235	3.231856	0.188834	6.281129	3.000365	1.083318
33	7.554638	-0.795096	0.508241	-7.656414	1.004335	0.158005	7.696788	0.924146	0.158041
34	3.715626	-2.689451	1.138392	-3.655941	2.651168	0.082519	3.753191	2.463154	1.055515
35	1.746909	2.159695	-1.418256	-2.155599	-2.968589	-0.224732	2.086275	-2.764613	-0.897551
36	-0.308251	2.084631	-0.953019	-0.374592	1.313503	-0.674313	0.381315	1.412679	0.269501
37	0.349771	-2.058412	0.025878	-0.101081	-2.848832	0.402219	0.099188	-2.870197	-0.117328
38	-2.166975	-3.060165	1.172877	2.187561	-3.589983	1.226586	-2.521296	-3.665829	1.035259
39	-1.661642	-3.603525	-0.420663	2.611428	-3.787762	-0.469023	-2.237208	-3.738811	-0.698403
40	-4.158848	-3.852604	0.110559	4.689082	-3.520508	0.726645	-4.691438	-3.613386	-0.004746
41	-6.45347	-0.821218	-0.0955	5.975344	-0.710914	0.912201	-6.05154	-0.885319	-0.559085
42	-6.483588	-2.587513	0.086274	6.686285	-1.980802	-0.10605	-6.198282	-0.296658	1.115378
43	-5.976055	-1.86657	-1.456327	6.256742	-0.412356	-0.820999	-6.650198	-1.967485	0.715533
44	-4.804748	-2.457511	2.148508	5.115549	-3.266864	-1.803695	-3.323684	-2.164938	2.537098
45	-5.104966	-0.719032	1.950787	4.98899	-1.650739	-2.530766	-4.774487	-1.21509	2.899831
46	-3.443678	-1.329087	2.000358	3.521336	-2.556781	-2.126038	-4.922257	-2.932937	2.471017
47	-3.502982	-2.340284	-2.185844	3.614882	-1.210426	1.960904	-3.799713	-1.522977	-1.689532
48	-2.459648	2.805719	-1.623422	1.608144	2.485436	-1.382804	-1.569119	2.672417	0.90541
49	-3.832995	1.70745	-1.675906	3.165199	1.658562	-1.491585	-3.107552	1.868362	1.230503
50	-4.186387	3.563654	-0.138281	1.863993	2.827324	1.006975	-1.968514	2.643969	-1.490447
51	-1.22196	4.24322	-0.306992	3.388641	5.786189	-0.669142	-2.539855	4.754213	0.921231
52	-2.466827	5.275731	0.38788	1.844674	5.075436	-0.148259	-3.413436	5.824455	-0.187384
53	-1.046782	4.824553	1.357738	2.586501	4.555443	-1.658485	-1.898457	5.043724	-0.693026
54	-2.748684	1.790951	2.641368	5.357279	3.137311	1.025214	-5.463341	4.733736	-0.828324
55	-1.421666	3.053941	2.935753	5.396832	4.817978	0.24262	-5.456731	2.953726	-1.348885
56	-4.554329	1.011163	0.378268	4.005406	1.047727	0.757438	-4.084341	0.929532	-0.847031
57	5.934581	2.018288	-0.979467	-6.309986	-2.299536	-0.002258	6.256373	-2.152312	-0.943728
58	7.238171	-2.831185	1.478807	-7.147195	3.228699	0.232236	7.252463	2.993001	1.019632

	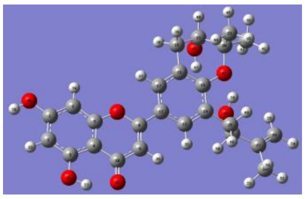			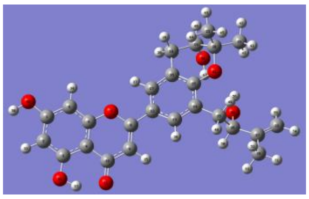			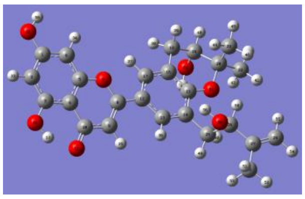		
	Conformer 19			Conformer 20			Conformer 21		
1	6.128805	-1.488581	-0.655988	-6.209981	-1.514312	-0.035683	6.078706	-1.421527	0.838376
2	6.791	-0.287175	-0.363555	-6.83028	-0.261469	0.07934	6.741818	-0.263299	0.404852
3	6.052917	0.822108	0.032112	-6.047976	0.88706	0.118241	6.004467	0.790296	-0.119118
4	4.638403	0.744708	0.138032	-4.631914	0.79671	0.042578	4.588053	0.701356	-0.216524
5	4.024071	-0.482113	-0.167102	-4.061934	-0.483172	-0.069908	3.9742	-0.480294	0.229122
6	4.737061	-1.603436	-0.563525	-4.818945	-1.643896	-0.111933	4.688026	-1.547802	0.755816
7	2.664057	-0.61429	-0.085753	-2.703732	-0.632075	-0.151035	2.614845	-0.623464	0.166141
8	1.878725	0.426078	0.307932	-1.875134	0.447654	-0.106202	1.828499	0.364482	-0.344123
9	2.417475	1.637167	0.630422	-2.36644	1.71405	0.016606	2.365054	1.531006	-0.80401
10	3.83296	1.873672	0.555263	-3.77989	1.967217	0.08745	3.781011	1.775295	-0.758865
11	4.349899	2.991157	0.842666	-4.255657	3.133696	0.192718	4.296917	2.851855	-1.17473
12	0.45896	0.072175	0.341987	-0.464516	0.067285	-0.191202	0.410262	0.005643	-0.339088
13	-0.52745	1.067367	0.480128	0.523328	1.021796	-0.498496	-0.581099	0.988892	-0.520292
14	-1.876086	0.7475	0.533381	1.864594	0.674947	-0.575643	-1.930835	0.66176	-0.538505
15	-2.246684	-0.610967	0.458749	2.224008	-0.670143	-0.352256	-2.297781	-0.689167	-0.354414
16	-1.293599	-1.623308	0.276485	1.268448	-1.640048	-0.015855	-1.333173	-1.692723	-0.175855
17	0.052259	-1.264051	0.222487	-0.069383	-1.255177	0.053612	0.011954	-1.327326	-0.168333
18	-1.73683	-3.056272	0.094875	1.703436	-3.048127	0.3166	-1.757935	-3.13303	-0.003651
19	-3.214062	-3.121664	-0.29758	3.193482	-3.086988	0.660459	-3.206196	-3.346921	-0.448878
20	-4.057749	-2.247007	0.648273	4.009355	-2.34462	-0.414619	-4.113223	-2.239046	0.120496
21	-3.591073	-0.863306	0.507836	3.562089	-0.947584	-0.43029	-3.63474	-0.955114	-0.401219
22	-5.520488	-2.197017	0.231896	5.487081	-2.26562	-0.06034	-5.541598	-2.346199	-0.392068
23	-3.912632	-2.655905	2.112826	3.804748	-2.927345	-1.811583	-4.077235	-2.187408	1.647409
24	-3.406611	-2.738927	-1.663269	3.440786	-2.548474	1.963467	-3.305406	-3.41803	-1.874656
25	-2.936495	1.816233	0.597261	2.93023	1.70618	-0.841924	-2.994859	1.720771	-0.67184
26	-3.506529	2.168571	-0.802108	3.588238	2.229173	0.462299	-3.600532	2.095291	0.702191
27	-4.652442	3.160301	-0.686563	4.742179	3.166714	0.147674	-4.719365	3.104214	0.561438
28	-4.302952	4.501743	-0.095087	4.383779	4.418752	-0.610661	-4.334189	4.52243	0.233797
29	-5.888716	2.854802	-1.089873	5.993011	2.890578	0.526381	-5.990128	2.711547	0.692953
30	-3.857155	0.998527	-1.541984	3.962842	1.161169	1.332763	-2.596588	2.659381	1.558743
31	6.688149	1.977348	0.317445	-6.641165	2.09342	0.230407	6.637741	1.905906	-0.537865
32	6.808337	-2.59897	-1.042872	-6.932582	-2.662943	-0.078577	6.857752	-2.41325	1.342005
33	7.871602	-0.216461	-0.440598	-7.911332	-0.181717	0.140446	7.821311	-0.193416	0.480323
34	4.234525	-2.535675	-0.792427	-4.350183	-2.616619	-0.200881	4.175777	-2.44283	1.091983
35	1.782798	2.447462	0.964972	-1.691911	2.558348	0.079797	1.727965	2.294107	-1.232182
36	-0.245981	2.114206	0.526848	0.245671	2.051795	-0.697805	-0.303327	2.031574	-0.633873
37	0.792318	-2.044343	0.082047	-0.810396	-1.998978	0.326195	0.76043	-2.102566	-0.043028
38	-1.14038	-3.536619	-0.687556	1.132141	-3.424755	1.171207	-1.108206	-3.792529	-0.588384

39	-1.573119	-3.631039	1.014458	1.499975	-3.724472	-0.522354	-1.647892	-3.433927	1.045569
40	-3.573752	-4.1516	-0.231898	3.540692	-4.122272	0.70517	-3.568105	-4.312616	-0.086801
41	-6.061715	-1.474762	0.851427	6.012495	-1.633523	-0.783395	-6.128898	-1.4905	-0.043308
42	-5.977748	-3.1824	0.369182	5.928323	-3.267251	-0.091028	-5.564591	-2.370474	-1.484085
43	-5.619205	-1.910913	-0.818031	5.628029	-1.85385	0.941906	-6.00441	-3.262749	-0.011813
44	-2.892063	-2.514001	2.478274	2.772248	-2.816066	-2.153494	-3.086809	-1.918788	2.025153
45	-4.583022	-2.051659	2.732343	4.457004	-2.41327	-2.524752	-4.351595	-3.162453	2.064232
46	-4.180848	-3.710312	2.238846	4.057197	-3.993103	-1.816945	-4.794081	-1.443833	2.010214
47	-3.187776	-1.794256	-1.750017	3.222713	-1.599733	1.950191	-3.08392	-2.541792	-2.235134
48	-2.513974	2.722835	1.038273	2.490871	2.554441	-1.373242	-3.804744	1.369179	-1.318332
49	-3.764434	1.491707	1.23726	3.714483	1.284971	-1.480745	-2.563308	2.617981	-1.125676
50	-2.70011	2.641852	-1.379484	2.8258	2.796123	1.014606	-4.002923	1.181894	1.157943
51	-5.129303	5.210538	-0.206963	5.228443	5.113627	-0.648807	-3.71028	4.952327	1.026481
52	-4.069474	4.424919	0.974778	4.085845	4.195208	-1.643228	-5.220294	5.153024	0.112442
53	-3.414558	4.927653	-0.580745	3.533717	4.933665	-0.142979	-3.746254	4.575716	-0.691529
54	-6.702995	3.570949	-1.004706	6.814082	3.56662	0.29843	-6.818773	3.401611	0.548924
55	-6.123917	1.887343	-1.522923	6.234007	1.988029	1.079582	-6.246718	1.683211	0.94012
56	-4.24726	0.357037	-0.9204	4.302376	0.431897	0.782001	-1.881924	2.003486	1.632896
57	5.972106	2.627873	0.579082	-5.898229	2.766219	0.242096	5.92112	2.521319	-0.872416
58	7.763949	-2.41222	-1.06895	-7.881845	-2.45622	-0.017223	6.302886	-3.164696	1.617627
	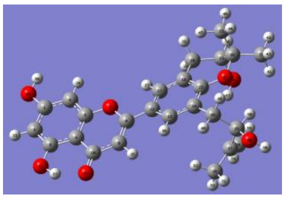			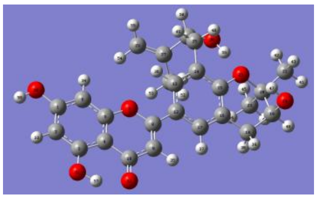			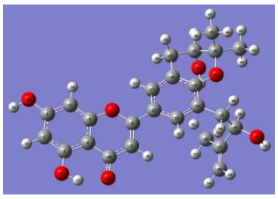		
	Conformer 22			Conformer 23			Conformer 24		
1	-5.936937	-1.817381	0.196127	5.682218	1.594709	-0.254393	-5.882015	-1.847213	0.212
2	-6.630719	-0.598352	0.161347	6.458308	0.447502	-0.030945	-6.578295	-0.630247	0.162095
3	-5.921339	0.587493	0.026595	5.830891	-0.782442	0.129455	-5.871419	0.556029	0.0025
4	-4.502797	0.571303	-0.076364	4.414651	-0.881494	0.070753	-4.455155	0.540347	-0.110404
5	-3.856781	-0.674884	-0.03286	3.684768	0.297985	-0.157164	-3.806746	-0.705359	-0.052766
6	-4.542286	-1.874647	0.101696	4.285617	1.536546	-0.321452	-4.487848	-1.902553	0.105689
7	-2.494598	-0.754839	-0.134559	2.317731	0.264898	-0.219733	-2.444751	-0.782939	-0.162836
8	-1.733126	0.367633	-0.261684	1.636344	-0.905623	-0.081446	-1.685861	0.338928	-0.305826
9	-2.299797	1.608221	-0.291579	2.287588	-2.084617	0.130493	-2.254912	1.5781	-0.35017
10	-3.723812	1.785306	-0.212966	3.720822	-2.143204	0.229732	-3.679122	1.753501	-0.267738
11	-4.268628	2.925584	-0.250175	4.34261	-3.225172	0.431403	-4.226578	2.892074	-0.318158
12	-0.305106	0.063963	-0.344759	0.188718	-0.720342	-0.200216	-0.257043	0.035184	-0.385119
13	0.610221	1.028755	-0.805859	-0.33264	0.465952	-0.747511	0.663873	1.002834	-0.827996
14	1.970993	0.766451	-0.876852	-1.701561	0.668736	-0.875669	2.024121	0.735125	-0.885915
15	2.426821	-0.512406	-0.492214	-2.56679	-0.345824	-0.423317	2.476079	-0.543109	-0.498187
16	1.541626	-1.491191	-0.01055	-2.08124	-1.551485	0.105231	1.584076	-1.5282	-0.044259
17	0.183414	-1.187323	0.054225	-0.702638	-1.719148	0.213781	0.225797	-1.22206	0.005
18	2.070458	-2.825814	0.462653	-3.038604	-2.628559	0.562193	2.10728	-2.865019	0.426676

19	3.569434	-2.74483	0.75242	-4.45122	-2.396864	0.021919	3.597156	-2.778432	0.761112
20	4.30311	-2.068069	-0.422051	-4.861163	-0.924318	0.205253	4.362873	-2.084428	-0.382098
21	3.772011	-0.711548	-0.573401	-3.909319	-0.097997	-0.5558	3.826509	-0.728303	-0.530373
22	5.783879	-1.868262	-0.133861	-6.209673	-0.614278	-0.425867	5.831501	-1.875554	-0.042615
23	4.095669	-2.813409	-1.740357	-4.820793	-0.487948	1.669149	4.201691	-2.812931	-1.715501
24	3.823765	-2.070468	1.989403	-4.562558	-2.805447	-1.344302	3.809124	-2.113287	2.010813
25	2.941385	1.829546	-1.326624	-2.229416	1.957462	-1.463988	3.008558	1.801998	-1.283941
26	3.92544	2.286728	-0.22903	-2.780854	2.9925	-0.449575	3.801758	2.394664	-0.098845
27	3.253922	2.547412	1.104563	-1.827315	3.200831	0.721461	2.896339	2.865535	1.030188
28	2.26024	3.675338	1.164846	-2.054459	2.380283	1.961877	2.656623	1.883112	2.146054
29	3.546766	1.777861	2.158584	-0.816306	4.068385	0.609892	2.360046	4.089746	1.015769
30	4.54681	3.474669	-0.751863	-4.106701	2.665931	-0.01531	4.572083	3.457465	-0.672258
31	-6.584954	1.761924	-0.0085	6.575973	-1.88685	0.341458	-6.538711	1.727382	-0.045023
32	-6.691927	-2.93886	0.324015	6.248253	2.818532	-0.415656	-6.528426	-3.031377	0.365361
33	-7.712357	-0.585377	0.237137	7.541065	0.511476	0.016998	-7.660513	-0.607751	0.246614
34	-4.007102	-2.817726	0.128235	3.694524	2.428124	-0.493639	-3.959277	-2.847529	0.144253
35	-1.678363	2.491847	-0.360778	1.731757	-3.00975	0.211782	-1.636061	2.462295	-0.433076
36	0.257313	2.000963	-1.132513	0.341551	1.244758	-1.084188	0.320918	1.983272	-1.140115
37	-0.499356	-1.93625	0.441346	-0.331376	-2.642591	0.646901	-0.462718	-1.973893	0.375535
38	1.548011	-3.136446	1.373364	-3.066571	-2.663518	1.658029	1.561272	-3.190511	1.318033
39	1.882137	-3.60181	-0.288983	-2.690388	-3.610841	0.226406	1.945904	-3.632356	-0.340255
40	3.981894	-3.74861	0.882579	-5.16339	-3.02171	0.566912	4.010042	-3.781486	0.89507
41	5.932118	-1.329017	0.804545	-6.998692	-1.159561	0.102085	5.941373	-1.341049	0.904012
42	6.281534	-2.840864	-0.059864	-6.420022	0.457707	-0.352326	6.334044	-2.844637	0.042653
43	6.250503	-1.302164	-0.946483	-6.225271	-0.90771	-1.478019	6.320996	-1.299769	-0.834644
44	4.688375	-2.338884	-2.528985	-3.813809	-0.54974	2.089826	4.814142	-2.323349	-2.47943
45	3.047762	-2.80652	-2.052339	-5.163502	0.547672	1.756639	4.531256	-3.853255	-1.621467
46	4.420015	-3.855049	-1.642677	-5.482987	-1.123444	2.266526	3.163641	-2.809965	-2.058918
47	3.425243	-1.184016	1.942434	-3.994223	-2.226134	-1.88092	3.514968	-1.190372	1.917416
48	3.537457	1.476469	-2.176713	-3.025285	1.751553	-2.190086	3.732949	1.408651	-2.006154
49	2.377599	2.705625	-1.662466	-1.411843	2.442074	-2.005352	2.472007	2.630099	-1.75836
50	4.684736	1.511364	-0.0893	-2.867746	3.939429	-0.994557	4.476617	1.622991	0.295426
51	2.707533	4.608013	0.801456	-3.030717	2.60823	2.405662	2.207542	0.9527	1.778601
52	1.898797	3.835185	2.185263	-2.053174	1.306676	1.741584	3.605522	1.606046	2.625909
53	1.392071	3.471516	0.524712	-1.279678	2.577582	2.709402	1.993661	2.30072	2.910641
54	3.069839	1.923058	3.12577	-0.088711	4.205821	1.40678	1.708718	4.435456	1.815177
55	4.273273	0.97059	2.087659	-0.677209	4.670397	-0.286633	2.564025	4.78903	0.209025
56	5.240867	3.733063	-0.12219	-4.207621	1.699316	-0.096674	5.038826	3.898662	0.057651
57	-5.885539	2.472013	-0.11046	5.927089	-2.64551	0.424461	-5.841545	2.437773	-0.161658
58	-6.118263	-3.725312	0.327214	7.216808	2.750073	-0.341939	-7.488451	-2.879882	0.423618

	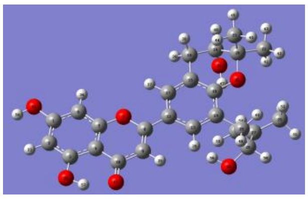<br>Conformer 25		
1	-5.849748 -1.824702 0.684439		
2	-6.623253 -0.691924 0.389382		
3	-5.99443 0.471261 -0.040353		
4	-4.580969 0.516493 -0.179043		
5	-3.854415 -0.646653 0.127077		
6	-4.456423 -1.819383 0.556144		
7	-2.490955 -0.661874 0.013047		
8	-1.804885 0.440012 -0.399323		
9	-2.454463 1.598218 -0.712723		
10	-3.884491 1.707882 -0.619307		
11	-4.502769 2.773234 -0.904942		
12	-0.361497 0.205596 -0.454683		
13	0.534116 1.253791 -0.740791		
14	1.907324 1.045432 -0.803825		
15	2.398404 -0.263568 -0.603044		
16	1.53336 -1.320101 -0.273226		
17	0.165091 -1.068747 -0.203786		
18	2.091916 -2.688657 0.039733		
19	3.590776 -2.612431 0.331456		
20	4.299476 -1.78028 -0.752675		
21	3.747096 -0.423047 -0.70087		
22	5.782399 -1.608201 -0.457846		
23	4.080558 -2.338303 -2.158087		
24	3.84082 -2.078991 1.636149		
25	2.846685 2.210444 -0.978564		
26	3.206852 2.87448 0.383091		
27	4.054865 1.979024 1.270959		
28	5.504541 1.874802 0.879131		
29	3.552319 1.346223 2.336953		
30	2.029286 3.360779 1.02596		
31	-6.733975 1.562573 -0.326055		
32	-6.415139 -2.983303 1.111068		
33	-7.703605 -0.716472 0.494837		
34	-3.868925 -2.699915 0.787299		
35	-1.90111 2.465346 -1.048264		
36	0.164643 2.259544 -0.909662		
37	-0.498166 -1.887246 0.053016		
38	1.91257 -3.377669 -0.794485		

39	1.581869	-3.110863	0.911925		
40	4.021906	-3.6168	0.339708		
41	5.939637	-1.211033	0.546936		
42	6.235566	-0.928034	-1.185744		
43	6.285109	-2.578161	-0.532688		
44	3.028681	-2.303991	-2.453923		
45	4.656607	-1.751425	-2.880739		
46	4.419338	-3.378631	-2.208521		
47	3.603866	-1.133442	1.631163		
48	2.367204	2.979497	-1.592291		
49	3.763887	1.902175	-1.485771		
50	3.791084	3.77328	0.15296		
51	5.991295	2.857011	0.954857		
52	6.043667	1.175102	1.524403		
53	5.622384	1.543624	-0.158503		
54	4.180091	0.716179	2.963695		
55	2.516015	1.45573	2.64346		
56	1.410264	2.615634	1.11839		
57	-6.082463	2.270197	-0.607541		
58	-7.380985	-2.878861	1.17411		

^{13}C NMR calculation parts

Table S16. Energies and populations of conformers of the calculated configuration of **3** at B3LYP/6-311G(d,p) theory level.

(2''*S*,2'''*S*)-**3**		
Conformer	E (Hartree)	Population (%)
1	-1494.612558	3.19
2	-1494.612189	2.16
3	-1494.612023	1.81
4	-1494.613704	10.73
5	-1494.611822	1.46
6	-1494.612966	4.91
7	-1494.61268	3.63
8	-1494.612606	3.35
9	-1494.612125	2.01
10	-1494.61299	5.04
11	-1494.612396	2.68
12	-1494.612599	3.33
13	-1494.6136	9.61
14	-1494.612013	1.79
15	-1494.612597	3.32

16	-1494.61408	15.97
17	-1494.612541	3.13
18	-1494.612139	2.04
19	-1494.612362	2.59
20	-1494.613039	5.30
21	-1494.61302	5.20
22	-1494.613268	6.76
(2"*R*,2"'*S*)-**3**		
Conformer	E (Hartree)	Population (%)
1	-1494.613102	9.24
2	-1494.611477	1.65
3	-1494.613125	9.47
4	-1494.612218	3.62
5	-1494.613983	23.50
6	-1494.611821	2.38
7	-1494.611219	1.26
8	-1494.612792	6.66
9	-1494.611801	2.33
10	-1494.614342	34.37
11	-1494.612175	3.46
12	-1494.611676	2.04

TableS17. Energies and populations of conformers of the calculated configuration of **6** at B3LYP/6-311G(d,p) theory level.

(2"*S*,2"'*R*)-**6**		
Conformer	E (Hartree)	Population (%)
1	-1494.615425	3.09
2	-1494.61551	3.38
3	-1494.614566	1.24
4	-1494.61482	1.63
5	-1494.615026	2.03
6	-1494.616573	10.43
7	-1494.616788	13.09
8	-1494.616464	9.29
9	-1494.615625	3.82
10	-1494.615216	2.48
11	-1494.615455	3.19
12	-1494.615627	3.83
13	-1494.615215	2.47
14	-1494.616781	13.00
15	-1494.6153	2.71
16	-1494.615306	2.73

17	-1494.615121	2.24
18	-1494.615049	2.08
19	-1494.615168	2.35
20	-1494.615002	1.97
21	-1494.614617	1.31
22	-1494.61464	1.35
23	-1494.614751	1.51
24	-1494.614704	1.44
25	-1494.616241	7.34
(2''*R*,2'''*R*)-**6**		
Conformer	E (Hartree)	Population (%)
1	-1494.614858	1.98
2	-1494.614937	2.15
3	-1494.614763	1.79
4	-1494.615094	2.54
5	-1494.615394	3.49
6	-1494.61436	1.17
7	-1494.615327	3.25
8	-1494.615284	3.10
9	-1494.614956	2.19
10	-1494.614307	1.10
11	-1494.617064	20.44
12	-1494.616423	10.37
13	-1494.615129	2.63
14	-1494.61501	2.32
15	-1494.614824	1.91
16	-1494.616429	10.43
17	-1494.614974	2.23
18	-1494.614751	1.76
19	-1494.614996	2.29
20	-1494.615124	2.62
21	-1494.615045	2.41
22	-1494.61433	1.13
23	-1494.614886	2.04
24	-1494.615247	2.98
25	-1494.614615	1.53
26	-1494.614965	2.21
27	-1494.615853	5.67
28	-1494.614988	2.27
